# Smurf2-mediated degradation of EZH2 enhances neuron differentiation and improves functional recovery after ischaemic stroke

**DOI:** 10.1002/emmm.201201783

**Published:** 2013-03-25

**Authors:** Yung-Luen Yu, Ruey-Hwang Chou, Woei-Cherng Shyu, Shu-Ching Hsieh, Chen-Shiou Wu, Shu-Ya Chiang, Wei-Jung Chang, Jia-Ni Chen, Yen-Ju Tseng, Yu-Hsuan Lin, Wei Lee, Su-Peng Yeh, Jennifer L Hsu, Cheng-Chieh Yang, Shih-Chieh Hung, Mien-Chie Hung

**Affiliations:** 1 Graduate Institute of Cancer Biology, Center for Molecular Medicine, China Medical UniversityTaichung, Taiwan; 2 Department of Biotechnology, Asia UniversityTaichung, Taiwan; 3 Graduate Institute of Immunology, Translational Medicine Research Center, Center for Neuropsychiatry, China Medical UniversityTaichung, Taiwan; 4 The Ph.D. Program for Cancer Biology and Drug Discovery, China Medical UniversityTaichung, Taiwan; 5 Division of Hematology and Oncology, Department of Medicine, China Medical University HospitalTaichung, Taiwan; 6 Department of Molecular and Cellular Oncology, The University of Texas MD Anderson Cancer CenterHouston, TX, USA; 7 Department of Stomatology, Taipei Veteran General Hospital, and Dental School, National Yang-Ming UniversityTaipei, Taiwan; 8 Stem Cell Laboratory, Department of Medical Research and Education, Orthopaedics and Traumatology, Taipei Veterans General Hospital, Institute of Clinical Medicine, Institute of Pharmacology, National Yang-Ming UniversityTaipei, Taiwan

**Keywords:** EZH2, human mesenchymal stem cells (hMSCs), ischaemic neuronal injury, neuron differentiation, Smurf2

## Abstract

EZH2 plays an important role in stem cell renewal and maintenance by inducing gene silencing via its histone methyltransferase activity. Previously, we showed that EZH2 downregulation enhances neuron differentiation of human mesenchymal stem cells (hMSCs); however, the underlying mechanisms of EZH2-regulated neuron differentiation are still unclear. Here, we identify Smurf2 as the E3 ubiquitin ligase responsible for the polyubiquitination and proteasome-mediated degradation of EZH2, which is required for neuron differentiation. A ChIP-on-chip screen combined with gene microarray analysis revealed that PPARγ was the only gene involved in neuron differentiation with significant changes in both its modification and expression status during differentiation. Moreover, knocking down PPARγ prevented cells from undergoing efficient neuron differentiation. In animal model, rats implanted with intracerebral EZH2-knocked-down hMSCs or hMSCs plus treatment with PPARγ agonist (rosiglitazone) showed better improvement than those without EZH2 knockdown or rosiglitazone treatment after a stroke. Together, our results support Smurf2 as a regulator of EZH2 turnover to facilitate PPARγ expression, which is specifically required for neuron differentiation, providing a molecular mechanism for clinical applications in the neurodegenerative diseases.

## INTRODUCTION

Functional recovery after a neurogenic response injury of the brain and spinal cord is very limited and insufficient. Human mesenchymal stem cells (hMSCs) exhibit a wide range of flexibility similar to that of other adult stem cell populations, which have intrinsic neurogenic potential for differentiation into neural cells both *in vitro* and *in vivo* (Brazelton et al, 2000; Ding et al, 2007; Kabos et al, 2002; Nakano et al, 2001; Sanchez-Ramos et al, 2000). Recently, transplantation of bone marrow-derived MSCs was reported to improve recovery of the injured brain and spinal cord in animal models (Croft & Przyborski, 2009; Gu et al, 2010). However, the molecular mechanisms associated with MSCs ability to directly differentiate or indirectly improve regeneration of the central nervous system (CNS) and repair injured brain and spinal cord remain elusive.

Polycomb group (PcG) proteins are transcription repressors that form chromatin-remodelling complexes. Two of the major PcG complexes are polycomb repressive complex 1 (PRC1) and 2 (PRC2; Ringrose & Paro, 2004; Schwartz & Pirrotta, 2007). PRC2 contains suppressor of zeste 12 (SUZ12), embryonic ectoderm development (EED), and a methyltransferase, enhancer of zeste homolog 2 (EZH2). EZH2 catalyses histone H3 trimethylation of lysine 27 (H3K27me3) to provide a platform to recruit PRC1 for PcG-mediated epigenetic gene silencing (Cao & Zhang, 2004; Cao et al, 2002; Min et al, 2003).

The PcG proteins have been demonstrated to dynamically bind to their target genes in embryonic stem cells (ESCs) during subsequent cell lineage commitment events (Boyer et al, 2006; Lee et al, 2006). The importance of PcG proteins in ESCs is well illustrated by several PcG knockout mouse models in which ESCs lacking *EED* or *SUZ12* are not able to maintain their pluripotency and are prone to differentiation (Erhardt et al, 2003; Faust et al, 1995; Pasini et al, 2007); furthermore, *EZH2*-deficient ESCs cannot be established, which largely affects the propagation of the pluripotent state of ESCs and stepwise differentiation of tissue-specific stem cells (Erhardt et al, 2003; Ezhkova et al, 2009; O'Carroll et al, 2001). Interestingly, PcG proteins also regulate differentiation of a wide range of cell lineages (Chou et al, 2011). For example, recent reports identified a switch between adipogenesis and osteogenesis that can be epigenetically regulated by EZH2 (Chen et al, 2011; Wang et al, 2010), and suppression of the methyltransferase activity via phosphorylation of EZH2 at Thr 487 by cyclin-dependent kinase 1 (CDK1) results in hMSC differentiation into osteoblasts (Wei et al, 2011). In addition, a decrease in the binding of PcG proteins at neuron-specific genes occurs during ESC differentiation into neural precursor cells and neuron differentiation (Boyer et al, 2006; Bracken et al, 2006; Mikkelsen et al, 2007). Inactivation of PcG by *EZH2* knockout in ESCs markedly enhances neuron differentiation during neocortical development, demonstrating a role for EZH2 in the regulation of neural precursor cells' fate (Hirabayashi et al, 2009). Recently, we demonstrated that EZH2 also plays an important role in hMSC differentiation into functional neuron lineage (Yu et al, 2011).

Preferential removal of EZH2 from transcribed chromatin regions occurs through posttranscriptional (Juan et al, 2009; Wong & Tellam, 2008) or posttranslational (Kaneko et al, 2010; Wei et al, 2011; Wu & Zhang, 2011) regulation, but the underlying mechanisms are not fully understood. MicroRNAs have been shown to regulate posttranscriptional gene silencing and to play an important role in cellular differentiation and development of ESCs (Lewis et al, 2005; Marson et al, 2008). In particular, microRNAs miR-26a and miR-214 repress EZH2 posttranscriptionally during skeletal muscle cell and ESC differentiation and establish a regulatory loop controlling EZH2-dependent gene expression during differentiation (Juan et al, 2009; Wong & Tellam, 2008). Although posttranslational modifications of EZH2 have been shown to inactivate EZH2's transcriptional silencing function (Kaneko et al, 2010; Wei et al, 2011; Wu & Zhang, 2011), how these modifications regulate EZH2 is largely unknown.

In this study, we identified Smad ubiquitination regulatory factor-2 (Smurf2) as the ubiquitin E3 ligase responsible for proteasome-mediated degradation of EZH2, a process that is required for neuron differentiation. In addition, our behavioural measurements of neurological deficit after stroke in a rat model showed better improvement after intracerebral implantation of hMSC with EZH2 knockdown than after implantation of hMSCs without EZH2 knockdown. We also identified peroxisome proliferator-activated receptor gamma (PPARγ) as an EZH2 target gene during neuron differentiation. Upregulation of PPARγ via Smurf2-mediated degradation of EZH2 was accompanied by accelerated neuron differentiation of hMSCs. Together, a pathway that is critical for neuron differentiation is established, and modification of hMSCs to accelerate neuron differentiation may have important clinical implications in the regeneration of CNS repair of injured brain and spinal cord.

## RESULTS

### Downregulation of EZH2 promotes neuron differentiation of hMSCs

**Figure 1:**
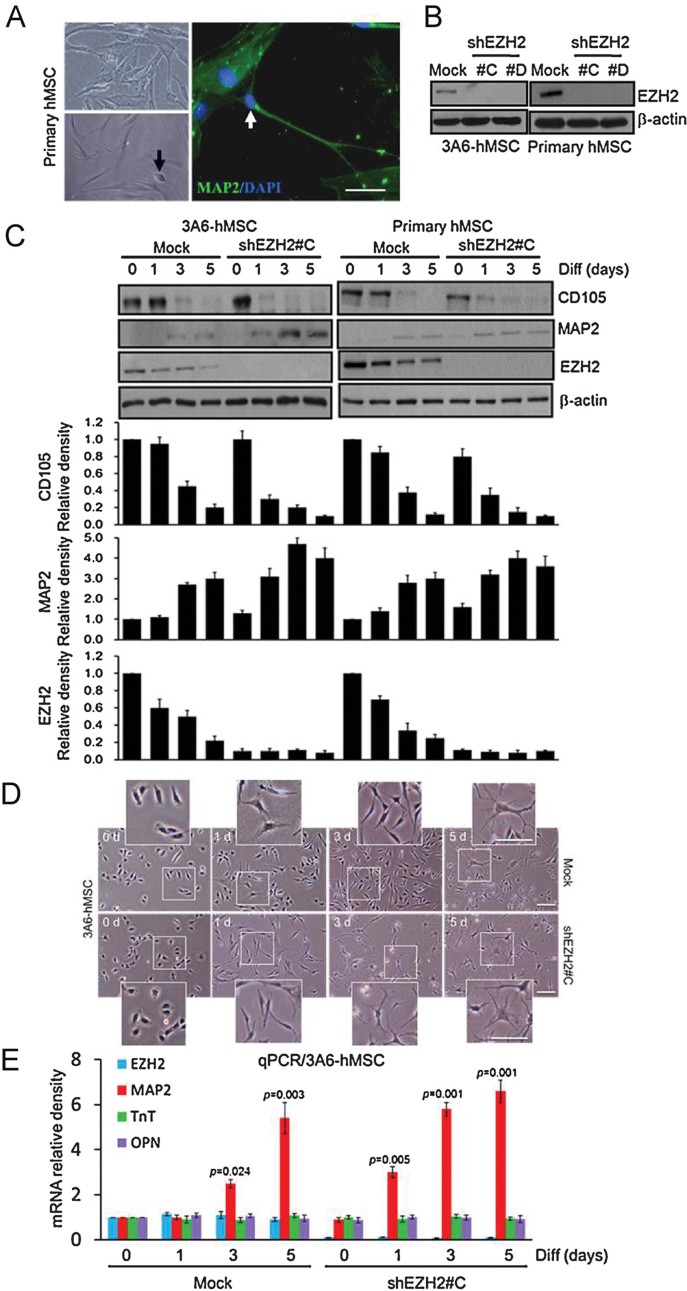
Downregulation of EZH2 promotes neuron differentiation of hMSCs. A.Representative undifferentiated primary hMSCs (top left) and differentiated neuron with cell body morphologies (black arrow) as well as extended neurite-like structures (bottom left), which shows MAP2 expression (white arrow) by immunocytochemical analysis (right). Scale bars: 50 µm. B. Lentiviral-mediated shRNA interference targeting EZH2 was used to allow for the generation of hMSCs stably expressing shEZH2 (shEZH2 #C and #D). C. The effects of 3A6-hMSCs and primary hMSCs stably expressing shRNA targeting EZH2 (shEZH2 #C) and treat with NIM for indicated time intervals during neuron differentiation (Diff). The total cell lysate at each time interval was extracted and immunoblotted with the indicated antibodies. The plots (bottom) represent the relative density of EZH2, MSC marker (CD105), and neuron marker (MAP2) determined by scanning densitometric tracings. Error bars represent the SEM from three independent experiments (*n* = 3). Source data is available for this figure in the Supporting Information. D. Cell morphology of 3A6-hMSCs with or without stably expressing shEZH2 (shEZH2 #C) and treat with NIM at indicated time interval during neuron differentiation was observed under an inverted phase microscope. The inset is an enlarged image of the boxed (white) region. Scale bars: 50 µm. E. Quantitative PCR analysis of EZH2, MAP2 (neuron marker), troponin T (TnT; cardiac marker), osteopontin (OPN; osteogenic marker) mRNA levels in NIM-treated 3A6-hMSCs at indicated time intervals during neuron differentiation (Diff). A change in the neuron marker (MAP2) was observed. Error bars represent the SEM from three independent experiments. Significant *p* values are indicated (*n* = 3, Student's *t*-test).

Previously, we reported that 3A6-hMSCs exhibit extension of neurite-like structures and successfully differentiate into functional neuron lineage after induction in the neuronal induction medium (NIM; Yu et al, 2011). Consistent with these observations, we showed here that primary bone marrow-derived hMSCs also exhibited cell body morphologies with extended neurite-like structures in NIM (Fig 1A, bottom left). The hMSC-derived neuronal cells were then stained with the MAP2 (neuron marker) for immunocytochemical analysis to further validate neuron differentiation with dendritic arborization (Fig 1A, right; green fluorescence). To determine how EZH2 regulates neuron differentiation of hMSCs, EZH2 was stably knocked down by two independent short-hairpin RNAs (shRNAs) in 3A6-hMSCs and primary hMSCs (Fig 1B). Under neuron differentiation culture condition in both 3A6-hMSCs and primary hMSCs, downregulation of EZH2 decreased CD105 (MSC marker) and increased MAP2 (neuron marker) protein expression (Fig 1C). We also observed an increase in neuron specific enolase mRNA expression (NSE; neuron marker; Supporting Information Fig S1). In addition, cell morphology analysis in EZH2 knockdown hMSCs in the NIM showed that neuron differentiation with dendritic arborization was enhanced, occurring at Day 3 compared to Day 5 in the mock (Fig 1D). To determine whether differentiation upon EZH2 knockdown is a general phenomenon or is limited to the neuronal lineage, we analysed expression of genes from other linages by quantitative PCR (qPCR). We found that EZH2 knockdown hMSCs are more prone to differentiation into the neuronal lineage as indicated by the increase in MAP2 (neuron marker) gene expression after induction in NIM but not expression of genes from other lineages examined (Fig 1E), such as troponin T (TnT; cardiac marker) and osteopontin (OPN; osteogenic marker). Together, these results suggest that knocking down EZH2 accelerates the differentiation of hMSCs preferentially to the neuronal lineage after induction in NIM.

### Degradation of EZH2 via ubiquitin-proteasome degradation pathway requires Smurf2

**Figure 2:**
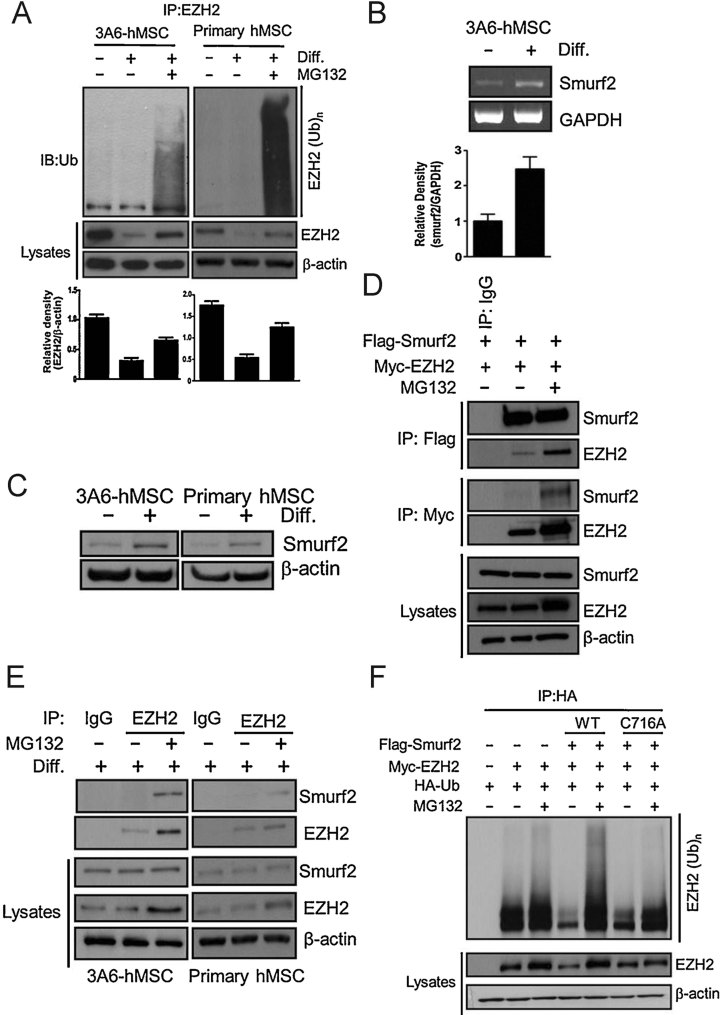
Degradation of EZH2 via the ubiquitin-proteasome-dependent degradation pathway requires Smurf2. Source data is available for this figure in the Supporting Information. A. 3A6-hMSCs were treated with or without NIM and/or proteasome inhibitor MG132 (5 µM) for 5 days. EZH2 was then immunoprecipitated by an EZH2 antibody and blotted with an anti-ubiquitin antibody (immunoblot, top). The plots (bottom) represent the relative density of EZH2 determined by scanning densitometric tracings. Error bars represent the SEM from three independent experiments (*n* = 3). B. RT-PCR analysis of Smurf2 mRNA expression during neuron differentiation on Day 1 of 3A6-hMSCs (RT-PCR, top). The plot (bottom) represents the relative density of Smurf2, determined by scanning densitometric tracings. Error bars represent the SEM from three independent experiments (*n* = 3). C. Immunoblotting analysis of Smurf2 protein levels during neuron differentiation on Day 1 of both 3A6 and primary hMSCs. D. Ectopic expression of Flag-Smurf2 and Myc-EZH2 in HEK 293 cells with or without MG132 and co-immunoprecipitation of Smurf2 and EZH2. E. Co-immunoprecipitation of endogenous Smurf2 and EZH2 in 3A6-hMSCs treated with NIM for 1 day with or without MG132. F. *In vivo* ubiquitination assay. Ectopic expression of HA-Ubiquitin (Ub), Myc-EZH2, Flag-Smurf2, or Flag-Smurf2-C716A (catalytically inactive mutant of Smurf2) in HEK 293 cells with or without MG132. Polyubiquitinated EZH2 was detected by immunoprecipitation of HA-tagged ubiquitin followed by immunoblotting for Myc-EZH2.

Since EZH2 has been shown to be regulated by the proteasome in various cancer cells (Wu & Zhang, 2011; Zoabi et al, 2011), we asked whether the decrease in EZH2 protein levels might be a result of proteasome degradation during neuron differentiation of hMSCs. In the presence of the proteasome inhibitor MG132, the levels of polyubiquitinated EZH2 accumulated after neuron differentiation in both 3A6-hMSCs and primary hMSCs (Fig 2A). Consistently, inhibition of proteasome degradation restored the EZH2 protein level (Fig 2A). These results suggest that EZH2 is posttranslationally regulated by the ubiquitin-proteasome-dependent degradation pathway during neuron differentiation. To identify which E3 ubiquitin ligases might be responsible for the EZH2 degradation, we screened a panel of E3 ubiquitin ligases for EZH2 degradation (Supporting Information Fig S2). Expression of the E3 ubiquitin ligase, Smurf2, had the most significant effect on EZH2 protein levels compared with the other E3 ubiquitin ligases (Mdm2, Fbw7, Cbl-b, Skp2 and Smurf1), and Smurf2-mediated decrease in EZH2 was restored by the addition of a proteasome inhibitor, MG132 (Supporting Information Fig S2), suggesting that Smurf2 is the primary E3 ligase for EZH2.

Smurf2 is required for the establishment of neuronal polarity (Schwamborn et al, 2007). We found that its mRNA (Fig 2B) and protein levels (Fig 2C) were increased during neuron differentiation. To test whether Smurf2 interacts with EZH2, we performed co-immunoprecipitation experiments by transiently transfecting HEK 293 cells with a Flag-tagged Smurf2 and Myc-tagged EZH2 and then treated the cells with MG132 to inhibit EZH2 degradation (Fig 2D). Figure 2D shows the co-immunoprecipitation experiment demonstrating the interaction between EZH2 and Smurf2, which is more evident in the presence of MG132. A similar result was also observed between endogenous Smurf2 and EZH2 proteins (Fig 2E). To determine whether the catalytic activity of Smurf2 is required for EZH2 degradation, we used a catalytically inactive Smurf2 mutant (Smurf2-C716A). Indeed, only the wild-type Smurf2 but not the C716A mutant was active in polyubiquitinating EZH2, which led to a decrease in EZH2 proteins (Fig 2F). Taken together, these data indicate that Smurf2 interacts with and polyubiquitinates EZH2 for degradation.

### Lysine 421 of EZH2 is critical for Smurf2-mediated EZH2 degradation

**Figure 3:**
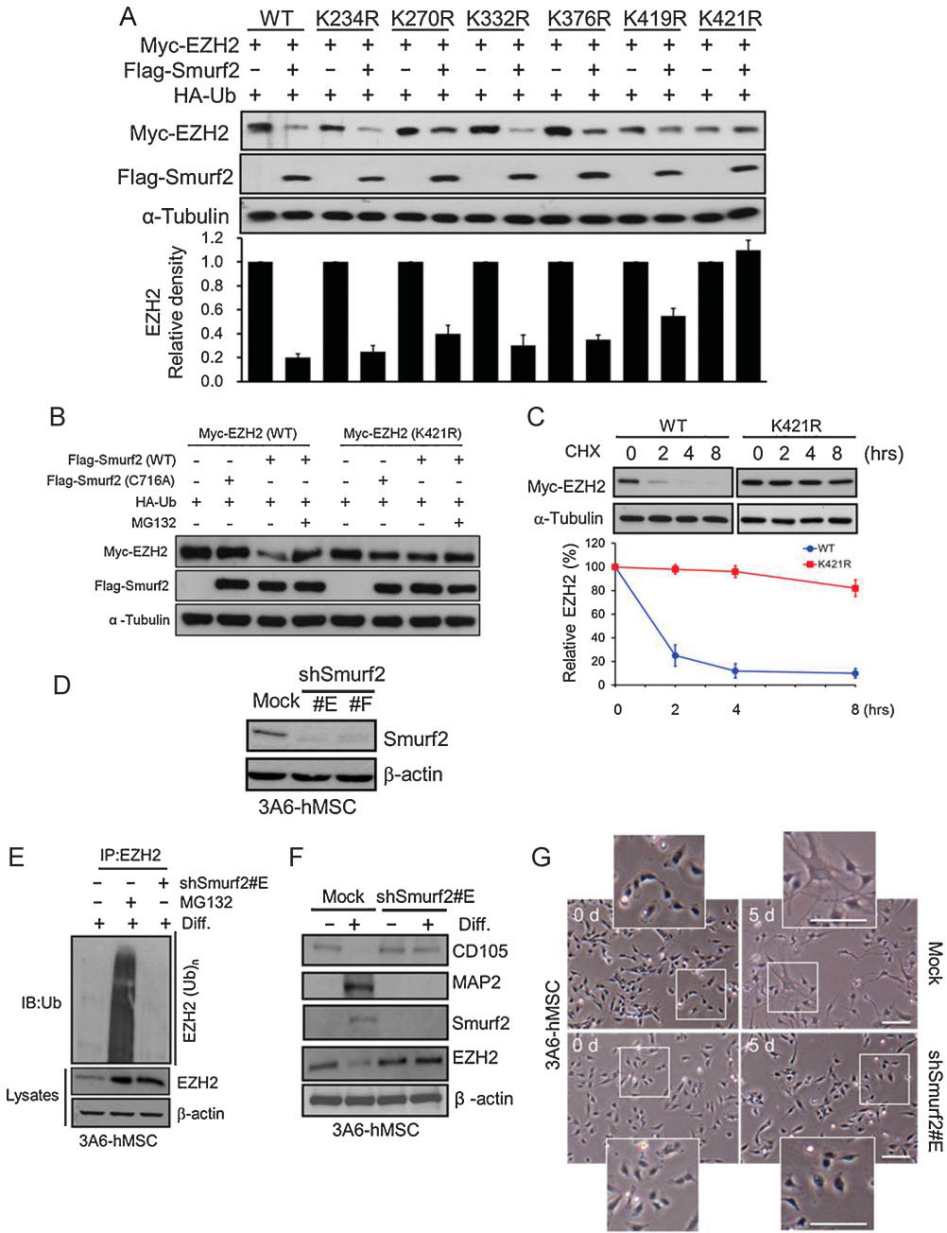
K421 of EZH2 is critical for Smurf2-mediated EZH2 degradation, which is required for neuron differentiation. Source data is available for this figure in the Supporting Information. A. HEK 293 cells were cotransfected wild-type or K to R mutants of Myc-EZH2 with HA-Ub with or without Flag-Smurf2 plasmids for 24 h. Total cell lysate was extracted, and expression of exogenous Myc-EZH2 and Flag-Smurf2 was examined (immunoblot, top). The plot (bottom) represents the relative density of EZH2 (wild-type and mutants) determined by scanning densitometric tracings. Error bars represent the SEM from three independent experiments (*n* = 3). B. Expression of ectopic Myc-EZH2 (wild-type or K421R) in the absence or presence of Flag-Smurf2 (wild-type or C716A) with or without MG132 in HEK 293 cells. C. HEK 293 cells were transfected with Myc-EZH2 (wild-type or K421R) plasmids. Each transfectant was treated with 50 µM cycloheximide for the indicated times. The levels of indicated proteins were determined by immunoblotting (top). The percentage of EZH2 degradation was calculated by the relative level of Myc-EZH2 (WT: red and K421R: blue) normalized to α-tubulin (bottom). Error bars represent the SEM from three independent experiments (*n* = 3). D. Immunoblotting of Smurf2 protein levels of 3A6-hMSCs with Smurf2 knockdown (shSmurf2 #E and #F). E. 3A6-hMSCs stably expressing Smurf2 shRNA (shSmurf2 #E) or pretreated with MG132 were treated with NIM for 5 days. EZH2 was then immunoprecipitated by an EZH2 antibody and blotted with an anti-ubiquitin antibody. F. The total cell lysates with or without induction of neuron differentiation in 3A6-hMSCs for 5 days and cells stably expressing Smurf2 shRNA (shSmurf2 #E) at the neuron stage were immunoblotted with the indicated antibodies. Changes in the MSC (CD105) and the neuron (MAP2) markers were analysed by immunoblotting. G. Cell morphology of NIM-treated 3A6-hMSCs with or without stably expressing shSmurf2 (shSmurf2 #E) at indicated times during neuron differentiation was observed under an inverted phase microscope. The inset is an enlarged image of the boxed (white) region. Scale bars: 50 µm.

To identify which lysine residue of EZH2 is critical for Smurf2-mediated degradation, we predicted potential ubiquitination sites by the UbPred program (http://www.ubpred.org) (Radivojac et al, 2010). Several lysine residues at positions 234, 270, 332, 376, 419 and 421 of EZH2 were predicted for ubiquitination with high confidence in five of six positions (except K270) that had also been predicted by another programme (UbiPred; http://iclab.life.nctu.edu.tw/ubipred/; Tung & Ho, 2008). We mutated each predicted Lys to Arg and determined the effect of each mutant on EZH2 stability in the absence or presence of Smurf2. As shown in Fig 3A, after the addition of Smurf2, the protein levels of wild-type EZH2 and of all of the mutants (K234R, K270R, K332R, K376R and K419R) except for the K421R mutant were decreased. In addition, the Smurf2-C716A mutant did not alter levels of EZH2, and treatment of MG132 restored the wild-type Smurf2-mediated decrease of EZH2 but not the decrease of the EZH2-K421R mutant (Fig 3B). We further examined the protein stability of wild-type and K421R mutant EZH2 by treating cells with 100 µM cycloheximide. The half-life of wild-type EZH2 was about 1.5 h, but that of the K421R mutant did not change significantly, even at 8 h (Fig 3C), indicating that the K421R mutant is more stable than wild-type EZH2 in the presence of Smurf2. Taken together, these results suggest that K421 of EZH2 is critical for Smurf2-mediated EZH2 degradation.

### Neuron differentiation requires Smurf2-mediated EZH2 degradation

To further determine the functional impact of Smurf2-mediated EZH2 degradation during neuron differentiation, we generated 3A6-hMSC cells stably expressing shSmurf2 (hMSCs/shSmurf2). As expected, the Smurf2 protein levels were dramatically reduced in 3A6-hMSCs when we knocked down Smurf2 (Fig 3D). In hMSCs/shSmurf2 cells, EZH2 protein expression was recovered, and polyubiquitination of EZH2 was virtually undetectable after neuron differentiation (Fig 3E). Knocking down Smurf2 also prevented hMSCs from undergoing neuron differentiation, as was evident from the unchanged CD105 expression and the absence of MAP2 (Fig 3F). In addition, cell body morphologies with dendritic arborization were also reduced compared with the parental control cell under neuron differentiation culture condition (Fig 3G). Collectively, these results suggest that Smurf2-mediated EZH2 degradation is a key step in neuron differentiation of hMSCs.

### Transcriptional repression of PPARγ by EZH2 in hMSCs

**Figure 4:**
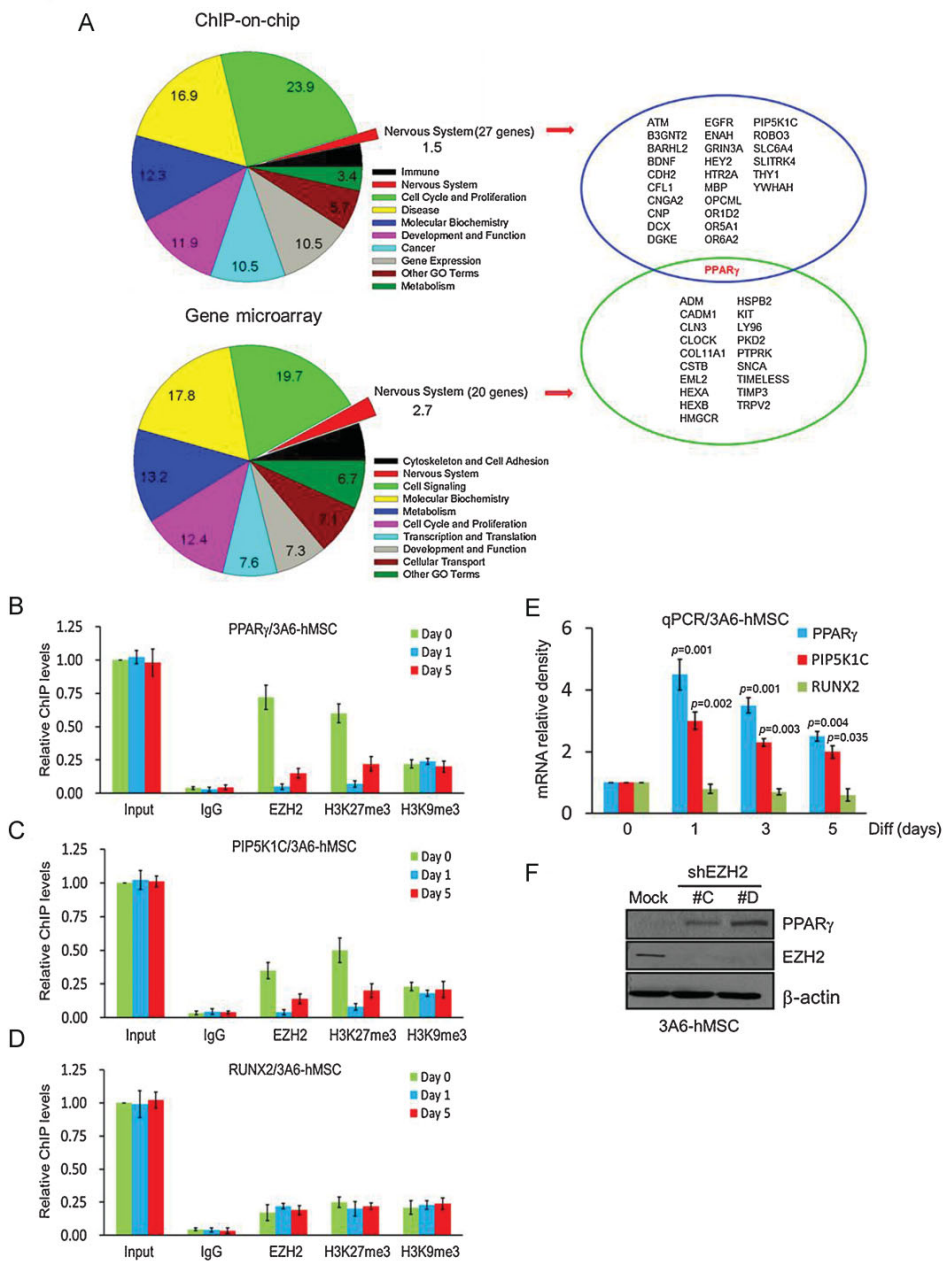
Expression of EZH2 target gene PPARγ during neuron differentiation. A. GO analysis of EZH2 target genes in ChIP-on-chip and gene microarray. Seventy-eight GO terms were found from the 388 overlapping genes by ChIP-on-chip analysis (peak score > 0.2; FDR < 0.05) and 218 GO terms were found from the 370 overlapping genes by gene microarray analysis (log 2(ratio) >1; *p*-value < 0.05). These category or GO terms were classified into 10 groups and the percentage is shown. B–D. qChIP was carried out with use of antibodies against EZH2, H3K27me3 and H3K9me3 on three EZH2 target gene promoters, PPARγ (B), PIP5K1C (C) and RUNX2 (D). Fold enrichment of precipitated DNA relative to 1:50 dilution of input chromatin. Error bars represent the SEM from three independent experiments (*n* = 3). E. qPCR analysis of EZH2 targeted genes, PPARγ, PIP5K1C and RUNX2 expression during neuron differentiation of 3A6-hMSCs. Error bars represent the SEM from three independent experiments. Significant *p* values are indicated (*n* = 3, Student's *t*-test). F. Restoration of PPARγ protein level after knockdown of EZH2 by shRNA (shEZH2 #C and #D) was performed by Western blot analysis.

Next, to understand how EZH2 regulates neuron differentiation of hMSCs, we wanted to identify the target genes of EZH2. To this end, we performed ChIP-on-chip (chromatin immunoprecipitation on chip) experiments by using specific EZH2 antibodies followed by a human promoter array for the whole human genome. Our initial screening revealed that among 28,869 promoters in the human genome (HG18; NCBI Build 36), 4506 of 6249 (15.6%; 4082 undifferentiated + 424 overlapped) were bound by EZH2 in undifferentiated hMSCs, and 1,743 of 6,249 (6.0%; 1,319 neuron-differentiated + 424 overlapped) were bound by EZH2 in neuron-differentiated hMSCs (a peak score >0.2 was considered as high-confidence binding sites for EZH2; Supporting Information Fig S3 and Table S1). We further analysed these 4082 genes that lose EZH2 binding upon differentiation by Ingenuity System Analysis. The 78 gene ontology (GO) terms (Supporting Information Table S2) identified from a total 3110 genes, including 388 overlapping genes (peak score >0.2 and FDR <0.05; Supporting Information Table S3), were classified into 10 groups including 27 overlapping genes in nervous system (1.5%; Fig 4A and Supporting Information Table S2).

In addition, the gene microarray analysis (Human Whole Genome OneArray) from 29,178 human genome probes showed that the expression of 815 genes from hMSCs/shEZH2 were upregulated ≥2-fold compared with the shMSCs/mock control (listed as heatmap in Supporting Information Table S4). GO analysis was performed by using the Biological Process category. The 218 GO terms identified from the 1423 genes including 370 overlapping genes (log 2(ratio) >1 and *p*-value <0.05; Supporting Information Tables S5 and S6) were classified into 10 groups including 20 overlapping genes in nervous system (2.7%; Fig 4A and Supporting Information Table S5). Interestingly, between these analyses, PPARγ was the only one identified as a potential EZH2 target gene involved in neuron differentiation (Fig 4A).

To determine if the 4082 genes that lose EZH2 binding retain trimethylation of histone H3 at lysine 27 (H3K27me3), we further validated the enrichment profiles of selected EZH2 target genes that were bound by EZH2 in undifferentiated hMSCs (PPARγ, PIP5K1C) or overlapped in both undifferentiated and neuron-differentiated hMSCs (RUNX2, as control) after neuron differentiation (Supporting Information Table S1) and quantitated EZH2 and two molecular marks, H3K27me3 and H3K9me3, that are associated with transcriptional silencing enrichments (Fig 4B–D). The results from quantitative ChIP (qChIP) analysis were similar to those from the ChIP-on-chip analysis (Supporting Information Table S1) showing that loss of EZH2 binding and H3K27me3 occurred at the same time at both PPARγ (Fig 4B) and PIP5K1C (Fig 4C) promoters during neuron differentiation of hMSCs. Our data indicated that EZH2 was released with a concomitant decrease in H3K27me3 at the PPARγ promoter on Day 1, and by Day 5, both EZH2 and H3K27me3 were reestablished, likely to maintain functional PPARγ level after neuron differentiation (Figs 4B and S4). The H3K9me3 status at these two EZH2 target genes, however, did not change during neuron differentiation, and thus it may not take over the role of EZH2 if H3K27me3 cannot be maintained. We did not observe loss of binding of either EZH2 or two histone trimethylation marks (H3K27me3 and H3K9me3) at the RUNX2 promoter during neuron differentiation (Fig 4D) which is not surprising as we previously demonstrated that this EZH2 target gene is important for osteogenic hMSC differentiation (Wei et al, 2011). We also analysed the mRNA expression levels of these EZH2 target genes and found that they were significantly increased for PPARγ and PIP5K1C but not for the osteogenic-specific RUNX2 during neuron differentiation, which peaked on Day 1 and then gradually decreased to a level higher than undifferentiated hMSCs on Day 5, presumably to sustain their expression (Fig 4E). Moreover, knocking down EZH2 also increased the protein expression of PPARγ (Fig 4F). Collectively, these results demonstrate that PPARγ is one of the major target genes of EZH2 during neuron differentiation of hMSCs and that its expression is suppressed at the MSC stage.

### Knockdown of PPARγ disturbs hMSC neuron differentiation

**Figure 5:**
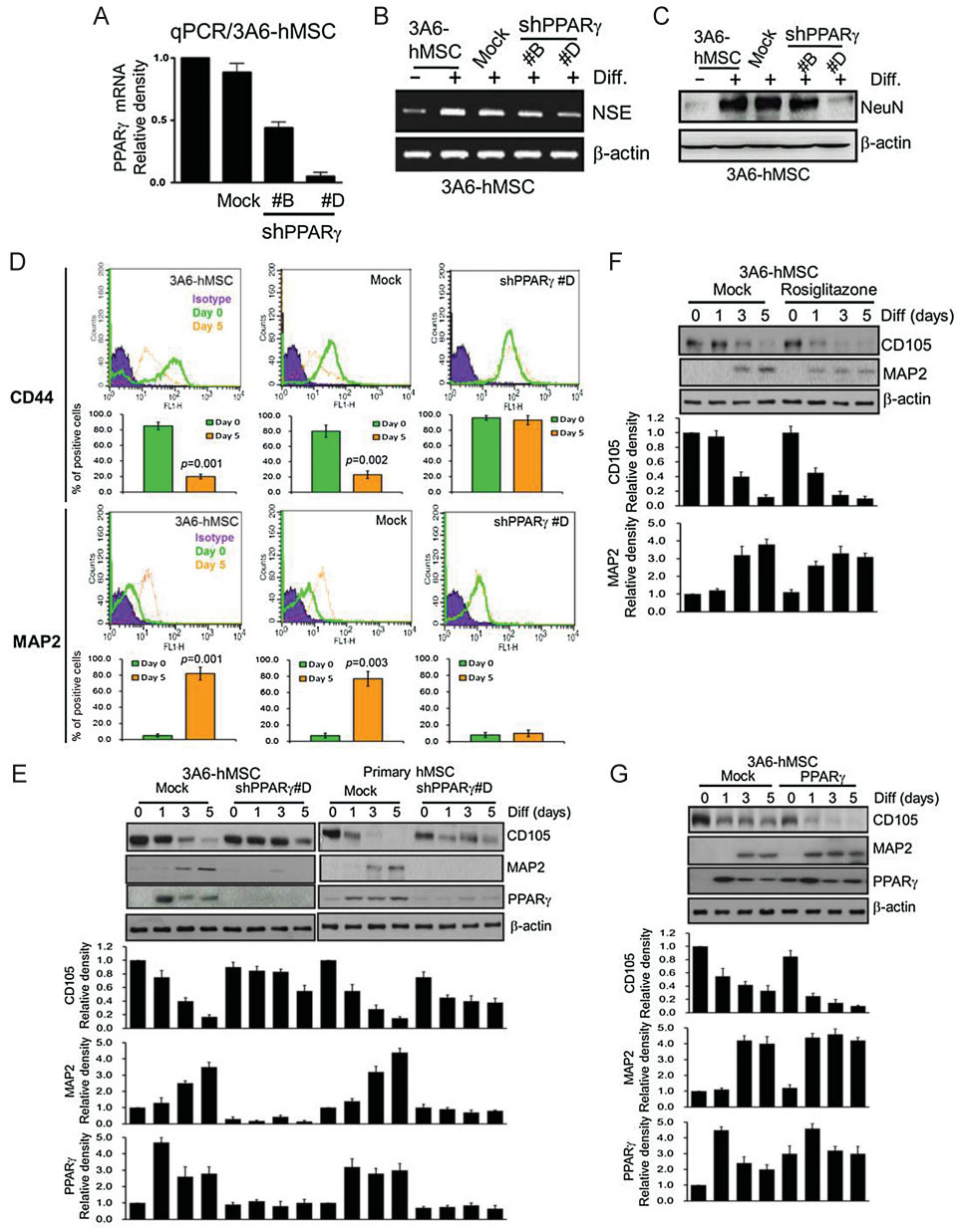
PPARγ accelerates neuron differentiation of hMSCs. Source data is available for this figure in the Supporting Information. A. qPCR analysis of lentiviral-mediated shRNA interference targeting PPARγ was used to allow for the generation of hMSC cells stably expressing shPPARγ (shPPARγ #B and #D). shLuc against luciferase was used as a negative control (mock). Error bars represent the SEM from three independent experiments (*n* = 3). B. RT-PCR analysis of NSE (neuron marker) mRNA levels with or without PPARγ shRNAs (shPPARγ #B and #D) in 3A6-hMSCs after neuron differentiation. β-actin was used as an internal control. shLuc against luciferase was used as a negative control (mock). C. Immunoblotting of NeuN (neuron marker) protein expression with or without PPARγ shRNAs (shPPARγ #B and #D) in 3A6-hMSCs after neuron differentiation. D. Flow cytometric analysis of MSC marker (CD44) and neuron marker (MAP2) in 3A6-hMSCs with or without PPARγ knockdown at Day 0 and Day 5 after differentiation. The plots show the percentage of cells in undifferentiated (Day 0) and neuronal differentiated (Day 5) hMSCs (bottom). shLuc against luciferase was used as a negative control (mock). Error bars represent the SEM from three independent experiments. Significant *p* values are indicated (*n* = 3, Student's *t*-test). E. The effect of PPARγ knockdown during neuron differentiation of 3A6-hMSCs and human primary MSCs (immunoblot, top). The plots (bottom) represent the relative density of MSC marker (CD105), neuron marker (MAP2) and PPARγ determined by scanning densitometric tracings. Error bars represent the SEM from three independent experiments (*n* = 3). F. The effect of the PPARγ agonist rosiglitazone during neuron differentiation of 3A6-hMSCs (immunoblot, top). The plots (bottom) represent the relative density of MSC marker (CD105) and neuron marker (MAP2) determined by scanning densitometric tracings. Error bars represent the SEM from three independent experiments (*n* = 3). G. Immunoblot analysis of the MSC (CD105) and neuron (MAP2) markers in 3A6-hMSCs during neuron differentiation with ectopically expressed PPARγ (top). The plots (bottom) represent the relative density of MSC marker (CD105), neuron marker (MAP2) and PPARγ determined by scanning densitometric tracings. β-actin was used as an internal control. Error bars represent the SEM from three independent experiments (*n* = 3).

It is interesting to note that PPARγ deficiency has been associated with ischaemic brain damage (Zhao et al, 2009). To further demonstrate the role of PPARγ in neuron differentiation, we generated 3A6-hMSCs stably expressing shPPARγ (hMSCs/shPPARγ). As expected, qPCR analysis showed that shPPARγ efficiently reduced the mRNA level of PPARγ (Fig 5A). Downregulation of PPARγ in hMSCs inhibited the expression of neuron differentiation-induced neuron markers NSE and NeuN (Fig 5B and C). Furthermore, when these hMSCs/shPPARγ cells were cultured with NIM, there was neither a reduction in the CD44 MSC marker nor an increase in the expression MAP2 neuron marker as determined by flow cytometry (Fig 5D). Consistent with this, knocking down PPARγ reduced the expression of the MAP2 neuron marker and further disturbed neuron differentiation from hMSC in either the 3A6-hMSCs or primary hMSCs (Fig 5E). In contrast, treatment with the PPARγ agonist rosiglitazone or overexpression of PPARγ induced early expression of MAP2 and accelerated the neuron differentiation compared with the mock control (Fig 5F and G). Collectively, these data suggest that upregulation of PPARγ is required for neuron differentiation.

### Transplantation of hMSCs/shEZH2 in rats improves neurological dysfunction after cerebral ischaemia

**Figure 6:**
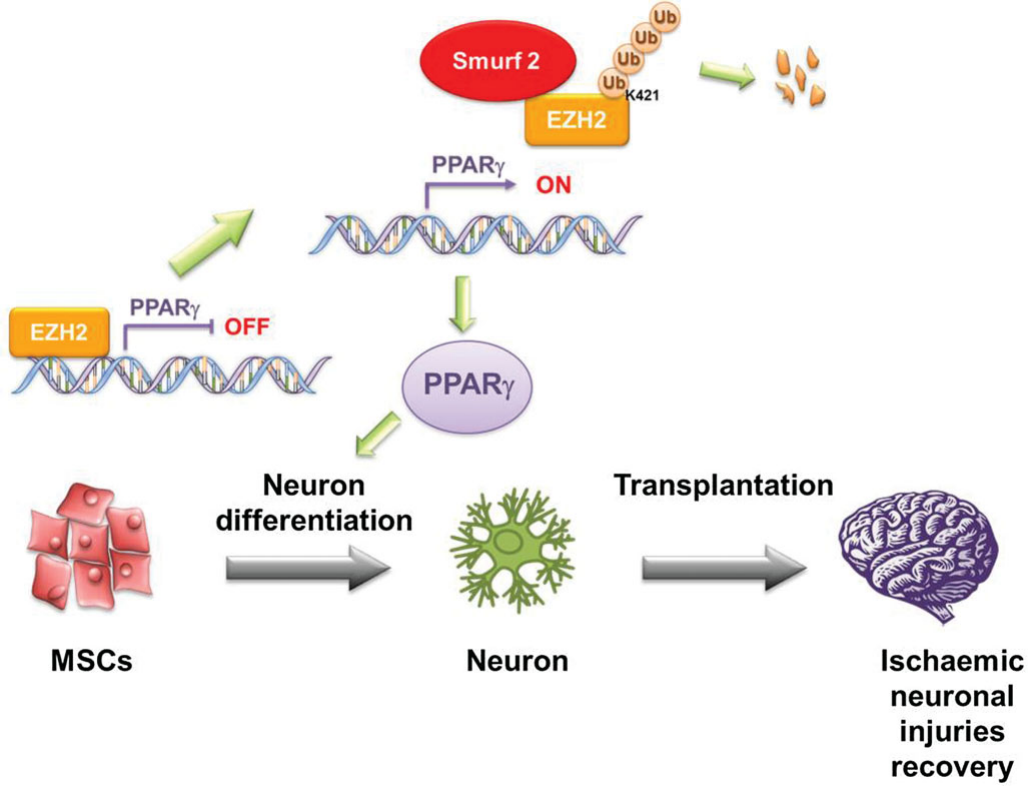
Smurf2-mediated degradation of EZH2 induces neuron differentiation of hMSCs. In proliferating hMSCs, EZH2 binds to the PPARγ promoter to repress neuron differentiation. After induction to neuron differentiation, EZH2 dissociates from the PPARγ promoter and is downregulated through Smurf2-mediated degradation to enhance gene expression of PPARγ to promote hMSC neuron differentiation.

hMSCs have been reported to improve neurological dysfunctions in animals, likely through their ability to differentiate into neurons (Chen et al, 2001; Li et al, 2002). Thus, transplantation of hMSCs may have clinical potential for the treatment of neurological dysfunctions. The current study demonstrates that upregulation of PPARγ facilitates neuron differentiation via Smurf2-mediated EZH2 degradation, as shown in the proposed model (Fig 6). Presumably, hMSCs can be engineered to accelerate neuron differentiation by modification of this pathway in hMSCs to increase therapeutic efficacy.

**Figure 7:**
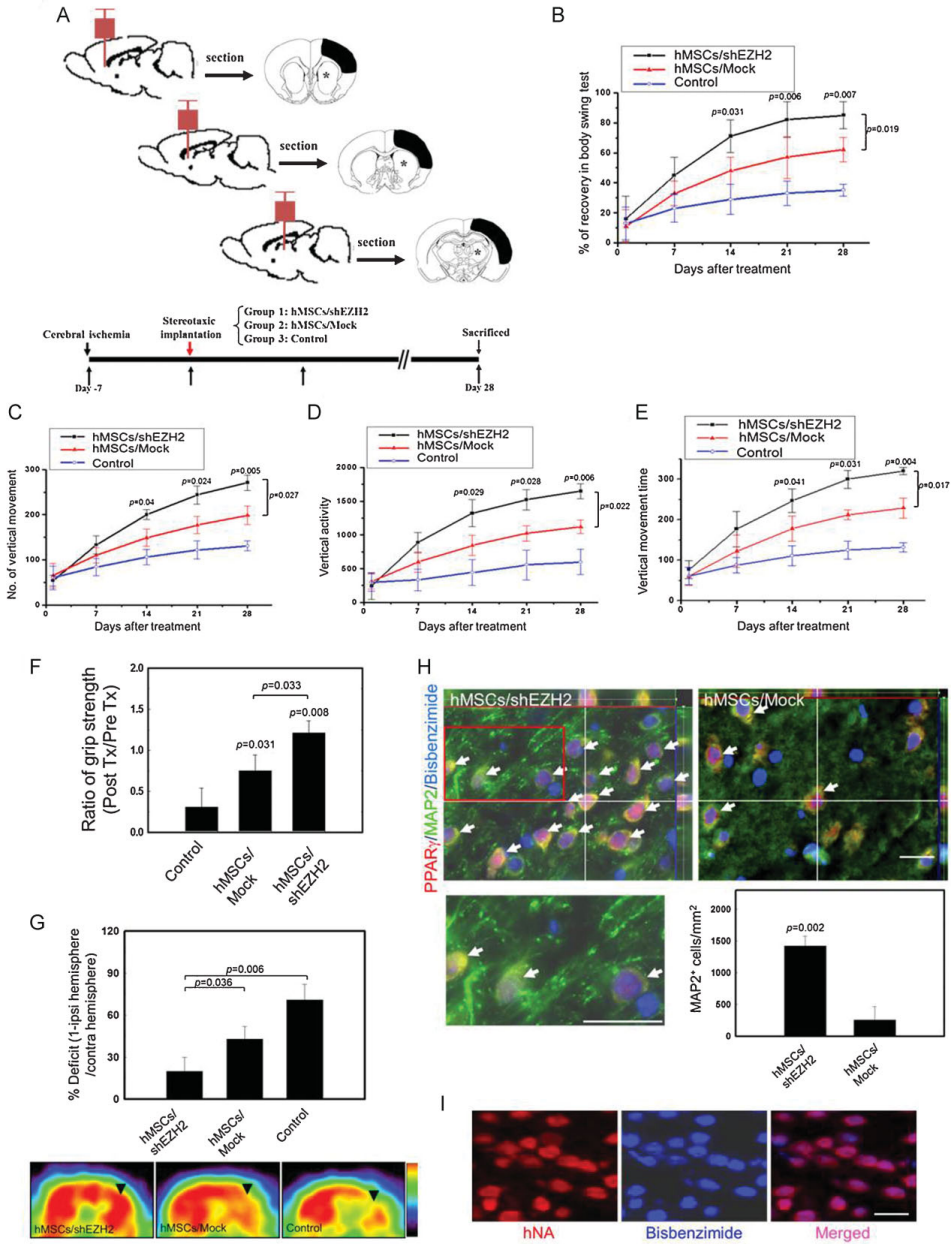
Intracerebral hMSCs/shEZH2 transplantation improves functional recovery after cerebral ischaemia. A. Schematic representation for the investigation of chronic cerebral ischaemia with three therapeutic protocols (Group 1 to 3, bottom). Three cell implantation sites are located from anterior to caudal portion in the sagittal view of rat brain (top) and comparative injection sites (starred) as well as infarction areas (black) are in the coronal view of rat brain. B. A body asymmetry trial was used to assess body swing before and after middle cerebral artery ligation. Data are expressed as mean ± SEM. Significant *p* values are indicated (*n* = 10, two-way ANOVA). C–E. Locomotor activities (vertical activity, vertical movement time, and the number of vertical movements) of all experimental rats were examined, Data are expressed as mean ± SEM. Significant *p* values are indicated (*n* = 10, two-way ANOVA). F. Grip strength measurement of the grasping power of forelimbs before (pre Tx) and 28 days after each of the three treatments (post Tx). Data are expressed as mean ± SEM. Significant *p* values are indicated (*n* = 10, two-way ANOVA). G. Semiquantitative measurement showing the relative glucose metabolic activity in the right cortex (top). Data are expressed as mean ± SEM. Significant *p* values are indicated (*n* = 6, two-way ANOVA). Representative images (coronal view) of ^18^FDG-PET of the right cortex (black arrowhead) of hMSCs/shEZH2-treated, mock hMSCs-treated (hMSCs/mock), and vehicle-treated control groups (Control). ipsi, ipsilateral; contra, contralateral (bottom). H. Representative three-dimensional image of bisbenzimide-labelled hMSCs (blue fluorescence) implantation in rat brain. The white arrows indicate the implanted bisbenzimide-labelled, PPARγ (red fluorescence) and MAP2-positive hMSCs with dendritic arborization (green fluorescence) in the ischaemic brains (top). The inset is an enlarged image of the boxed (red) region (bottom left). Scale bars: 50 µm. Quantitative analysis of the implanted MAP2-positive cells numbers in both the hMSCs/shEZH2-treated rats and mock hMSCs-treated rats (bottom right). Data are expressed as mean ± SEM. Significant *p* values are indicated (*n* = 6, two-way ANOVA). I. Colocalization of hNA (red fluorescence) with the implanted bisbenzimide-labelled hMSCs (blue fluorescence) indicated that cells were of human origin. Scale bars: 50 µm.

To determine whether engineered hMSCs can improve neurological functions after cerebral ischaemia, we evaluated the effect of implanting mock hMSCs without EZH2 knockdown (hMSCs/mock) in rats after cerebral ischaemia (Chang et al, 2003; Dunnett et al, 1998; Shyu et al, 2008) and of implanting hMSCs with EZH2 knockdown (hMSCs/shEZH2) and vehicle-treated controls (control) as illustrated in Fig 7A. We measured results from body asymmetry trials, locomotor activity tests and grip strength tests before and after middle cerebral artery (MCA) ligation in hMSCs/shEZH2- and hMSCs/mock-treated rats (Chen et al, 1986). The behavioural measurement scores were all normalized to the baseline scores. Since unilateral cerebral ischaemia causes imbalanced motor activity, all of the experimental rats developed significant body asymmetry, turning contralateral to the side of the induced ischaemia on Day 1 after cerebral ischaemia. Between 14 and 28 days after each treatment, rats with hMSCs/shEZH2 intracerebral transplants exhibited enhanced body symmetry, as indicated by the increase in the percentage of recovery in the body swing test compared with that of mock hMSCs-treated and untreated (control) rats (Fig 7B). Locomotor activity was also examined before and after cerebral ischaemia in all animals. Vertical activity, vertical movement time, and the number of vertical movements significantly increased between 14 and 28 days after cerebral ischaemia in rats that received hMSCs/shEZH2 transplantation compared with these movements in rats that received mock hMSCs treatment or no treatment (control) rats (Fig 7C–E). Furthermore, forelimb grip strength for all experimental rats was measured before and 28 days after treatment in each of the groups. The hMSCs/shEZH2-treated rats demonstrated higher postischaemic grip strength than did those treated with mock hMSCs or those that were untreated (Fig 7F). Since glucose metabolism is strongly correlated with functional plasticity of the brain, to determine whether hMSCs/shEZH2 implantation could enhance glucose metabolic activity (Hamacher et al, 1986; Matsumura et al, 2003), experimental rats were examined by positron emission tomography with [^18^F]-fluoro-2-deoxyglucose (FDG-PET) with use of a microPET scanner (Brownell et al, 1998; Carmichael et al, 2004; Kornblum et al, 2000). At 4 weeks after each treatment, FDG uptake was significantly greater in the right cortical region (ischaemic area of model) of the hMSCs/shEZH2-treated group (Fig 7F). Semiquantitative measurement of the relative glucose metabolic activity in the right cortical region revealed higher activities in the hMSCs/shEZH2-treated rats than in the mock hMSCs-treated or control rats (Fig 7G).

To further investigate the effect of EZH2 knockdown and increased PPARγ expression in neuron differentiation *in vivo*, we implanted hMSCs with or without knockdown of EZH2 to the ischaemic rat brains for 3 weeks and examined the expression of MAP2 in their tissue sections. Three-dimensional images from the colocalization study showed exogenous implanted bisbenzimide-labelled hMSCs (blue fluorescence), PPARγ (red fluorescence) and MAP2 (green fluorescence) positive cells (PPARγ^+^/MAP2^+^/bisbenzimide^+^) in implanted hMSCs as indicated by the arrows (Fig 7H, top). Co-expression of human nuclear antigen (hNA) in the exogenous implanted bisbenzimide-labelled hMSCs further confirmed that cells were of human origin (Fig 7I). A significant increase in the numbers of PPARγ^+^/MAP2^+^/bisbenzimide^+^ cells with dendritic arborization was found in the hMSCs/shEZH2-treated rats compared to the hMSCs/mock-treated rats (Fig 7H, top; inset, bottom left). Quantitative analysis showed that the implanted MAP2-positive cells is significantly higher in EZH2-silenced hMSCs than in the mock-treated hMSCs *in vivo* (Fig 7H, bottom right). Taken together, these data indicate that transplantation of hMSCs/shEZH2 in rats induced functional recovery better than did transplantation of parental hMSCs after ischaemic neuronal injuries.

### PPARγ agonist (rosiglitazone) can augment recovery of injuries by hMSC injection after cerebral ischaemia

**Figure 8:**
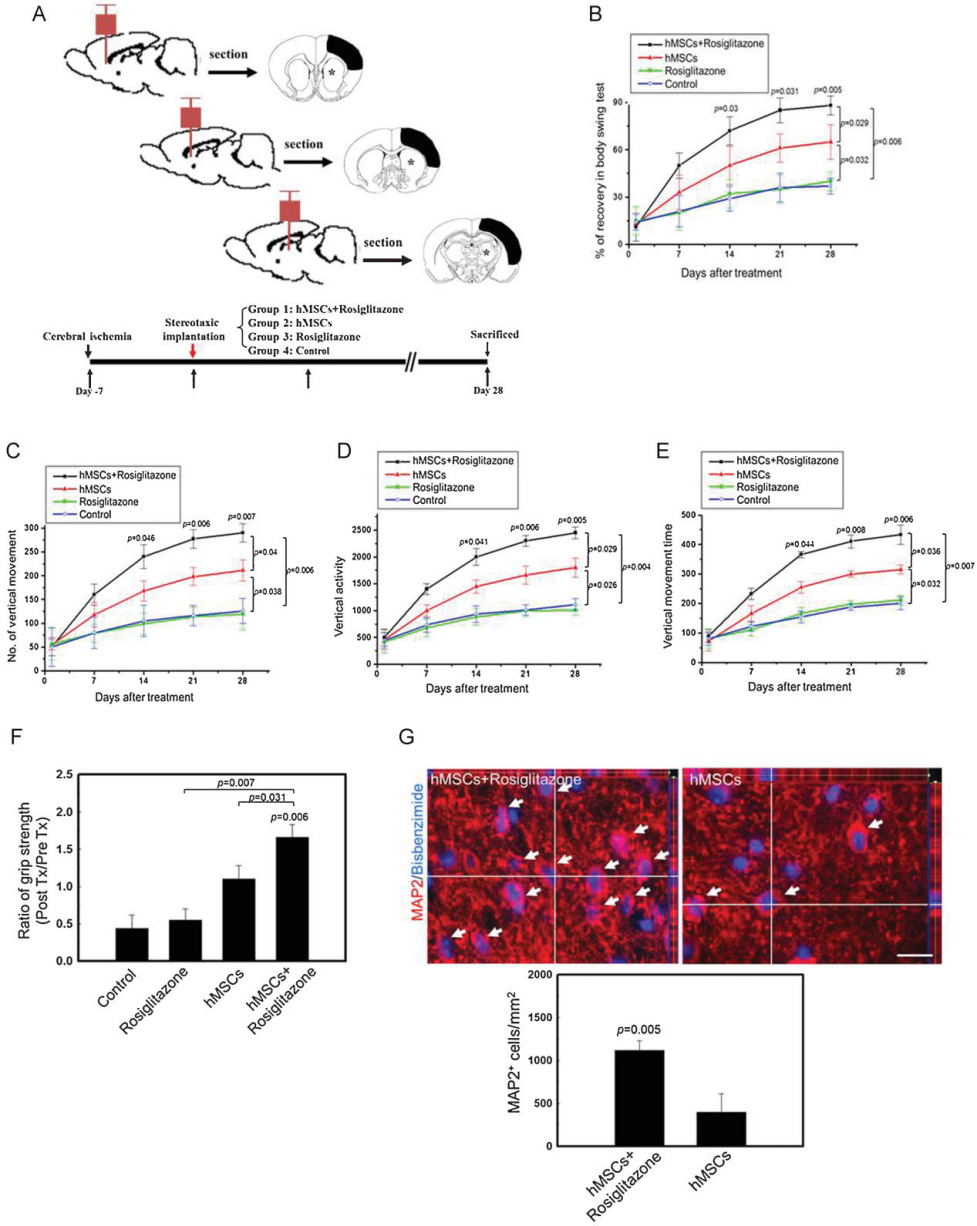
PPARγ agonist (rosiglitazone) can augment recovery of injuries by hMSC injection after cerebral ischaemia. A. Schematic representation for the investigation of chronic cerebral ischaemia with four therapeutic protocols (Group 1–4, bottom). Three cell implantation sites are located from anterior to caudal portion in the sagittal view of rat brain (top), and comparative injection sites (starred) as well as infarction areas (black) are in the coronal view of rat brain. B. A body asymmetry trial was used to assess body swing before and after middle cerebral artery ligation. Data are expressed as mean ± SEM. Significant *p* values are indicated (*n* = 10, two-way ANOVA). C–E. Locomotor activities (vertical activity, vertical movement time, and the number of vertical movements) of all experimental rats were examined, Data are expressed as mean ± SEM. Significant *p* values are indicated (*n* = 10, Two-way ANOVA). F. Grip strength measurement of the grasping power of forelimbs before (pre Tx) and 28 days after each of the three treatments (post Tx). Data are expressed as mean ± SEM. Significant *p* values are indicated (*n* = 10, two-way ANOVA). G. Representative three-dimensional images of bisbenzimide-labelled hMSCs (blue fluorescence) implantation in rat brain. The white arrows indicate the implanted MAP2 (red fluorescence)-positive hMSCs in the ischaemic brains (top). Scale bars: 50 µm. Quantitative analysis of the implanted MAP2-positive cells numbers in both the hMSCs + rosiglitazone-treated rats and mock hMSCs-treated rats (bottom). Data are expressed as mean ± SEM. Significant *p* values are indicated (*n* = 6, two-way ANOVA).

The use of PPARγ agonist might be a potential strategy to augment recovery of injuries by hMSC injection after cerebral ischaemia as PPARγ agonist (rosiglitazone) induced early expression of MAP2 and accelerated the neuron differentiation *in vitro* (Fig 5F). Therefore, we investigated whether the rosiglitazone administration exerts a similar effect on neurological improvement as illustrated in Fig 8A. Surprisingly, we observed significant improvement in neurological behaviours (body asymmetry, locomotor activity, and grip strengths) in the hMSCs + rosiglitazone-treated group compared the hMSCs-treated control and rosiglitazone-alone groups (Fig 8B–F). In addition, the number of exogenous implanted MAP2^+^/bisbenzimide^+^ neurons with dendritic arborization (red fluorescence) was significantly increased in rats that received hMSCs implantation plus rosiglitazone than rats that did not receive rosiglitazone (Fig 8G). These results indicate that rosiglitazone can augment recovery of injuries by wild-type hMSC implantation to enhance neuron differentiation.

## DISCUSSION

MSCs exhibit a broad degree of plasticity similar to that of other adult stem cell populations, have an intrinsic neurogenic potential, and can differentiate into neural cells both *in vitro* and *in vivo* (Brazelton et al, 2000; Kabos et al, 2002; Nakano et al, 2001; Sanchez-Ramos et al, 2000). Moreover, a dramatic decrease in the binding of the H3K27me3 and PcG proteins at neuron-specific genes occurs during ESC differentiation into neural precursor cells and during neuron differentiation (Boyer et al, 2006; Bracken et al, 2006; Mikkelsen et al, 2007). Importantly, knockdown of the *EZH2* gene in ESCs markedly enhances neuron differentiation during neocortical development (Hirabayashi et al, 2009). However, it remains unclear how PcG proteins are preferentially removed from transcribed regions of MSCs during neuron differentiation via posttranscriptional or posttranslational regulation.

Recently, Wu et al. reported that CDK1-mediated phosphorylation of EZH2 regulates its stability and provide a mechanism by which EZH2 protein levels can be regulated in cells (Wu & Zhang, 2011). In addition, Zoabi et al. demonstrated that a ubiquitin ligase, PRAJA1, can target individual PRC2 members for ubiquitination and subsequent proteasomal degradation in a cell-free system (Zoabi et al, 2011). Nonetheless, the regulation of EZH2 stability is still unclear. Here, we report a model showing that Smurf2, an ubiquitin E3 ligase, regulates EZH2 through a proteasome-mediated degradation mechanism during neuron differentiation of hMSCs. Smurf2 is related to ubiquitin E3 ligases of the C2-WW-HECT family that play an important role in signalling regulation and planar cell polarity and motility (Izzi & Attisano, 2006; Narimatsu et al, 2009). It has been suggested that Smurf2 targets Smad1, Smad2, Smad7 and the TGF-β receptor for degradation and is involved in the establishment of neuronal polarity via ubiquitination of the GTPase Rap1B (Kavsak et al, 2000; Lin et al, 2000; Schwamborn et al, 2007; Zhang et al, 2001). In particular, mice with the Smurf2 mutant display defects that include failure to close the neural tube (Narimatsu et al, 2009). Therefore, our results linking ubiquitin E3 ligase and PcG proteins during neuron differentiation support the model in which EZH2 protein downregulation occurs during neuron differentiation via posttranslational regulation (Figs 1C and 2A).

In our previously study, we demonstrated that after induction to neuron differentiation, decreased EZH2 leads to hMSC differentiation into functional neuron lineage. We also provided evidence to establish that PIP5K1C is transcriptionally suppressed by EZH2 and silencing EZH2 enhanced neuron differentiation might be mediated via one of the pathways by which activation of PIP5K1C to evoke intracellular Ca^2+^ signalling (Yu et al, 2011). Therefore, EZH2 may suppress several key target genes that are involved and/or crosstalk with Ca^2+^ signalling in neuron differentiation from hMSCs. Furthermore, our genome-wide analysis of ChIP-on-chip and gene microarray identified one EZH2 target gene, PPARγ, which is bound by EZH2 in undifferentiated hMSCs and upregulated during neuron differentiation (Fig 4A and B) among several other target genes when EZH2 is knocked down in hMSCs (Supporting Information Tables S1 and S3). Indeed, it has been reported that PPARγ plays an important role in regulating neuron differentiation of embryonic midbrain cells, and a deficiency in PPARγ is associated with ischaemic brain damage (Zhao et al, 2009). However, the underlying molecular mechanisms remain to be elucidated. In addition, PPARγ agonists rosiglitazone and 15d-PGJ2 have been reported to effectively attenuate tissue damage caused by ischaemia reperfusion in animal models (Lin et al, 2006; Shimazu et al, 2005; Sundararajan et al, 2005) and may modulate Ca^2+^ signalling and homeostasis in neurons (Pancani et al, 2009). PPARγ-mediated 14-3-3ε upregulation also plays a pivotal role in neuroprotection and the beneficial effects of rosiglitazone against ischaemic stroke (Wu et al, 2009). Our data showed that knockdown of PPARγ inhibited cells from undergoing efficient neuron differentiation and arrested them at the MSC stage (Figs 5B–E) and that physiological concentrations of the PPARγ agonist rosiglitazone and ectopic expression of PPARγ accelerated neuron differentiation of hMSCs *in vitro* (Fig 5F and G). Therefore, PPARγ agonist treatment after hMSCs implantation may augment recovery of injuries by hMSC injection by enhancing neuron differentiation *in vivo* (Fig 8B–G).

Although functional recovery after a neurogenic response injury of the brain and spinal cord has been very limited and inefficient, MSCs have been shown to enhance neurogenesis and angiogenesis in stroke and ischaemic limb models; thus MSCs, obtained from bone marrow stroma and human umbilical cord matrixes, are attractive candidates for clinical use in stem cell therapy (Croft & Przyborski, 2009; Ding et al, 2007; Gu et al, 2010; Kim et al, 2006). In addition, several human clinical trials have demonstrated the efficacy of administering hMSCs for the treatment of various diseases (Horwitz et al, 2002; Koc et al, 2002). In addition, many reports have suggested that the injured brain might specifically attract stem cells or that stem cells might ‘home in’ on the injured brain for direct (neuron differentiation) or indirect (production of cytokines and/or growth factors) repair (Chen et al, 2001; Ding et al, 2007; Petersen et al, 1999), making stem cell transplantation one of the better strategies for treating various neurodegenerative disorders (Borlongan et al, 2004; Yasuhara et al, 2010). Strikingly, in a rat model of stroke, rats that underwent intracerebral hMSC implantation showed improvement in behavioural measures of neurological deficit after stroke (Chang et al, 2003; Dunnett et al, 1998). In the current study, we discovered a signal pathway that is critical for neuron differentiation by hMSCs (Fig 6). This novel signal pathway offers the opportunity to engineer hMSCs to accelerate neuron differentiation by either knocking down EZH2, overexpressing Smurf2, or activating PPARγ through its agonist rosiglitazone and/or protein overexpression. For proof of concept, indeed, knocking down EZH2 in hMSCs or treatment with PPARγ agonist after hMSCs implantation significantly improved behavioural measures of neurological deficit after stroke in the animal model, opening an avenue for using hMSC transplantation to treat neurodegenerative diseases.

## MATERIALS AND METHODS

### ChIP-on-chip for EZH2

The 3A6-hMSCs were differentiated into neuron for 5 days, and then 10^9^ cells were harvested for the ChIP-on-chip assay. The procedure was based on the manufacturer's instructions (NimbleGen, USA). Results from the NimbleGen human ChIP-chip 385K promoter array (two-array set; 28,869 promoters (HG18; NCBI Build 36); 385,000 probes on a single glass slide) were analysed by SignalMapTM software and classified by Ingenuity System Analysis. A detection *p*-value of 0.05 was used to set the threshold of the probesets as present (*p* ≤ 0.05) or absent (*p* > 0.05). Probesets were identified and a peak score >0.2 and FDR <0.05 was considered as high-confidence binding sites for EZH2 and normalized to the replicate mean and visualized with heatmaps to assist in interpretation. The raw data have been deposited in a Gene Expression Omnibus (GEO) database (http://www.ncbi.nlm.nih.gov/geo/; accession number GSE42686).

**The paper explained**

**PROBLEM**

Functional recovery after a neurogenic response injury of the brain and spinal cord is very limited and insufficient. hMSCs exhibit a wide range of flexibility similar to that of other adult stem cell populations, which have intrinsic neurogenic potential for differentiation into neural cells both *in vitro* and *in vivo*. Recently, transplantation of bone marrow-derived hMSCs was reported to improve recovery of the injured brain and spinal cord in animal models. However, the molecular mechanisms associated with hMSCs' ability to directly differentiate or indirectly improve regeneration of the central nervous system and repair injured brain and spinal cord remain elusive.

**RESULTS**

In this work, we identified a novel signal pathway that is critical for neuron differentiation, namely that Smurf2 represses EZH2, which normally represses expression of PPARγ, which in turn causes neuron differentiation. Knocking down EZH2 in hMSCs significantly improved behavioural measures of neurological deficit after stroke in an animal model.

**IMPACT**

Our study demonstrates that EZH2 plays an important role in improving ischaemic neuronal injuries and suggests a model of PPARγ-dependent neuron differentiation through Smurf2-mediated degradation of EZH2. This modification of hMSCs to accelerate neuron differentiation may have important clinical implication in the regeneration of neurodegenerative diseases.

### Gene microarray analysis

Total RNA were extracted from 3A6-hMSCs/mock and 3A6-hMSCs/shEZH2. The RNA samples analysed by the Human Whole Genome OneArrayH v5 (Phalanx Biotech Group, Taiwan) for gene microarray analysis. The array contains 30,275 DNA oligonucleotide probes, 29,178 human genome probes, and 1088 experimental control probes formed as 60-mer sense-strand DNA elements. The procedure was based on the manufacturer's instructions (Phalanx Biotech Group, Taiwan). A detection *p*-value of 0.05 was used to set the threshold of the probesets as present (*p* ≤ 0.05) or absent (*p* > 0.05). Probesets were identified as differentially expressed when the absolute fold change was >2, and the probeset selected for visualization were log 2-transformed and normalized to the replicate mean. These normalized data were analysed with hierarchical clustering (Pearson correlation, average linkage) and visualized with heatmaps to assist in interpretation. The raw data have been deposited in a GEO database (accession number GSE42687).

### *In vivo* brain ischaemia/reperfusion

Adult male Sprague-Dawley (SD) rats (weight, >300 g; age, 7–8 weeks) were used in this study. All animal experiments were approved by the Institutional Review Board of Animal Experiments at the China Medical University Hospital (100-187-N). The rats were anaesthetized with chloral hydrate [0.4 g/kg intraperitoneally (i.p.)] and subjected to right MCA ligation and bilateral common carotid artery (CCA) clamping as previously described (Chen et al, 1986). Briefly, bilateral CCAs were clamped with nontraumatic arterial clips. With use of a surgical microscope, a 2-mm × 2-mm craniotomy was drilled at the point where the zygoma fuses to the squamosal bone, and the right MCA was then ligated with a 10-0 nylon suture. Cortical blood flow was measured continuously with a laser Doppler flowmeter (PF-5010, Periflux system; Perimed AB, Ardmore, PA, USA) in anesthetized animals. A photodetector probe (0.45-mm diameter) was stereotaxically placed through a skull burr hole (1-mm diameter) in the frontoparietal cortex (1.3 mm posterior, 2.8 mm lateral to the bregma and 1.0 mm below the dura). After 90 min of ischaemia, the 10-0 suture on the MCA and arterial clips on CCAs were removed to allow for reperfusion. During recovery from anaesthesia, body temperature was maintained at 37°C with a heat lamp.

### Neuron differentiation from hMSCs in animal brains

Before implantation, hMSCs with or without knockdown of EZH2 were incubated with 1 µg/ml bisbenzimide Hoechst 33342 (Sigma–Aldrich) for 5 h at 37°C to label nuclei with blue fluorescence. After the labelled hMSCs were washed three times with phosphate-buffered saline, they were counted with use of a cytometer to ensure an adequate cell number for implantation. The SD rats were anaesthetized with chloral hydrate (0.4 g/kg, i.p.) and then were injected stereotaxically with approximately 1 × 10^6^ cells in 3–5 µl of DMEM medium through a 26-gauge Hamilton syringe into three cortical areas adjacent to the right MCA, 3.0–5.0 mm below the dura mater. Injections of cyclosporin A (CsA), an immunosuppressant drug (1 mg/kg/d i.p.; Novartis, USA), were given daily to each experimental rat for 3 weeks (Shyu et al, 2008). In rosiglitazone treatment after stroke, rats were subdivided into three groups: hMSCs implantation combined with intraperitoneal injection of rosiglitazone (3 mg/kg/day in 10% DMSO for 8 days, Millipore), hMSCs alone, and vehicle-control (Di Paola et al, 2006; Pereira et al, 2006).

### Neurological behavioural measurements

Behavioural assessments were performed 3 days before and 72 h after cerebral ischaemia. The tests measured body asymmetry and locomotor activity as previously described (Chang et al, 2003; Shyu et al, 2008). Furthermore, grip strength was analysed with use of the Grip Strength Meter (TSE-Systems, USA), as previously described with modification (Dunnett et al, 1998; Shyu et al, 2008). Briefly, the percentage of improvement in grip strength was measured on each forelimb separately and was calculated as the ratio between the mean strength of 20 pulls of the side contralateral to the ischaemia and the ipsilateral side. In addition, the ratio of grip strength after treatment to baseline was also calculated, and changes were presented as percent of baseline. The neurological behaviour of the rats was then measured as described above to compare with the control group.

### Laser scanning confocal microscopy for immunofluorescence colocalization analysis

To determine the neuron differentiation of hMSCs in rat brain, tissue sections from exogenous implanted bisbenzimide-labelled hMSCs (blue fluorescence) were stained with specific primary antibodies (1:300) against PPARγ and a neuron specific marker, MAP2. Subsequently, the tissue sections were stained with Cy5- and Cy3-conjugated secondary antibodies (1:500, Jackson Immunoresearch). The immunofluorescent colocalization study with three-dimensional images was performed to test for the expression of exogenous implanted hMSC (blue fluorescence), PPARγ (red fluorescence), and MAP2 (green or red fluorescence) with dendritic arborization. The three-dimensional images were observed under a Carl Zeiss LSM510 laserscanning confocal microscope. Co-expression of human nuclear antigen (hNA, 1:100, Chemicon) in the bisbenzimide-labelled cells further confirmed that cells implanted were of human origin.

### ^18^FDG-PET analysis

The experimental rats were examined by FDG-PET with use of a microPET scanner to measure relative glucose metabolic activity, as previously described (Matsumura et al, 2003). In brief, ^18^F was produced by the ^18^O(p,n)^18^F nuclear reaction in a cyclotron at the China Medical University and Hospital, and FDG was synthesized as previously described with an automated FDG synthesis system (Hamacher et al, 1986). Data were collected with a high-resolution small animal PET (microPET Rodent R4; Concorde Microsystems, Malvern, PA, USA). The system parameters were as previously described (Carmichael et al, 2004). After 4 weeks of each treatment, animals were anesthetized with chloral hydrate (0.4 g/kg, i.p.), and the head was fixed in a customized stereotactic head holder and positioned in the microPET scanner. The animals were then given an intravenous bolus injection of FDG (200–250 µCi/rat) dissolved in 0.5 ml of saline. Data acquisition began at the same time and continued for 60 min in one bed position with use of a 3D acquisition protocol. The image data acquired from microPET were displayed and analysed by Interactive Data Language (IDL) version 5.5 (Research Systems, Malvern, PA, USA) and ASIPro version 3.2 (Concorde Microsystems, Malvern, PA, USA). FDG-PET images were reconstructed by using a posterior-based 3D iterative algorithm (Kornblum et al, 2000) and overlaid on MR templates to confirm anatomical location (Brownell et al, 1998). Coronal sections for striatal and cortical measurements represented brain areas between 0 and +1 mm from the bregma, whereas thalamic measurements were between −2 and −3 mm from the bregma, as estimated by visual inspection of the contralateral side. The relative metabolic activity in regions of interest of the striatum and cortex was expressed as percent deficit, as previously described with modification (Carmichael et al, 2004).

### Statistical analysis

Statistical analysis was performed with Student's *t*-test or two-way ANOVA with appropriate post hoc Newman–Keuls testing between different groups. Quantitative data were presented as means ± SEM from at least three independent experiments. The criterion of significance was set as *p* < 0.05.

For more detailed Materials and Methods see the Supporting Information.

## Author contributions

The author(s) have made the following declarations about their contributions: YLY and MCH conceived and designed the experiments. YLY, RHC, WCS and ShuCH performed the experiments. CSW, SYC, WJC, JNC, YJT and YHL analysed the data. WL, SPY, CCY and ShiCH contributed reagents/materials/analysis tools. YLY, JLH, and MCH wrote the paper.

## Notes

As a service to our authors and readers, this journal provides supporting information supplied by the authors. Such materials are peer reviewed and may be re-organized for online delivery, but are not copy-edited or typeset. Technical support issues arising from supporting information (other than missing files) should be addressed to the authors.
