# Supplementary figures and images for: Smurf2-mediated degradation of EZH2 enhances neuron differentiation and improves functional recovery after ischaemic stroke
Source: EMBO Mol Med. 2013 Mar 25;5(4):531–47. doi: 10.1002/emmm.201201783 (PMC3628108; doi:10.1002/emmm.201201783)

Fig 1C

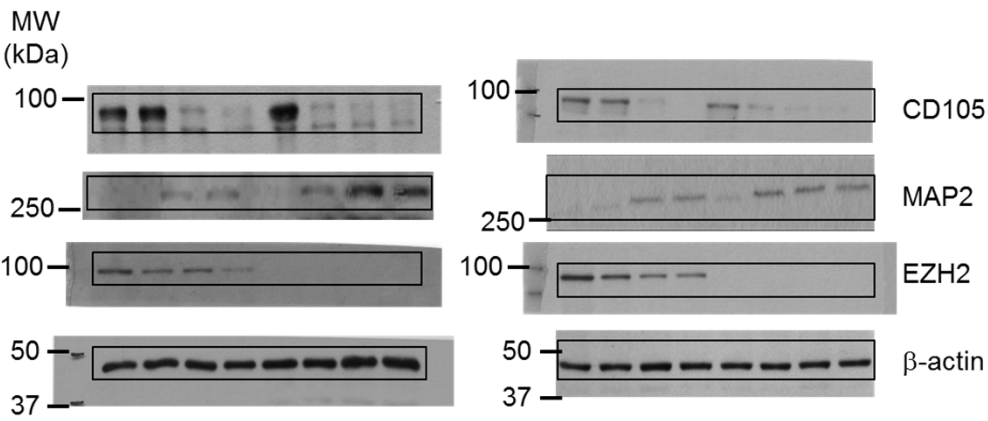

Supplement: Supplementary file 1 [file emmm0005-0531-sd1.pdf]

Fig 2A

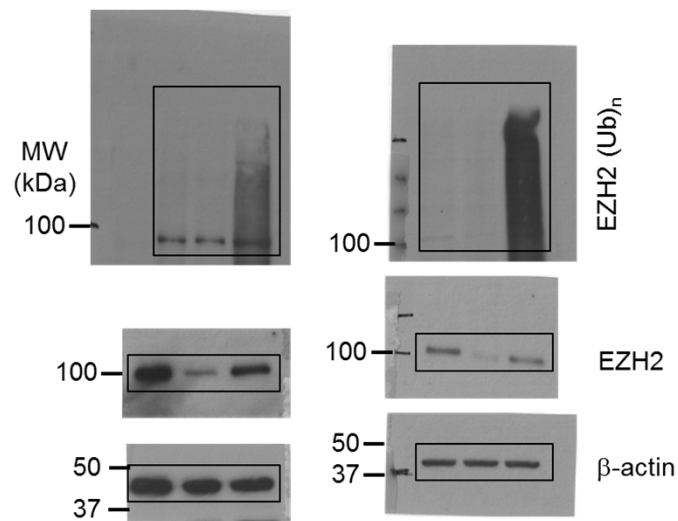

Fig 2E

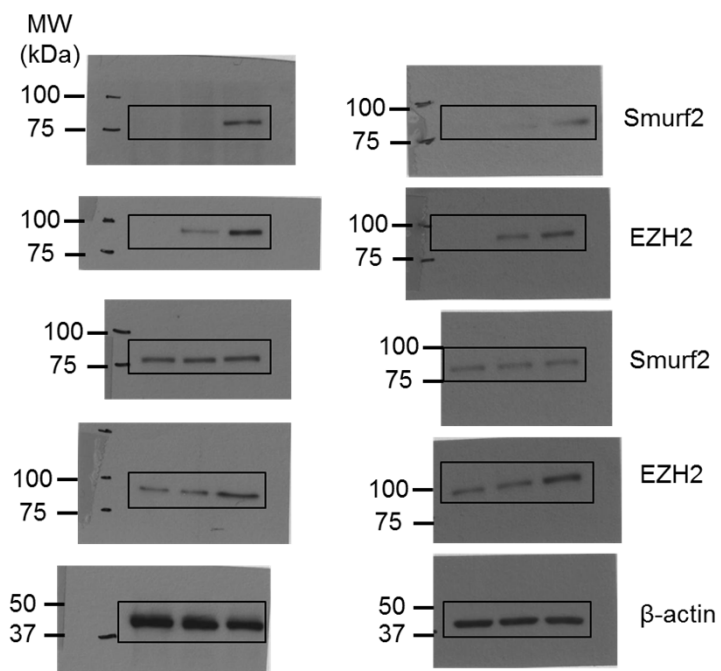

Supplement: Supplementary file 2 [file emmm0005-0531-sd2.pdf]

Fig 3A

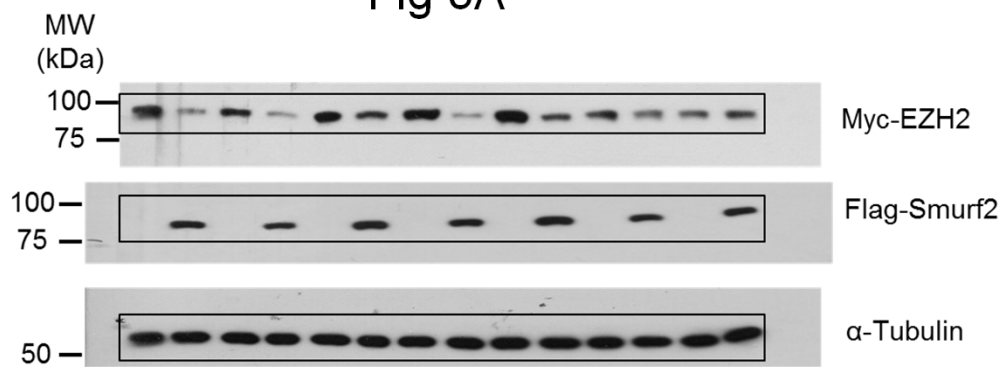

Fig 3B

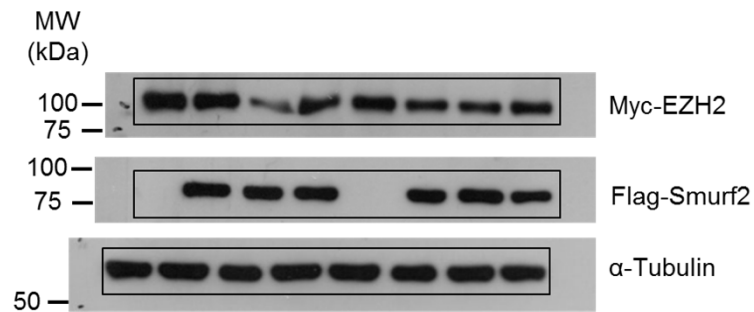

Fig 3E

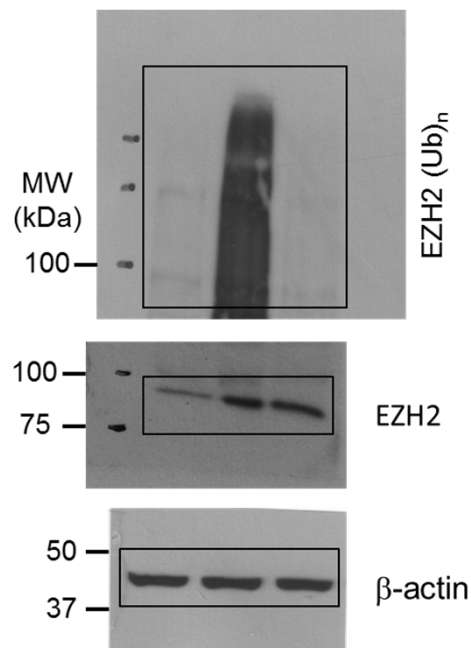

Supplement: Supplementary file 3 [file emmm0005-0531-sd3.pdf]

Fig 5E

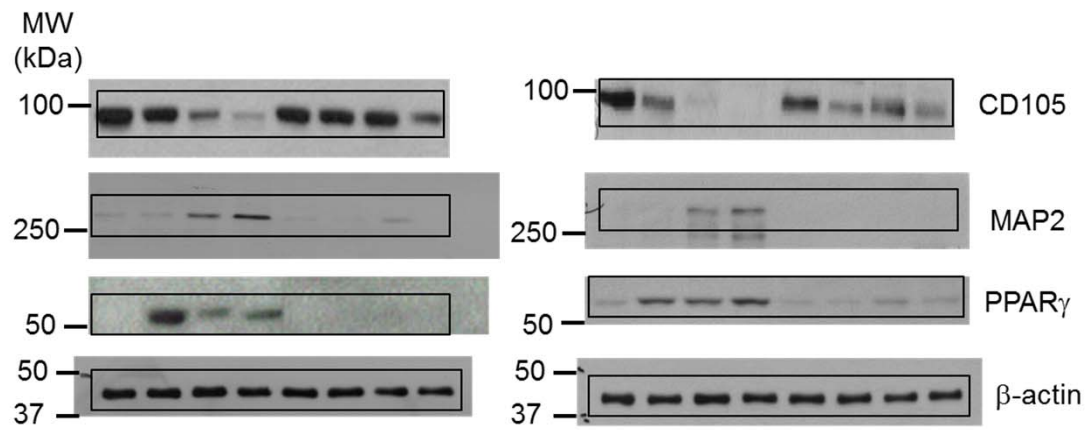

Fig 5F

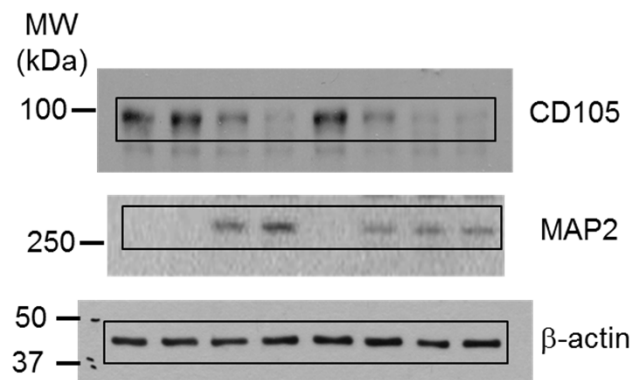

Supplement: Supplementary file 4 [file emmm0005-0531-sd4.pdf]
