# Supplementary material for: Smurf2-mediated degradation of EZH2 enhances neuron differentiation and improves functional recovery after ischaemic stroke
Source: EMBO Mol Med. 2013 Mar 25;5(4):531–47. doi: 10.1002/emmm.201201783 (PMC3628108; doi:10.1002/emmm.201201783)
Supplement: Supplementary file 5 [file emmm0005-0531-sd5.pdf]

Smurf2-mediated degradation of EZH2 enhances neuron differentiation and improves functional recovery after ischemic stroke

Yung-Luen Yu, Ruey-Hwang Chou, Woei-Cherng Shyu, Shu-Ching Hsieh, Chen-Shiou Wu, Shu-Ya Chiang, Wei-Jung Chang, Jia-Ni Chen, Yen-Ju Tseng, Yu-Hsuan Lin, Wei Lee, Su-Peng Yeh, Jennifer L. Hsu, Cheng-Chieh Yang, Shih-Chieh Hung, Mien-Chie Hung

Corresponding author: Mien-Chie Hung, MD Anderson Cancer Center

Review timeline:

Submission date:	25 July 2012
Editorial Decision:	29 August 2012
Revision received:	28 November 2013
Editorial Decision:	04 January 2013
Revision received:	16 January 2013
Accepted:	17 January 2013

Transaction Report:

(Note: With the exception of the correction of typographical or spelling errors that could be a source of ambiguity, letters and reports are not edited. The original formatting of letters and referee reports may not be reflected in this compilation.)

Editor: Anneke Funk / Natascha Bushati

1st Editorial Decision 29 August 2012

Thank you for the submission of your manuscript "Smurf2-mediated degradation of EZH2 enhances neuron differentiation of mesenchymal stem cells and improves functional recovery after ischemic neuronal injuries" to EMBO Molecular Medicine. We have now heard back from the referees whom we asked to evaluate your manuscript. You will see that they find the topic of your manuscript potentially interesting. However, they also raise significant concerns on the study, which should be addressed in a major revision of the manuscript.

In particular, the reviewers highlight that the neuronal differentiation of the hMSCs should be further characterized and that the hMSCs should be investigated for neuronal characteristics after transplantation. In addition, both reviewers highlight that the microarray and ChIP-on-chip data need to be more extensively analyzed and described.

Given the balance of these evaluations, we feel that we can consider a revision of your manuscript if you can convincingly address the issues that have been raised within the time constraints outlined below.

Revised manuscripts should be submitted within three months of a request for revision. They will otherwise be treated as new submissions, unless arranged otherwise with the editor.

I look forward to seeing a revised form of your manuscript as soon as possible.

***** Reviewer's comments *****

Referee #1:

In their manuscript ≥Smurf2-mediated degradation of Ezh2 enhances neuron differentiation of mesenchymal stem cells and improves functional recovery after ischemic neuronal injuries≤, Hung and coworkers expand on a previous study showing that downregulation of Ezh2 enhances differentiation of human mesenchymal stem cells (hMSCs) into neurons. In the present study, they provide evidence that Ezh2 is degraded through the ubiquitin-proteasome pathway during neuronal differentiation, and that the E3 ligase Smurf2 is responsible for ubiquitination of Ezh2 at lysine 421. Smurf2 is required for neuronal differentiation, presumably through its action on Ezh2. The authors go on to identify Ezh2 target genes related to the differentiation process by performing ChIP-on-chip experiments. They identify PPARgamma as the target gene with the highest change in expression, and knockdown of PPARgamma hampers neuronal differentiation. Finally, the authors show that hMSCs that have been depleted for Ezh2 display enhanced functional recovery in implantation post cerebral ischemia.

The experiments performed are overall of high technical quality. It is, however, not always evident how many times an experiment has been performed as error bars are either very small or absent in some panels. The conclusions drawn in each subsection are well supported by the data. Still, some additional controls should be performed. As a major issue, the study appears to combine various story lines and at times the connection between the different parts seems forced. The animal studies are interesting, but at present it seems unclear whether the injected cells indeed differentiate into neurons at the site of injection. PPARgamma had previously been implicated in regeneration after ischemia/reperfusion, and the authors should set their data into the context of existing studies. The findings regarding the degradation of Ezh2 are novel and of interest to the chromatin field, whereas the animal model studies are of potential interest from a medical perspective. Significant improvement of the manuscript, however, would be required for publication in EMBO Molecular Medicine.

Major criticism:

1.) It would be helpful if neuronal differentiation could be further analyzed by monitoring cell morphology, at least in some of the Figures in the context of Ezh2 knockdown and enhanced differentiation and Smurf2 KD and diminished differentation. Do morphological changes parallel upregulation of neuronal markers upon knockdown of Ezh2 (Figure 1)? Related to that question, are the cells more prone to differentiation in general upon Ezh2 knockdown, or is that limited to the neuronal lineage? Genes from other lineages such as e.g. heart should be analyzed as negative controls to probe specificity for neuronal differentiation.

2.) With regard to the ChIP-on-chip studies, the authors focus on 35 genes involved in neuronal differentiation. What are the GO terms of the remaining 4,082 genes that lose Ezh2 binding upon differentiation, and what genes are present at the sites that gain Ezh2 binding? How does Ezh2 bind to those promoters despite degradation? Can the authors estimate the amount of remaining Ezh2 protein in the differentiated cells? Do the genes that lose Ezh2 binding retain H3K27me3, do they become repressed by other means instead or do they gain expression?

It is understood that even though addressing these questions would add significantly to the paper, some are likely beyond the scope of this study. However, more in-depth analysis of the microarray and ChIP-on-chip data should be feasible and will answer some of these questions. Moreover, analysis of H3K27me3 status at a selection of genes that stay repressed would be highly informative and comparatively easy to do as well. H3K9me3 could be analyzed as well to probe whether different repression mechanisms take over the role of Ezh2 if H3K27me3 cannot be maintained.

3.) Does loss of Ezh2 at the PPARgamma promoter correlate with loss of H3K27me3? Does downregulation of PPARgamma after its initial peak involve rebinding of Ezh2 and reestablishment of H3K27me3 or are other pathways involved? The ChIP in Figure 4C suggests rebinding.

4.) It would strengthen the paper if the authors could provide evidence that the hMSCs injected into rats indeed differentiate into neurons at their point of action rather than augment the regeneration of

endogenous neurons in other ways.

5.) To further test the predictions of their model, the authors should test whether rosiglitazone can augment recovery of injuries by wild-type hMSC injection. Circumventing Ezh2 knockdown would make this approach much easier in a therpeutical context.

6.) In the discussion, the authors should put their findings regarding PPARgamma in the context of what is known about its role in regeneration after ischemia/reperfusion. Likewise, the authors should connect the results from their present study with the preceding one implicating Ezh2 in suppression of calcium signaling prior to differentiation.

Minor points:

1.) The writing of the manuscript should be improved. The meaning of some sentences is hard to grasp due to use of unsuitable expressions and grammatical issues. Moreover, the connection between the different sets of data needs to be strengthened at least in the writing. For example, the transition from Figure 1 to Figure 2 is especially confusing. The authors describe downregulation of Ezh2 by shRNA in Figure 1, to proceed stating that downregulation of Ezh2 did not affect its mRNA level in Figure 2. It is not clear to the reader that the authors refer to the normal differentiation process in the absence of Ezh2 knockdown in the latter case.

2.) How was densitometry of Western blots performed? Was it based on film or performed with a CCD-based system? Some bands appear to be saturated. For figures with densitometry data (for example Fig. 1B, 3A, 3C, 5E-G, S1), the authors should include error bars and state in the legends how many times the respective experiments were performed.

3.) The blots shown in Figure 3C are not ideal for densitometry, given for example the black spot in the K421R 8 hrs lane. If it was included in the densitometry, it would distort the data and the interpretation. Different blots should be analyzed. In the graph below it is not clear how many times the experiment was performed.

4.) The reference to the endogenous IP of Smurf2 and Ezh2 is given as Fig 2D not 2E in the text on page 9.

5.) The authors should enable the reader to access their ChIP-on-chip and microarray data in an unprocessed way by depositing the data set to suitable databases. How were thresholds set for Ezh2 peaks in ChIP assays? The methods section does not provide sufficient information to this end.

6.) The data on acceleration of differentiation with rosiglitazone presented in Figure 5F are not convincing. From the present data, it is unclear whether the effect is significant or not. To a lesser extent this also applies to Figure 5G.

7.) The statements on miR and phosphorylation regulation of Ezh2 in the Discussion are redundant with the Introduction and might be cut at either place.

Referee #2:

The authors report that the Polycomb protein EZH2 is a target of Smurf2-mediated ubiquitination and consequent degradation. ChIP-on-chip experiments revealed that PPARgamma expression was induced upon EZH2's knock-down. Transplantion of human mesenchymal stem cells (hMSC) with reduced EZH2 levels in rats subjected to experimental stroke improved their functional recovery. Overall, the experiments are well conducted and the conclusions supported by the data. However, there are several issues that need to be addressed before considering the manuscript further.

1.Throughout the study, neuronal differentiation of hMSC is evaluated by monitoring few molecular markers. Since transplantation of hMSC with EZH2 knocked-down results in functional

neurological improvement after a stroke, the authors should better characterize these cells both in molecular and cellular terms (morphology, dendritic arborization, migration, etc.

2.A better description of the results obtained by microarray and ChIP-on-chip experiments is essential, including replicate, statistical treatment (significance, FDR,p-values), gene ontology etc.

3.How many animals were employed for the transplantation studies?

4.After transplantation, the hMSC cells (control and EZH2 knock-down) should be investigated for the potential acquisition of neuronal characteristics.

1st Revision - authors' response 28 November 2013

Referee #1

In their manuscript "Smurf2-mediated degradation of Ezh2 enhances neuron differentiation of mesenchymal stem cells and improves functional recovery after ischemic neuronal injuries", Hung and co-workers expand on a previous study showing that down regulation of Ezh2 enhances differentiation of human mesenchymal stem cells (hMSCs) into neurons. In the present study, they provide evidence that Ezh2 is degraded through the ubiquitin-proteasome pathway during neuronal differentiation, and that the E3 ligase Smurf2 is responsible for ubiquitination of Ezh2 at lysine 421. Smurf2 is required for neuronal differentiation, presumably through its action on Ezh2. The authors go on to identify Ezh2 target genes related to the differentiation process by performing ChIP-on-chip experiments. They identify PPARgamma as the target gene with the highest change in expression, and knockdown of PPARgamma hampers neuronal differentiation. Finally, the authors show that hMSCs that have been depleted for Ezh2 display enhanced functional recovery in implantation post cerebral ischemia.

The experiments performed are overall of high technical quality. It is, however, not always evident how many times an experiment has been performed as error bars are either very small or absent in some panels. The conclusions drawn in each subsection are well supported by the data. Still, some additional controls should be performed. As a major issue, the study appears to combine various story lines and at times the connection between the different parts seems forced. The animal studies are interesting, but at present it seems unclear whether the injected cells indeed differentiate into neurons at the site of injection. PPARgamma had previously been implicated in regeneration after ischemia/reperfusion, and the authors should set their data into the context of existing studies. The findings regarding the degradation of Ezh2 are novel and of interest to the chromatin field, whereas the animal model studies are of potential interest from a medical perspective. Significant improvement of the manuscript, however, would be required for publication in EMBO Molecular Medicine.

Response: We are deeply appreciative of the reviewers' comments and that he/she feels our findings regarding EZH2 degradation are novel and our animal model studies are of potential interest from a medical perspective. We have addressed the reviewer's concerns regarding the experimental controls, differentiation of injected cells, number of times in which experiments were conducted, and connection between the stories in details below.

Major criticism:

Point #1: *It would be helpful if neuronal differentiation could be further analyzed by monitoring cell morphology, at least in some of the Figures in the context of Ezh2 knockdown and enhanced differentiation and Smurf2 KD and diminished differentation. Do morphological changes parallel upregulation of neuronal markers upon knockdown of Ezh2 (Figure 1)? Related to that question, are the cells more prone to differentiation in general upon Ezh2 knockdown, or is that limited to the*

neuronal lineage? Genes from other lineages such as e.g. heart should be analyzed as negative controls to probe specificity for neuronal differentiation.

Response to Point #1: We thank the reviewer for this suggestion and have performed new experiments to address these points. We first analyzed primary bone marrow–derived hMSCs and demonstrated that they exhibit cell body morphologies with extended neurite-like structures under neuronal induction medium (NIM; Fig 1A, bottom left). The hMSC-derived neuronal cells were then stained with the MAP2 (neuron marker) for immunocytochemical analysis to further validate neuron differentiation (Fig 1A, right). We also analyzed cell morphology in EZH2 and Smurf2 knockdown hMSCs in the NIM. In EZH2 knockdown cells, neuron differentiation was accelerated, occurring at day 3 compared to day 5 in the mock (Fig. 1D). In contrast, in Smurf2 knockdown cells, no differentiation was observed compared to mock on day 5 (Fig. 3G). These morphological changes were parallel to the upregulation or downregulation of neuronal makers shown in Figures 1C and 3F. We have added the following new data (Figs. 1A, 1D and 3G) to the revised manuscript.

We thank the reviewer for the question on whether differentiation upon EZH2 knockdown is a general phenomenon or is limited to the neuronal lineage. We found that EZH2 knockdown hMSCs are more prone to differentiation into the neuronal lineage as indicated by the increase in MAP2 (neuron marker) gene expression after induction in NIM but not expression of genes from other lineages examined, such as troponin T (TnT; cardiac marker) and osteopontin (OPN; osteogenic marker), by qPCR analysis (Fig 1E).

Point #2: *With regard to the ChIP-on-chip studies, the authors focus on 35 genes involved in neuronal differentiation. What are the GO terms of the remaining 4,082 genes that lose Ezh2 binding upon differentiation, and what genes are present at the sites that gain Ezh2 binding? How does Ezh2 bind to those promoters despite degradation? Can the authors estimate the amount of remaining Ezh2 protein in the differentiated cells? Do the genes that lose Ezh2 binding retain H3K27me3, do they become repressed by other means instead or do they gain expression? It is understood that even though addressing these questions would add significantly to the paper, some are likely beyond the scope of this study. However, more in-depth analysis of the microarray and ChIP-on-chip data should be feasible and will answer some of these questions. Moreover, analysis of H3K27me3 status at a selection of genes that stay repressed would be highly informative and comparatively easy to do as well. H3K9me3 could be analysed as well to probe whether different repression mechanisms take over the role of Ezh2 if H3K27me3 cannot be maintained.*

Response to Point #2: We thank the reviewer for pointing out this important issue. We have performed more in-depth analysis of the gene microarray and ChIP-on-chip data shown in Fig 4A as well as in Supporting Information Tables S1 to S6 (heatmap, gene ontology (GO) terms, and genes list). The answers to the reviewer's questions are as follows:

1. "What are the GO terms of the remaining 4,082 genes that lose Ezh2 binding upon differentiation, and what genes are present at the sites that gain Ezh2 binding?"

To answer this question, we further analyzed these 4,082 genes that lose EZH2 binding upon differentiation by Ingenuity System Analysis. The 78 GO terms (Supporting Information Table S2) identified from a total 3,110 genes, including 388 overlapping genes (peak score > 0.2 and FDR < 0.05; Supporting Information Table S3), were classified into 10 groups including 27 overlapping genes in nervous system (1.5%; Fig 4A and Supporting Information Table S2).

2. "How does Ezh2 bind to those promoters despite degradation? Can the authors estimate the

© EMBO

amount of remaining Ezh2 protein in the differentiated cells?"

To answer this question, we found that the protein level of EZH2 was decreased during neuron differentiation (Fig 1C), and the amount of remaining EZH2 protein in the differentiated 3A6-hMSCs or primary hMSCs cells was estimated to be about 20%. (Fig 1C, bottom).

3."Do the genes that lose Ezh2 binding retain H3K27me3, do they become repressed by other means instead or do they gain expression? Moreover, analysis of H3K27me3 status at a selection of genes that stay repressed would be highly informative and comparatively easy to do as well. H3K9me3 could be analyzed as well to probe whether different repression mechanisms take over the role of Ezh2 if H3K27me3 cannot be maintained."

To answer this question, we further validated the enrichment profiles of selected EZH2 target genes including that were bound by EZH2 in undifferentiated hMSCs (PPARγ, PIP5K1C) or overlapped in both undifferentiated and neuron-differentiated hMSCs (RUNX2, as control) after neuron differentiation (Supporting Information Table S1) and quantitated EZH2 and two molecular marks, trimethylation of histone H3 at lysine 27 (H3K27me3) and trimethylation of histone H3 at lysine 9 (H3K9me3), that are associated with transcriptional silencing enrichments (Fig 4B, C and D). The results from qChIP analysis were similar to those from the ChIP-on-chip analysis (Supporting Information Table S1) showing that loss of EZH2 binding and H3K27me3 occurred at the same time at both PPARγ (Fig 4B) and PIP5K1C (Fig 4C) promoters during neuron differentiation of hMSCs. Our data indicated that EZH2 was released with a concomitant decrease in H3K27me3 at the PPARγ promoter on day 1, and by day 5, both EZH2 and H3K27me3 were slightly recovered, likely to maintain functional PPARγ and PIP5K1C level after neuron differentiation (Fig 4B and C). The H3K9me3 status at these two EZH2 target genes, however, did not change during neuron differentiation, and thus it may not take over the role of EZH2 if H3K27me3 cannot be maintained. We did not observe loss of binding of either EZH2 or two histone trimethylation marks (H3K27me3 and H3K9me3) at the RUNX2 promoter during neuron differentiation (Fig 4D) which is not surprising as we previously demonstrated that this EZH2 target gene is important for osteogenic hMSC differentiation (Wei et al, 2011).

Point #3: *Does loss of Ezh2 at the PPARgamma promoter correlate with loss of H3K27me3? Does down regulation of PPARgamma after its initial peak involve rebinding of Ezh2 and reestablishment of H3K27me3 or are other pathways involved? The ChIP in Figure 4C suggests rebinding.*

Response to Point #3: We thank the reviewer for raising this important point. Indeed, loss of EZH2 at the PPARγ promoter correlated with loss of H3K27me3 (Fig 4B). We also found that the strongest binding of EZH2 and H3K27me3 to PPARγ promoter was lost on day 1 which was slightly reestablished on day 5 and compared to IgG control of hMSCs after neuron differentiation (Fig 4B and S4). Fig S4 of the revised version is Fig 4C from the prior submission.

Point #4*: It would strengthen the paper if the authors could provide evidence that the hMSCs injected into rats indeed differentiate into neurons at their point of action rather than augment the regeneration of endogenous neurons in other ways.*

Response to Point #4: We thank the reviewer for raising this important point. To show that hMSCs injected into rats differentiated into neurons at their point of action, we implanted hMSCs with or without knockdown of EZH2 to the ischemic rat brains for 3 weeks and examined the expression of

MAP2 (neuron marker) in their tissue sections. Three-dimensional images from the colocalization study showed exogenous implanted bisbenzimide-labeled hMSCs (blue fluorescence), PPARγ (red fluorescence) and MAP2 (green fluorescence) positive cells (PPARγ$^+$/MAP2$^+$/bisbenzimide$^+$) in implanted hMSCs as indicated by the arrows (Fig 7H, top). Co-expression of human nuclear antigen (hNA) in the exogenous implanted bisbenzimide-labeled hMSCs further confirmed that cells were of human origin (Fig 7I). A significant increase in the numbers of PPARγ$^+$/MAP2$^+$/bisbenzimide$^+$ cells with dendritic arborization was found in the hMSCs/shEZH2-treated rats compared to the hMSCs/Mock-treated rats (Fig 7H, top; inset, bottom left). Quantitative analysis showed that the implanted MAP2-positive cells is significantly higher in EZH2-silenced hMSCs than in the mock-treated hMSCs *in vivo* (Fig 7H, bottom right).

Point #5: *To further test the predictions of their model, the authors should test whether rosiglitazone can augment recovery of injuries by wild-type hMSC injection. Circumventing Ezh2 knockdown would make this approach much easier in a therapeutical context.*

Response to Point #5: We thank the reviewer for the suggestion to test whether rosiglitazone can augment recovery of injuries by wild-type hMSC injection. Indeed, rats that received the hMSCs+rosiglitazone treatment exhibited significantly better improvement in neurological behaviors (body asymmetry trials, locomotor activity tests and grip strength measurement) than rats that received hMSCs or rosiglitazone alone (Fig 8B to F). In addition, hMSCs+rosiglitazone treatment also increased the numbers of exogenous implanted MAP2$^+$/bisbenzimide$^+$ neurons in the rats that received this treatment (Fig 8G). These findings are in agreement with the reviewer's suggestion that the use of rosiglitazone with wild-type hMSCs is an easier approach in a therapeutical context.

Point #6: *In the discussion, the authors should put their findings regarding PPARgamma in the context of what is known about its role in regeneration after ischemia/reperfusion. Likewise, the authors should connect the results from their present study with the preceding one implicating Ezh2 in suppression of calcium signalling prior to differentiation.*

Response to Point #6: We thank the reviewer for the comment. We have described PPARγ in the context of what is known about its role in regeneration after ischemia/reperfusion and addressed the connection between the present study and the preceding one implicating EZH2 in suppression of calcium signalling prior to differentiation in the Discussion section as shown below:

"In our previous study, we demonstrated that after induction to neuron differentiation, decreased EZH2 leads to hMSC differentiation into functional neuron lineage. We also provided evidence to establish that PIP5K1C is transcriptionally suppressed by EZH2 and silencing EZH2 enhanced neuron differentiation might be mediated via one of the pathways by which activation of PIP5K1C to evoke intracellular Ca^{2+} signaling (Yu et al, 2011). Therefore, EZH2 may suppress several key target genes that are involved and/or crosstalk with Ca^{2+} signaling in neuron differentiation from hMSCs. Furthermore, our genome-wide analysis of ChIP-on-chip and gene microarray identified one EZH2 target gene, PPARγ, which is bound by EZH2 in undifferentiated hMSCs and upregulated during neuron differentiation (Fig 4A and B) among several other target genes when EZH2 is knocked down in hMSCs (Supporting Information Table S1 and Table S3). Indeed, it has been reported that PPARγ plays an important role in regulating neuron differentiation of embryonic midbrain cells, and a deficiency in PPARγ is associated with ischemic brain damage (Zhao et al, 2009). However, the underlying molecular mechanisms remain to be elucidated. In addition, PPARγ agonists rosiglitazone and 15d-PGJ2 have been reported to effectively attenuate tissue damage caused by ischemia reperfusion in animal models (Lin et al, 2006; Shimazu et al, 2005; Sundararajan et al, 2005) and may modulate Ca^{2+} signaling and homeostasis in neurons (Pancani et al, 2009). PPARγ-mediated 14-3-3ε upregulation also plays a pivotal role in neuroprotection and the

beneficial effects of rosiglitazone against ischemic stroke (Wu et al, 2009). Our data showed that knockdown of PPARγ inhibited cells from undergoing efficient neuron differentiation and arrested them at the MSC stage (Figs 5B to E) and that physiological concentrations of the PPARγ agonist rosiglitazone and ectopic expression of PPARγ accelerated neuron differentiation of hMSCs *in vitro* (Fig 5F and G). Therefore, PPARγ agonist treatment after hMSCs implantation may augment recovery of injuries by hMSC injection via enhance neuron differentiation *in vivo* (Fig 8B to G)."

Minor points:

Point #1: *The writing of the manuscript should be improved. The meaning of some sentences is hard to grasp due to use of unsuitable expressions and grammatical issues. Moreover, the connection between the different sets of data needs to be strengthened at least in the writing. For example, the transition from Figure 1 to Figure 2 is especially confusing. The authors describe downregulation of Ezh2 by shRNA in Figure 1, to proceed stating that downregulation of Ezh2 did not affect its mRNA level in Figure 2. It is not clear to the reader that the authors refer to the normal differentiation process in the absence of Ezh2 knockdown in the latter case.*

Response to Point #1: We thank the reviewer for the comment. The revised manuscript has been edited by Dr. Tamara K. Locke at the Scientific Publications office at MD Anderson Cancer to improve the writing. We also changed the wordings in the text to strengthen the connection between the different sets of data in the revised manuscript.

Point #2: *How was densitometry of Western blots performed? Was it based on film or performed with a CCD-based system? Some bands appear to be saturated. For figures with densitometry data (for example Fig. 1B, 3A, 3C, 5E-G, S1), the authors should include error bars and state in the legends how many times the respective experiments were performed.*

Response to Point #2: We thank the reviewer for pointing out the issue. Indeed, the densitometry of Western blots was performed based on film. We have replaced several Western blots that had bands that appeared saturated with new blots and included error bars. We also stated in the legends that three independent experiments were performed (Figs 1C, 3A, 3C, 5D-G, and S2). Fig 1C and S2 of the revised version are Fig 1B and S1 from the prior submission.

Point #3: *The blots shown in Figure 3C are not ideal for densitometry, given for example the black spot in the K421R 8 hrs lane. If it was included in the densitometry, it would distort the data and the interpretation. Different blots should be analysed. In the graph below it is not clear how many times the experiment was performed.*

Response to Point #3: We thank the reviewer for pointing out the issue. We have repeated the Western blot to obtain more suitable blots for densitometric analysis. We also included error bars and indicated in the legends that three independent experiments were performed.

Point #4: *The reference to the endogenous IP of Smurf2 and Ezh2 is given as Fig 2D not 2E in the text on page 9.*

Response to Point #4: We apologize for the error and have corrected it in the revised manuscript.

Point #5: *The authors should enable the reader to access their ChIP-on-chip and microarray data in an unprocessed way by depositing the data set to suitable databases. How were thresholds set for Ezh2 peaks in ChIP assays? The methods section does not provide sufficient information to this end.*

Response to Point #5: We thank the reviewer for the comment. We have uploaded and deposited our dataset to Gene Expression Omnibus (GEO) databases to provide access to our ChIP-on-chip and microarray data in an unprocessed way (pending accession number). We have added the thresholds for the ChIP assays and modified the Materials and Methods section to include more detailed information how the thresholds were set (Page 21 of revised manuscript).

Point #6: *The data on acceleration of differentiation with rosiglitazone presented in Figure 5F are not convincing. From the present data, it is unclear whether the effect is significant or not. To a lesser extent this also applies to Figure 5G.*

Response to Point #6: The reviewer raised a valid concern regarding the data shown in Figs 5F and 5G. To provide more convincing data, we have replaced and repeated several Western blots with new blots and included the error bars from three independent experiments (Figs 5F and G).

Point #7: *The statements on miR and phosphorylation regulation of Ezh2 in the Discussion are redundant with the Introduction and might be cut at either place.*

Response to Point #7: We thank the reviewer for the comment. We have removed information already mentioned in the Introduction from the Discussion section.

<u>Referee #2:</u>

The authors report that the Polycomb protein EZH2 is a target of Smurf2-mediated ubiquitination and consequent degradation. ChIP-on-chip experiments revealed that PPARgamma expression was induced upon EZH2's knock-down. Transplantion of human mesenchymal stem cells (hMSC) with reduced EZH2 levels in rats subjected to experimental stroke improved their functional recovery. Overall, the experiments are well conducted and the conclusions supported by the data. However, there are several issues that need to be addressed before considering the manuscript further.

Point #1: *Throughout the study, neuronal differentiation of hMSC is evaluated by monitoring few molecular markers. Since transplantation of hMSC with EZH2 knocked-down results in functional neurological improvement after a stroke, the authors should better characterize these cells both in molecular and cellular terms (morphology, dendritic arborisation, migration, etc.*

Response to Point #1: We thank the reviewer for raising this important point. To better characterize these cells, we have added new data (Figs. 1A, 1D, and 7H) that show changes in cell morphology and dendritic arborization after induction in the NIM. We first analyzed primary bone marrow–derived hMSCs and demonstrated that they exhibit cell body morphologies with extended neurite-like structures in NIM (Fig 1A, bottom left). The hMSC-derived neuronal cells were then stained with the MAP2 (neuron marker) for immunocytochemical analysis to further validate neuron differentiation with dendritic arborization (Fig 1A, right; green fluorescence). We also analyzed cell morphology in EZH2 knockdown hMSCs in NIM and showed that neuron differentiation with dendritic arborization was enhanced (Fig 1D).

To further monitor the cell morphology and dendritic arborization *in vivo*, we implanted hMSCs with or without EZH2 knockdown in brains of ischemic rats for 3 weeks and examined the expression of MAP2 (neuron marker) in their tissue sections. Three-dimensional images from the colocalization study showed exogenous implanted bisbenzimide-labeled hMSCs (blue fluorescence), PPARγ (red fluorescence) and MAP2 (green fluorescence) positive cells (PPARγ$^+$/MAP2$^+$/bisbenzimide$^+$) in implanted hMSCs as indicated by the arrows (Fig 7H, top). A significant increase in the numbers of PPARγ$^+$/MAP2$^+$/bisbenzimide$^+$ cells with dendritic arborization was found in the hMSCs/shEZH2-treated rats compared to the hMSCs/Mock-treated rats (Fig 7H, top; insets). Quantitative analysis showed that the implanted MAP2-positive cells is significantly higher in EZH2-silenced hMSCs than in the mock-treated hMSCs *in vivo* (Fig 7H, bottom right).

Point #2: *A better description of the results obtained by microarray and ChIP-on-chip experiments is essential, including replicate, statistical treatment (significance, FDR,p-values), gene ontology etc.*

Response to Point #2: We thank the reviewer for pointing out this important issue. We have performed more in-depth analysis of the gene microarray and ChIP-on-chip data and included better description of the analysis as shown below as well as on page 10-11 of the Results section of the revised manuscript.

"For ChIP-on-chip, our initial screening revealed that among 28,869 promoters in the human genome (HG18; NCBI Build 36), 4,506 of 6,249 (15.6%; 4,082 undifferentiated + 424 overlapped) were bound by EZH2 in undifferentiated hMSCs and 1,743 of 6,249 (6.0%; 1,319 neuron-differentiated + 424 overlapped) were bound by EZH2 in neuron-differentiated hMSCs (a peak score >0.2 was considered as high-confidence binding sites for EZH2; Supporting Information Fig S3). These EZH2 target genes are listed as heatmap as shown in Supporting Information Table S1. We further analyzed these 4,082 genes that lose EZH2 binding upon differentiation by Ingenuity System Analysis. The 78 GO terms (Supporting Information Table S2) identified from a total 3,110 genes, including 388 overlapping genes (peak score > 0.2 and FDR < 0.05; Supporting Information Table S3), were classified into 10 groups including 27 overlapping genes in nervous system (1.5%; Fig 4A and Supporting Information Table S2).

In addition, the gene microarray analysis (Human Whole Genome OneArray) from 29,178 human genome probes showed that the expression of 815 genes from hMSCs/shEZH2 were upregulated ≥ 2 fold compared with the shMSCs/Mock control (listed as heatmap in Supporting Information Table S4). GO analysis was performed by using the Biological Process category. The 218 GO terms identified from the 1,423 genes including 370 overlapping genes (log2(Ratio) >1 and p-value < 0.05; Supporting Information Table S5 and S6) were classified into 10 groups including 20 overlapping genes in nervous system (2.7%; Fig 4A and Supporting Information Table S5)."

Point #3: *How many animals were employed for the transplantation studies?*

Response to Point #3: We apologize for not including this information. We used 10 SD rats (N=10) for each condition in this study and indicated this in the legends. We have also included error bars in the figures (Fig 7 and 8).

Point #4: *After transplantation, the hMSC cells (control and EZH2 knock-down) should be investigated for the potential acquisition of neuronal characteristics.*

Response to Point #4: We thank the reviewer for the suggestion. Please see response to Point #1.

References:

Lin TN, Cheung WM, Wu JS, Chen JJ, Lin H, Liou JY, Shyue SK, Wu KK (2006) 15d-prostaglandin J2 protects brain from ischemia-reperfusion injury. Arterioscler Thromb Vasc Biol 26(3): 481-487

Pancani T, Phelps JT, Searcy JL, Kilgore MW, Chen KC, Porter NM, Thibault O (2009) Distinct modulation of voltage-gated and ligand-gated Ca2+ currents by PPAR-gamma agonists in cultured hippocampal neurons. J Neurochem 109(6): 1800-1811

Shimazu T, Inoue I, Araki N, Asano Y, Sawada M, Furuya D, Nagoya H, Greenberg JH (2005) A peroxisome proliferator-activated receptor-gamma agonist reduces infarct size in transient but not in permanent ischemia. Stroke 36(2): 353-359

Sundararajan S, Gamboa JL, Victor NA, Wanderi EW, Lust WD, Landreth GE (2005) Peroxisome proliferator-activated receptor-gamma ligands reduce inflammation and infarction size in transient focal ischemia. Neuroscience 130(3): 685-696

Wei Y, Chen YH, Li LY, Lang J, Yeh SP, Shi B, Yang CC, Yang JY, Lin CY, Lai CC, Hung MC (2011) CDK1-dependent phosphorylation of EZH2 suppresses methylation of H3K27 and promotes osteogenic differentiation of human mesenchymal stem cells. Nat Cell Biol 13(1): 87-94

Wu JS, Lin TN, Wu KK (2009) Rosiglitazone and PPAR-gamma overexpression protect mitochondrial membrane potential and prevent apoptosis by up regulating anti-apoptotic Bcl-2 family proteins. J Cell Physiol 220(1): 58-71

Yu YL, Chou RH, Chen LT, Shyu WC, Hsieh SC, Wu CS, Zeng HJ, Yeh SP, Yang DM, Hung SC, Hung MC (2011) EZH2 regulates neuronal differentiation of mesenchymal stem cells through PIP5K1C-dependent calcium signalling. J Biol Chem 286(11): 9657-9667

Zhao X, Strong R, Zhang J, Sun G, Tsien JZ, Cui Z, Grotta JC, Aronowski J (2009) Neuronal PPARgamma deficiency increases susceptibility to brain damage after cerebral ischemia. J Neurosci 29(19): 6186-6195

1st Editorial Decision 04 January 2013

Thank you for the submission of your revised manuscript to EMBO Molecular Medicine. We have now received the enclosed reports from the referees that were asked to re-assess it. As you will see the reviewers are now globally supportive and I am pleased to inform you that we will be able to accept your manuscript pending the following final amendments:

- Please change the abstract according to reviewer 1's suggestion.

- Please shorten the title.

Please submit your revised manuscript within two weeks. I look forward to seeing a revised form of your manuscript.

***** Reviewer's comments *****

Referee #1 (General Remarks):

Hung and coworkers significantly improved the manuscript in the revised version. A concerns of the reviewers have been addressed in a very thorough and constructive way, and I fully support publication in EMBO Molecular Medicine. The paper provides insight into the role Ezh2 and its degradation in neuronal differentiation, while further exploring the utility of reducing Ezh2 or stimulating its transcriptional target PPARgamma for the enhancement of recovery from cerebral ischemia.

In the revised version, new data has been added to more thoroughly monitor the differentiation process, and the presentation and analysis of the ChIP-on-chip data has been much improved. The authors now show that the transplanted MSCs do differentiate into neurons in situ, and that not only Ezh2 knockdown but also treatment with a PPARgamma agonist augments recovery after cerebral ischemia. The writing of the manuscript has been much improved as well.

As a minor comment, the abstract contains a statement now that PPARgamma is the only gene that changed significantly. However, in both ChIP-on-chip as well as the microarray analysis, many genes change in a significant way (e.g. more than 2fold upregulation of expression by microarray analysis). Nonetheless, PPARgamma seems to be the only gene involved in neuronal differentiation that changes significantly in both its expression status and its modification status during differentiation. The authors might want to consider changing the abstract to better reflect their observations.

Referee #2:

The authors have satisfactorily addressed my comments and concerns.

2nd Revision - authors' response 16 January 2013

Comments from Reviewer #1: *Hung and co-workers significantly improved the manuscript in the revised version. A concerns of the reviewers have been addressed in a very thorough and constructive way, and I fully support publication in EMBO Molecular Medicine. The paper provides insight into the role Ezh2 and its degradation in neuronal differentiation, while further exploring the utility of reducing Ezh2 or stimulating its transcriptional target PPARgamma for the enhancement of recovery from cerebral ischemia. In the revised version, new data has been added to more thoroughly monitor the differentiation process, and the presentation and analysis of the ChIP-on-chip data has been much improved. The authors now show that the transplanted MSCs do differentiate into neurons in situ, and that not only Ezh2 knockdown but also treatment with a PPARgamma agonist augments recovery after cerebral ischemia. The writing of the manuscript has been much improved as well.*

As a minor comment, the abstract contains a statement now that PPARgamma is the only gene that changed significantly. However, in both ChIP-on-chip as well as the microarray analysis, many genes change in a significant way (e.g. more than 2fold up regulation of expression by microarray analysis). Nonetheless, PPARgamma seems to be the only gene involved in neuronal differentiation that changes significantly in both its expression status and its modification status during differentiation. The authors might want to consider changing the abstract to better reflect their observations.

Response to Reviewer's Comments: We are deeply appreciative of the reviewer's comments and have modified the abstract to better reflect our observations as shown below:

© EMBO

"EZH2 plays an important role in stem cell renewal and maintenance by inducing gene silencing via its histone methyltransferase activity. Previously, we showed EZH2 down regulation enhances neuron differentiation of human mesenchymal stem cells (hMSCs); however, the underlying mechanisms of EZH2-regulated neuron differentiation are still unclear. Here, we identify Smurf2 as the E3 ubiquitin ligase responsible for the polyubiquitination and proteasome-mediated degradation of EZH2, which is required for neuron differentiation. ChIP-on-chip screen combined with gene microarray analysis revealed that PPARγ was the only gene involved in neuron differentiation with significant changes in both its modification and expression status during differentiation. Moreover, knocking down PPARγ prevented cells from undergoing efficient neuron differentiation. In animal model, rats implanted with intracerebral EZH2-knocked-down hMSCs or hMSCs plus treatment with PPARγ agonist (rosiglitazone) showed better improvement than did those without EZH2 knockdown or rosiglitazone treatment after a stroke. Together, our results support Smurf2 as a regulator of EZH2 turnover to facilitate PPARγ expression that is specifically required for neuron differentiation, providing a molecular mechanism for clinical applications in the neurodegenerative diseases."
