# Supplementary material for: Smurf2-mediated degradation of EZH2 enhances neuron differentiation and improves functional recovery after ischaemic stroke
Source: EMBO Mol Med. 2013 Mar 25;5(4):531–47. doi: 10.1002/emmm.201201783 (PMC3628108; doi:10.1002/emmm.201201783)
Supplement: Supplementary file 6 [file emmm0005-0531-sd6.pdf]

Supporting Information

Yu et al, Smurf2-mediated degradation of EZH2 enhances neuron differentiation and improves functional recovery after ischemic stroke

Table of Contents:

Supporting Information Methods

Supporting Information Figure S1: Knockdown of EZH2 enhances neuron differentiation of hMSCs.

Supporting Information Figure S2: Smurf2 is a potential E3 ubiquitin ligase of EZH2.

Supporting Information Figure S3: Genome-wide mapping of EZH2 target promoters by ChIP-on-chip.

Supporting Information Figure S4: PPARγ is a EZH2 target genes during neuron differentiation of hMSCs.

Supporting Information Table S1: Heatmap derived from ChIP-on-chip data of EZH2 target genes in hMSCs neuron differentiation.

Supporting Information Table S2: Gene ontology from ChIP-on-chip data of EZH2 target genes in undifferentiated hMSCs.

Supporting Information Table S3: Genes list from gene ontology data of EZH2 target genes in undifferentiated hMSCs by ChIP-on-chip.

Supporting Information Table S4: Heatmap derived from gene microarray data of EZH2 regulated genes in hMSCs.

Supporting Information Table S5: Gene ontology from gene microarray data of EZH2-regulated genes in undifferentiated hMSCs.

Supporting Information Table S6: Genes list from gene ontology data of EZH2 regulated genes in undifferentiated hMSCs by gene microarray.

Supporting Information Methods

Materials

Chemicals used in the induction of neuron differentiation, namely, dexamethasone, ascorbic acid-2-phosphate, indomethacin, insulin, and 3-isobutyl-1-methyl-xanthine, polybrene, and puromycin, were purchased from Sigma-Aldrich (St. Louis, MO, USA). We purchased the following antibodies from the companies listed: anti-CD105, anti-MAP2, AlexFluor 488 conjugated anti-MAP2A and AlexFluor 647 conjugated anti-β-tubulin III (BD Biosciences, San Jose, CA, USA); anti-β-tubulin III (Covance, Princeton, NJ, USA); anti-Neu-N (Chemicon, Billerica, MA, USA); anti-β-actin (Sigma-Aldrich); anti-EZH2, anti-PPARγ (Cell Signaling, Danvers, MA, USA); and anti-Smurf2 (Santa Cruz Biotechnology, Santa Cruz, CA, USA).

Cell culture and induction to differentiation into neuronal lineage

Human mesenchymal stem cells (hMSCs), size-sieved from human bone marrow, were isolated and characterized previously as having multilineage potential to form bone, fat, cartilage (Hung et al, 2002a), and electrically active neural cells (Hung et al, 2002b). The hMSCs were immortalized without neoplastic transformation by transduction with HPV16 E6/E7 genes (Hung et al, 2004). The E6/E7-immortalized hMSC derivative, 3A6, contains a human telomerase reverse transcriptase (hTERT) gene for more stem-like properties (Tsai et al, 2010). The 3A6 cells were grown in Dulbecco's modified Eagle's medium-low glucose (DMEM-LG) (HyClone, MA, USA) with 10% fetal bovine serum, 100 U/ml penicillin, and 100 μg/ml streptomycin in a humidified incubator with 5% CO_2 at 37°C. To induce neuron differentiation, cells were seeded at a density of 4000 cells/cm^2 in the regular medium on the day before the experiment and then treated with serum-free DMEM-high glucose (DMEM-HG) (HyClone) neuronal induction

medium (NIM) containing 10^{-7} M dexamethasone, 50 μg/ml ascorbic acid-2-phosphate, 50 μM indomethacin, 10 μg/ml insulin, and 0.45 mM 3-isobutyl-1-methyl-xanthine for 1-5 days, and the neuronal induction medium was changed every 3 days (Yu et al, 2011).

Western blot analysis

Cells were washed twice with phosphate buffer saline (PBS, containing 137 mM NaCl, 2.7 mM KCl, 10 mM Na_2HPO_4, 2 mM KH_2PO_4), and then lysed in RIPA Buffer (50 mM Tris at pH 7.5, 150 mM NaCl, 1 mM EDTA, 0.25% Na-deoxycholate, 1% NP-40, 1 mM NaF, 1 mM Na_3VO_4, 1 mM PMSF, 1 μg/ml aprotinin) by sonication. The soluble extraction was collected from the supernatant after centrifugation at 15,000 g for 10 min at 4°C. The extract was boiled at 100°C for 5 min, separated by SDS-PAGE, and transferred to a PVDF membrane. Subsequently, the membrane was blocked with 5% skim milk in PBST buffer (PBS containing 0.1% Tween-20) for 1 h at room temperature, and then hybridized with primary antibody with gentle agitation overnight at 4°C. After washing with PBST, the membrane was incubated with HRP-conjugated secondary antibody (Chemicon) for 1 h at room temperature. The immunoreactive bands were visualized by the enhanced chemiluminescence (ECL) detection reagent (GE Healthcare).

RNA extraction, reverse transcriptase-polymerase chain reaction (RT-PCR) and quantitative PCR (qPCR).

Total RNA was extracted with TRIZOL reagent (Invitrogen) according to the manufacturer's instruction. The complementary DNA (cDNA) was synthesized from 5 μg of total RNA in a reaction mixture containing 2.5 μM oligo (dT) primer, 0.5 mM dNTP mixture, 200 U SuperScript III reverse transcriptase, 40 U RNaseOUT, an RNase inhibitor (all from Invitrogen). After

incubation at 50°C for 50 min, the reaction mixture was heat inactivated at 85°C for 5 min and then treated with 2 U RNase H at 37°C for 20 min. The qPCR was performed by using the LightCycler 480 equipment (Roche). Primers were designed using the PrimerFinder software (https://www.roche-applied-science.com/sis/rtpcr/upl/index.jsp?id=UP030000). The primer sets are as follows:

β-actin (ACTB):

5′-ATTGGCAATGAGCGGTTC-3′ (forward)

5′-GGATGCCACAGGACTCCAT-3′ (reverse)

EZH2:

5′-GACTGGCGAAGAGCTGTTTT-3′ (forward)

5′- TCTTTCGATGCCGACATACTT-3′ (reverse)

MAP2:

5′-CTCAACAGTTCTATCTCTTCTTCA-3′ (forward)

5′-TCTTCTTGTTTAAAATCCTAACCT-3′ (reverse)

Troponin T (TnT):

5′-GGACAAGGTGGATGAAGAGA-3′ (forward)

5′- AGGGTGGGCCGCTTAAACT-3′ (reverse)

Osteopontin (OPN):

5′-ACGCCGACCAAGGAAAACTC-3′ (forward)

5′-GTCCATAAACCACACTATCACCTCG-3′ (reverse)

Smurf2:

5′- AGATTTTTACGAGGCATTGAGG-3′ (forward)

5′- TCCAAGTCCACAAATAATGAGC-3′ (reverse)

PPARγ:

5′- TTGCTGTCATTATTCTCAGTGGA-3′ (forward)

5′- GAGGACTCAGGGTGGTTCAG-3′ (reverse)

PIP5K1C:

5′-CGCCACCGACATCTACTTTC-3′ (forward)

5′-ATAGTGGAGCGGGGAGTACA-3′ (reverse)

RUNX2:5′-GGCAGGCACAGTCTTCCC-3′ (forward)

5′-GGCCCAGTTCTGAAGCACC-3′ (reverse)

The qPCR was examined by incubating the cDNA in a reaction mixture containing, 0.5 μM of each primer, and 1-fold concentration of Master reagent (Roche). The amplification conditions initial denaturation at 95°C for 10 min, followed by 45 cycles of 95°C for 10 sec, 55°C for 30 sec, and 72°C for 1 sec. The fluorescent signal was detected at 72°C step of each cycle. The relative quantification of interested gene was normalized by β-actin, and calculated by the value of cross-point (CP) in each fluorescence curve of each gene. For traditional PCR, the reaction mixture containing 2 μl cDNA, 0.2 mM dNTP mixture, 2 μM of each primers, 1 U Taq DNA polymerase, and 1-fold concentration of ThermalPol Buffer (New England BioLabs) was started by denatured 95°C for 5 min, followed by amplification of indicated cycles of 95°C for 30 sec, 55°C for 30 sec, and 72°C for 30 sec. The numbers of cycles for amplification of NSE, EZH2, and β-actin were 35, 35, and 25, respectively. The specific primer sequences for these genes are as follows:

NSE

5'-CATCGACAAGGCTGGCTACACG-3' (forward)

5'-GACAGTTGCAGGCCTTTTCTTC-3' (reverse)

EZH2

5'-CAGTAAAAATGTGTCCTGCAAGAA-3' (forward)

5'-TCAAGGGATTTCCATTTCTCTTTCGA-3' (reverse)

β-actin

5'-GCACTCTTCCAGCCTTCCTTCC-3' (forward)

5'-TCACCTTCACCGTTCCAGTTTTT-3' (reverse)

Smurf2

5'-TCAATTACCTTGGATGACATGGAGTTA-3' (forward)

5'-TCGGGCCTGCAGCACCTTGCAATGCT-3' (reverse)

Gene knockdown by shRNA

Knockdown of genes was performed with the specific shRNAs delivered by the lentiviral system from National RNAi Core Facility (Academia Sinica, Taipei, TW) according to the instruction manual. Briefly, to generate the lentivirus containing specific shRNA, 293T cells were cotransfected with 2.25 μg of pCMV-ΔR8.91 plasmid harboring Gag and Pol genes, 0.25 μg of pMD.G plasmid containing VSV-G gene for expression of envelope glycoprotein, and 2.5 μg of pLKO.1 plasmid bearing the specific shRNA for 16 h; cells were then cultured in growth medium containing 1% BSA for another 24 h. The cultured medium containing lentivirus was collected and stored at –80°C as aliquots for further use. To deliver the specific shRNA construct, approximately 80% confluent cells were infected with the lentivirus bearing specific shRNA in

growth medium containing 8 μg/ml polybrene and incubated at 37°C for 24 h. Afterward, cells were subcultured and selected with 2 μg/ml puromycin. The shRNA constructs targeting the genes of interest were as follows: PPARγ: TRCN0000001671 (#B) and TRCN0000001673 (#D) corresponding to sequences 5′-CTGGCCTCCTTGATGAATAAA-3′ and 5′-CAGCATTTCTACTCCACATTA-3′, respectively; EZH2: TRCN0000040076 (#C) and TRCN0000010475 (#D) corresponding to sequences 5′-CGGAAATCTTAAACCAAGAAT-3′ and 5′-GAAACAGCTGCCTTAGCTTCA-3′, respectively; and Smurf2: EZH2: TRCN0000003478 (#E) and TRCN0000010792 (#F) corresponding to sequences 5′-CCACCCTATGAAAGCTATGAA-3′ and 5′-GCTGGATTTCTCGGTTGTGTT-3′, respectively. The shRNA construct against luciferase (shLuc), TRCN0000072244, referring to sequence 5′-ATCACAGAATCGTCGTATGCA-3′, was used as the negative control.

Chromatin immunoprecipitation (ChIP) and Quantitative ChIP (qChIP) assay

The ChIP assay was performed with use of the EZ ChIP kit (Upstate/Millipore, Billerica, MA, USA). In brief, chromatin and proteins from approximately 2×10^6 cells were crosslinked with 1% formaldehyde for 10 min at room temperature. These cells were collected, lysed, and sonicated on ice to shear the chromatin DNA to a length between 200 and 1000 bp by using the Sonicator 3000 (Misonix, USA) equipped with a microtip (setting: output level 4, 5 times of pulse for 10 sec and pause for 1 min). The sonicated chromatin lysate was immunoprecipitated with specific antibodies against EZH2 (Cell Signaling), H3K27me3 and H3K9me3 (Abcam, Cambridge, MA, USA), and collected with Protein A/G agarose beads (Pierce Biotechnology, Rockford, IL, USA). The protein/DNA crosslinks of the immunoprecipitated complexes were

reversed by incubation in 0.2 M NaCl at 65°C for 4-5 h, and then the DNA was purified and applied to RT-PCR or subjected to qPCR using SYBR Green system according to the manufacturer's instructions (Roche Applied Science, Indianapolis, IN, USA). Data were presented as the fold enrichment of precipitated DNA relative to 1:50 dilution of input chromatin as described above to determine the binding ability of EZH2 to its potential target gene, PPARγ, PIP5K1C and RUNX2. The sequences of the primers specific to the promoter of PPARγ were 5'-TTCATCCTATGGATGGTCCC-3' (forward), and 5'-AGTCCTAGGAGAGATGGCCC-3' (reverse); PIP5K1C were 5'-GACCTACACAGCACATGCCA-3' (forward), and 5'-GCATGTATTGTGCATATCCG-3' (reverse).

Gene Ontology Analysis

We performed Gene Ontology (GO) analysis and observed signals were processed with quantile normalization and probes were collected whose log2 ratios of intensities to control were more than 1. All GO terms in the annotation file were statistically evaluated using hypergeometric mean. Significantly (p value < 0.05) enriched terms classified in GO category Biological Process.

Immunocytochemical analysis

For immunocytochemistry, cells were washed with PBS and fixed for 30 minutes at room temperature in 1% paraformaldehyde. After washing with PBS, the fixed cultured cells were treated for 30 minutes with blocking solution (10 g/L BSA, 0.03% Triton X-100, and 4% serum in PBS). Cells were incubated overnight at 4°C with an antibody against MAP2 (1:200, BD Bioscience) conjugated with FITC. Finally, some of the preparation was lightly counterstained

with DAPI, and then mounted. The preparations were analyzed with a Carl Zeiss LSM510 laser-scanning confocal microscope.

References:

Hung SC, Chen NJ, Hsieh SL, Li H, Ma HL, Lo WH (2002a) Isolation and characterization of size-sieved stem cells from human bone marrow. Stem Cells 20(3)**:** 249-258

Hung SC, Cheng H, Pan CY, Tsai MJ, Kao LS, Ma HL (2002b) In vitro differentiation of size-sieved stem cells into electrically active neural cells. Stem Cells 20(6)**:** 522-529

Hung SC, Yang DM, Chang CF, Lin RJ, Wang JS, Low-Tone Ho L, Yang WK (2004) Immortalization without neoplastic transformation of human mesenchymal stem cells by transduction with HPV16 E6/E7 genes. Int J Cancer 110(3)**:** 313-319

Tsai CC, Chen CL, Liu HC, Lee YT, Wang HW, Hou LT, Hung SC (2010) Overexpression of hTERT increases stem-like properties and decreases spontaneous differentiation in human mesenchymal stem cell lines. J Biomed Sci 17**:** 64

Yu YL, Chou RH, Chen LT, Shyu WC, Hsieh SC, Wu CS, Zeng HJ, Yeh SP, Yang DM, Hung SC, Hung MC (2011) EZH2 regulates neuronal differentiation of mesenchymal stem cells through PIP5K1C-dependent calcium signaling. J Biol Chem 286(11)**:** 9657-9667

Supporting Information Figure S1: Knockdown of EZH2 enhances neuron differentiation of hMSCs. mRNA from different time points (day 0 to day 5) during neuron differentiation were subjected to RT-PCR with the EZH2 and indicated markers. A change in the neuron marker (NSE) marker was observed.

Supporting Information Figure S2. Smurf2 is a potential E3 ubiquitin ligase of EZH2. Immunoblot of the lysate of 293 cells transfected with Myc-EZH2, HA-Ub, along with various E3 ligases for ubiquitination analysis. *Top*, immunoblot; *bottom*, accompanying plot of relative density by scanning densitometric tracing. Error bars represent the SEM from three independent experiments.

Supporting Information Figure S3. Genome-wide mapping of EZH2 target promoters by ChIP-on-chip. Chromatin and proteins within undifferentiated and neuron differentiated 3A6-hMSCs were crosslinked, sheared, and immunoprecipitated with antibody against EZH2. The precipitated genomic DNA fragments were further amplified by LM-PCR and applied to a human promoter microarray (NimbleGen). The Venn diagram depicts the number of significant and overlapping target genes of EZH2 in undifferentiated (undifferentiation) and neuron differentiated (neuron differentiation) 3A6-hMSCs.

Supporting Information Figure S4. PPARγ as a direct target gene of EZH2 during neuron differentiation. ChIP was carried out with use of antibodies against EZH2 (*top*, RT-PCR, and accompanying plot of relative density by scanning densitometric tracings, *bottom*). Error bars represent the SEM from three independent experiments.

Fig S1

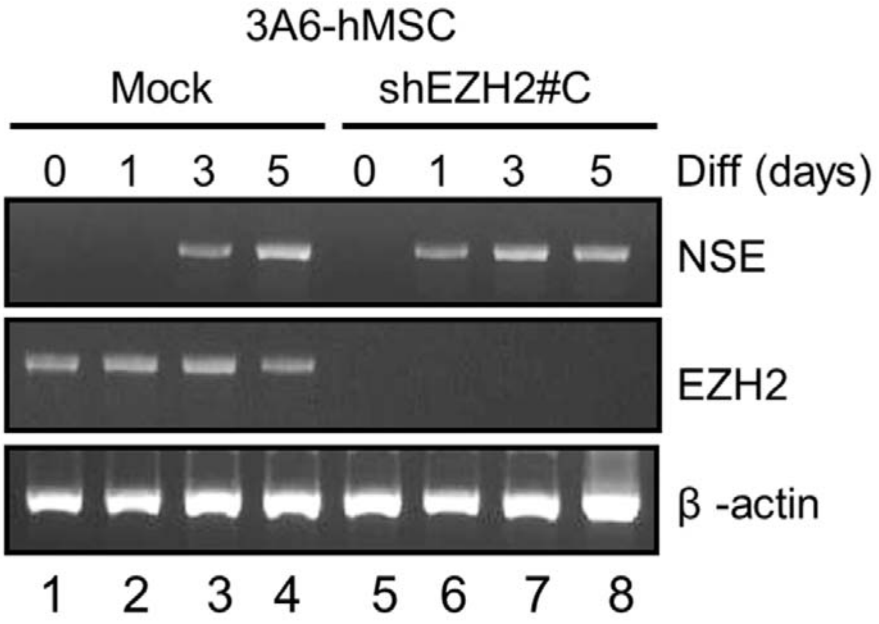
3A6-hMSC
Mock
shEZH2#C
0 1 3 5 0 1 3 5 Diff (days)
NSE
EZH2
β -actin
1 2 3 4 5 6 7 8

Fig S2

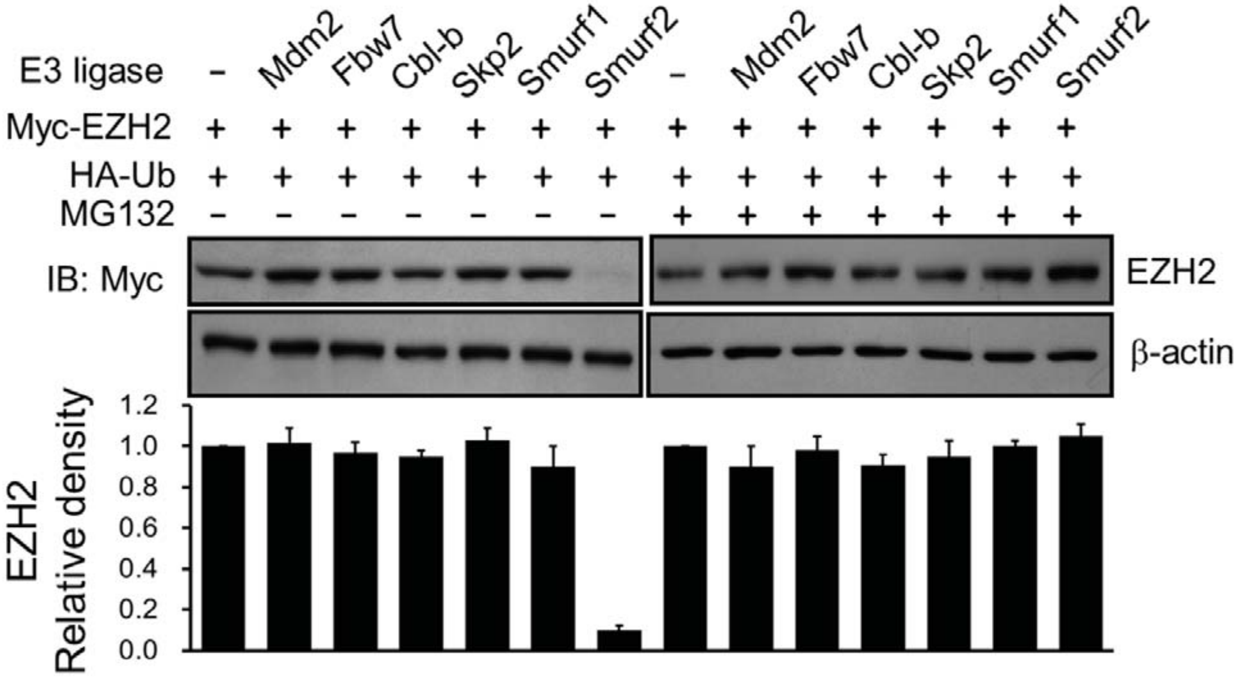

E3 ligase
–
Mdm2
Fbw7
Cbl-b
Skp2
Smurf1
Smurf2
–
Mdm2
Fbw7
Cbl-b
Skp2
Smurf1
Smurf2
Myc-EZH2
HA-Ub
MG132
IB: Myc
EZH2
β-actin
EZH2
Relative density
1.2
1.0
0.8
0.6
0.4
0.2
0.0

Fig S3

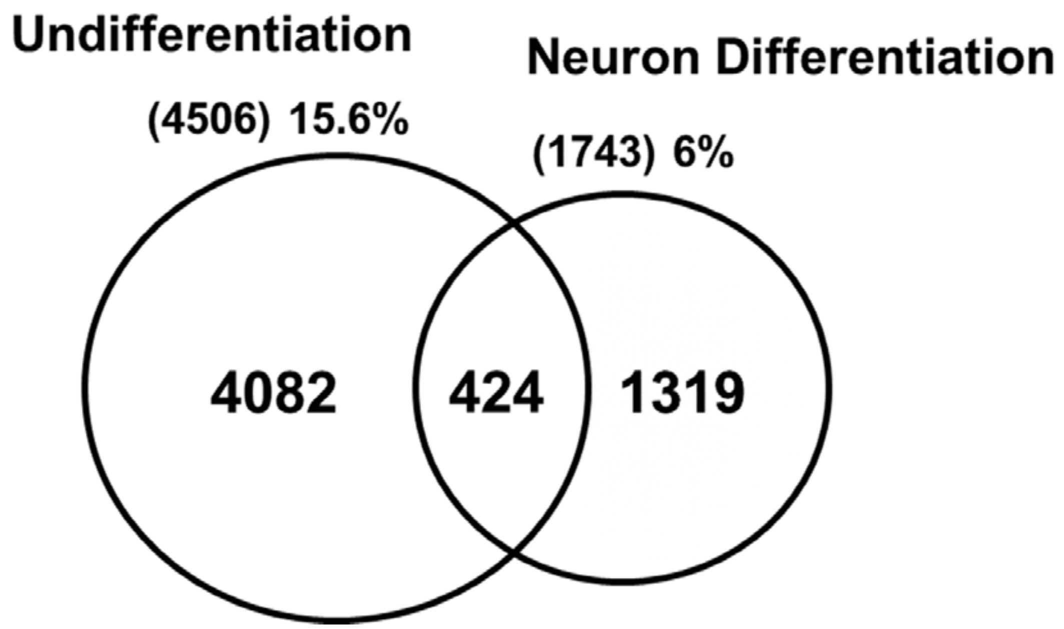
Undifferentiation
(4506) 15.6%
Neuron Differentiation
(1743) 6%
4082
424
1319

Fig S4

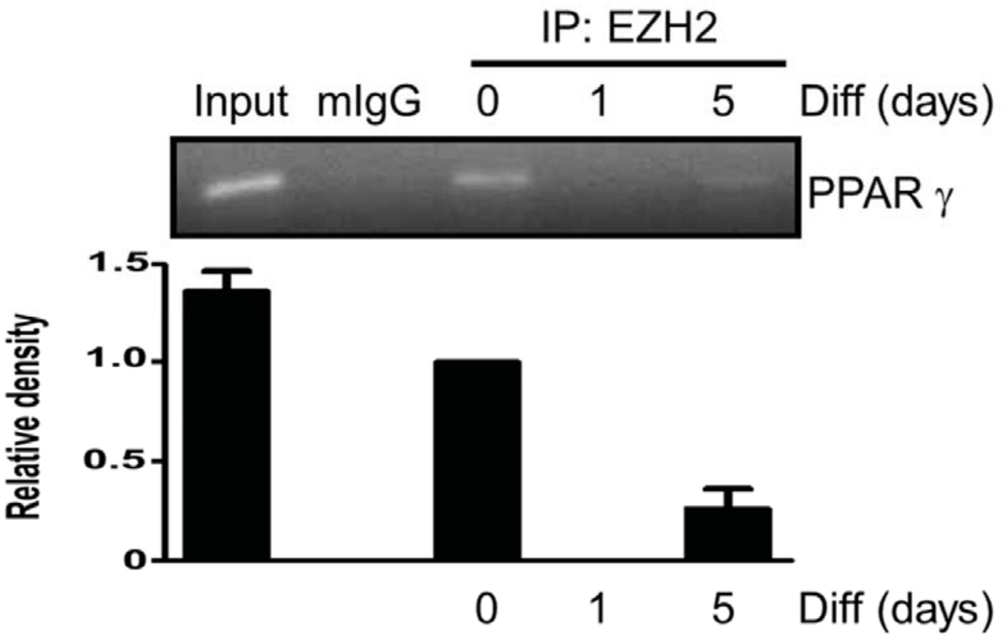
IP: EZH2
Input
mIgG
0
1
5
Diff (days)
PPAR γ
Relative density
1.5
1.0
0.5
0
0
1
5
Diff (days)

Supporting Information Table S1. Heatmap derived from ChIP on chip data of EZH2 target genes in hMSCs neuron differentiation

(Peak Score > 0.2 was considered confidence binding sites)

0.2 3.0

Undifferentiation

PEAK_SCORE	Name	accession
0.92	A2BP1	NM_145893
0.65	A2M	NM_000014
2.19	A2ML1	NM_144670
0.48	A4GALT	NM_017436
0.65	AAA1	AY321516
0.72	AAAS	NM_015665
0.62	AANAT	BC092430
0.8	ABAT	BC008990
1.08	ABC1	NM_022070
0.8	ABCA12	AF418105
0.5	ABCA3	NM_001089
0.62	ABCA5	AK056533
0.54	ABCA7	NM_033308
0.56	ABCA8	NM_007168
0.49	ABCA9	NM_172386
1.07	ABCB7	NM_004299
0.59	ABCB9	NM_203444
0.44	ABCC1	NM_019902
0.98	ABCC11	NM_145186
1.3	ABCC12	BC036378
0.53	ABCC2	NM_000392
0.56	ABCG1	NM_207630
0.72	ABHD11	NM_148916
0.62	ABHD12	NM_015600
0.95	ABHD2	NM_152924
0.83	ABHD9	NM_024794
0.76	ABL2	NM_007314
0.66	ABLIM1	NM_006720
0.92	ACAA2	NM_006111
0.46	ACACA	NM_198839
0.76	ACAD8	NM_014384
0.96	ACADL	NM_001608
1.04	ACAT1	NM_000019
0.62	ACCN1	NM_183377
1.03	ACIN1	NM_014977
0.53	ACO2	NM_001098
0.57	ACOT9	NM_001037171
0.59	ACOX1	NM_007292
0.76	ACRC	NM_052957
0.66	ACSL4	NM_022977
0.77	ACSM1	NM_052956
0.44	ACSM2	NM_182617
0.49	ACSS1	AK125058
0.71	ACSS2	NM_139274
0.47	ACTA2	NM_001613
0.7	ACTG2	NM_001615
0.88	ACTR8	BC032744
0.52	ACYP1	NM_203488
1.08	ADAL	NM_001012969
0.85	ADAM12	NM_021641
0.78	ADAM22	NM_021723
0.57	ADAMTS10	NM_030957
0.92	ADAMTS18	NM_199355
0.78	ADAMTS20	NM_175851
0.59	ADAMTS5	NM_007038
0.6	ADAMTS6	AK131039
0.63	ADARB2	NM_018702
0.74	ADAT1	NM_012091
0.58	ADCY4	AK131333
0.75	ADCY6	NM_020983
0.67	ADH5	NM_000671
0.77	ADH7	X76342
0.92	ADPGK	NM_031284
1.02	ADRA1D	NM_000678
0.87	ADRB2	NM_000024
0.6	ADRBK1	NM_001619
0.48	ADSSL1	NM_152328
0.59	AFG3L1	BC021245
0.68	AFMID	NM_001010982
0.67	AFP	NM_001134
0.88	AGBL2	NM_024783
0.66	AGBL4	NM_032785
0.85	AGPAT1	NM_032741
0.59	AGPAT3	BC040603
0.66	AGPS	NM_003659
1.36	AHCYL1	AK131563
0.55	AHSA1	NM_012111
0.56	AKAP1	NM_139275
0.52	AKAP3	NM_006422
0.77	AKAP5	NM_004857
1.09	AKAP6	NM_004274
0.62	AKAP8	NM_005858
0.78	AKR1B1	NM_001628
1.03	AKR1C3	NM_003739
1.22	AKT2	NM_001626
0.63	AKT3	NM_181690
0.68	ALAD	NM_001003945
0.75	ALCAM	AK127617
1.22	ALDH16A1	NM_153329
0.71	ALDH1A1	NM_000689
0.59	ALDH1L2	XM_927536
1.23	ALDH3A2	NM_001031806
0.63	ALDH3B1	NM_001030010
0.93	ALDH6A1	NM_005589
0.62	ALG10	NM_032834
0.69	ALG11	NM_001004127
1.07	ALG12	NM_024105
1.32	ALG9	NM_024740
0.76	ALKBH8	NM_138775
0.63	ALMS1	NM_015120
0.8	ALOXE3	NM_021628
0.63	AMELX	NM_182681
1.73	AMID	BC006121
0.54	AMOTL1	NM_130847
0.63	AMPD1	NM_000036
0.5	AMPD3	NM_001025389
0.63	AMY2B	BC020861
0.8	ANG	NM_001145
0.79	ANGPTL5	NM_178127
0.53	ANK3	NM_020987
1.14	ANKFN1	NM_153228
0.78	ANKIB1	AK001179
0.62	ANKMY2	NM_020319
0.64	ANKRA2	NM_023039
0.94	ANKRD1	NM_014391
0.83	ANKRD11	NM_013275
0.62	ANKRD19	NM_001010925
0.72	ANKRD22	BC021671
0.92	ANKRD28	NM_015199
0.69	ANUBL1	NM_174890
0.71	ANXA1	NM_000700

Neuron differentiation

PEAK_SCORE	Name	accession
0.57	ABCB1	AF016535
0.56	ABCC9	BC033804
0.35	ABCD1	NM_000033
0.44	ABCF1	NM_001090
0.82	ABHD1	NM_152870
0.42	ABT1	NM_013375
0.39	ACD	NM_022914
0.52	ACF	AJ272078
0.56	ACTN2	NM_001103
0.43	ACTR3B	NM_020445
0.43	ACVR1C	NM_145259
0.61	ACVR2B	NM_001106
0.56	ADAM17	NM_003183
0.56	ADAM30	NM_021794
0.48	ADAMTS13	NM_139028
0.5	ADAMTS3	NM_014243
0.75	ADAMTSL1	NM_139264
0.6	ADAR	NM_015841
0.6	ADCK4	NM_024876
0.47	ADIPOR2	NM_024551
0.64	ADORA3	NM_020683
0.59	AGBL3	NM_178563
0.43	AGR2	NM_006408
0.5	AGTR2	NM_000686
0.56	AGXT2	NM_031900
0.49	AHSA2	NM_152392
0.46	AKNA	NM_030767
0.47	ALK	NM_004304
0.57	ALKBH4	NM_017621
0.41	ALS2CR14	NM_178231
0.41	ALS2CR15	NM_138468
0.54	AMH	NM_000479
0.46	ANAPC2	NM_013366
0.52	ANAPC4	NM_013367
0.63	ANKRD12	NM_015208
0.53	ANKRD13B	AK098421
0.48	ANKRD49	NM_017704
0.48	ANKS1A	NM_015245
0.49	ANKS1B	AF145204
0.49	ANTXR1	NM_053034
0.45	AOAH	NM_001637
0.7	APBB2	NM_173075
0.43	APC2	NM_005883
0.51	APEG1	AK055387
0.46	APTX	NM_017692
0.57	ARG1	NM_000045
0.48	ARHGEF10L	NM_001011722
0.5	ARHGEF11	NM_198236
0.42	ARHGEF3	NM_019555
0.59	ARL4A	NM_212460
0.69	ARL6IP5	NM_006407
0.58	ARL9	NM_206919
0.49	ARMC3	NM_173081
0.55	ARMC8	NM_213654
0.45	ARPC2	U50523
0.48	ARTS-1	BC030775
0.41	ASAP	NM_024826
0.4	ASTE1	NM_014065
0.53	ASXL2	NM_018263
0.55	ATAD2	CR749832
0.45	ATG16L1	NM_198890
0.41	ATOH1	NM_005172
0.44	ATP13A5	NM_198505
0.41	ATP1B1	NM_001677
0.42	ATP2C1	NM_014382
0.43	ATP5F1	NM_001688
0.59	ATP6V1A	NM_001690
0.53	ATP6V1B1	NM_001692
0.46	ATP8A1	NM_006095
0.56	AUP1	NM_181576
0.48	AVPI1	NM_021732
0.44	B3GALT4	NM_003782
0.59	B3GNT1	NM_006876
0.57	BAG2	NM_004282
0.49	BAIAP3	NM_003933
0.68	BAPX1	NM_001189
0.46	BAT1	NM_080598
0.69	BBS4	NM_033028
0.41	BCAN	NM_198427
0.51	BCMP11	NM_176813
0.41	BIN3	NM_018688
0.43	BIRC6	AK023788
0.43	BLZF1	NM_003666
0.54	BMP8A	XM_931937
0.39	BMPER	NM_133468
0.42	BOC	BC034614
0.52	BRCC3	NM_024332
0.59	BRMS1	NM_015399
0.5	BRWD2	AK124522
0.71	BSPRY	AK092607
0.43	BTBD8	NM_183242
0.44	BTBD9	AB067467
0.42	BTLA	NM_181780
0.44	BTN3A2	NM_007047
0.56	BXDC2	NM_018321
0.51	C10orf18	XM_931567
0.48	C10orf59	NM_018363
0.48	C10orf61	NM_015631
0.61	C11orf40	NM_144663
0.67	C12orf30	NM_024953
0.56	C12orf48	NM_017915
0.48	C14orf102	AK024113
0.48	C14orf112	NM_016468
0.55	C14orf168	NM_031427
0.55	C14orf58	NM_017791
0.51	C16orf34	NM_144570
1.01	C1orf102	NM_206837
0.5	C1orf157	NM_182579
0.35	C1orf164	AK001459
0.45	C1orf34	XM_375729
0.48	C1orf50	NM_024097
0.37	C1orf58	NM_144695
0.41	C1orf64	NM_178840
0.43	C1orf74	NM_152485
0.37	C1orf80	NM_022831
0.54	C1orf9	NM_016227
0.49	C1QTNF8	NM_207419
0.45	C20orf151	NM_080833
0.48	C20orf28	NM_015417
0.56	C20orf42	AK092195
0.86	C2orf25	NM_015702

Overlap

PEAK_SCORE	Name	accession
0.54	AASDH	NM_181806
0.48	AASDHPPT	NM_015423
0.5	ABCA10	NM_080282
0.41	ABCA13	AK131360
0.49	ACAD10	AK097425
0.6	ACOT11	NM_147161
0.56	ACSM3	BC002790
0.48	ACTR1A	NM_005736
0.61	ADAM20	AF029899
0.55	ADAM9	NM_003816
0.46	ADAMTS14	NM_139155
0.44	ADK	NM_001123
0.5	ADNP	NM_181442
0.7	ADPRHL1	NM_199162
0.37	AKR1D1	NM_005989
0.69	ALDH1A2	NM_170697
0.46	ALDH1L1	BC045645
0.43	ALS2CR11	NM_152525
0.5	AMMECR1	AK091430
0.49	ANKRD10	NM_017664
0.53	AOC2	NM_009590
0.35	AOX1	NM_001159
0.52	AQP4	NM_004028
0.41	AREG	NM_001657
0.52	ARHGAP6	NM_013423
0.71	ARHGEF10	NM_014629
0.54	ASB17	NM_080868
0.41	ATF3	NM_001030287
0.47	ATF7IP2	NM_024997
0.46	ATG12	NM_004707
0.46	ATP11B	NM_014616
0.48	ATP5E	NM_006886
0.81	ATXN3	S75313
0.51	AVPR1A	NM_000706
0.43	BAZ2B	BX641037
0.42	BCKDHB	NM_183050
0.41	BCL10	BC053617
0.5	BCOR	AY316592
0.63	BTBD10	NM_032320
0.5	C10orf28	AK094479
0.44	C10orf54	NM_022153
0.5	C10orf55	BC049374
0.55	C11orf65	NM_152587
0.68	C11orf67	NM_024684
0.49	C12orf42	NM_198521
0.38	C13orf23	NM_170719
0.48	C14orf119	NM_017924
0.81	C14orf39	NM_174978
0.55	C17orf41	NM_024857
0.52	C18orf16	NM_153010
0.58	C18orf54	BC036054
0.58	C1orf162	NM_174896
0.52	C20orf132	NM_213632
0.52	C20orf141	NM_080739
0.35	C20orf45	NM_016045
0.63	C21orf128	NM_152507
0.46	C21orf34	NM_001005734
0.59	C21orf7	NM_020152
0.47	C22orf13	NM_031444
0.43	C3HC4	NM_022826
0.55	C3orf55	XM_930043
0.34	C6orf168	AK057793
0.59	C6orf195	NM_152554
0.45	C8ORFK23	NM_001039112
0.52	C9orf127	BC098265
0.54	C9orf70	BC007366
0.57	CA5B	NM_007220
0.43	CAB39L	NM_001031724
0.98	CAMLG	NM_001745
0.47	CARD8	BX641091
0.48	CAST	U58996
0.47	CBLN1	NM_004352
0.44	CBR4	NM_032783
0.65	CBX5	NM_012117
0.51	CCDC25	NM_018246
0.57	CCNB1IP1	NM_182852
0.48	CCNL2	NM_030937
0.42	CCR2	BC095540
0.52	CD200R1	NM_170780
0.4	CDC23	NM_004661
0.52	CDC42SE1	NM_020239
0.36	CENPJ	AF217509
0.48	CEP250	NM_007186
0.54	CHODL	NM_024944
0.48	CHST3	NM_004273
0.58	CIAPIN1	CR605182
0.5	CLCN4	NM_001830
0.57	CLDND1	NM_019895
0.49	CLSTN3	BC039075
0.43	CLUL1	NM_014410
0.48	CMTM8	NM_178868
0.72	CNDP2	BC003176
0.42	CNKSR3	AY328894
0.46	CNTN4	NM_175613
0.52	COL4A5	NM_033381
0.54	COL4A6	NM_033641
0.58	COQ9	NM_020312
0.58	CPLX3	NM_001030005
0.46	CREB3L1	NM_052854
0.54	CSF3R	NM_172313
0.41	CSPP1	BX649147
0.44	CTNNA3	BC065819
0.48	CTS9	NM_017418
0.41	CXCL13	NM_006419
0.83	CXorf36	AK094877
0.61	CXorf56	NM_022101
0.61	CXorf6	NM_005491
0.63	DBNDD2	NM_033542
0.46	DCHS2	NM_017639
0.62	DCN	NM_133507
0.76	DCUN1D1	NM_020640
0.48	DDX25	NM_013264
0.5	DDX6	NM_004397
0.69	DERL1	NM_024295
0.53	DGKA	NM_201554
0.5	DKFZP586P0123	AK125894
0.51	DKFZp666G057	NM_001008226
0.44	DKFZp667G2110	BX647079
0.52	DMD	NM_000109
0.59	DNAJC7	NM_003315
0.93	DOCK10	AK001253

0.66	ANXA11	NM_145869
0.56	ANXA2	BC052558
0.83	ANXA4	BC063672
0.63	ANXA9	NM_003568
0.63	AOF2	NM_015013
0.53	AP1G1	NM_001128
0.71	AP2A1	NM_130787
0.88	AP3M1	NM_207012
0.62	AP3M2	NM_006803
0.89	AP4B1	NM_006594
0.8	APEX1	NM_080649
0.91	API5	NM_006595
0.83	APLP1	AK126907
1.04	APOA5	NM_052968
0.56	APOBEC3A	NM_145699
0.45	APOBEC3F	NM_145298
0.63	APOBEC4	NM_203454
1.28	APOC2	NM_000483
1.28	APOC4	NM_001646
1.13	APOL3	NM_145642
0.77	APOL6	NM_030641
0.49	APPBP2	NM_006380
0.86	APRIN	NM_015928
0.49	AQP6	NM_053286
0.71	AQP8	NM_001169
0.82	AR	NM_001011645
0.79	ARD1A	NM_003491
0.82	ARFGEF1	NM_006421
0.74	ARFIP1	NM_014447
0.57	ARHGAP1	NM_004308
0.72	ARHGAP11A	NM_199357
0.76	ARHGAP12	AK001833
0.5	ARHGAP17	BC001241
0.85	ARHGAP18	NM_033515
0.82	ARHGAP20	NM_020809
0.6	ARHGAP22	NM_021226
0.69	ARHGAP28	BC033668
0.82	ARHGAP4	NM_001666
0.68	ARHGAP5	NM_001173
0.85	ARHGAP9	NM_032496
0.55	ARHGDIB	NM_001175
0.76	ARHGEF6	NM_004840
0.54	ARHGEF9	NM_015185
1.11	ARID2	BX648152
0.51	ARID3A	NM_005224
0.56	ARID3B	NM_006465
0.64	ARID4A	NM_023001
0.66	ARID5B	NM_032199
0.82	ARL14	NM_025047
1.26	ARL3	NM_004311
0.71	ARL6IP	NM_015161
0.59	ARL6IP4	NM_018694
0.82	ARMC1	NM_018120
0.56	ARMC5	NM_024742
1.23	ARMCX2	NM_177949
0.56	ARPP-19	NM_006628
0.66	ARR3	NM_004312
0.54	ARRB1	NM_020251
0.54	ARRDC3	NM_020801
0.57	ARSF	BC022389
0.99	ARSG	NM_014960
0.62	ASAH1	NM_177924
0.57	ASAH2	NM_019893
1.03	ASB3	NM_145863
0.72	ASB7	NM_198243
0.57	ASCC1	NM_015947
0.51	ASCC2	AK025241
0.6	ASCL2	NM_005170
0.92	ASF1A	NM_014034
0.72	ASMT	NM_004043
0.68	ASPHD2	NM_020437
0.74	ASPSCR1	NM_024083
0.7	ASTN	NM_207108
0.55	ATF7IP	AK001001
0.75	ATG7	NM_006395
0.82	ATM	NM_000051
0.79	ATP11C	NM_173694
0.51	ATP13A1	AK125861
0.48	ATP4A	NM_000704
1	ATP5G3	NM_001689
0.54	ATP5L	NM_006476
0.98	ATP6AP1	NM_001183
0.65	ATP6V0A1	NM_005177
0.64	ATP6V1D	NM_015994
0.45	ATP6V1E1	NM_001696
0.66	ATP6V1G3	NM_133326
0.69	ATP7B	NM_001005918
0.89	ATP8A2	NM_016529
0.73	ATP8B2	NM_020452
0.65	ATP9A	NM_006045
1.01	ATP9B	NM_198531
0.56	ATPBD4	NM_080650
0.51	ATXN10	NM_013236
0.92	ATXN7	NM_000333
0.99	AURKA	NM_198437
0.62	AURKB	NM_004217
0.62	AURKC	NM_003160
0.69	AVEN	NM_020371
0.65	AVIL	NM_006576
0.63	AVPR1B	NM_000707
0.48	AXL	NM_021913
0.85	B2M	S82297
0.85	B3GAT1	NM_054025
0.86	B3GNT2	BC030579
0.65	B3GNT8	NM_198540
0.59	B4GALNT3	AK131277
0.77	B4GALT5	NM_004776
0.57	BACE1	AL833810
0.56	BACH1	NM_001011545
0.49	BAIAP2	NM_017451
0.83	BARHL2	NM_020063
0.82	BBOX1	BC011034
0.54	BBS1	NM_024649
0.59	BBS2	NM_031885
0.62	BC37295_3	NM_001005850
1.28	BCAM	NM_005581
0.65	BCHE	NM_000055
1.04	BCKDHA	AK127598
0.49	BCL2A1	NM_004049
0.6	BCL2L12	NM_138639
1.95	BCL7C	NM_004765
0.47	BCMO1	NM_017429
0.62	BCR	AK128501
1.01	BDNF	NM_170735
0.58	BET3L	AK002042
0.6	BEX2	NM_032621
0.65	BFSP1	NM_001195
0.66	BHLHB9	NM_030639
0.82	BICD1	NM_001714

0.56	C2orf28	NM_080592
0.41	C2orf7	NM_032319
0.55	C3orf18	NM_016210
0.46	C3orf35	NM_178344
0.36	C3orf40	NM_144635
0.46	C3orf44	NM_152394
0.42	C3orf51	BC074933
0.42	C3orf54	NM_203370
0.42	C3orf57	NM_145035
0.55	C3orf59	NM_178496
0.55	C3orf65	XM_930522
0.5	C3orf9	AK126736
0.44	C4orf15	NM_024511
0.52	C4orf9	NM_003703
1.04	C5orf22	NM_018356
0.44	C6orf103	XM_929679
0.42	C6orf114	NM_033069
0.5	C6orf115	XM_371848
0.42	C6orf139	NM_018132
0.59	C6orf211	NM_024573
0.48	C6orf217	XM_499044
0.46	C6orf47	NM_021184
0.48	C6orf52	BC016820
0.59	C6orf96	NM_017909
0.59	C7orf30	NM_138446
0.75	C7orf38	NM_145111
0.55	C8orf32	NM_018024
0.43	C8orf34	AK094191
0.47	C8orf44	BC014448
0.52	C9orf10OS	NM_198841
0.4	C9orf112	NM_138778
0.46	C9orf119	XM_372143
0.6	C9orf126	NM_173690
0.73	C9orf29	NM_001037807
0.46	C9orf48	BC110502
0.4	C9orf86	DQ099383
0.4	C9orf9	NM_018956
0.4	C9orf98	NM_152572
0.55	CA10	NM_020178
0.61	CACNB2	NM_201570
0.55	CANT1	BC065038
0.53	CAPN10	NM_023089
0.57	CAPN6	BC000730
0.49	CAPZA3	NM_033328
0.45	CARD12	NM_021209
0.51	CART1	NM_006982
0.5	CATSPER3	NM_178019
0.46	CBR3	NM_001236
0.46	CBX2	NM_032647
0.72	CBX6	NM_014292
0.52	CCDC19	NM_012337
0.6	CCDC4	NM_207406
0.53	CCDC63	BC073147
0.56	CCDC98	NM_139076
0.6	CCL20	NM_004591
0.46	CCR4	BC074935
0.46	CCR5	NM_000579
0.42	CCR9	NM_031200
0.41	CCT7	NM_006429
0.58	CD180	NM_005582
0.5	CD1E	NM_030893
0.45	CD302	BC020646
0.41	CD34	NM_001773
0.52	CD53	NM_000560
0.5	CD86	NM_006889
0.51	CD9	NM_001769
0.41	CDA	BC054036
0.49	CDC26	AF503918
0.42	CDC5L	NM_001253
0.41	CDH17	NM_004063
0.59	CDH23	AY563165
0.36	CDIPT	NM_145752
0.6	CDK5RAP2	NM_018249
0.5	CENPF	NM_016343
0.75	CER1	NM_005454
0.54	CFHR5	NM_030787
0.7	CFL2	NM_138638
0.52	CGI-115	NM_016052
0.82	CGREF1	NM_006569
0.48	CH25H	NM_003956
0.6	CHD2	NM_001271
0.59	ChGn	NM_018371
0.56	CHI3L1	NM_001276
0.52	CHIA	BC106910
0.75	CHMP6	NM_024591
0.45	CHN1	NM_001822
0.45	CHPF	NM_024536
0.45	CHRNB2	NM_000748
0.42	CLASP2	AB014527
0.42	CLDN1	NM_021101
0.46	CLDN20	NM_001001346
0.49	CLDN6	NM_021195
0.56	CLGN	NM_004362
0.54	CLIC4	NM_013943
0.4	CLIC5	NM_016929
0.5	CLK4	NM_020666
0.41	CLOCK	BC041878
0.59	CNKSR2	NM_014927
0.59	CNTF	NM_000614
0.52	COG3	NM_031431
0.5	COIL	NM_004645
0.42	COL11A2	AK130938
0.5	COL15A1	NM_001855
0.43	COL16A1	AB209571
0.76	COL24A1	NM_152890
0.43	COL6A3	AB209636
0.43	COPA	NM_004371
0.47	COPE	NM_199444
0.41	COPS5	NM_006837
0.65	COQ3	NM_017421
0.46	CORIN	NM_006587
0.41	COX6C	NM_004374
0.46	CPA3	NM_001870
0.49	CPA5	NM_080385
0.63	CPLX2	NM_006650
0.46	CPNE9	NM_153635
0.5	CRB1	NM_201253
0.5	CREB3	NM_006368
0.44	CRISP3	NM_006061
0.56	CRMP1	NM_001313
0.43	CRP	NM_000567
0.73	CRSP3	NM_015979
0.44	CRSP8	NM_004269
0.48	CRTC2	NM_181715
0.66	CRYZ	NM_001889
0.5	CRYZL1	NM_145858
0.35	CSF2	NM_000758
0.49	CSMD1	NM_033225
0.58	CSNK1A1	NM_001892

0.51	DPYSL2	NM_001386
0.43	EFEMP1	U03877
0.57	EFTUD2	NM_004247
0.56	ELMO3	NM_024712
0.49	EPN1	NM_013333
0.51	ESCO2	NM_001017420
0.5	ET	NM_024311
0.51	ETV1	X87175
0.87	FAF1	NM_131917
0.52	FAM63A	NM_018379
0.56	FAM70B	NM_182614
0.54	FAS	NM_152877
0.46	FBXL11	NM_012308
0.44	FBXO38	BC056147
0.6	FBXO4	NM_033484
0.6	FCMD	BC101808
0.48	FLAD1	NM_201398
0.49	FLJ13391	NM_032181
0.65	FLJ20366	AL713645
0.41	FLJ21062	AK024715
0.48	FLJ25976	NM_174943
0.51	FLJ26175	BX641145
0.6	FLJ37549	NM_152605
0.61	FLJ44715	AK126671
0.58	FMO6	AK130511
0.79	FRYL	XM_932817
0.63	FSHPRH1	NM_006733
0.72	FUT8	NM_178156
0.55	GAB2	NM_080491
0.55	GAS2	NM_005256
0.48	GDF5	BC032495
0.41	GKN1	NM_019617
0.63	GNA12	NM_007353
0.45	GNAL	NM_182978
0.35	GNAS	NM_080425
0.46	GOLPH2	NM_177937
0.55	GOSR1	NM_004871
0.45	GPC6	NM_005708
0.52	H2AFZ	NM_002106
0.7	HBB	NM_000518
0.55	HDAC9	AK097178
0.45	HDGF	NM_004494
0.49	HDGF2	NM_032631
0.48	HELZ	BC094881
0.57	HEXIM2	NM_144608
0.83	HIPK2	AF326592
0.52	HIST1H1D	NM_005320
0.54	HIST1H3D	NM_003530
0.52	HIST1H4F	NM_003540
0.64	HLF	NM_002126
0.65	HNRPA1	NM_031157
0.49	HNRPUL1	NM_144734
0.45	HOXA3	NM_153632
0.48	HOXB9	NM_024017
0.58	HOXC4	NM_014620
0.58	HOXC6	NM_153693
0.46	HSF2	NM_004506
0.62	IGFL2	AK125754
0.36	IGSF11	NM_001015887
0.48	IL18	NM_001562
0.56	IL8	NM_000584
0.73	ING4	NM_198287
0.54	IRX5	NM_005853
0.6	KCNMB4	NM_014505
0.51	KHDRBS3	NM_006558
0.56	KIAA0350	NM_015226
0.54	KIAA0802	BC040542
0.43	KIAA1324	NM_020775
0.45	KIAA1549	AL136736
0.48	KIAA1754	AY189283
0.91	KIAA1815	NM_024896
0.5	KIF13A	AK025303
0.52	KIF21B	XM_930567
0.42	KIF24	XM_929828
0.55	KLHDC1	NM_172193
0.4	KLHDC6	NM_207335
0.5	KLHL4	NM_057162
0.39	LMX1A	NM_177398
0.48	LOC162427	NM_178126
0.56	LOC201725	BC094775
0.49	LOC286016	BC053624
0.54	LOC342897	NM_001001414
0.57	LOC388389	NM_213607
0.46	LOC390110	NM_001031854
0.62	LOC401923	XM_377538
0.87	LOC51336	AF242772
0.45	LOC641705	XM_925793
0.39	LOC642767	XM_926190
0.6	LOC643225	XM_931419
0.41	LOC643514	XM_931594
0.48	LOC643556	XM_933649
0.54	LOC643911	XM_931911
0.5	LOC643936	XM_927194
0.57	LOC644613	XM_927728
0.6	LOC644916	XM_932514
0.48	LOC645281	XM_932783
0.59	LOC645431	XM_934774
0.51	LOC645495	XM_932940
0.81	LOC645563	XM_928579
0.55	LOC645851	XM_928834
0.48	LOC646178	XM_933455
0.61	LOC653084	XM_925895
0.56	LOC653319	XM_933442
0.56	LOC653461	XM_932126
0.33	LOC81558	NM_030802
0.55	LOH11CR2A	NM_198315
0.54	LONRF3	NM_024778
0.41	LRBA	NM_006726
0.58	LRIG3	NM_153377
0.6	LRRK1	XM_932785
0.52	LRRN6C	NM_152570
0.52	LTBP2	NM_000428
0.5	MBNL3	NM_133486
0.61	METTL4	NM_022840
0.48	MFRP	NM_031433
0.49	MGC16824	NM_020314
0.64	MGC24665	BC039361
0.76	MLLT11	NM_006818
0.34	MRCL3	NM_006471
0.48	M-RIP	NM_201274
0.51	MRPS33	BC015462
0.43	MULK	BC022777
0.54	MUM1L1	BC031229
0.56	MYO5B	XM_933749
0.95	N/A	AF090938
0.65	N/A	AF461897
0.54	N/A	AK026318
0.82	N/A	AK026889
0.65	N/A	AK057771

0.45	BIK	NM_001197
0.6	BIRC2	NM_001166
0.54	BIRC3	NM_182962
0.79	BIRC4	NM_001167
0.6	BIRC8	NM_033341
0.62	BLCAP	NM_006698
0.77	BLMH	NM_000386
0.47	BLNK	NM_013314
0.63	BLOC1S2	NM_173809
0.57	BLR1	NM_001716
0.6	BMPR1B	NM_001203
0.63	BMX	NM_001721
0.65	BNC2	CR933649
1.04	BNIP3	NM_004052
0.44	BOLA2B	NM_001039182
0.8	BPIL1	NM_025227
0.98	BPIL2	NM_174932
0.49	BPIL3	NM_174897
0.56	BPY2	NM_004678
0.53	BPY2C	NM_001002761
0.52	BRAP	NM_006768
0.52	BRCA1	NM_007306
0.51	BRD1	NM_014577
0.83	BRD4	NM_058243
0.8	BRD7	NM_013263
0.63	BRDT	NM_001726
0.75	BRF2	NM_018310
0.47	BRSK2	NM_003957
0.49	BRUNOL6	BC033838
0.76	BRWD3	NM_153252
0.66	BSCL2	NM_032667
0.59	BTBD12	NM_032444
0.5	BTBD15	BC050723
0.86	BTBD3	NM_014962
1.26	BTBD7	NM_018167
0.63	BTK	NM_000061
0.56	BUB1B	NM_001211
0.59	BUD31	NM_003910
0.65	BZW2	NM_014038
0.66	C10orf10	NM_007021
0.57	C10orf104	NM_173473
0.53	C10orf116	NM_006829
0.5	C10orf118	NM_018017
1.01	C10orf12	NM_015652
0.91	C10orf120	NM_001010912
0.5	C10orf128	XM_931121
0.72	C10orf13	NM_152429
0.66	C10orf137	AK025270
0.63	C10orf21	XM_374787
0.82	C10orf35	NM_145306
0.53	C10orf39	NM_194303
0.72	C10orf4	NM_203441
0.69	C10orf46	NM_153810
0.72	C10orf53	NM_182554
0.98	C10orf56	NM_153367
0.5	C10orf64	XM_930592
0.5	C10orf68	NM_024688
0.76	C10orf76	NM_024541
0.53	C10orf77	NM_024789
0.6	C10orf78	NM_145247
1.51	C10orf81	BC036365
0.82	C10orf90	NM_001004298
0.7	C10orf97	NM_024948
1.32	C11orf1	NM_022761
0.69	C11orf10	NM_014206
0.5	C11orf11	NM_006133
0.85	C11orf16	NM_020643
0.54	C11orf17	NM_182901
0.82	C11orf30	NM_020193
0.5	C11orf31	NM_170746
0.66	C11orf37	NM_001007543
0.82	C11orf38	NM_212555
0.85	C11orf39	NM_207432
0.5	C11orf41	AK124395
0.63	C11orf52	BC110872
0.69	C11orf53	NM_198498
0.66	C11orf56	NM_032127
0.88	C11orf57	BC030546
0.57	C11orf58	NM_014267
0.69	C11orf59	NM_017907
0.57	C11orf63	NM_199124
0.98	C11orf64	NM_174939
0.88	C11orf71	NM_019021
0.65	C12orf10	NM_021640
0.69	C12orf11	NM_018164
0.55	C12orf23	NM_152261
0.65	C12orf28	NM_182530
0.95	C12orf29	NM_001009894
0.59	C12orf35	AK001514
0.59	C12orf39	NM_030572
1.57	C12orf4	NM_020374
0.65	C12orf41	NM_017822
1.14	C12orf43	NM_022895
0.59	C12orf49	NM_024738
0.59	C12orf52	NM_032848
0.55	C12orf53	NM_153685
0.65	C12orf58	NM_024685
1.08	C12orf59	NM_153022
0.52	C12orf61	NM_175895
0.56	C13orf12	NM_015932
0.79	C13orf18	NM_025113
0.71	C14orf104	NM_018139
0.52	C14orf106	NM_018353
0.77	C14orf111	NM_015962
0.52	C14orf118	NM_017972
2.32	C14orf122	NM_016049
0.55	C14orf124	NM_020195
0.68	C14orf128	BC007251
0.68	C14orf131	AK093342
0.68	C14orf132	BC042922
0.55	C14orf133	NM_022067
0.68	C14orf140	NM_024643
0.52	C14orf143	NM_145231
0.55	C14orf145	AK125925
0.52	C14orf148	NM_138791
0.61	C14orf152	NM_138344
0.55	C14orf161	NM_024764
0.55	C14orf166	NM_016039
0.58	C14orf24	NM_173607
0.64	C14orf28	NM_001017923
0.68	C14orf29	NM_181814
0.61	C14orf37	BC062772
0.93	C14orf46	NM_001024674
0.68	C14orf54	NM_173526
0.87	C14orf93	NM_021944
0.69	C15orf33	NM_152647
0.52	C15orf34	NM_001037223
1.34	C15orf41	BC006254
0.56	C15orf42	NM_152259

0.46	CSNK1G3	NM_004384
0.59	CSTF2	NM_001325
0.61	CTBP2	NM_001329
0.49	CTCF	NM_006565
0.53	CTDSPL2	NM_016396
0.69	CTNNA1	BC031262
0.63	CTSG	NM_001911
0.41	CYB5R1	NM_016243
0.48	CYBASC3	NM_153611
0.37	CYorf15B	NM_032576
0.56	CYP27B1	NM_000785
0.5	DAB2	NM_001343
0.42	DAMS	AF071111
0.52	DAOA	NM_172370
0.4	DBR1	AK000116
0.46	DCBLD1	NM_173674
0.92	DCBLD2	NM_080927
0.49	DDIT3	NM_004083
0.82	DDX3Y	NM_004660
0.47	DDX49	NM_019070
0.52	DEAF1	NM_021008
0.53	DEFA5	NM_021010
0.41	DEFB103A	NM_018661
0.53	DEFB104A	NM_080389
0.39	DENND2D	AK026110
0.96	DEPDC4	NM_152317
0.56	DEXI	NM_014015
0.42	DHFRL1	NM_176815
1.26	DHPS	NM_013407
0.66	DHRS7B	NM_015510
0.48	DIO2	NM_013989
0.41	DIRAS3	NM_004675
0.65	DIRC2	NM_032839
0.41	DISP2	NM_033510
0.48	DKFZp434E1119	XM_495888
0.56	DKFZP586D0919	NM_206914
0.44	DKFZp686D0972	NM_001017992
0.7	DKK2	NM_014421
0.47	DKK4	NM_014420
0.61	DLEC1	NM_007338
0.53	DNAJA4	BC031044
0.5	DNAJB14	NM_001031723
0.43	DNAJB4	NM_007034
0.42	DNASE1L3	NM_004944
0.43	DNTTIP2	NM_014597
0.41	DPP4	NM_001935
0.45	DPY19L4	NM_181787
0.55	DRD3	L20469
0.61	DSCR9	NM_148675
0.53	DUOX2	NM_014080
0.43	DUSP10	NM_144728
0.59	DUSP3	BC008286
0.49	DUSP6	NM_022652
0.48	DYNLT1	NM_006519
0.56	E2F1	NM_005225
0.46	E2F3	NM_001949
0.44	EAF2	NM_018456
0.39	EBF2	AY700779
0.55	ECGF1	NM_001953
0.48	EGFL6	NM_015507
0.47	EHBP1	NM_015252
0.48	EHMT1	AB058779
0.51	EIF2C2	NM_012154
0.48	ELAC2	NM_018127
0.46	ELF2	NM_201999
0.75	ELL2	NM_012081
0.77	Ells1	AL834391
0.54	ELOVL1	NM_022821
0.52	EMD	NM_000117
0.45	EMILIN1	NM_007046
0.42	ENC1	NM_003633
0.48	EPHA10	NM_001004338
0.49	EPHB6	NM_004445
0.45	EPS15	NM_001981
0.52	ESM1	NM_007036
0.5	ESR2	NM_001437
0.41	ESRRG	NM_001438
0.56	ETFDH	NM_004453
0.57	ETV5	NM_004454
0.64	EVI2A	NM_014210
0.6	EXOC8	NM_175876
0.37	EXOSC4	NM_019037
0.48	F12	NM_000505
0.52	F13A1	NM_000129
0.5	F2	NM_000506
0.48	F8	NM_019863
0.48	F8A1	NM_012151
0.48	FAAH	NM_001441
0.57	FABP5	NM_001444
0.5	FAM100B	NM_182565
0.49	FAM107B	NM_031453
0.52	FAM26C	NM_001001412
0.43	FAM40B	NM_020704
0.43	FAM49B	BC016345
0.58	FAM5B	NM_021165
0.41	FAM71A	NM_153606
0.47	FAM83A	NM_207006
0.44	FANCF	NM_022725
0.56	FBN3	NM_032447
0.54	FBS1	AK021680
0.49	FBXL16	BC034014
0.44	FBXO34	NM_017943
0.47	FBXO36	NM_174899
0.5	FCER1G	NM_004106
0.39	FCRL4	NM_031282
0.64	FDXR	NM_024417
0.48	FETUB	NM_014375
0.45	FHAD1	AK096961
0.58	FIS	BC093643
0.45	FKSG2	NM_021631
0.52	FKSG49	AF336887
0.42	FKSG83	NM_032030
0.48	FLJ10986	NM_018291
0.47	FLJ12949	NM_178159
0.43	FLJ13646	NM_024584
0.42	FLJ16323	NM_001004352
0.47	FLJ20758	NM_017952
0.47	FLJ21075	NM_025031
0.41	FLJ21986	NM_024913
0.53	FLJ22374	AK128558
0.4	FLJ23049	NM_024687
0.47	FLJ25143	NM_182500
0.5	FLJ25996	NM_001001699
0.58	FLJ32214	NM_152473
0.52	FLJ32742	AK057304
0.47	FLJ33534	NM_182586
0.4	FLJ33641	NM_152687
0.52	FLJ33718	NM_173660
0.52	FLJ35630	NM_152618

0.56	N/A	AK075438
0.75	N/A	AK095225
0.6	N/A	AK127441
0.75	N/A	AK127740
0.85	N/A	AK128498
1.27	N/A	AK130865
1.04	N/A	BC039352
0.57	N/A	BC071734
0.79	N/A	NM_001039843
0.41	NARG1	NM_057175
0.48	NCOA4	NM_005437
0.63	NCOA7	BC071782
0.48	NDST4	NM_022569
0.41	NDUFC1	BC107682
0.41	NEK7	NM_133494
0.66	NF1	NM_000267
0.59	NKIRAS2	NM_017595
0.65	NKRF	NM_017544
0.42	NPHP3	NM_153240
0.47	NRXN1	BX647616
0.5	NRXN3	AJ316284
0.48	NSF	NM_006178
0.49	NTF3	NM_002527
0.48	OLFML3	NM_020190
0.54	OR10H3	NM_013938
0.51	OR2F1	NM_012369
0.55	OR4C6	NM_001004704
0.44	OR52L1	NM_001005173
0.35	OR5B17	NM_001005489
0.79	OR5D18	NM_001001952
0.5	OR5R1	NM_001004744
0.44	OR6T1	NM_001005187
0.45	OR9A2	NM_001001658
0.6	ORC6L	NM_014321
0.56	OSBPL6	BC021248
0.41	OSR2	NM_053001
0.46	P2RY10	BC095498
0.44	PACRG	NM_152410
0.52	PCGF3	BC107061
0.43	PCM1	NM_006197
0.37	PCYT1B	NM_004845
0.43	PDE4B	NM_002600
0.49	PDIA6	AK127433
0.52	PDXK	BC003651
0.65	PGGT1B	NM_005023
0.62	PGLYRP1	NM_005091
0.74	PHGDHL1	NM_177967
0.48	PIGA	NM_020473
0.42	PKD2L2	NM_014386
0.48	PKNOX1	BC007746
0.45	PLCL1	NM_006226
0.55	POLE2	NM_002692
0.5	POLR1C	BC008863
0.57	PPP1R11	NM_170781
0.48	PPP2R2B	NM_181678
0.4	PRDM4	NM_012406
0.49	PRICKLE1	NM_153026
0.47	PRMT7	NM_019023
0.52	PRND	NM_012409
0.46	PRPF39	AK001990
0.52	PRUNE	NM_021222
0.75	PSME3	NM_176863
0.48	PTEN	NM_000314
0.48	PUS3	NM_031307
0.79	QSER1	NM_024774
0.65	RAB31	NM_006868
0.51	RAB3IP	NM_175627
0.47	RAB4B	NM_016154
0.48	RAI14	NM_015577
0.52	RALGPS2	NM_152663
0.49	RB1CC1	BC017556
0.56	RBBP6	BC051317
0.5	RBM15	AK025596
0.48	RBPSUH	NM_203284
0.48	REEP3	BC018658
0.55	RFPL3S	BC031635
0.5	RNF43	NM_017763
0.45	RNPC3	BC010697
0.49	RP11-114G1.1	BC036697
0.42	RP11-19J3.3	NM_001012267
0.63	RP11-50D16.3	NM_001017370
0.65	RP11-78J21.1	BC108266
0.55	RP5-821D11.2	XM_934902
0.62	RPL7A	BC023624
0.41	RPL9	NM_001024921
0.52	RPN2	BC003560
0.4	RPS18	NM_022551
0.5	RTN3	NM_201430
0.54	S100A11	NM_005620
0.7	SAPS3	BC107599
0.48	SARM1	NM_015077
0.82	SCHIP1	BC005947
0.44	SEC22L2	NM_012430
0.56	SEPTIN2	NM_006155
0.56	SERPINB2	NM_002575
0.52	SERPINH1	NM_001235
0.5	SEZ6	NM_178860
0.52	SFRS6	U30828
0.51	SHC4	NM_203349
0.56	SIGLEC8	NM_014442
0.48	SLA/LP	NM_153825
0.52	SLC17A6	NM_020346
0.48	SLC22A10	XM_933441
0.48	SLC25A24	NM_013386
0.49	SLC38A2	NM_018976
0.46	SLC38A6	NM_153811
0.52	SLC4A11	NM_032034
0.47	SLC7A6OS	NM_032178
0.41	SLC9A7	NM_032591
0.47	SMAD2	NM_005901
0.41	SMC6L1	NM_024624
0.68	SPAG9	NM_003971
0.57	SPECC1	NM_001033555
0.54	SPERT	NM_152719
0.59	SRP46	NM_032102
0.6	ST13	NM_003932
0.59	ST8SIA6	NM_001004470
0.56	STAMBP	BC101469
0.4	STT3B	BC015880
0.48	SUFU	NM_016169
0.48	SYNE1	AF444779
0.79	SYT16	BC040924
0.59	SYTL2	NM_032379
0.36	T2R55	NM_181429
0.45	TBC1D4	NM_014832
0.5	TBC1D9	NM_015130
0.83	TBXAS1	BC014117
0.7	TCF7L2	NM_030756
0.48	TEX12	NM_031275

0.52	C15orf49	BC069077
0.5	C16orf47	NM_207385
0.89	C16orf50	NM_032269
1.01	C16orf52	NM_173501
0.47	C16orf68	NM_024109
0.56	C16orf7	NM_004913
0.59	C16orf70	AK022138
0.49	C17orf27	BC032220
0.68	C17orf32	NM_152464
0.46	C17orf45	NM_152350
0.96	C17orf47	NM_001038704
1.14	C17orf48	NM_020233
0.56	C17orf49	NM_174893
0.99	C17orf68	NM_025099
0.59	C17orf69	NM_152466
0.56	C17orf74	NM_175734
0.62	C17orf80	NM_017941
0.75	C18orf17	NM_153211
0.65	C18orf18	XM_928362
0.75	C18orf19	NM_152352
0.95	C18orf22	NM_024805
0.59	C18orf26	NM_173629
0.85	C18orf37	NM_194281
0.85	C18orf4	NM_032160
1.05	C18orf43	BC007528
1.08	C18orf45	BC054507
0.56	C18orf55	NM_014177
1.83	C18orf8	NM_013326
0.57	C19orf15	NM_021185
0.71	C19orf16	XM_371195
0.86	C19orf18	NM_152474
0.57	C19orf30	NM_174947
0.62	C19orf37	NM_182498
0.71	C19orf4	NM_012109
0.54	C19orf40	NM_152266
0.51	C19orf41	NM_152358
0.7	C1orf121	NM_016076
0.63	C1orf140	NM_001010913
0.83	C1orf149	NM_022756
0.76	C1orf150	NM_145278
0.76	C1orf160	NM_032125
0.7	C1orf177	NM_152607
0.83	C1orf97	NM_032705
0.7	C1QB	NM_000491
0.99	C1QBP	NM_001212
0.95	C1QDC1	NM_032156
0.56	C1QTNF6	AK128125
0.55	C20orf100	AK055135
0.68	C20orf106	NM_001012971
1.2	C20orf11	NM_017896
0.46	C20orf111	NM_016470
0.52	C20orf118	NM_080628
0.95	C20orf12	NM_018152
0.59	C20orf127	NM_080757
0.55	C20orf133	NM_080676
0.55	C20orf165	NM_080608
0.49	C20orf177	NM_022106
0.62	C20orf18	NM_031229
0.52	C20orf185	NM_182658
0.65	C20orf186	NM_182519
0.71	C20orf23	NM_024704
0.62	C20orf26	NM_015585
0.83	C20orf3	NM_020531
0.89	C20orf44	NM_199513
0.55	C20orf50	XM_046437
0.49	C20orf74	XM_933550
0.8	C20orf77	NM_021215
0.77	C20orf79	NM_178483
0.65	C20orf82	AK074473
0.83	C20orf85	NM_178456
0.65	C20orf94	NM_001009608
0.49	C20orf96	NM_153269
0.49	C20orf98	NM_024958
0.8	C21orf100	NM_145033
0.68	C21orf124	NM_032920
0.46	C21orf13	NM_152505
0.62	C21orf24	NM_001001789
0.8	C21orf30	AL117578
0.56	C21orf37	NM_001005521
0.49	C21orf49	NM_001006116
0.71	C21orf58	NM_199071
0.74	C21orf62	NM_019596
0.52	C21orf67	NM_058188
0.74	C21orf88	NM_153754
0.86	C21orf89	AF426268
0.83	C21orf91	NM_017447
0.71	C21orf93	NM_145179
0.51	C22orf18	NM_024053
0.95	C22orf23	NM_032561
0.53	C22orf24	AL050256
0.51	C22orf9	NM_015264
0.83	C2orf13	NM_173545
1.03	C2orf30	NM_015701
0.63	C2orf32	BC035125
0.73	C2orf33	BC000797
0.7	C2orf34	NM_024766
0.99	C3orf14	NM_020685
0.68	C3orf21	AK057046
0.68	C3orf24	NM_173472
0.78	C3orf28	NM_014367
0.92	C3orf52	NM_024616
0.68	C3orf58	NM_173552
0.63	C4BPA	NM_000715
0.64	C4orf17	BC074759
0.84	C4orf18	NM_016613
0.84	C5orf16	NM_173828
0.77	C5orf21	NM_032042
0.81	C5orf5	BC056887
0.78	C6orf130	NM_145063
0.71	C6orf142	BC009010
0.81	C6orf157	NM_198920
1.12	C6orf163	NM_001010868
0.78	C6orf182	BC064365
0.58	C6orf188	NM_153711
0.68	C6orf190	AK128377
0.68	C6orf213	NM_001010852
1.02	C6orf35	NM_018452
1.43	C6orf55	NM_016485
0.92	C6orf61	XM_927492
0.62	C8orf38	NM_152416
0.75	C8orf39	AF116672
0.65	C8orf4	NM_020130
0.65	C8orf53	NM_032334
0.65	C8orf59	XM_934008
0.55	C9orf10	NM_014612
0.91	C9orf150	NM_203403
0.62	C9orf18	NM_198469
0.58	C9orf27	NM_021208
0.87	C9orf30	NM_080655

0.46	FLJ35725	NM_152544
0.53	FLJ35848	NM_001033659
0.39	FLJ36031	NM_175884
0.52	FLJ37228	XM_930209
0.46	FLJ37543	NM_173667
0.41	FLJ40869	NM_182625
0.53	FLJ42289	NM_207383
0.58	FLJ42986	NM_207403
0.45	FLJ43374	NM_198582
0.42	FLJ43763	XM_925858
0.42	FLJ46082	NM_207417
0.47	FLJ46365	NM_207504
0.52	FLNA	NM_001456
0.35	FN1	CR749666
0.42	FNDC6	NM_144717
0.4	FRMD3	NM_174938
0.44	FSTL4	NM_015082
0.48	FTCD	BC032037
0.53	FUT10	NM_032664
0.74	G30	AY138548
0.48	GAB3	NM_080612
0.52	GABBR1	NM_021903
0.62	GABBR2	NM_005458
0.5	GABRA2	NM_000807
0.42	GABRG2	NM_198904
0.44	GABRP	BC074865
0.45	GAL3ST4	NM_024637
0.56	GAS6	NM_000820
0.46	GATA2	NM_032638
0.62	GCA	NM_012198
0.51	GCG	NM_002054
0.42	GCNT2	NM_145655
0.42	GDNF	NM_199234
0.5	GFI1B	BC043371
0.44	GFM1	AK022724
0.54	GGA2	NM_015044
0.54	GJA8	NM_005267
0.43	GJB3	NM_024009
0.43	GJB5	NM_005268
0.56	GLI2	DQ086814
0.44	GLO1	NM_006708
0.44	GLRA3	U93917
0.56	GLRX2	NM_197962
0.43	GNAI3	NM_006496
0.61	GNAT1	NM_144499
0.48	GNAT2	NM_005272
0.58	GNB1	BC005888
0.5	GNB4	NM_021629
0.44	GNPDA1	NM_005471
0.49	GNS	NM_002076
0.37	GPAA1	NM_003801
0.48	GPHN	NM_020806
0.42	GPLD1	NM_177483
0.54	GPR110	NM_153840
0.5	GPR137C	XM_290615
0.44	GPR150	NM_199243
0.41	GPR154	NM_207173
0.42	GPR156	NM_153002
0.63	GPR178	AB037844
0.5	GPR23	NM_005296
0.51	GPR39	NM_001508
0.5	GPR89A	U78723
0.6	GPX3	NM_002084
0.52	GRK4	NM_182982
0.59	GRM1	U31216
0.42	GRM2	NM_000839
0.64	GSN	NM_198252
0.42	GSTA1	NM_145740
0.47	GTDC1	NM_024659
0.42	GZMK	NM_002104
0.48	H2AFB1	NM_001017990
0.48	H2AFB3	BC101415
0.41	H2AFV	NM_201517
0.37	H2AFY2	NM_018649
0.6	H3F3A	NM_002107
0.41	HAND2	NM_021973
0.49	HBM	NM_001003938
0.44	HCG18	XM_933653
0.69	HDDC2	NM_016063
0.51	HDDC3	NM_198527
0.44	HEL308	NM_133636
0.55	HEMK1	NM_016173
0.53	HERC2	AL834183
0.5	HEYL	NM_014571
0.46	HFE	NM_139011
0.42	HIATL1	NM_032558
0.59	HIF1A	NM_181054
0.54	HIST1H2AD	NM_021065
0.65	HIST1H4D	NM_003539
0.54	HIST1H4E	NM_003545
0.44	HLA-DMA	NM_006120
0.44	HLA-DPB2	BC017967
0.46	HLA-DRA	NM_019111
0.4	HMCN2	AK093583
0.54	HMGA1	NM_145905
0.41	HMGB4	NM_145205
0.6	HMGCS2	NM_005518
0.48	HMP19	NM_015980
0.37	HNRPA3	NM_194247
0.54	HNRPAB	NM_031266
0.43	HNRPU	NM_031844
0.53	HOXA2	NM_006735
0.43	HOXA4	NM_002141
0.43	HOXD10	NM_002148
0.42	HRASLS	NM_020386
0.62	HSDL2	NM_032303
0.6	HSF4	NM_001538
0.45	HSPA14	NM_016299
0.65	HSPBAP1	NM_024610
0.47	HSPC268	NM_197964
0.63	HTR3E	NM_182589
0.59	HYAL1	AF502911
0.59	HYAL3	CR621832
0.52	IARS	NM_013417
0.49	IDH1	NM_005896
0.75	IER2	NM_004907
0.53	IFIH1	NM_022168
0.59	IFRD2	Y12395
0.5	IFRG15	NM_022347
0.4	IFT80	NM_020800
0.4	IGFBPL1	NM_001007563
0.7	IGKC	BC070336
0.49	IGKV1-5	BC073792
0.5	IGSF4	NM_014333
1.19	IL11RA	NM_147162
0.63	IL12A	NM_000882
0.48	IL12RB2	NM_001559
0.5	IL19	NM_153758
0.39	IL1F10	NM_173161

0.58	TIGD7	NM_033208
0.55	TM2D2	NM_078473
0.59	TM9SF3	AY358819
0.35	TMED5	BC038511
0.48	TMEM106A	NM_145041
0.48	TMEM138	NM_016464
0.53	TMEM99	NM_145274
0.49	TMTC1	AK055962
0.49	TNPO3	BC068530
0.59	TRIM22	NM_006074
0.59	TRIM31	NM_052816
0.59	TRIM5	NM_033093
0.46	TRMT5	NM_020810
0.48	TTC14	NM_133462
0.53	TTC21B	NM_024753
0.49	TTC9B	NM_152479
0.48	TUBG1	NM_001070
0.41	TWIST2	BC033168
0.76	UBE1	NM_153280
0.49	USP9Y	NM_004654
0.42	UTS2D	NM_198152
0.5	UTX	NM_021140
0.55	VEPH1	NM_024621
0.6	VPS35	BC002414
0.43	VPS37A	BC022363
0.56	VPS37B	NM_024667
0.39	VPS4A	NM_013245
0.44	VPS52	NM_022553
0.48	VTN	NM_000638
0.54	WDFY3	NM_178585
0.46	WDR52	NM_018338
0.65	WDSOF1	NM_015420
0.53	WIBG	NM_032345
0.5	YIPF3	NM_015388
0.59	YY1	NM_003403
0.57	ZBTB20	NM_015642
0.6	ZCCHC11	BX648783
0.61	ZCCHC5	NM_152694
0.35	ZFHX4	NM_024721
0.56	ZIM3	NM_052882
0.59	ZMYND11	DQ335455
0.47	ZNF235	NM_004234
0.5	ZNF445	BC101486
0.47	ZNF720	NM_001004300
0.58	ZNF75A	NM_153028

0.62	C9orf39	BX647069
0.74	C9orf47	NM_001001938
0.68	C9orf66	NM_152569
0.78	C9orf68	NM_017985
0.97	C9orf76	BC064937
0.65	C9orf93	AK096586
1.15	CA12	BC001012
0.47	CA5A	NM_001739
0.83	CA7	NM_005182
0.59	CABP1	NM_031205
0.49	CABYR	NM_153770
0.63	CACHD1	BC039301
0.69	CACNA2D1	NM_000722
1.21	CACNA2D4	NM_172364
0.62	CACNG1	NM_000727
0.48	CACNG2	NM_006078
0.56	CACNG4	NM_014405
0.74	CACNG8	NM_031895
0.76	CACYBP	NM_014412
0.72	CADPS2	NM_017954
0.49	CAMKK1	NM_172206
0.89	CAMTA2	BC016163
0.98	CAND1	NM_018448
0.76	CAPN1	NM_005186
0.62	CAPNS2	NM_032330
0.98	CARD10	NM_014550
0.68	CARD14	NM_052819
0.47	CARD15	NM_022162
0.56	CARHSP1	BC108283
0.49	CASC4	NM_177974
0.76	CASP5	NM_004347
0.65	CAST1	NM_015576
1.01	CBL	NM_005188
0.72	CBLN2	NM_182511
0.65	CBLN4	NM_080617
1.01	CBX7	NM_175709
0.74	CC2D1A	CR608350
0.52	CCBE1	BC046645
0.5	CCDC102A	NM_033212
0.78	CCDC14	NM_022757
0.66	CCDC15	NM_025004
0.5	CCDC34	NM_080654
0.75	CCDC38	NM_182496
0.49	CCDC42	NM_144681
0.8	CCDC45	NM_138363
0.65	CCDC46	NM_001037325
0.59	CCDC53	NM_016053
0.96	CCDC56	BC002698
0.71	CCDC57	NM_152675
1.16	CCDC6	NM_005436
0.54	CCDC67	NM_181645
0.76	CCDC7	AK058148
0.6	CCDC82	NM_024725
0.69	CCDC91	BC028682
0.62	CCDC97	NM_052848
1.8	CCKBR	NM_176875
0.68	CCL14	NM_032963
0.47	CCL17	NM_002987
0.56	CCL3L1	NM_021006
0.56	CCL3L3	BC007783
0.52	CCL5	NM_002985
0.59	CCL7	NM_006273
1.67	CCNB2	NM_004701
1.04	CCNB3	NM_033671
0.64	CCNH	NM_001239
0.49	CCNT1	NM_001240
0.79	CCPG1	BC034914
0.71	CCT8	NM_006585
1.21	CD163L1	NM_174941
0.5	CD19	NM_001770
0.8	CD1A	BC031645
0.63	CD1D	NM_001766
0.62	CD226	BC074787
0.84	CD274	BC074984
0.72	CD276	NM_025240
1.16	CD33	NM_001772
0.82	CD33L3	NM_213602
0.62	CD36	NM_001001548
0.6	CD3D	NM_000732
0.57	CD3EAP	NM_012099
0.6	CD3G	NM_000073
0.6	CD44	NM_001001392
0.73	CD46	NM_172361
0.63	CD59	BC001506
0.46	CD68	NM_001040059
0.5	CD99L2	NM_134446
1.12	CDC16	NM_003903
0.66	CDC2	NM_033379
0.76	CDC20	NM_001255
0.78	CDC2L5	BC001274
0.78	CDC37L1	NM_017913
0.54	CDC42BPG	NM_017525
0.5	CDC42EP2	NM_006779
0.48	CDC42EP5	NM_145057
0.84	CDC42SE2	NM_020240
0.83	CDC6	NM_001254
0.86	CDC73	NM_024529
0.7	CDCA1	NM_145697
0.5	CDCA5	NM_080668
0.59	CDH11	BC013609
0.64	CDH18	NM_004934
0.85	CDH2	NM_001792
0.65	CDH20	NM_031891
0.71	CDH26	NM_021810
0.47	CDH3	NM_001793
0.89	CDH4	AK091496
0.65	CDH5	X59796
0.95	CDH7	NM_033646
0.52	CDK2	NM_052827
0.98	CDK2AP1	NM_004642
1.23	CDK2AP2	NM_005851
0.59	CDK4	NM_000075
0.69	CDK5	NM_004935
0.55	CDKN1B	NM_004064
0.56	CDR2	NM_001802
1.02	CDRT4	NM_173622
0.92	CDX4	NM_005193
0.62	CEACAM1	NM_001712
0.48	CEACAM16	NM_001039213
0.8	CEACAM20	NM_198444
0.68	CEACAM21	NM_033543
0.71	CEACAM7	NM_006890
0.48	CEBPA	NM_004364
1.13	CECR2	AL832377
0.68	CECR5	NM_033070
0.77	CECR6	NM_031890
0.56	CEP192	NM_032142
0.59	CEP27	NM_018097
0.59	CEP290	NM_025114

0.5	IL3	NM_000588
0.69	IMP3	NM_018285
0.52	INA	NM_032727
0.58	INPP4A	NM_004027
0.54	INPP5F	NM_198331
0.55	INSM2	NM_032594
0.44	IQCB1	NM_001023571
0.37	IQSEC2	AB011094
0.62	IRF2	NM_002199
0.46	ISOC1	NM_016048
0.54	ITGA10	NM_003637
0.53	ITGB6	NM_000888
0.47	ITIH5	NM_030569
0.6	ITPKC	NM_025194
0.44	ITPR1	U23850
0.48	JMJD1B	AF251039
0.46	JMJD2C	NM_015061
0.62	JMJD4	NM_023007
0.6	K6IRS3	NM_175068
0.42	KALRN	AK126954
0.48	KBTBD3	NM_198439
0.6	KCNA3	NM_002232
0.42	KCNAB1	NM_172159
0.57	KCNE3	BC113743
0.48	KCNG1	NM_002237
0.41	KCNIP3	NM_013434
0.43	KCNJ13	NM_002242
0.59	KCNMB3	NM_171828
0.53	KCNQ3	NM_004519
0.41	KCNT2	NM_198503
0.43	KEL	NM_000420
0.56	KERA	NM_007035
0.48	KIAA0319L	AK090878
0.48	KIAA0367	NM_015225
0.7	KIAA0460	BC045623
0.47	KIAA0738	NM_014719
0.58	KIAA0748	XM_934154
0.74	KIAA0853	NM_015070
0.48	KIAA0859	NM_015935
0.47	KIAA1217	BX648451
0.5	KIAA1244	NM_020340
0.5	KIAA1344	NM_020784
0.43	KIAA1414	AK001513
0.41	KIAA1458	XM_932792
0.71	KIAA1462	AB040895
0.56	KIAA1468	NM_020854
0.41	KIAA1486	XM_934913
0.51	KIAA1706	NM_030636
0.47	KIAA1833	XM_932057
0.39	KIF5C	AB011103
0.45	KIRREL	NM_018240
0.43	KLF17	NM_173484
0.41	KLF3	NM_016531
0.48	KLHDC8A	NM_018203
0.58	KLHL1	NM_020866
0.73	KPNA1	NM_002264
0.48	KRTAP11-1	NM_175858
0.61	KRTAP20-1	NM_181615
0.61	KRTAP6-1	NM_181602
0.48	KRTAP8-1	NM_175857
0.49	KRTCAP3	NM_173853
0.38	KY	NM_178554
0.72	LAMA3	NM_198129
0.54	LARP1	NM_033551
0.41	LCE4A	NM_178356
0.44	LCN6	NM_198946
0.41	LDLRAP1	NM_015627
0.72	LEREPO4	NM_018471
0.61	LHFPL3	NM_199000
0.65	LIFR	NM_002310
0.7	LIPF	NM_004190
0.35	LIPI	NM_198996
0.48	LIPL1	NM_001010939
0.43	LIPT1	NM_145199
0.42	LMAN2	NM_006816
0.69	LMBRD1	NM_018368
0.36	LOC124216	BC093998
0.48	LOC126661	XM_934315
0.62	LOC126860	XM_929969
0.59	LOC131873	AL713792
0.42	LOC132241	XM_934203
0.49	LOC136306	NM_174959
0.4	LOC138255	NM_001010940
0.6	LOC148696	AL137491
0.41	LOC152586	XM_929404
0.57	LOC155060	NM_001004302
0.39	LOC219854	XM_933919
0.44	LOC280665	AF547222
0.54	LOC283537	NM_181785
0.56	LOC284323	AK091274
0.47	LOC284948	NM_201594
0.45	LOC285016	NM_001002919
0.44	LOC317671	BC024023
0.41	LOC340228	XM_291204
0.45	LOC340281	AK056484
0.39	LOC388284	NM_001012984
0.42	LOC389118	NM_001007540
0.43	LOC389641	XM_374260
0.52	LOC389715	BX648118
0.45	LOC400948	XM_376043
0.48	LOC401286	NM_001023565
0.61	LOC401398	NM_001023566
0.53	LOC405753	NM_207581
0.36	LOC440356	XM_934751
0.55	LOC440836	S72487
0.63	LOC440944	NM_001013713
0.42	LOC440990	XM_934932
0.48	LOC441178	NM_001025489
0.46	LOC441179	NM_001013721
0.45	LOC441204	XM_499061
0.45	LOC441208	NM_001013723
0.53	LOC441548	XM_927923
0.76	LOC51255	NM_016494
0.48	LOC606495	NM_001031672
0.6	LOC63929	NM_022098
0.53	LOC642340	XM_930713
0.49	LOC642411	XM_926162
0.57	LOC642891	XM_931148
0.69	LOC642924	XM_931168
0.46	LOC643273	XM_931453
0.5	LOC643436	XM_931546
0.44	LOC643493	XM_931578
0.49	LOC643645	XM_931710
0.45	LOC643723	XM_931767
0.45	LOC643903	XM_929330
0.41	LOC644075	XM_930109
0.65	LOC644245	XM_932072
0.45	LOC644371	XM_929845
0.47	LOC644629	XM_932271

0.86	CEP350	NM_014810
0.59	CEP76	NM_024899
0.53	CES1	S73751
0.56	CES7	NM_145024
0.71	CFDP1	NM_006324
0.6	CFHR3	NM_021023
0.6	CFHR4	NM_006684
1.04	CFL1	NM_005507
0.57	CGB	BC041054
0.57	CGB5	BC106724
0.57	CGB7	NM_033142
0.62	CGNL1	BC112049
0.53	CHAT	NM_020986
0.65	CHCHD7	NM_024300
1.21	CHD4	NM_001273
0.65	CHD7	NM_017780
0.55	CHD8	AB046784
0.77	CHES1	XM_929939
1.16	CHKB	NM_005198
0.54	CHM	BC073987
0.66	CHML	NM_001821
0.56	CHMP1B	NM_020412
0.58	CHMP4A	NM_014169
0.5	CHORDC1	NM_012124
0.59	CHP	NM_007236
0.98	CHRM2	NM_001006633
0.63	CHRM4	NM_000741
0.85	CHRM5	BC068528
0.76	CHRNA1	NM_001039523
1.75	CHRNE	NM_000080
0.68	CHST5	NM_024533
1.16	CHUK	NM_001278
0.58	CHX10	NM_182894
0.59	CIB1	NM_006384
0.77	CIITA	U18288
1.03	CINP	NM_032630
0.55	CIP29	NM_033082
0.66	CIR	NM_199075
1.33	CIRH1A	NM_032830
0.57	CITED1	NM_004143
0.86	CKAP1	NM_001281
0.59	CKLF	NM_181641
0.98	CKM	NM_001824
0.6	CKMT2	NM_001825
0.86	CLDN10	NM_006984
0.89	CLDN14	NM_012130
0.5	CLDN2	BC014424
0.62	CLDN23	NM_194284
0.72	CLEC12A	AY547296
0.55	CLEC1B	NM_016509
0.59	CLEC2A	NM_207375
0.56	CLEC3A	NM_005752
1.17	CLEC4A	NM_194450
0.51	CLEC4M	NM_214679
1.21	CLEC5A	NM_013252
0.69	CLIC2	NM_001289
0.52	CLLU1OS	NM_001025232
0.55	CLMN	NM_024734
0.89	CLN5	NM_006493
0.63	CLPB	NM_030813
0.62	CLTC	NM_004859
0.61	CMA1	NM_001836
1.06	CMTM5	NM_138460
0.85	CNAP1	NM_014865
0.74	CNGA1	S42457
1.39	CNGA2	NM_005140
0.69	CNGA4	XM_927349
0.68	CNGB1	U58837
1	CNIH	NM_005776
0.65	CNOT2	NM_014515
0.48	CNOT3	NM_014516
0.68	CNP	AK124861
1.04	CNTN1	BC036569
0.6	CNTN5	NM_175566
0.8	CNTROB	NM_053051
0.86	COASY	NM_025233
0.83	COG8	NM_032382
0.58	COL10A1	NM_000493
0.63	COL13A1	NM_080815
0.76	COL17A1	NM_130778
0.77	COL23A1	NM_173465
0.77	COL25A1	NM_198721
1.01	COL2A1	NM_033150
0.6	COL5A2	BC043613
0.74	COL5A3	NM_015719
0.56	COL6A2	NM_058175
0.85	COMMD9	NM_014186
0.54	COMP	NM_000095
0.65	COMT	BC100018
0.69	COPS2	NM_004236
0.63	COPS8	NM_198189
0.53	COQ10B	NM_025147
0.56	CORO2B	NM_006091
0.56	CORO7	NM_024535
0.47	COTL1	NM_021149
0.5	COVA1	NM_182314
0.74	COX11	NM_004375
1.56	COX15	NM_078470
0.55	COX6A1	NM_004373
0.57	COX6B1	NM_001863
0.54	COX7B	NM_001866
0.55	COX8C	NM_182971
0.62	CPAMD8	NM_015692
0.75	CPLX4	NM_181654
1.17	CPNE1	NM_003915
0.74	CPNE2	NM_152727
0.65	CPNE4	AF465771
0.49	CPNE8	NM_153634
0.7	CPS1	NM_001875
0.62	CPSF1	NM_013291
0.69	CPT1A	NM_001876
1.16	CPT1B	NM_152247
0.72	CRADD	NM_003805
0.68	CRB3	NM_174881
0.85	CREB3L2	NM_194071
0.64	CRHBP	NM_001882
0.56	CRI1	NM_014335
0.63	CRIPT	NM_014171
0.71	CRLF1	NM_004750
0.62	CRNKL1	AL832435
0.59	CROP	AF069250
0.88	CROT	NM_021151
0.6	CRSP2	NM_004229
0.76	CRTAC1	AJ421515
0.54	CRTC1	NM_025021
0.52	CRTC3	NM_022769
0.82	CRY2	NM_021117
0.49	CSAD	NM_015989
0.74	CSN2	X55739

0.42	LOC644891	XM_927976
0.44	LOC645187	XM_932714
0.48	LOC645335	XM_928372
0.48	LOC645342	XM_928376
0.56	LOC645396	XM_932872
0.41	LOC645676	XM_935153
0.44	LOC645793	XM_371850
0.5	LOC646051	XM_933368
0.49	LOC646240	XM_930540
0.35	LOC646345	XM_933543
0.41	LOC646484	XM_933646
0.44	LOC646536	XM_929465
0.41	LOC646626	XM_933781
0.43	LOC646817	XM_929774
0.39	LOC646854	XM_933973
0.52	LOC646894	XM_929857
0.51	LOC646977	XM_934116
0.39	LOC647079	XM_930090
0.43	LOC647089	XM_934342
0.43	LOC647090	XM_930104
0.43	LOC647243	XM_934564
0.5	LOC647251	XM_934577
0.41	LOC647326	XM_930394
0.46	LOC653033	XM_930589
0.83	LOC653052	XM_925800
0.48	LOC653463	XM_376558
0.46	LOC653482	XM_496836
0.37	LOC653516	XM_927858
0.46	LOC653725	XM_929216
0.56	LOC653752	XM_933660
0.43	LOC653754	XM_929449
0.64	LOC654434	AF130050
0.43	LOC664727	AK093659
0.5	LRCH2	NM_020871
0.44	LRP11	NM_032832
0.37	LRRC4C	NM_020929
0.41	LRRC51	NM_145309
0.48	LRRC8B	NM_015350
0.41	LRRFIP1	BC010662
0.45	LRRN3	NM_018334
0.58	LRRN5	NM_201630
0.53	LRRN6A	NM_032808
0.69	LRRTM2	NM_015564
0.52	LSG1	NM_018385
0.58	LUZP1	BC033219
0.45	LYPLA2	NM_007260
0.45	LYPLAL1	NM_138794
0.6	LYSMD1	NM_212551
0.53	M6PR	NM_002355
0.59	MAK3	NM_025146
0.54	MAML1	NM_014757
0.42	MAN2A1	BC043416
0.45	MAP3K8	NM_005204
0.49	MARCO	NM_006770
0.45	MATN2	NM_030583
0.45	MBD5	NM_018328
0.49	MBD6	AK093078
0.53	MCM10	NM_182751
0.42	MCM3	NM_002388
0.43	MCOLN2	NM_153259
0.44	MED12L	NM_053002
0.5	MEG3	CR615599
0.5	MEGF6	XM_927400
0.64	MFAP1	U04209
0.46	MGC10981	BC004397
0.64	MGC41945	NM_203299
0.47	MGC5509	NM_024093
0.39	MGC87042	BC066301
0.45	MGC99813	NM_001005209
0.45	MGST3	NM_004528
0.53	MLC1	NM_139202
0.69	MLF2	NM_005439
0.56	MLLT3	NM_004529
0.46	MLR1	AK055258
0.45	MMACHC	NM_015506
0.52	MME	X07166
0.56	MMP15	NM_002428
0.51	MON1B	AK128411
0.48	MORF4	NM_006792
0.48	MOSC2	NM_017898
0.52	MOSPD2	NM_152581
0.62	MOV10	NM_020963
0.53	MOV10L1	NM_018995
0.66	MPZL1	NM_024569
0.57	MRPL13	NM_014078
0.48	MRPL37	NM_016491
0.66	MRPL50	NM_019051
0.53	MSC	NM_005098
0.6	MSH3	NM_002439
0.43	MSRA	NM_012331
0.42	MSX2	NM_002449
0.57	MTBP	NM_022045
0.43	MTHFD2	NM_006636
0.43	MTPN	NM_145808
0.65	MTUS1	NM_001001931
0.42	MUT	NM_000255
0.52	MX2	NM_002463
0.43	MYBPH	NM_004997
0.5	MYBPHL	NM_001010985
0.48	MYEOV	NM_138768
0.43	MYOC	NM_000261
0.42	MYOZ3	NM_133371
0.43	MYST1	NM_032188
0.63	N/A	AB064667
0.42	N/A	AB082531
0.61	N/A	AB096956
0.6	N/A	AB126828
0.6	N/A	AB126829
0.5	N/A	AF090938
0.4	N/A	AF116706
0.54	N/A	AF130056
0.45	N/A	AF130061
0.62	N/A	AF130099
0.62	N/A	AF289572
0.55	N/A	AF318333
0.42	N/A	AF351612
0.56	N/A	AF461897
0.59	N/A	AK023045
0.55	N/A	AK026318
0.43	N/A	AK026836
0.79	N/A	AK026889
0.41	N/A	AK027319
0.45	N/A	AK056230
0.45	N/A	AK057771
0.58	N/A	AK075438
0.71	N/A	AK090511
0.56	N/A	AK092303
0.4	N/A	AK093333
0.49	N/A	AK094373

0.69	CSNK1A1L	NM_145203
0.69	CSNK2A1P	AY112721
0.54	CSPG3	NM_004386
0.47	CSPG4LYP1	AF332228
0.95	CSPG5	NM_006574
0.62	CSRP2BP	NM_020536
0.57	CSRP3	BC024010
0.71	CSTB	NM_000100
0.52	CSTF1	NM_001324
1.48	CSTL1	NM_138283
0.62	CTA-126B4.3	NM_015703
0.45	CTA-216E10.6	BC007210
0.56	CTA-250D10.11	NM_152613
0.5	CTAG1A	NM_139250
0.5	CTAG1B	NM_001327
0.64	CTAGE5	NM_203357
0.72	CTAGE6	XM_498461
1.04	CTB-1048E9.5	NM_001013694
0.79	CTDP1	NM_048368
1.95	CTF1	NM_001330
0.75	CTGF	NM_001901
1.04	CTNNAL1	NM_003798
1.36	CTPS2	NM_175859
0.8	CTSS	NM_004079
0.62	CUL1	NM_003592
0.66	CUL3	NM_003590
1.54	CUTC	NM_015960
0.46	CXCL16	NM_022059
0.73	CXorf1	NM_004709
0.54	CXorf2	BC026183
0.73	CXorf20	NM_153346
0.54	CXorf22	NM_152632
0.63	CXorf23	NM_198279
1.01	CXorf25	AK094108
0.6	CXorf33	NM_198450
0.57	CXorf42	NM_173522
0.73	CXorf43	NM_144657
0.63	CXorf44	NM_138362
0.6	CXorf48	NM_017863
0.5	CXorf50	BC041962
0.54	CXorf55	NM_152425
0.5	CXX1	NM_003928
0.62	CYB5D2	NM_144611
0.48	CYB5R3	AK124528
0.6	CYBB	NM_000397
0.71	CYLD	NM_015247
1.34	CYP19A1	M28420
0.51	CYP2A13	NM_000766
0.48	CYP2A6	NM_000762
0.57	CYP2B7P1	BC041174
0.63	CYP2C18	NM_000772
0.48	CYP4F2	NM_001082
0.58	DAAM1	NM_014992
0.6	DACH2	NM_053281
0.69	DAK	NM_015533
0.72	DAPK2	NM_014326
0.58	DAZ2	U21663
0.58	DAZ3	NM_020364
0.5	DAZ4	NM_020420
1.11	DAZAP2	NM_014764
0.73	DBX1	NM_001029865
0.55	DCAL1	NM_172004
0.66	DCAMKL1	NM_004734
0.47	DCI	NM_001919
1.16	DCLRE1A	NM_014881
0.83	DCUN1D3	NM_173475
0.85	DCX	NM_178153
0.52	DDHD1	NM_030637
1.04	DDN	NM_015086
0.63	DDX18	AK091227
0.65	DDX27	AK000603
0.82	DDX47	NM_201224
0.65	DDX5	NM_004396
0.89	DDX52	NM_152300
0.69	DDX53	NM_182699
0.59	DDX54	NM_024072
0.52	DDX55	NM_020936
0.5	DDX59	NM_031306
0.99	DEFB123	NM_153324
0.65	DEFB126	NM_030931
0.59	DEFB128	NM_001037732
0.63	DENND1B	NM_144977
1.11	DENND4A	NM_005848
0.63	DEPDC1	NM_017779
1.08	DEPDC2	NM_025170
0.51	DEPDC5	NM_014662
0.55	DERA	NM_015954
0.52	DERL2	NM_016041
1.33	DERPC	NM_017804
0.85	DFNA5	NM_004403
0.66	DGAT2L3	NM_001013579
0.48	DGCR6	NM_005675
0.77	DGKE	NM_003647
0.66	DGKK	NM_001013742
0.74	DHRS4	NM_021004
0.68	DHRS4L2	NM_198083
0.63	DHTKD1	NM_018706
0.65	DHX16	NM_003587
0.5	DHX29	NM_019030
0.53	DHX32	NM_018180
0.62	DHX33	NM_020162
0.68	DHX35	AK025541
0.52	DHX40	NM_024612
0.74	DHX8	NM_004941
1.46	DHX9	NM_001357
0.65	DIABLO	NM_138930
0.63	DIAPH2	NM_007309
0.91	DIP2B	AB040896
0.92	DJ122O8.2	NM_020466
0.92	dJ341D10.1	NM_001007535
0.63	DKC1	NM_001363
0.79	DKFZp313A2432	XM_934482
0.95	DKFZp451A211	NM_001003399
0.66	DKFZp451M2119	NM_182585
0.5	DKFZp547E087	BC094882
0.66	DKFZP564B147	BC071991
0.92	DKFZp686I15217	NM_207495
0.6	DKFZp686I1569	NM_001024596
1.14	DKFZp762I137	NM_152411
0.66	DKFZp779M0652	XM_374877
1.04	DKFZp779O175	XM_932926
0.57	DKKL1	NM_014419
0.79	DLG2	CR933674
0.63	DLG5	NM_004747
0.71	DLGAP4	NM_183006
0.58	DLST	NM_001933
0.52	DLX3	NM_005220
0.48	DMC1	NM_007068
0.87	DMRTA1	NM_022160

0.41	N/A	AK094604
0.57	N/A	AK094853
0.44	N/A	AK095061
0.52	N/A	AK095225
0.48	N/A	AK097099
0.48	N/A	AK122760
0.41	N/A	AK123599
0.47	N/A	AK123697
0.45	N/A	AK124216
0.49	N/A	AK124265
0.57	N/A	AK124397
0.68	N/A	AK124509
0.39	N/A	AK124606
0.47	N/A	AK124960
0.51	N/A	AK124965
0.57	N/A	AK125068
0.53	N/A	AK125229
0.39	N/A	AK125579
0.52	N/A	AK125630
0.52	N/A	AK126107
0.42	N/A	AK126504
0.41	N/A	AK126559
0.36	N/A	AK126656
0.67	N/A	AK126960
0.41	N/A	AK127105
0.41	N/A	AK127108
0.48	N/A	AK127441
1.08	N/A	AK127462
0.49	N/A	AK127488
0.5	N/A	AK127740
0.54	N/A	AK127880
0.39	N/A	AK127999
0.39	N/A	AK128036
0.58	N/A	AK128197
0.41	N/A	AK128227
0.39	N/A	AK128355
0.43	N/A	AK128498
0.5	N/A	AK128703
0.52	N/A	AK129721
0.49	N/A	AK129966
0.41	N/A	AK130267
0.69	N/A	AK130286
0.61	N/A	AK130290
0.34	N/A	AK130540
0.42	N/A	AK130729
0.39	N/A	AK130865
0.49	N/A	AK130913
0.59	N/A	AK131288
0.52	N/A	AK131317
0.49	N/A	AK131319
0.56	N/A	AK131328
0.48	N/A	AK131430
0.57	N/A	AK172748
0.76	N/A	AL359596
0.51	N/A	AX773941
0.51	N/A	AX773943
0.41	N/A	AY203950
0.51	N/A	AY203962
0.45	N/A	AY358263
0.53	N/A	AY382899
0.42	N/A	AY831680
0.46	N/A	BC011600
0.61	N/A	BC027486
0.49	N/A	BC030813
0.54	N/A	BC031525
0.49	N/A	BC039352
0.48	N/A	BC042900
0.5	N/A	BC047021
0.58	N/A	BC051366
0.42	N/A	BC068082
0.42	N/A	BC069735
0.68	N/A	BC071734
0.43	N/A	BC075797
0.54	N/A	BC098418
0.49	N/A	BC110303
0.49	N/A	BX538248
0.39	N/A	BX649074
0.67	N/A	CR591960
0.75	N/A	CR601641
0.39	N/A	CR602867
0.65	N/A	CR605673
0.5	N/A	CR608653
0.5	N/A	CR611169
0.47	N/A	CR749477
0.6	N/A	NM_001039843
0.36	N/A	NM_152340
0.41	NAG	NM_015909
0.57	NALP1	BC016965
0.53	NAT1	NM_000662
0.59	NAT6	NM_012191
0.56	NCOA5	NM_020967
0.43	NCSTN	NM_015331
0.48	NDST2	BC110588
0.41	NDUFA4	NM_002489
0.51	NDUFA6	NM_002490
0.4	NEK11	NM_145910
0.37	NEURL	NM_004210
0.47	NEUROD6	NM_022728
0.48	NFKBIE	U91616
0.46	NFKBIL1	NM_005007
0.58	NFYC	AF191744
0.46	NGLY1	NM_018297
0.62	NHLH1	NM_005598
0.51	NKPD1	NM_198478
0.58	NKX2-2	NM_002509
0.51	NLGN4Y	NM_014893
0.57	NLK	NM_016231
0.43	NME7	NM_197972
0.56	NOL4	NM_003787
0.36	NOL8	NM_017948
0.47	NPBWR1	NM_005285
0.45	NPHP4	AL117405
0.56	NPNT	NM_001033047
0.42	NRN1	NM_016588
0.42	NSUN3	NM_022072
0.37	NT5C	NM_014595
0.4	NTNG2	NM_032536
0.41	NUAK2	NM_030952
0.56	NUP37	NM_024057
0.42	OGG1	NM_016829
0.39	OLFML2B	NM_015441
0.49	OR10A7	NM_001005280
0.5	OR11A1	NM_013937
0.38	OR2A4	NM_030908
0.47	OR2A7	NM_001005328
0.66	OR2S2	NM_019897
0.5	OR2T8	NM_001005522
0.41	OR2W5	NM_001004698
0.55	OR4C11	NM_001004700

0.57	DMRTC1	NM_033053
1.25	DMWD	BC019266
0.85	DMXL2	NM_015263
0.8	DNAH3	AK056509
0.71	DNAH8	NM_001371
0.62	DNAH9	NM_001372
0.52	DNAHL1	NM_173628
0.72	DNAJB12	NM_017626
1.13	DNAJB7	NM_145174
0.73	DNAJC1	NM_022365
0.7	DNAJC10	NM_018981
0.76	DNAJC11	AL109978
0.82	DNAJC14	NM_032364
0.65	DNAJC17	NM_018163
1.2	DNAJC5	NM_025219
0.89	DNALI1	NM_003462
0.95	DNASE1	NM_005223
0.73	DNHD1	NM_144666
0.6	DNM2	AK097875
0.93	DNMT2	NM_176086
0.69	DOCK11	NM_144658
0.75	DOCK4	NM_014705
0.68	DOCK5	AK126249
0.8	DOCK6	BC008335
0.92	DOCK9	AK127329
0.99	DOK5	NM_177959
0.65	DOM3Z	NM_005510
1.26	DPYD	NM_000110
0.63	DPYSL5	NM_020134
0.72	DRCTNNB1A	NM_032581
0.5	DRD2	AF176812
0.86	DRG1	NM_004147
0.75	DSC3	NM_024423
0.8	DSCR1L2	NM_013441
0.56	DSG4	NM_177986
0.71	DST	NM_020388
0.55	DSTN	NM_006870
0.63	DTL	NM_016448
1.5	DTWD1	NM_020234
0.72	DTX2	BC064535
0.85	DTX3	NM_178502
0.83	DTYMK	NM_012145
0.5	DUPD1	NM_001003892
0.63	DUSP11	NM_003584
0.53	DUSP13	NM_016364
0.65	DUSP18	NM_152511
0.83	DUSP19	NM_080876
0.72	DUT	NM_001948
0.65	DUXA	NM_001012729
0.52	DYM	NM_017653
0.8	DYNC1I2	NM_001378
0.47	DYNC2H1	BX538093
0.77	DYNLL2	NM_080677
0.86	DYNLRB2	NM_130897
0.83	DYRK3	NM_003582
0.72	EBI2	NM_004951
0.56	EBPL	NM_032565
0.62	ECM2	NM_001393
0.82	EDA	NM_001399
0.69	EDA2R	NM_021783
1.29	EDEM1	NM_014674
0.6	EDEM3	BC016464
0.78	EDG2	BC073167
0.74	EDG3	NM_005226
0.67	EDIL3	NM_005711
1.02	EDNRB	NM_003991
0.66	EED	NM_152991
0.65	EEF2	NM_001961
0.53	EFHA1	NM_152726
0.57	EFHC2	CR749363
0.68	EFS	NM_032459
0.71	EGFL11	NM_198283
0.82	EGFR	NM_201284
0.92	EHD2	NM_014601
0.82	EHF	NM_012153
1.14	EID3	NM_001008394
0.48	EID-3	NM_152361
0.69	EIF1AY	NM_004681
0.56	EIF2AK1	NM_014413
0.63	EIF2AK2	NM_002759
0.64	EIF2S1	NM_004094
0.62	EIF2S2	BC007888
0.59	EIF3S1	NM_003758
0.53	EIF3S10	NM_003750
1.16	EIF3S12	NM_013234
0.77	EIF3S6IP	AK098457
0.54	EIF4G2	AB063323
0.7	EIF5B	NM_015904
0.89	ELAVL1	NM_001419
0.68	ELAVL2	NM_004432
1.36	ELF5	NM_198381
0.49	ELMO2	NM_182764
0.68	ELOVL2	NM_017770
0.5	ELOVL3	NM_152310
0.49	EME1	NM_152463
0.8	EMID1	NM_133455
0.52	EMILIN2	NM_032048
0.5	EMP2	NM_001424
0.6	EMX2	NM_004098
0.86	ENAH	NM_018212
0.76	ENO1P	BC046928
1.34	ENTPD4	BC034477
0.85	ENTPD7	NM_020354
0.55	EPB41L1	NM_012156
0.71	EPB41L2	AY512660
0.88	EPB41L3	BC008377
0.91	EPB41L4A	NM_022140
0.75	EPHA7	NM_004440
0.68	EPM2A	NM_005670
0.8	EPN2	NM_148921
0.52	EPN3	NM_017957
0.74	ERAF	NM_016633
0.63	ERCC6	NM_000124
1.01	ERN2	NM_033266
0.6	ESAM	NM_138961
0.59	ESCO1	BC089426
0.49	ESD	NM_001984
0.88	ESPL1	NM_012291
0.64	ESRRB	AY451390
0.95	ETFB	AK128845
0.94	EVC	NM_153717
0.94	EVC2	AY185210
0.84	EVL	BC023997
0.59	EXDL1	NM_152596
0.53	EXOC6	AK002113
1.01	EXOSC1	NM_016046
0.57	EXT2	U72263
1.03	EXTL2	NM_001439
0.59	EYA1	NM_172060

0.6	OR4F15	NM_001001674
0.66	OR4S2	NM_001004059
0.48	OR52H1	NM_001005289
0.68	OR52M1	NM_001004137
0.48	OR5AN1	NM_001004729
0.55	OR5B21	NM_001005218
0.46	OR5H1	NM_001005338
0.79	OR5T3	NM_001004747
0.63	OR5V1	NM_030876
0.65	OR6C74	NM_001005490
0.57	OR8A1	NM_001005194
0.5	OR8D4	NM_001005197
0.44	OR8G1	NM_001002905
0.55	OR8J1	NM_001005205
0.41	ORMDL1	BC005200
0.46	OXSM	NM_017897
0.44	PACSIN1	BC040228
0.43	PAFAH2	NM_000437
0.48	PAK1IP1	NM_017906
0.41	PALLD	AB023209
0.41	PALMD	NM_017734
0.44	PARK2	NM_013988
0.61	PARP2	NM_005484
0.49	PAX8	NM_013992
0.47	PAXIP1	BC033781
0.46	PCBP4	NM_033010
0.48	PCDHA10	NM_031860
0.48	PCDHB1	NM_013340
0.38	PCDHGA4	NM_032053
0.38	PCDHGB1	NM_032095
0.42	PCDHGC5	NM_032407
0.47	PCLO	BX649041
0.43	PCMTD1	AF293366
0.52	PCOTH	NM_001014442
0.48	PCSK1N	NM_013271
0.73	PDCD2L	NM_032346
0.5	PDCD6IP	NM_013374
0.42	PDE10A	BC104858
0.41	PDE11A	NM_016953
0.62	PDE3A	NM_000921
0.44	PDE5A	NM_033437
0.41	PDGFC	NM_016205
0.44	PDGFRA	NM_006206
0.44	PDHA2	NM_005390
0.44	PECI	NM_206836
0.52	PET112L	NM_004564
0.45	PFDN2	NM_012394
0.61	PHF1	NM_024165
0.46	PHF10	NM_133325
0.44	PHF12	NM_020889
0.44	PHF17	NM_024900
0.59	PHF20L1	BC050655
0.51	PHKG2	NM_000294
0.46	PIGO	NM_152850
0.62	PIGR	NM_002644
0.48	PIGV	NM_017837
0.38	PIK3R1	NM_181523
0.42	PIM1	NM_002648
0.47	PIP5K3	NM_152671
0.48	PJA2	NM_014819
0.41	PKD2	NM_000297
0.5	PLCXD3	NM_001005473
0.49	PLCZ1	NM_033123
0.55	PLD1	BC068976
0.55	PLDN	NM_012388
0.52	PLEKHB1	NM_021200
0.34	PLSCR2	NM_020359
0.54	PLXNA2	BC094835
0.41	PMS1	AB102869
0.45	PNMA2	NM_007257
0.5	PODN	NM_153703
0.62	POLR3K	NM_016310
0.44	POMT1	NM_007171
0.41	PON1	NM_000446
0.45	PPIH	NM_006347
0.43	PPOX	NM_000309
0.41	PPP1CB	NM_206877
0.42	PPP1R10	NM_002714
0.57	PPP2R5C	AK131391
0.55	PPP2R5E	NM_006246
0.49	PPP5C	NM_006247
0.46	PPT2	NM_138717
0.58	PRDM8	NM_020226
0.48	PREP	NM_002726
0.45	PRG4	NM_005807
0.43	PRKACB	CR936631
0.51	PRKAR1B	BC026734
0.41	PRO0132	BC020670
0.55	PRO1880	AF118080
0.51	PROM2	NM_144707
0.5	PROP1	NM_006261
0.53	PRPF40B	NM_001031698
0.53	PRR6	NM_181716
0.46	PRRT1	NM_030651
0.43	PSD3	NM_206909
0.51	PSMA4	NM_002789
0.65	PSMB9	U01025
0.42	PSMD6	NM_014814
0.58	PSRC2	NM_144982
0.76	PTCHD2	AL117236
0.47	PTN	NM_002825
0.54	PTPRC	Y00638
0.44	PXT1	NM_152990
0.54	PYCR2	AL834217
0.52	QKI	NM_206855
0.41	QPCT	NM_012413
0.45	RAB11FIP1	NM_001002233
0.53	RAB21	NM_014999
0.58	RAB4A	NM_004578
0.82	RAB7	NM_004637
0.49	RAC2	NM_002872
0.56	RAD1	NM_002853
0.47	RAD23A	NM_005053
0.37	RAD54L	NM_003579
0.45	RAMP1	NM_005855
0.48	RANBP6	NM_012416
0.49	RAPGEF4	NM_007023
0.42	RARB	NM_016152
0.55	RASD2	NM_014310
0.52	RASGEF1C	NM_001031799
0.67	RASGRF2	NM_006909
0.5	RASSF5	NM_182664
0.46	RDBP	NM_002904
0.47	RET	NM_020975
0.42	RETNLB	NM_032579
0.48	REXO4	NM_020385
0.48	RFC1	NM_002913
0.44	RFP	NM_030950
0.42	RFT1	NM_052859

0.62	EYA2	AY705349
0.67	F2R	NM_001992
0.66	F3	NM_001993
0.77	FABP2	NM_000134
0.65	FABP4	NM_001442
0.71	FAHD1	BC020615
0.56	FALZ	NM_182641
0.74	FAM104A	NM_032837
0.71	FAM106A	BC098363
0.52	FAM109A	AK093052
0.71	FAM109B	NM_001002034
0.5	FAM10A5	AF512499
0.88	FAM111A	NM_198847
0.46	FAM112A	NM_176791
0.6	FAM11A	NM_032508
0.8	FAM12A	NM_006683
0.94	FAM13A1	NM_001015045
0.57	FAM21C	BC006456
1.35	FAM24A	NM_001029888
0.53	FAM24B	NM_152644
0.56	FAM27L	NM_203392
0.59	FAM33A	NM_182620
0.91	FAM35A	NM_019054
0.63	FAM39A	XM_933149
0.73	FAM40A	AK125944
0.54	FAM43A	NM_153690
0.64	FAM44B	NM_138369
0.54	FAM45B	NM_018472
0.63	FAM49A	NM_030797
0.76	FAM50A	NM_004699
1.26	FAM51A1	NM_017856
0.49	FAM57A	NM_024792
0.93	FAM5C	BC105052
0.59	FAM60A	NM_021238
0.62	FAM64A	NM_019013
0.5	FAM65A	NM_024519
0.62	FAM7A2	XM_931050
0.7	FAM84A	NM_145175
0.78	FAM91A1	NM_144963
0.88	FAM96A	NM_032231
0.54	FAM9A	NM_174951
0.54	FAM9B	NM_205849
0.6	FAM9C	NM_174901
0.83	FANCA	NM_001018112
0.47	FANCB	NM_152633
0.91	FANK1	AY251163
1.13	FARP2	NM_014808
0.62	FARSLA	NM_004461
0.77	FAT4	NM_024582
0.47	FATE1	NM_033085
0.73	FAU	NM_001997
0.8	FBL	NM_001436
0.65	FBN1	NM_000138
0.74	FBN2	NM_001999
0.62	FBXL10	NM_032590
0.55	FBXL14	NM_152441
0.82	FBXL2	NM_012157
0.59	FBXL22	NM_203373
0.62	FBXO15	NM_152676
0.68	FBXO46	XM_934743
0.59	FBXO47	NM_001008777
0.65	FBXO7	NM_012179
0.68	FBXO9	NM_012347
0.51	FBXW9	NM_032301
0.51	FCGBP	NM_003890
0.82	FEM1B	NM_015322
0.6	FEN1	NM_004111
1.35	FER1L3	NM_133337
0.88	FERD3L	NM_152898
0.54	FFAR1	NM_005303
0.6	FGD1	NM_004463
0.77	FGF10	NM_004465
0.49	FGF11	NM_004112
0.56	FGF14	NM_175929
0.5	FGF18	NM_033649
0.6	FGF2	NM_002006
0.59	FGF23	NM_020638
0.59	FGF9	NM_002010
0.69	FGFR1OP2	NM_015633
0.79	FHL1	NM_001449
0.82	FIGF	NM_004469
0.67	FIP1L1	AK090938
0.59	FKBP10	AK025874
0.47	FLI1	NM_002017
0.46	FLII	NM_002018
0.52	FLJ10038	AK000900
0.52	FLJ10120	XM_934274
0.82	FLJ10213	NM_018029
0.91	FLJ10292	NM_018048
1.09	FLJ10357	CR618390
0.74	FLJ10404	NM_019057
0.45	FLJ10781	NM_018215
0.72	FLJ11021	NM_198262
0.52	FLJ11506	NM_024666
0.8	FLJ11806	NM_207661
1.24	FLJ12331	NM_024986
0.82	FLJ12529	NM_024811
0.65	FLJ12986	AK023048
0.72	FLJ13639	NM_024705
0.52	FLJ13769	AK023831
0.74	FLJ14154	BC011267
0.76	FLJ14213	BC008922
0.54	FLJ14640	NM_032816
0.96	FLJ14668	NM_032822
0.8	FLJ14768	NM_032836
0.69	FLJ14834	NM_032849
0.64	FLJ16171	NM_001004348
0.77	FLJ16360	NM_001023561
0.63	FLJ20054	BC074735
0.54	FLJ20105	NM_017669
0.66	FLJ20160	NM_017694
0.67	FLJ20280	NM_017741
0.82	FLJ20487	NM_017841
0.65	FLJ20551	NM_017875
0.88	FLJ20582	NM_014106
0.9	FLJ20647	NM_017918
1.47	FLJ20674	BC034471
0.69	FLJ21103	CR625659
0.69	FLJ21127	NM_024549
0.82	FLJ21687	NM_024859
0.73	FLJ21901	BC032687
1.14	FLJ22028	NM_024854
0.77	FLJ22184	AK096643
0.74	FLJ22222	NM_175902
1.1	FLJ22349	NM_024821
0.85	FLJ22596	AK026249
0.72	FLJ22795	BC007811
0.74	FLJ23577	AK026817
0.77	FLJ25006	NM_144610

0.54	RGAG1	NM_020769
0.45	RGS1	NM_002922
0.42	RGS14	NM_006480
0.48	RGS2	NM_002923
0.46	RGS3	NM_144488
0.6	RHEBL1	NM_144593
0.48	RHOH	NM_004310
0.72	RIBC1	NM_144968
0.43	RINT1	NM_021930
0.48	RIPK3	NM_006871
0.52	RLF	NM_012421
0.41	RNASEH1	NM_002936
0.43	RNASEL	NM_021133
1.04	RNASEN	NM_013235
0.44	RNF14	NM_183401
0.55	RNF185	NM_152267
0.62	RNF25	NM_022453
0.55	RNF32	NM_030936
0.34	RNGTT	NM_003800
0.4	ROPN1B	BC015413
0.57	RP11-321G1.1	AK000305
0.46	RP11-35N6.1	NM_207299
0.48	RP11-444E17.5	XM_496810
0.45	RP5-1065J22.5	XM_059104
0.48	RPE65	NM_000329
0.69	RPL14	NM_003973
0.42	RPL22L1	BC107708
0.5	RPRC1	NM_018067
0.56	RRAGA	NM_006570
0.39	RRBP1	NM_004587
0.42	RREB1	NM_002955
0.41	RTN4	AF063601
0.41	RUNX1T1	NM_004349
0.59	RUNX2	NM_004348
0.39	RY1	NM_006857
0.62	S100PBP	AK022965
0.5	SALL2	NM_005407
0.41	SCARA5	NM_173833
0.64	SCARF1	NM_145352
0.54	SCMH1	NM_001031694
0.61	SCN10A	NM_006514
0.41	SCN3A	NM_006922
0.6	SCNM1	NM_024041
0.47	SCO2	NM_005138
0.96	SCYL2	NM_017988
0.62	SEC24D	NM_014822
0.52	SENP2	NM_021627
0.57	SERPINE1	NM_000602
0.43	SERPINE2	BC042628
0.6	SESTD1	NM_178123
0.5	SETD5	XM_931471
0.57	SF1	BC038446
0.58	SFRS11	NM_004768
0.5	SFRS12	NM_139168
0.41	SFT2D3	NM_032740
0.5	SH2BP1	NM_014633
0.44	SH2D3C	NM_005489
0.54	SH3GL2	NM_003026
0.4	SHB	X75342
0.46	SKIV2L	NM_006929
0.43	SLA	NM_006748
0.4	SLC11A2	NM_000617
0.46	SLC12A8	BC020506
0.59	SLC13A1	NM_022444
0.48	SLC22A4	NM_003059
0.44	SLC23A1	NM_152685
0.55	SLC25A1	NM_005984
0.6	SLC25A4	NM_001151
0.67	SLC27A6	NM_014031
0.66	SLC29A2	NM_001532
0.48	SLC31A2	NM_001860
0.63	SLC35B4	NM_032826
0.48	SLC37A2	NM_198277
0.41	SLC39A8	NM_022154
0.48	SLC43A2	NM_152346
0.48	SLC44A5	NM_152697
0.56	SLC5A6	BC012806
0.49	SLC5A7	NM_021815
0.49	SLC7A1	BC069358
0.41	SLC7A13	NM_138817
0.48	SLC7A14	NM_020949
0.45	SLC7A2	AL832016
0.47	SLC8A1	BX648299
0.46	SMARCA2	BC066596
0.72	SMC1L1	NM_006306
0.4	SMC4L1	NM_005496
0.41	SMG7	NM_201569
0.61	SMNDC1	NM_005871
0.54	SNRPD1	NM_006938
0.47	SNRPD3	NM_004175
0.48	SNW1	NM_012245
0.35	SNX25	BC029868
0.44	SNX9	NM_016224
0.5	SOAT1	NM_003101
0.45	SP110	NM_080424
0.45	SP140	U63420
0.6	SP3	NM_003111
0.35	SPANX-N4	NM_001009613
0.5	SPATA2	NM_006038
0.51	SPATC1	NM_198572
0.39	SPBC25	NM_020675
0.46	SPINK5	NM_006846
0.48	SPP1	NM_001040060
0.5	SPRR4	NM_173080
0.48	SPSB4	NM_080862
0.47	SPTBN1	NM_178313
0.47	SRD5A2	NM_000348
0.46	SRFBP1	NM_152546
0.48	SRM	NM_003132
0.41	SRP9	NM_003133
0.58	SSBP2	NM_012446
0.46	SSNA1	NM_003731
0.53	SSPO	XM_934888
0.52	ST6GALNAC3	NM_152996
0.48	STAG2	NM_006603
0.53	STAT4	NM_003151
0.48	STRBP	NM_018387
0.75	STX10	NM_003765
0.54	STX6	NM_005819
0.57	STX8	NM_004853
0.55	SUZ12	NM_015355
0.42	SVEP1	AK075235
0.48	SYTL3	NM_001009991
0.48	TAF11	NM_005643
0.43	TAF12	NM_005644
0.7	TAF5L	NM_014409
0.55	TAS2R39	NM_176881
0.43	TAS2R60	NM_177437
0.5	TBC1D14	NM_020773

0.63	FLJ25222	BC063682
0.44	FLJ25404	BC101436
0.48	FLJ25773	NM_182560
0.65	FLJ26850	NM_001001687
0.63	FLJ30046	NM_144595
0.66	FLJ30294	BC020898
0.64	FLJ30655	NM_144643
0.69	FLJ30707	NM_145019
0.9	FLJ30851	BC050462
0.56	FLJ31196	NM_152908
0.5	FLJ31875	NM_182531
0.47	FLJ31958	AK056520
0.56	FLJ32310	NM_152336
0.89	FLJ32784	AK057346
1.13	FLJ32810	XM_925815
1.96	FLJ32926	NM_144577
0.74	FLJ33706	NM_182584
0.65	FLJ33860	NM_173644
1.08	FLJ34047	NM_173669
0.89	FLJ34306	NM_199340
0.49	FLJ35773	NM_152599
0.95	FLJ35834	NM_178827
0.62	FLJ35894	NM_173616
0.65	FLJ36004	NM_152590
0.57	FLJ36070	NM_182574
1.07	FLJ36180	NM_178556
0.68	FLJ36701	NM_173617
0.45	FLJ36888	BC023623
0.95	FLJ38288	NM_173632
0.6	FLJ38451	NM_175872
0.68	FLJ38717	NM_001004322
0.56	FLJ38725	NM_153218
0.83	FLJ39639	XM_370932
1.03	FLJ40172	NM_173649
0.5	FLJ40411	XM_496622
1.13	FLJ40536	AK097855
0.73	FLJ40712	NM_207484
0.62	FLJ40722	AK098041
0.72	FLJ40852	NM_173677
0.91	FLJ41309	XM_928122
0.52	FLJ42022	XM_929698
0.65	FLJ42177	AK124171
0.71	FLJ42200	AK124194
0.69	FLJ42291	NM_207367
0.59	FLJ42953	NM_207474
0.34	FLJ43315	BC057848
0.7	FLJ43706	XM_927613
0.68	FLJ43980	NM_001004299
0.63	FLJ43987	XM_371500
0.77	FLJ45231	XM_928121
0.66	FLJ45300	NM_001001681
0.65	FLJ45508	XM_928644
0.71	FLJ45530	NM_001001682
0.64	FLJ45721	NM_207490
0.56	FLJ46020	NM_207472
0.44	FLJ46121	XM_930273
0.79	FLJ46154	NM_198462
0.58	FLJ46156	NM_198499
0.54	FLJ90231	NM_173581
0.54	FLJ90396	NM_153358
0.49	FLJ90757	NM_001004336
0.93	FLOT2	BC017292
0.5	FLRT1	NM_013280
0.9	FLRT2	NM_013231
0.56	FLT1	NM_002019
0.77	FMNL1	NM_005892
0.62	FMNL3	NM_198900
0.76	FMO1	NM_002021
0.86	FMO3	NM_006894
0.66	FMR1NB	NM_152578
0.73	FNBP1L	AK000282
1.23	FNBP4	NM_015308
1.58	FNDC3A	NM_014923
0.88	FNDC3B	BC039297
0.66	FNDC4	BC032725
0.48	FOSB	NM_006732
0.55	FOXA1	NM_004496
1.53	FOXM1	NM_202003
0.55	FOXN4	NM_213596
1.09	FOXO3A	NM_201559
0.6	FOXP3	NM_014009
0.85	FOXRED1	NM_017547
0.66	FPGT	NM_003838
1.01	FPR1	NM_002029
0.68	FPRL1	BC029125
0.92	FPRL2	BC069070
0.63	FRMPD2	NM_001018071
0.71	FTL	NM_000146
0.66	FTSJ1	NM_177439
0.65	FTSJ3	NM_017647
0.59	FURIN	NM_002569
0.66	FUT4	NM_002033
0.57	FXYD6	NM_022003
0.5	FZD4	NM_012193
0.64	GAB1	AK022142
0.65	GABARAP	NM_007278
0.52	GABPB2	NM_181427
1.01	GABRA3	NM_000808
0.79	GABRE	Y07637
0.86	GAD1	NM_013445
0.8	GAK	NM_005255
0.91	GAL3ST3	NM_033036
0.87	GALC	NM_001037525
0.62	GALNS	NM_000512
0.88	GALNT1	NM_020474
0.72	GALNT4	NM_003774
0.72	GALNT6	NM_007210
0.92	GALR1	NM_001480
0.52	GALR2	NM_003857
0.88	GAP43	NM_002045
0.52	GART	NM_175085
0.6	GATA1	NM_002049
0.69	GATAD1	NM_021167
0.57	GATAD2A	NM_017660
0.59	GATM	NM_001482
0.62	GATS	AK055399
0.76	GBF1	NM_004193
0.66	GBP4	NM_052941
0.63	GBP7	NM_207398
0.66	GCKR	NM_001486
0.59	GCP4	NM_014444
0.83	GDAP2	NM_017686
0.53	GDF2	NM_016204
1	GDF8	NM_005259
0.63	GDI1	NM_001493
1.1	GDPD4	NM_182833
0.62	GEMIN4	NM_015721
0.68	GEMIN7	NM_024707
0.83	GFOD2	NM_030819

0.46	TBC1D2	NM_018421
0.49	TBK1	NM_013254
0.39	TBX15	NM_152380
0.61	TBX20	NM_020417
0.54	TCEAL7	NM_152278
0.48	TCEAL8	NM_153333
0.66	TCEB3	NM_003198
0.6	TCF7L1	NM_031283
0.49	TCN2	NM_000355
0.54	TDRD3	BC060876
0.62	TDRD5	NM_173533
0.44	TDRD6	NM_001010870
0.44	TEC	NM_003215
0.45	TESK2	NM_007170
0.4	TEX264	NM_015926
0.44	TFEB	NM_007162
0.6	TGFBI	NM_000358
0.47	TGFBRAP1	NM_004257
0.58	THAP2	NM_031435
0.72	THAP4	NM_015963
0.56	THEG	NM_199202
0.55	TIAF1	NM_004740
0.59	TIAM2	AF120323
0.57	TINAG	NM_014464
0.5	TLN1	NM_006289
0.44	TLR3	NM_003265
0.4	TLR4	NM_138557
0.59	TM4SF18	NM_138786
0.42	TM4SF4	NM_004617
0.51	TM6SF1	NM_023003
0.6	TMC3	XM_930010
0.62	TMCC2	NM_014858
0.49	TMEM106C	NM_024056
0.37	TMEM12	NM_152311
0.55	TMEM123	NM_052932
0.7	TMEM132A	AK023577
0.49	TMEM147	NM_032635
0.46	TMEM14A	NM_014051
0.4	TMEM14B	NM_030969
0.57	TMEM41A	NM_080652
0.45	TMEM50A	NM_014313
0.57	TMEM63B	NM_018426
0.48	TMEM69	NM_016486
0.52	TMEM80	NM_174940
0.46	TMPRSS11F	NM_207407
0.4	TNC	BX641111
0.39	TNFAIP8L2	NM_024575
0.49	TNFRSF12A	NM_016639
0.52	TNFRSF8	NM_001243
0.48	TNK1	NM_003985
0.58	TNN	NM_022093
0.41	TNP1	NM_003284
0.44	TOPBP1	NM_007027
0.61	TP53RK	NM_033550
0.6	TRADD	NM_003789
0.46	TRAF2	BC064662
0.46	TRAK1	AB028965
0.53	TRAK2	NM_015049
0.54	TREM1	NM_018643
0.34	TRIM10	NM_052828
0.41	TRIM11	NM_145214
0.34	TRIM15	NM_052812
0.59	TRIM26	NM_003449
0.44	TRIM39	NM_172016
0.63	TRIM4	NM_033091
0.5	TRIM45	NM_025188
0.46	TRIM61	NM_001012414
0.52	TRIO	AK131423
0.47	TRIP12	NM_004238
0.5	TSC1	NM_001008567
0.52	TSC22D1	NM_006022
0.54	TSPAN1	NM_005727
0.53	TSPAN33	NM_178562
0.6	TSSK3	NM_052841
0.46	TTBK1	NM_032538
0.58	TTC7A	NM_020458
0.42	TTF1	NM_007344
0.42	TTLL3	NM_015644
0.52	TUBGCP3	NM_006322
0.52	TUFT1	NM_020127
0.56	TXK	NM_003328
0.62	UBAP1	NM_016525
0.69	UBE1C	NM_198197
0.47	UBE2L3	NM_198157
0.48	UBTF	NM_014233
0.43	UCK2	NM_012474
0.41	UGT2A3	NM_024743
0.64	UNC119	NM_054035
0.51	UNC45A	NM_018671
0.42	UNC5A	NM_133369
0.43	UNC5D	AY358147
0.63	UNQ846	NM_207316
0.55	UNQ9391	NM_198464
0.52	UNQ9438	NM_207377
0.55	UPF2	NM_080599
0.48	USP25	AK022574
0.41	USP48	AK026707
0.63	USPL1	NM_005800
0.43	VANGL1	BC000907
0.83	VAV3	AF118886
0.47	VCPIP1	NM_025054
0.91	VDAC2	NM_003375
0.56	VDP	NM_003715
0.51	VMD2L3	NM_152439
0.41	VPS72	NM_005997
0.56	VSIG8	NM_001013661
0.56	VSX1	NM_199425
0.5	WAPAL	BC017393
0.54	WDFY4	BC032420
0.57	WDR16	NM_145054
0.38	WDR5	NM_052821
0.57	WDR50	NM_016001
0.76	WDR7	NM_052834
0.64	WDR76	NM_024908
0.43	WDR77	NM_024102
0.41	WFS1	NM_006005
0.47	WIPI2	NM_016003
0.41	WISP1	NM_080838
0.48	WNK2	NM_006648
0.6	WNT10A	NM_025216
0.43	XAB1	NM_007266
0.48	XK	NM_021083
0.41	XKR9	NM_001011720
0.47	XRCC5	NM_021141
0.51	YAF2	NM_005748
0.62	YARS	NM_003680
0.41	YWHAQ	NM_006826
0.52	ZBTB1	NM_014950
0.52	ZBTB25	NM_006977

0.71	GGTL3	NM_178026
0.82	GHITM	NM_014394
0.69	GIMAP2	NM_015660
0.72	GIMAP5	AK055568
0.45	GIOT-1	NM_153257
0.6	GJB1	NM_000166
0.65	GLI1	NM_005269
0.63	GLMN	NM_053274
0.49	GLP2R	NM_004246
0.88	GLS2	NM_138566
0.63	GLT25D2	AK095090
1.11	GLT8D3	XM_290597
0.55	GLTP	NM_016433
0.57	GLYATL1	BC008353
0.75	GLYCAM1	XM_934129
0.75	GLYCAM1	XM_934132
0.54	GMFG	NM_004877
0.89	GNA15	NM_002068
0.56	GNAO1	NM_138736
0.48	GNAZ	BC096828
0.72	GNB5	NM_016194
1.22	GNG2	BC020774
0.66	GNG3	NM_012202
0.78	GNGT1	NM_021955
0.78	GNRH1	NM_000825
0.74	GOLGA5	NM_005113
0.65	GOLGA8A	CR749361
0.65	GOLGA8B	NM_001023567
0.71	GOLPH3	NM_022130
0.8	GON4L	AK026693
1.01	GPC4	NM_001448
0.72	GPC5	U66033
0.82	GPHA2	NM_130769
0.69	GPIAP1	NM_203364
0.54	GPM6B	NM_005278
0.83	GPR1	NM_005279
0.5	GPR114	NM_153837
1.09	GPR135	NM_022571
1.07	GPR143	NM_000273
0.6	GPR32	NM_001506
0.79	GPR34	NM_005300
0.99	GPR37L1	NM_004767
0.57	GPR44	NM_004778
0.48	GPR68	BC096071
0.73	GPR82	NM_080817
0.55	GPR84	NM_020370
0.99	GPR87	NM_023915
0.57	GPRASP2	NM_138437
0.53	GRAP2	NM_004810
0.59	GRB10	NM_001001555
0.63	GRIA3	NM_181894
0.74	GRID2	NM_001510
0.8	GRIK1	NM_175611
0.57	GRIN2D	NM_000836
0.87	GRIN3A	NM_133445
1.15	GRINL1A	NM_015532
0.64	GRK6	NM_002082
0.68	GRN	NM_002087
0.57	GRPR	NM_005314
0.56	GRTP1	NM_024719
0.64	GSC	NM_173849
0.71	GSG2	NM_031965
0.51	GSK3A	NM_019884
0.63	GSPT2	NM_018094
0.78	GSTA4	NM_001512
0.68	GSTA5	NM_153699
0.57	GSTCD	NM_001031720
0.77	GSTZ1	NM_145871
0.99	GTF2E1	AK223401
0.57	GTF2F1	NM_002096
0.66	GTF2H1	NM_005316
0.52	GTF2H3	NM_001516
0.75	GTF2H5	NM_207118
1.18	GTF3A	NM_002097
0.8	GTF3C3	NM_012086
0.51	GTSE1	NM_016426
0.6	GUCY1A2	Z50053
0.98	GUCY2C	NM_004963
0.8	GUCY2D	NM_000180
0.57	GUCY2F	NM_001522
0.67	GUF1	NM_021927
0.66	GULP1	NM_016315
0.6	GVIN1	BX538342
0.5	GYG2	U94364
0.49	GYS2	NM_021957
0.75	H1FNT	NM_181788
0.73	H3/o	NM_001005464
0.57	HABP2	NM_004132
0.71	HAGH	NM_005326
0.74	HAPLN1	NM_001884
0.57	HBE1	NM_005330
0.63	HBG1	NM_000559
0.67	HCAP-G	NM_022346
0.68	HCRP1	AY465895
0.84	HDAC3	NM_003883
0.63	HDAC4	NM_006037
0.98	HDAC6	NM_006044
0.66	HDAC8	NM_018486
0.7	HDCMA18P	NM_016648
1.08	HDGFRP3	NM_016073
0.66	HDLBP	NM_005336
0.63	HECTD3	NM_024602
1.07	HELLS	BC030963
0.84	HEMGN	NM_197978
0.83	HEMK2	NM_182749
0.72	HERC1	NM_003922
0.56	HES7	NM_032580
0.59	HEXA	NM_000520
0.74	HEXDC	NM_173620
1.02	HEY2	NM_012259
0.63	HHEX	NM_002729
0.77	HHIP	NM_022475
0.58	HHLA2	NM_007072
0.91	HIF1AN	NM_017902
0.51	HIF3A	NM_152794
0.93	HIGD1B	NM_016438
0.74	HINT1	NM_005340
0.8	HIRA	X77633
0.65	HIST1H1E	NM_005321
0.65	HIST1H2AE	NM_021052
0.85	HIST1H2AG	NM_021064
0.65	HIST1H2BD	NM_138720
0.92	HIST1H2BF	NM_003522
0.85	HIST1H2BJ	NM_021058
0.68	HIST1H4B	NM_003544
0.73	HIST2H2AA	BC096705
0.73	HIST2H3C	NM_021059
0.65	HIVEP1	NM_002114
0.66	HK2	BC064369
0.46	ZBTB34	BC020929
0.66	ZBTB37	NM_032522
0.44	ZBTB43	NM_014007
0.56	ZC3H11A	NM_014827
0.6	ZCCHC10	NM_017665
0.41	ZDHHC2	NM_016353
0.55	ZFP36L1	NM_004926
0.63	ZKSCAN1	BC112224
0.5	ZMAT2	NM_144723
0.44	ZNF141	NM_003441
0.56	ZNF181	NM_001029997
0.56	ZNF19	BC048974
0.56	ZNF23	NM_145911
0.54	ZNF283	XM_371174
0.46	ZNF322A	BC050425
0.46	ZNF322B	NM_199005
0.43	ZNF335	NM_022095
0.5	ZNF365	BX647904
0.53	ZNF385	BC029752
0.53	ZNF467	NM_207336
0.61	ZNF521	NM_015461
0.58	ZNF569	NM_152484
0.58	ZNF570	NM_144694
0.56	ZNF607	NM_032689
0.36	ZNF613	NM_024840
0.47	ZNF616	BC032805
0.41	ZNF644	NM_201269
0.75	ZNF655	NM_138494
0.48	ZNF660	NM_173658
0.59	ZNF662	NM_207404
0.36	ZNF664	BC093848
0.54	ZNF692	NM_017865
0.49	ZNF7	NM_003416
0.42	ZNRD1	NM_170783
0.41	ZPBP	NM_007009
0.41	ZUBR1	AB007931
0.52	ZYG11B	NM_024646

0.71	HKR1	BC053845
0.74	HKR2	NM_181846
0.75	HLA-DMB	NM_002118
0.71	HLA-DQA2	NM_020056
0.54	HLRC1	NM_031304
0.76	HMCN1	AL833232
0.78	HMFN0672	AK129923
0.53	HMGB1	BX647267
0.77	HMGN1	NM_004965
0.56	HMOX1	NM_002133
0.97	HNRPH1	NM_005520
0.6	HNRPUL2	XM_495877
0.69	HNT	NM_016522
1.08	HOMER2	NM_199332
0.77	HOP	NM_139211
0.62	HOXA1	S79910
0.99	HOXB2	NM_002145
0.59	HOXC12	NM_173860
0.76	HOXD12	NM_021193
0.59	HP	NM_005143
0.57	HPN	NM_182983
0.68	HPR	NM_020995
1.04	HPS4	NM_152842
0.66	HPS5	NM_181508
0.85	HPS6	NM_024747
0.88	HPSE2	NM_021828
0.95	HPX	NM_000613
1.24	HRK	NM_003806
0.98	HS3ST2	NM_006043
0.49	HS3ST3A1	BC044647
0.86	HS3ST4	NM_006040
0.5	HSD11B1	NM_181755
0.6	HSD17B12	NM_016142
0.64	HSD17B13	NM_178135
0.53	HSDL1	BC104218
0.59	HSMPP8	BC046214
0.79	HSP90AB4P	XM_930214
0.64	HSPA4	X67643
0.79	HSPA8	NM_153201
0.63	HSPB2	NM_001541
0.99	HSPC072	NM_014162
0.59	HSPC105	NM_145168
0.57	HSPC142	NM_014173
1.45	HSPC148	NM_016403
1.04	HSPC171	NM_014187
0.62	HSPC176	BC017440
0.99	HSPG2	X62515
0.88	HTATIP2	NM_006410
1.12	HTR2A	NM_000621
0.74	HTR4	Y12507
0.79	HTRA1	NM_002775
0.88	HVCN1	NM_032369
0.74	HYDIN	NM_017558
0.62	HYPE	NM_007076
0.69	HYPK	AK000438
0.68	IBRDC1	NM_152553
0.59	ICA1	NM_022308
0.94	ICF45	NM_017872
0.63	ID3	NM_002167
0.85	IDH3A	NM_005530
0.52	IDH3B	NM_174856
0.79	IDS	NM_006123
0.86	IFI16	AK094968
0.86	IFI30	NM_006332
0.53	IFIT3	NM_001031683
0.69	IFITM1	NM_003641
0.6	IFITM2	NM_006435
0.5	IFITM3	NM_021034
0.83	IFNAR2	X89814
0.94	IFNE1	NM_176891
0.69	IFNG	NM_000619
0.78	IFRD1	NM_001550
0.66	IFT172	NM_015662
0.56	IFT20	NM_174887
1.05	IFT88	NM_175605
0.73	IGBP1	NM_001551
0.72	IGFBP6	NM_002178
0.6	IGFL4	NM_001002923
0.68	IGHA1	BC016369
0.52	IGHD	BC063384
0.48	IGHG1	BC090940
0.64	IGHG3	BC033178
0.68	IGHG4	BC025985
0.71	IGHM	BC073767
0.8	IGLC1	BC089414
0.45	IGLC2	BC093098
0.68	IGLV4-3	BC020236
0.73	IGSF22	NM_173588
0.89	IGSF4C	NM_145296
0.72	IL16	NM_172217
0.68	IL17	NM_002190
1.06	IL17E	NM_172314
0.51	IL17RC	NM_153463
0.5	IL18BP	NM_173044
0.66	IL18RAP	BC106765
0.9	IL1A	BC013142
0.63	IL1F6	NM_014440
0.82	IL1RAP	NM_134470
0.6	IL1RL2	NM_003854
0.7	IL1RN	X52015
0.44	IL21R	NM_021798
0.59	IL22	NM_020525
0.48	IL28A	NM_172138
0.62	IL28B	NM_172139
0.5	IL3RA	NM_002183
0.91	ILK	NM_004517
0.47	IMAA	BC071656
0.66	IMMP1L	NM_144981
0.65	IMPAD1	AK090988
0.59	INDOL1	NM_194294
0.82	INE1	NM_003669
0.59	INOC1	BX640651
0.57	INTS12	NM_020395
0.7	IQWD1	AF150734
0.85	IRAK1	NM_001569
1.31	IRAK4	NM_016123
1.04	IRF1	NM_002198
0.6	IRF3	BC071721
0.82	ISG20	NM_002201
0.52	ISG20L1	NM_022767
0.98	ITFG1	NM_030790
0.77	ITGA1	NM_181501
0.65	ITGAE	NM_002208
0.66	ITGB1BP2	NM_012278
0.59	ITGB7	NM_000889
0.47	ITM2A	NM_004867
0.66	IWS1	NM_017969
0.86	IXL	NM_017592
0.62	JAK2	NM_004972

0.88	JM11	NM_033626
1.45	JMJD2D	NM_018039
0.6	JRKL	NM_003772
0.77	JUND	NM_005354
0.69	K6IRS2	NM_080747
0.52	KA21	NM_152349
0.92	KATNAL1	NM_032116
1.05	KATNAL2	NM_031303
0.79	KAZALD1	NM_030929
0.78	KBTBD2	NM_015483
0.76	KBTBD6	NM_152903
0.54	KCNA4	NM_002233
0.62	KCNA5	NM_002234
0.57	KCNA7	NM_031886
0.8	KCNAB3	NM_004732
0.55	KCNB1	NM_004975
1.04	KCNC2	NM_153748
0.69	KCND1	NM_004979
0.72	KCND2	NM_012281
0.8	KCNE1	NM_000219
0.73	KCNE1L	NM_012282
0.49	KCNE2	NM_172201
0.65	KCNH4	NM_012285
0.8	KCNH6	NM_173092
0.65	KCNJ15	NM_170737
0.59	KCNJ6	NM_002240
0.69	KCNJ8	NM_004982
0.52	KCNK10	NM_138318
0.6	KCNMA1	U11058
0.95	KCNMB2	NM_005832
0.49	KCNRG	NM_199464
0.77	KCNS1	NM_002251
0.62	KCR1	NM_001013620
0.52	KCTD10	NM_031954
0.96	KCTD18	NM_152387
0.59	KCTD19	XM_085367
0.47	KCTD5	NM_018992
0.69	KDELC2	NM_153705
0.57	KDELR1	NM_006801
0.65	KDELR3	NM_016657
1.07	KEAP1	BC015945
1.2	KIAA0020	NM_014878
0.73	KIAA0040	NM_014656
0.65	KIAA0101	NM_014736
0.95	KIAA0152	NM_014730
0.78	KIAA0240	NM_015349
0.77	KIAA0317	NM_014821
1.03	KIAA0329	NM_014844
0.95	KIAA0355	NM_014686
0.68	KIAA0376	NM_015330
0.91	KIAA0409	NM_015324
0.56	KIAA0513	NM_014732
0.83	KIAA0553	NM_001002909
0.47	KIAA0556	NM_015202
1.06	KIAA0586	NM_014749
0.62	KIAA0644	NM_014817
0.47	KIAA0683	NM_016111
0.59	KIAA0701	NM_015054
0.68	KIAA0753	BC113017
0.59	KIAA0774	NM_001033602
0.69	KIAA0789	NM_014653
0.65	KIAA0841	XM_932194
0.69	KIAA0895	NM_015314
0.81	KIAA0947	AB023164
0.82	KIAA0999	NM_025164
0.47	KIAA1005	NM_015272
0.88	KIAA1128	BC030528
0.7	KIAA1212	NM_018084
0.57	KIAA1279	NM_015634
0.75	KIAA1370	NM_019600
1.26	KIAA1409	NM_020818
1.02	KIAA1411	AK000183
0.55	KIAA1443	NM_020834
0.68	KIAA1456	NM_020844
0.58	KIAA1509	XM_935330
0.53	KIAA1598	NM_018330
0.68	KIAA1622	NM_058237
0.85	KIAA1632	BC036911
0.53	KIAA1704	NM_018559
0.62	KIAA1713	AB051500
0.67	KIAA1727	NM_033393
0.54	KIAA1731	AB051518
0.92	KIAA1840	BC024161
0.55	KIAA1853	NM_194286
0.78	KIAA1900	NM_052904
0.64	KIAA1909	AK075513
0.65	KIAA1913	NM_052913
0.5	KIAA1924	AK126622
0.53	KIAA1975	XM_370567
0.66	KIF18A	NM_031217
0.63	KIF1A	NM_004321
1.01	KIF22	NM_007317
0.52	KIF23	NM_138555
0.92	KIF4A	NM_012310
0.75	KIF9	NM_182903
0.68	KIFC3	BX647698
0.36	KIR2DS1	X98858
0.36	KIR2DS2	BC108917
0.47	KIR3DL1	X97232
0.6	KIRREL3	NM_032531
0.81	KLF4	NM_004235
0.84	KLF9	NM_001206
0.95	KLHDC4	AL133107
0.62	KLHDC5	NM_020782
0.65	KLHL14	NM_020805
0.75	KLHL18	NM_025010
0.76	KLHL21	NM_014851
0.87	KLHL5	AK002174
0.64	KLHL8	NM_020803
0.83	KLK2	NM_005551
1.22	KLK6	NM_002774
1.01	KLRD1	BC042884
0.72	KNTC1	NM_014708
0.53	KPNA3	NM_002267
0.56	KPNB1	NM_002265
0.59	KRAS	NM_033360
0.56	KRT10	NM_000421
0.59	KRT23	NM_173213
0.99	KRT25D	NM_181535
1.01	KRT2B	NM_015848
0.52	KRT4	NM_002272
0.49	KRT6B	NM_005555
1.11	KRT6L	NM_175834
0.78	KRT7	NM_005556
0.62	KRTAP10-11	NM_198692
0.52	KRTAP10-9	NM_198690
0.59	KRTAP12-3	NM_198697
0.59	KRTAP12-4	NM_198698
0.52	KRTAP1-3	NM_030966

0.71	KRTAP13-1	NM_181599
0.56	KRTAP13-3	NM_181622
0.56	KRTAP13-4	NM_181600
0.49	KRTAP1-5	NM_031957
0.71	KRTAP19-1	NM_181607
0.52	KRTAP19-2	NM_181608
0.56	KRTAP19-3	NM_181609
0.56	KRTAP19-4	NM_181610
0.52	KRTAP19-5	NM_181611
0.46	KRTAP2-1	XM_932450
1.17	KRTAP21-1	NM_181619
0.77	KRTAP22-1	NM_181620
0.46	KRTAP2-4	BC063625
0.62	KRTAP4-14	NM_033059
0.71	KRTAP4-2	NM_033062
0.54	KRTAP5-6	NM_001012416
0.77	KRTAP6-2	NM_181604
0.49	KRTAP6-3	NM_181605
0.65	KRTHA1	NM_002277
0.83	KRTHA3B	NM_002279
0.59	KRTHA8	NM_006771
0.62	KRTHB6	NM_002284
0.49	Kua	NM_199129
0.49	Kua-UEV	NM_199203
0.92	LACE1	NM_145315
0.65	LACRT	NM_033277
1.86	LARP4	NM_199190
0.77	LARS	AY513284
0.54	LAYN	NM_178834
0.73	LBH	NM_030915
0.6	LCE1D	NM_178352
1.09	LCE3D	NM_032563
0.86	LCE3E	NM_178435
0.75	LCHN	BC012493
1.08	LCMT2	NM_014793
1.18	LCP1	NM_002298
0.49	LCTL	NM_207338
0.57	LDB1	NM_003893
1.25	LECT1	NM_007015
0.68	LENG12	NM_033206
0.69	LEO1	NM_138792
0.52	LETMD1	NM_015416
0.5	LGALS12	NM_033101
0.73	LGALS8	NM_201544
0.64	LGI2	AK126093
0.64	LGMN	NM_005606
0.73	LHFPL1	NM_178175
0.49	LHX5	NM_022363
1.34	LIAS	NM_194451
1.08	LIG3	U40671
0.77	LILRA4	NM_012276
0.57	LILRB4	U82979
0.59	LIMA1	AF198455
0.48	LIN7B	NM_022165
0.72	LINS1	NM_181740
0.59	LITAF	U77396
0.8	LIX1L	NM_153713
0.98	LL22NC01-81G9.2	NM_178552
0.69	LMBR1	NM_022458
0.71	LMCD1	NM_014583
0.57	LMO1	NM_002315
0.5	LMO2	BC073973
0.75	LMO3	NM_018640
0.63	LMO4	NM_006769
0.67	LNX1	NM_032622
0.57	LOC113386	NM_138781
0.73	LOC113444	BC112231
0.88	LOC120379	NM_138789
1.86	LOC121006	XM_926818
0.53	LOC123876	AK091878
0.65	LOC124491	NM_145254
0.57	LOC126075	XM_934608
0.68	LOC129138	AK127583
0.93	LOC130074	NM_001009993
0.82	LOC138046	AK123437
0.57	LOC143941	XM_932933
1.47	LOC144305	BC001437
0.52	LOC145757	NM_001037224
0.65	LOC146177	AK128606
0.62	LOC147650	NM_207324
0.71	LOC162632	BC039345
0.76	LOC196264	NM_198275
0.95	LOC196541	NM_001010977
0.59	LOC196549	NM_145293
0.79	LOC196752	NM_001010864
0.5	LOC197336	NM_145294
0.65	LOC200261	NM_182535
0.7	LOC200383	AL512706
0.79	LOC203427	NM_145305
0.56	LOC220594	NM_145809
0.69	LOC221143	NM_174928
0.9	LOC254028	AK093210
0.64	LOC255187	XM_173160
0.55	LOC283331	NM_001037806
0.47	LOC283849	BC080183
0.56	LOC285927	BC044242
0.68	LOC286260	XM_926851
0.69	LOC286411	AK054978
0.65	LOC338809	NM_001037671
0.51	LOC339674	XM_934917
0.54	LOC387755	AB231748
0.6	LOC387758	NM_203371
0.69	LOC387856	NM_001013635
1.09	LOC387911	NM_001007537
0.58	LOC388022	NM_001013637
1.34	LOC388135	XM_370873
0.72	LOC388152	BC072390
0.62	LOC388335	NM_001004313
0.65	LOC388481	XM_373780
0.48	LOC388503	NM_001013640
0.7	LOC388610	NM_001013642
0.51	LOC388882	NM_001006606
0.68	LOC388885	XM_373949
0.9	LOC389217	XM_371701
0.88	LOC389429	NM_001010905
0.53	LOC389833	BC073934
0.51	LOC389834	NM_001013655
0.7	LOC390414	XM_372494
0.59	LOC390637	NM_001013657
0.47	LOC390667	NM_001013658
0.8	LOC390760	XM_372654
0.49	LOC390811	XM_497556
0.57	LOC390877	XM_372705
0.71	LOC390927	NM_001013659
0.95	LOC390980	NM_001023563
0.77	LOC391257	BC065723
0.6	LOC391356	NM_001013663
0.6	LOC399761	XM_931561
0.53	LOC399815	XM_374836

0.63	LOC399829	XM_927466
0.82	LOC399947	NM_207645
1.41	LOC400388	XM_378523
1.05	LOC400451	NM_207446
0.86	LOC400506	NM_001012991
0.59	LOC400509	NM_001012391
0.59	LOC400553	AK126852
0.51	LOC400707	NM_001013673
0.56	LOC400924	NM_001013676
0.68	LOC401056	XM_379180
0.9	LOC401152	NM_001001701
0.71	LOC401252	NM_001013681
1.29	LOC401280	NM_001013682
0.71	LOC401284	XM_379454
0.68	LOC401498	NM_212558
0.78	LOC401720	NM_001013690
0.95	LOC401911	XM_377527
1.2	LOC439914	AK094364
0.76	LOC440043	XM_495879
0.78	LOC440082	AK131474
0.75	LOC440093	NM_001013699
0.62	LOC440104	XM_927712
0.75	LOC440258	NM_001013702
0.47	LOC440334	XM_498631
0.71	LOC440337	NM_001013705
0.65	LOC440386	XM_498647
0.76	LOC440700	BC012108
0.8	LOC441052	XM_498986
0.98	LOC441294	NM_001008747
0.71	LOC441771	XM_497514
0.75	LOC442247	NM_001013734
0.59	LOC494150	BC014228
0.52	LOC51136	NM_016125
0.71	LOC51334	BC038838
0.88	LOC550643	BX537532
0.52	LOC554175	BC006530
0.88	LOC554203	AK056172
0.47	LOC554206	BC029609
0.54	LOC554234	BC058160
0.62	LOC554250	BC085019
0.62	LOC613206	NM_001033016
0.65	LOC619208	NM_001033564
1.1	LOC63928	NM_022097
0.75	LOC642333	XM_930918
0.49	LOC642432	XM_931151
1.13	LOC642452	XM_926229
0.63	LOC642520	XM_926016
0.59	LOC642534	XM_926470
0.52	LOC642580	XM_930925
1.13	LOC642622	XM_930958
0.48	LOC642648	XM_926474
0.5	LOC642696	XM_926604
1.15	LOC642712	XM_926777
1.19	LOC642757	XM_926793
0.98	LOC642769	XM_926800
0.82	LOC642821	XM_931087
0.53	LOC642834	XM_926933
0.53	LOC642934	XM_931177
0.49	LOC642966	XM_926351
0.55	LOC643018	XM_931244
0.68	LOC643052	XM_926422
0.55	LOC643231	XM_928365
0.74	LOC643260	XM_931443
0.51	LOC643318	XM_928219
0.85	LOC643328	XM_927097
0.53	LOC643386	XM_926717
0.9	LOC643464	XM_931563
1.07	LOC643495	XM_926815
1.08	LOC643625	XM_926927
0.49	LOC643659	XM_929273
0.71	LOC643678	XM_931742
0.6	LOC643687	XM_926989
0.72	LOC643696	XM_933066
0.72	LOC643707	XM_933129
0.51	LOC643761	XM_927051
0.66	LOC643785	XM_931799
0.63	LOC643795	XM_931810
0.49	LOC643868	XM_927138
0.52	LOC643891	XM_927156
0.64	LOC643901	XM_931900
0.6	LOC643986	XM_931962
0.62	LOC644001	XM_931970
0.6	LOC644011	XM_927260
0.57	LOC644106	XM_928596
0.66	LOC644156	XM_932023
1.04	LOC644182	XM_932029
0.72	LOC644216	XM_932049
0.7	LOC644224	XM_932054
0.49	LOC644229	XM_930055
0.88	LOC644241	XM_933651
0.64	LOC644248	XM_932073
0.63	LOC644302	XM_932115
0.63	LOC644338	XM_927502
0.62	LOC644348	XM_932138
0.74	LOC644349	XM_932139
0.49	LOC644361	XM_932144
0.55	LOC644542	XM_932235
0.5	LOC644544	XM_927666
1.02	LOC644715	XM_932323
0.62	LOC644802	XM_927897
0.63	LOC644883	XM_927970
1	LOC644925	XM_934676
0.74	LOC644946	XM_930409
0.74	LOC644951	XM_928035
0.76	LOC644961	XM_930420
0.64	LOC645006	XM_930170
0.59	LOC645085	XM_928124
0.74	LOC645113	XM_930333
0.74	LOC645125	XM_932659
0.56	LOC645127	XM_932663
0.59	LOC645212	XM_928252
0.91	LOC645238	XM_930310
0.89	LOC645276	XM_932782
0.8	LOC645346	XM_932837
0.68	LOC645364	XM_928406
0.64	LOC645370	XM_932854
0.62	LOC645401	XM_932879
0.8	LOC645442	XM_932901
0.95	LOC645485	XM_932931
0.6	LOC645497	XM_928520
0.8	LOC645555	XM_932966
0.62	LOC645569	XM_928587
0.64	LOC645594	XM_932987
1	LOC645602	XM_932995
0.69	LOC645617	XM_933003
0.77	LOC645663	XM_933036
1.13	LOC645723	XM_933083
0.73	LOC645801	XM_930471
0.68	LOC645843	XM_930419

0.6	LOC645902	XM_933254
0.63	LOC645915	XM_933265
0.69	LOC645974	XM_928939
0.57	LOC646010	XM_933332
0.65	LOC646067	XM_933376
0.73	LOC646215	XM_929166
0.83	LOC646225	XM_933476
0.63	LOC646263	XM_929208
0.6	LOC646299	XM_933524
0.57	LOC646312	XM_933531
0.53	LOC646403	XM_933591
0.48	LOC646407	XM_929333
0.91	LOC646447	XM_929374
0.52	LOC646456	XM_931272
0.53	LOC646573	XM_929508
0.64	LOC646576	XM_933718
0.9	LOC646593	XM_929529
0.45	LOC646599	XM_929536
1.1	LOC646667	XM_929610
0.68	LOC646716	XM_933839
1.08	LOC646719	XM_933842
0.74	LOC646744	XM_933882
0.73	LOC646796	XM_933943
1.01	LOC646858	XM_929815
0.85	LOC646897	XM_929859
0.71	LOC646903	XM_929864
0.9	LOC646919	XM_934052
0.66	LOC647052	XM_930572
0.63	LOC647070	XM_934286
0.78	LOC647163	XM_930189
0.68	LOC647184	XM_934465
0.8	LOC647197	XM_934499
0.89	LOC647318	XM_930386
0.9	LOC652848	NM_001040077
0.6	LOC653111	XM_926073
0.9	LOC653140	XM_931416
0.72	LOC653182	XM_927250
0.63	LOC653204	XM_926469
0.54	LOC653210	XM_931919
0.69	LOC653221	XM_926536
0.62	LOC653240	XM_926590
0.57	LOC653244	XM_927424
0.79	LOC653262	XM_928310
1.47	LOC653276	XM_931495
0.6	LOC653297	XM_926730
0.74	LOC653307	XM_932962
0.89	LOC653328	XM_926913
0.72	LOC653344	XM_933147
0.5	LOC653349	XM_932849
0.83	LOC653362	XM_927072
0.54	LOC653367	XM_927090
0.54	LOC653368	XM_927095
1.02	LOC653424	XM_932020
1.04	LOC653456	XM_927471
0.53	LOC653480	XM_932187
1.02	LOC653497	XM_927733
0.63	LOC653512	XM_932333
0.66	LOC653517	XM_932358
0.78	LOC653518	XM_934555
0.5	LOC653535	XM_934842
0.5	LOC653550	XM_934853
0.53	LOC653551	XM_934856
0.44	LOC653562	XM_934879
1.04	LOC653563	XM_928111
0.65	LOC653569	XM_928141
0.7	LOC653602	XM_928353
0.73	LOC653610	XM_928387
0.73	LOC653611	XM_928391
0.52	LOC653614	XM_932857
0.51	LOC653617	XM_928425
0.85	LOC653626	XM_928461
0.78	LOC653666	XM_928775
0.56	LOC653673	XM_935258
0.5	LOC653687	XM_928916
0.62	LOC653691	XM_934808
0.63	LOC653697	XM_929041
1.05	LOC653705	XM_929072
0.54	LOC653709	XM_929105
0.52	LOC653714	XM_929171
0.52	LOC653747	XM_929393
0.45	LOC653792	XM_929910
0.51	LOC653793	XM_929919
0.99	LOC653801	XM_934188
0.44	LOC653811	XM_930073
0.6	LOC653815	XM_930112
0.49	LOC654350	XM_946378
0.85	LOC89944	NM_138342
0.74	LOC90624	NM_181705
0.71	LOC90925	NM_175870
0.84	LOC92345	NM_138386
0.62	LOC96610	NM_080926
0.54	LOH3CR2A	NM_013343
0.98	LONPL	NM_031490
0.62	LOXL2	NM_002318
0.66	LOXL4	NM_032211
0.63	LPHN2	NM_012302
0.49	LPO	NM_006151
0.65	LPPR2	NM_022737
1.02	LRCH3	NM_032773
0.54	LRFN3	NM_024509
0.48	LRP3	NM_002333
0.6	LRP4	NM_002334
0.59	LRP6	NM_002336
0.53	LRRC18	AY358137
0.82	LRRC27	NM_030626
0.59	LRRC28	NM_144598
1.04	LRRC29	NM_012163
0.54	LRRC32	NM_005512
0.88	LRRC35	NM_152715
0.59	LRRC36	NM_018296
0.68	LRRC3B	BC087849
0.66	LRRC44	NM_145258
0.86	LRRC48	NM_031294
0.85	LRRC54	NM_015516
0.59	LRRC57	NM_153260
1.19	LRTM1	BC040732
0.85	LTA4H	NM_000895
0.87	LTB4R2	NM_019839
0.51	LTBP4	AK074499
0.65	LTF	NM_002343
0.59	LTK	NM_206961
0.63	LUZP2	NM_001009909
0.92	LUZP4	NM_016383
1.26	LYK5	NM_153335
0.73	LYPD1	NM_144586
0.57	LYPD5	NM_001031749
1.11	LYSMD2	BC022075
0.7	LYZL1	NM_032517
0.68	LYZL6	NM_020426

0.56	LZTR1	NM_006767
0.7	MAB21L2	NM_006439
0.8	MAD2L1	NM_002358
0.74	MAFF	NM_012323
0.52	MAFG	NM_002359
0.54	MAGEA3	NM_005362
0.82	MAGEB1	NM_177404
0.63	MAGEB2	NM_002364
0.79	MAGEB3	BC074756
0.69	MAGED1	NM_006986
0.54	MAGED2	NM_201222
0.54	MAGED4	AF329733
0.63	MAGEE1	NM_020932
0.57	MAGEE2	NM_138703
1.13	MAK10	NM_024635
0.79	MALAT1	BX538238
0.68	MAMDC1	NM_182830
0.75	MAN1A1	NM_005907
0.89	MANEAL	NM_152496
0.95	MAP1A	NM_002373
0.76	MAP2	U89330
0.75	MAP2K1	NM_002755
0.56	MAP2K4	NM_003010
0.62	MAP2K6	NM_002758
0.57	MAP3K10	NM_002446
0.46	MAP3K14	AK131438
0.92	MAP3K7IP1	NM_153497
1.29	MAP4	NM_030884
1.16	MAP4K1	NM_007181
0.86	MAPK8IP2	NM_139124
0.75	MAPKAPK5	NM_139078
0.69	MAPRE2	NM_014268
0.65	MARCKS	NM_002356
0.63	MARK1	NM_018650
0.69	MARK2	NM_017490
0.66	MARS2	NM_138395
0.65	MARVELD3	NM_052858
0.98	MBD1	NM_015847
0.65	MBD4	NM_003925
0.6	MBL2	NM_000242
0.82	MBP	NM_002385
0.59	MBTD1	NM_017643
0.63	MCART6	NM_001012755
0.76	MCF2L	AB002360
0.62	MCFP	NM_018843
0.48	MCHR1	NM_005297
0.71	MCOLN1	NM_020533
1.14	MCRS1	NM_006337
0.54	MCTP1	NM_001002796
0.85	MCTP2	BC041387
0.5	MCTS1	NM_014060
0.62	MDM2	U33203
0.54	ME3	NM_006680
0.6	MED12	U80742
0.56	MEF2A	Y16312
0.64	MEF2C	NM_002397
0.83	MEFV	NM_000243
0.82	MEGF11	NM_032445
0.6	MEGF8	NM_001410
0.72	MEIS2	AY349358
0.65	MESDC2	NM_015154
0.62	MEST	NM_177524
0.46	METT10D	NM_024086
0.66	METT5D1	NM_152636
0.68	MFI2	NM_033316
0.65	MGAM	NM_004668
0.52	MGAT2	NM_002408
0.59	MGAT5B	BC063862
0.75	MGC11257	NM_032350
0.6	MGC11266	NM_024322
0.67	MGC13034	AK055830
0.51	MGC13170	BC051842
0.85	MGC14425	XM_928066
0.54	MGC15476	NM_145056
0.74	MGC15875	NM_153373
0.5	MGC16186	BC007374
0.63	MGC16291	NM_032770
0.65	MGC16384	BC009492
0.59	MGC16385	NM_145039
0.69	MGC17403	XM_933483
0.87	MGC23985	NM_206966
0.75	MGC24039	AK095598
0.65	MGC26647	NM_152706
0.66	MGC26733	NM_144992
0.77	MGC2749	NM_024069
0.65	MGC2752	NM_023939
0.81	MGC33648	NM_153706
1.36	MGC34796	BC034822
0.86	MGC35048	BC034823
0.52	MGC35361	NM_147194
0.48	MGC35402	NM_203307
0.59	MGC39497	NM_152436
0.96	MGC39518	NM_173822
1.32	MGC40069	NM_182615
0.49	MGC4172	NM_024308
0.72	MGC42090	NM_152774
0.68	MGC45438	NM_152459
0.59	MGC4562	NM_133375
0.71	MGC46336	XM_290712
0.53	MGC4655	NM_033309
0.52	MGC50559	NM_173802
0.68	MGC51025	NM_178571
0.63	MGC52000	BC106020
0.92	MGC62100	NM_206894
0.57	MGC70924	NM_199285
0.56	MGC71993	NM_001004333
0.49	MGC72104	NM_207350
0.74	MGC88374	NM_001004331
0.75	MGP	NM_000900
0.49	MGST1	NM_145792
0.77	MICAL3	XM_931108
0.62	MICAL-L2	NM_182924
0.76	MID2	BC017707
0.62	MIF4GD	NM_020679
0.59	MINK1	AL157418
0.5	MINPP1	NM_004897
0.59	MIPEP	NM_005932
0.68	MIS12	NM_024039
0.65	MITF	NM_198177
0.63	MKI67	X65551
0.65	MKKS	NM_018848
0.71	MKL2	NM_014048
0.62	MKS1	NM_017777
0.55	MLL2	NM_003482
0.54	MLL4	NM_014727
0.65	MLLT6	NM_005937
0.65	MLSTD1	AK001324
1.32	MMP1	NM_002421
0.85	MMP12	NM_002426

0.5	MMP13	NM_002427
0.47	MMP2	NM_004530
0.88	MMP20	NM_004771
0.65	MMP24	NM_006690
0.66	MMP27	NM_022122
0.73	MMP3	NM_002422
0.92	MMP9	NM_004994
0.65	MNAB	BC044642
0.68	MNAT1	NM_002431
0.68	MOCS1	BC036839
0.6	MORF4L2	NM_012286
0.71	MPHOSPH6	NM_005792
0.52	MPHOSPH9	NM_022782
0.76	MPP1	NM_002436
0.7	MPP7	NM_173496
0.63	MPPED2	NM_001584
0.69	MRGPRD	NM_198923
0.6	MRGPRX2	NM_054030
0.73	MRGPRX3	NM_054031
0.91	MRPL21	NM_181515
0.59	MRPL40	NM_003776
0.62	MRPL42	NM_172178
0.59	MRPL46	NM_022163
0.47	MRPL48	BC036501
0.73	MRPL49	NM_004927
0.85	MRPL51	NM_016497
0.59	MRPS11	NM_176805
0.65	MRPS28	NM_014018
0.72	MRPS35	NM_021821
0.62	MRPS6	BC042752
0.57	MRVI1	NM_130385
0.79	MS4A10	NM_206893
0.85	MS4A3	NM_006138
0.54	MS4A6A	NM_152852
1.07	MS4A8B	NM_031457
0.85	MSI1	NM_002442
0.71	MSI2	NM_170721
0.68	MSL2L1	NM_018133
0.47	MSLN	NM_013404
0.5	MSMB	NM_138634
0.85	MSR1	NM_138716
0.45	MT	NM_173467
0.65	MT1B	NM_005947
0.77	MT1JP	NM_175622
1.01	MT1M	NM_176870
0.98	MT3	NM_005954
0.5	MT4	NM_032935
0.65	MTAP	NM_002451
1.04	MTCP1	NM_001018025
0.75	MTFMT	NM_139242
0.72	MTG1	NM_138384
0.72	MTIF3	NM_152912
0.57	MTL5	NM_004923
0.5	MTM	NM_175620
0.63	MTMR1	BC011250
0.71	MTMR4	NM_004687
0.92	MTMR8	NM_017677
0.75	MTRF1L	NM_019041
0.85	MTSS1	AB007889
0.63	MUC15	NM_145650
0.68	MUC4	NM_138299
1.04	MUS81	NM_025128
0.52	MVK	NM_000431
0.63	MYADML	NM_207329
0.69	MYF6	NM_002469
0.64	MYH6	NM_002471
0.77	MYH8	NM_002472
0.8	MYL1	NM_079420
0.72	MYL7	NM_021223
0.59	MYO15A	BX538062
0.62	MYO1A	NM_005379
0.96	MYO1B	AK022489
1.13	MYO9B	NM_004145
0.74	MYOCD	NM_153604
0.59	MYOHD1	NM_025109
0.52	MYT1	NM_004535
0.59	N/A	NM_138396
0.83	N/A	NM_006640
0.86	N/A	NM_203341
0.98	N/A	AB003177
0.47	N/A	AB040413
0.69	N/A	AB046614
0.47	N/A	AB055227
0.78	N/A	AB062477
0.49	N/A	AB096950
0.49	N/A	AB180042
0.77	N/A	AB180043
0.57	N/A	AB231740
0.52	N/A	AF023266
0.62	N/A	AF090898
0.59	N/A	AF090926
0.48	N/A	AF090927
0.48	N/A	AF090934
0.65	N/A	AF109190
0.54	N/A	AF116603
0.6	N/A	AF116611
0.72	N/A	AF116661
0.63	N/A	AF116668
0.72	N/A	AF116673
0.62	N/A	AF118069
0.53	N/A	AF119881
0.68	N/A	AF119886
0.77	N/A	AF119888
1.38	N/A	AF119912
0.78	N/A	AF130057
0.59	N/A	AF130083
0.58	N/A	AF130114
0.83	N/A	AF132201
0.82	N/A	AF132203
1.45	N/A	AF167994
0.68	N/A	AF190155
0.76	N/A	AF193050
0.5	N/A	AF204269
0.52	N/A	AF218021
0.47	N/A	AF220234
0.63	N/A	AF251047
1.04	N/A	AF258580
0.46	N/A	AF271776
1.26	N/A	AF274942
0.71	N/A	AF281279
0.56	N/A	AF289615
0.85	N/A	AF305820
0.65	N/A	AF336875
0.75	N/A	AF338194
0.72	N/A	AF370375
0.62	N/A	AF384996
0.62	N/A	AF385324
0.56	N/A	AF428135
0.64	N/A	AF494508

0.63	N/A	AF495727
0.53	N/A	AJ237663
0.68	N/A	AJ271747
0.63	N/A	AJ567757
0.59	N/A	AK000013
0.92	N/A	AK000357
0.74	N/A	AK000815
0.82	N/A	AK001176
1.17	N/A	AK001979
1.07	N/A	AK021432
0.87	N/A	AK023121
0.73	N/A	AK024190
0.63	N/A	AK025278
1.13	N/A	AK027082
0.84	N/A	AK027667
0.75	N/A	AK054879
0.74	N/A	AK055082
0.5	N/A	AK055679
0.9	N/A	AK056055
0.53	N/A	AK056283
0.68	N/A	AK056771
0.49	N/A	AK056971
1.19	N/A	AK057318
0.5	N/A	AK057352
0.72	N/A	AK058065
0.59	N/A	AK074369
0.64	N/A	AK091409
0.62	N/A	AK091555
0.6	N/A	AK092094
1.05	N/A	AK092594
0.56	N/A	AK092698
0.71	N/A	AK092964
0.73	N/A	AK093927
0.45	N/A	AK094244
0.49	N/A	AK094481
0.52	N/A	AK094571
0.57	N/A	AK094707
0.7	N/A	AK094743
0.52	N/A	AK094860
0.62	N/A	AK095032
0.76	N/A	AK095260
0.56	N/A	AK095410
1.15	N/A	AK095925
0.63	N/A	AK095945
0.6	N/A	AK096041
0.85	N/A	AK096721
0.6	N/A	AK096849
0.51	N/A	AK096917
0.54	N/A	AK096981
0.54	N/A	AK097222
0.46	N/A	AK097322
0.62	N/A	AK097637
0.46	N/A	AK097908
0.69	N/A	AK097924
0.77	N/A	AK098126
0.75	N/A	AK098414
1.04	N/A	AK098695
0.54	N/A	AK098761
0.75	N/A	AK122798
0.6	N/A	AK123323
0.7	N/A	AK123349
0.48	N/A	AK123383
0.86	N/A	AK123477
0.83	N/A	AK123491
0.53	N/A	AK123537
0.86	N/A	AK123615
0.82	N/A	AK123661
0.52	N/A	AK123704
0.65	N/A	AK123755
0.77	N/A	AK123839
0.65	N/A	AK123843
0.86	N/A	AK123848
0.48	N/A	AK123899
0.82	N/A	AK123920
0.77	N/A	AK124002
0.71	N/A	AK124181
0.59	N/A	AK124182
0.75	N/A	AK124200
0.62	N/A	AK124310
0.72	N/A	AK124359
0.71	N/A	AK124361
1.26	N/A	AK124483
0.68	N/A	AK124622
0.62	N/A	AK124631
0.51	N/A	AK124658
0.69	N/A	AK124722
0.69	N/A	AK124794
0.59	N/A	AK124820
0.86	N/A	AK124862
0.56	N/A	AK124933
1.08	N/A	AK125000
0.54	N/A	AK125136
0.63	N/A	AK125187
0.56	N/A	AK125338
0.56	N/A	AK125436
0.68	N/A	AK125549
0.73	N/A	AK125674
0.47	N/A	AK125684
1.33	N/A	AK125698
0.56	N/A	AK125712
0.62	N/A	AK125733
0.6	N/A	AK125829
0.72	N/A	AK125937
0.87	N/A	AK125949
0.98	N/A	AK126001
0.65	N/A	AK126029
0.55	N/A	AK126221
0.71	N/A	AK126237
0.65	N/A	AK126260
0.48	N/A	AK126297
0.59	N/A	AK126398
0.85	N/A	AK126528
0.53	N/A	AK126601
0.75	N/A	AK126633
0.63	N/A	AK126637
0.63	N/A	AK126643
0.45	N/A	AK126764
0.88	N/A	AK126796
0.54	N/A	AK126895
0.71	N/A	AK126948
0.59	N/A	AK126977
0.68	N/A	AK127032
0.71	N/A	AK127088
0.83	N/A	AK127099
1.2	N/A	AK127121
0.91	N/A	AK127229
0.63	N/A	AK127356
0.6	N/A	AK127359
0.5	N/A	AK127393

0.46	N/A	AK127421
0.49	N/A	AK127423
0.77	N/A	AK127479
0.82	N/A	AK127486
0.65	N/A	AK127507
0.46	N/A	AK127532
0.68	N/A	AK127534
0.95	N/A	AK127696
0.55	N/A	AK128046
0.49	N/A	AK128082
0.69	N/A	AK128146
0.5	N/A	AK128181
0.52	N/A	AK128397
0.76	N/A	AK128441
1.11	N/A	AK128535
0.83	N/A	AK128554
0.68	N/A	AK128685
1.01	N/A	AK128697
0.92	N/A	AK128722
0.45	N/A	AK128779
0.49	N/A	AK128800
1.01	N/A	AK128825
0.82	N/A	AK129559
0.49	N/A	AK129612
0.59	N/A	AK129619
0.56	N/A	AK129643
0.89	N/A	AK130016
0.67	N/A	AK130051
0.66	N/A	AK130276
0.49	N/A	AK130544
0.57	N/A	AK130638
0.69	N/A	AK130723
0.77	N/A	AK130744
0.52	N/A	AK130766
0.57	N/A	AK130852
1.01	N/A	AK131291
0.74	N/A	AK131343
0.68	N/A	AK131364
0.6	N/A	AK131383
0.52	N/A	AK131442
0.62	N/A	AK131479
0.49	N/A	AK131496
0.5	N/A	AK131510
0.74	N/A	AK131515
0.78	N/A	AK172759
0.84	N/A	AK172805
0.64	N/A	AL137737
0.68	N/A	AL831885
0.63	N/A	AX751581
0.68	N/A	AX772826
1.04	N/A	AX773938
1.22	N/A	AX775863
0.56	N/A	AX775949
0.85	N/A	AX776298
0.59	N/A	AX800072
0.62	N/A	AY129018
1.01	N/A	AY234408
0.46	N/A	AY358148
0.98	N/A	AY358194
0.65	N/A	AY358233
0.91	N/A	AY358248
0.68	N/A	AY358260
0.75	N/A	AY358410
0.66	N/A	AY358798
0.99	N/A	AY501002
0.66	N/A	BC004969
0.48	N/A	BC012026
0.59	N/A	BC012036
0.89	N/A	BC016673
0.81	N/A	BC024198
0.51	N/A	BC026081
0.75	N/A	BC030712
0.59	N/A	BC030789
0.59	N/A	BC031827
0.73	N/A	BC034966
0.9	N/A	BC035666
0.92	N/A	BC040330
0.85	N/A	BC040331
0.53	N/A	BC041884
0.73	N/A	BC042003
0.77	N/A	BC045622
0.71	N/A	BC046415
0.59	N/A	BC047782
0.83	N/A	BC050093
0.62	N/A	BC051709
0.56	N/A	BC052626
0.58	N/A	BC053984
0.45	N/A	BC054893
0.65	N/A	BC057999
0.73	N/A	BC058003
0.91	N/A	BC058837
0.75	N/A	BC062673
1.34	N/A	BC062751
0.71	N/A	BC063453
0.62	N/A	BC063881
0.72	N/A	BC065260
0.51	N/A	BC066878
0.74	N/A	BC067077
0.85	N/A	BC069659
0.86	N/A	BC070274
0.5	N/A	BC071744
0.87	N/A	BC073765
0.97	N/A	BC073778
0.45	N/A	BC073795
0.74	N/A	BC080530
0.55	N/A	BC080549
0.53	N/A	BC096733
0.62	N/A	BC100779
1.16	N/A	BC101331
0.44	N/A	BC101435
0.66	N/A	BC105287
0.5	N/A	BC106056
0.82	N/A	BC107149
0.65	N/A	BC107743
0.93	N/A	BC110642
0.68	N/A	BC110821
0.98	N/A	BC112395
0.58	N/A	BC114217
0.73	N/A	BC114375
0.53	N/A	BC114429
0.72	N/A	BC114505
0.8	N/A	BX161483
0.84	N/A	BX161511
0.46	N/A	BX647504
0.72	N/A	BX648788
1.2	N/A	CR593166
0.5	N/A	CR594675
0.51	N/A	CR602756
0.76	N/A	CR606408

0.56	N/A	CR611063
0.62	N/A	CR625101
1.01	N/A	CR625565
0.6	N/A	CR627384
0.72	N/A	L10374
0.46	N/A	L38933
0.47	N/A	M15530
1.26	N/A	NM_001040053
0.5	N/A	NM_173524
0.52	N/A	NM_173613
1.04	N/A	X57805
0.45	N/A	X57815
0.59	N/A	X57820
0.71	NAGA	NM_000262
0.62	NAGPA	NM_016256
0.45	NALP11	NM_145007
0.66	NALP14	NM_176822
0.45	NALP2	AF269288
1.1	NALP4	NM_134444
0.74	NANOS2	NM_001029861
1.04	NAP1L2	NM_021963
0.51	NAPA	NM_003827
0.75	NAPE-PLD	NM_198990
0.98	NAPG	NM_003826
0.62	NAPSA	AK055569
0.99	NARF	NM_031968
0.52	NARG2	BC013684
0.66	NARS2	NM_024678
0.59	NAT5	NM_181528
0.83	NAV1	NM_020443
0.82	NAV2	BC016054
0.78	NAV3	NM_014903
0.95	NBEA	NM_015678
0.46	NBR1	NM_031862
0.52	NBR2	NM_005821
0.92	NCF4	NM_013416
0.8	NCK2	NM_003581
0.52	NCKAP1L	NM_005337
0.65	NCOA2	NM_006540
1.23	NCOR1	AF303585
0.51	NCR1	NM_004829
0.56	NDEL1	AK123802
0.64	NDFIP1	NM_030571
1.15	NDFIP2	NM_019080
0.49	NDRG3	NM_032013
0.77	NDRG4	NM_020465
0.51	NDUFA3	NM_004542
0.52	NDUFA9	NM_005002
0.96	NDUFB3	NM_002491
0.74	NDUFB6	NM_182739
0.48	NDUFB7	NM_004146
0.54	NDUFC2	NM_004549
0.67	NDUFS6	NM_004553
0.72	NDUFV2	NM_021074
0.93	NEBL	NM_213569
0.52	NEDD4L	AY112985
0.71	NEDD8	NM_006156
0.53	NEFH	NM_021076
0.49	NEK3	AK131359
0.59	NEK5	NM_199289
0.59	NELL2	NM_006159
0.65	NETO1	NM_153181
0.55	NEURL2	NM_080749
0.63	NEXN	NM_144573
0.55	NFATC2	NM_173091
0.68	NFATC2IP	NM_032815
0.69	NFE2	NM_006163
0.62	NFE2L1	NM_003204
1.18	NFE2L3	AF125534
0.54	NFIC	NM_005597
0.79	NFKB2	NM_002502
0.86	NFKBIB	NM_002503
0.84	NFX1	BC006447
0.9	NFXL1	NM_152995
0.78	NFYA	BC039244
0.52	NGFR	NM_002507
1.16	NHLRC2	NM_198514
0.45	NHP2L1	NM_001003796
0.54	NHS	NM_198270
0.59	NIFUN	NM_213595
1.35	NIN	NM_016350
1.21	NIP30	NM_024946
0.83	NIP7	NM_016101
0.53	NKX2-3	NM_145285
0.72	NKX3-1	NM_006167
1.5	NLF1	NM_207322
0.6	NLGN3	NM_018977
1.04	NLGN4X	NM_181332
0.65	NMB	NM_205858
0.65	NME2	NM_002512
0.82	NME6	NM_005793
0.67	NMU	NM_006681
0.69	NNMT	NM_006169
0.76	NOC3L	NM_022451
0.49	NOL11	NM_015462
1.07	NOL6	NM_139235
0.62	NOLA3	NM_018648
0.5	NOLC1	NM_004741
0.53	NOMO2	AL512687
1.22	NOP17	NM_017916
0.72	NOS1	NM_000620
0.71	NOVA2	NM_002516
0.63	NOX1	NM_013955
0.48	NPAS1	NM_002517
0.52	NPAS3	NM_173159
0.59	NPC1	NM_000271
0.52	NPC2	NM_006432
0.5	NPIP	AF132984
0.73	NPL	NM_030769
0.79	NPM3	NM_006993
1.5	NPTN	NM_017455
0.67	NPY5R	U56079
0.47	NR0B1	NM_000475
0.73	NR1H3	CR618031
0.83	NR1I3	NM_005122
0.55	NR4A1	NM_173157
0.79	NRAP	NM_198060
0.66	NRD1	X93207
0.65	NRG1	NM_013964
0.71	NRL	BC012395
0.49	NSFL1C	NM_182483
1.37	NSMAF	NM_003580
1.39	NSMCE1	NM_145080
0.59	NT5C3	NM_016489
0.77	NUBPL	NM_025152
0.57	NUDT13	NM_015901
0.62	NUDT2	U30313
0.95	NUDT21	NM_007006
0.57	NUDT6	NM_198041

0.6	NUDT9P1	BC029544
0.53	NUFIP1	NM_012345
0.62	NULP1	NM_014972
0.52	NUMB	BC033824
0.81	NUP153	NM_005124
0.7	NUP54	NM_017426
0.52	NUSAP1	NM_018454
0.62	NUT	NM_175741
1.18	NUTF2	NM_005796
0.76	NXF1	NM_006362
0.5	NXF2	NM_017809
0.66	NXF4	AK124700
0.73	NXT2	NM_018698
0.89	NYD-SP11	XM_371164
0.68	NY-SAR-48	NM_033417
0.63	OBSL1	AK023854
0.66	ODF2L	NM_020729
0.63	ODZ1	NM_014253
0.68	OFCC1	NM_153003
0.6	OGDHL	NM_018245
0.47	OGFOD1	NM_001031707
0.58	OGN	NM_033014
0.67	OK/SW-CL.89	XM_926087
0.68	OKL38	NM_182980
0.73	OLFM3	NM_058170
0.52	OLR1	NM_002543
0.78	OPA1	NM_130837
0.88	OPCML	NM_001012393
0.63	OR10A4	NM_207186
0.54	OR10A6	NM_001004461
0.91	OR10G8	NM_001004464
0.8	OR10J1	NM_012351
0.6	OR10W1	NM_207374
0.78	OR12D2	NM_013936
0.98	OR13A1	BC104855
0.59	OR1A1	NM_014565
0.49	OR1A2	NM_012352
0.83	OR1C1	NM_012353
0.86	OR1D2	NM_002548
0.56	OR1D5	NM_014566
0.8	OR1F1	Y14442
0.62	OR2A5	NM_012365
0.5	OR2AG1	NM_001004489
0.69	OR2AG2	NM_001004490
0.68	OR2J2	NM_030905
0.83	OR2T1	NM_030904
0.86	OR2T10	NM_001004693
0.83	OR2T5	NM_001004697
0.91	OR2Y1	NM_001001657
0.59	OR3A3	NM_012373
0.49	OR3A4	NM_001005334
0.82	OR4A16	NM_001005274
1.01	OR4A47	NM_001005512
0.91	OR4B1	NM_001005470
0.6	OR4C13	NM_001001955
0.6	OR4C16	NM_001004701
0.66	OR4C3	NM_001004702
0.6	OR4C46	NM_001004703
0.65	OR4D1	NM_012374
0.6	OR4D10	NM_001004705
0.52	OR4F6	NM_001005326
0.52	OR4K14	NM_001004712
0.58	OR4K2	NM_001005501
0.61	OR4M1	NM_001005500
0.69	OR4M2	NM_001004719
0.52	OR4N4	AK097598
0.61	OR4N5	NM_001004724
0.82	OR4P4	NM_001004124
0.73	OR51A7	NM_001004749
0.79	OR51B6	NM_001004750
0.73	OR51E1	BC022401
0.5	OR51E2	NM_030774
0.82	OR51L1	NM_001004755
0.5	OR51S1	NM_001004758
0.73	OR51T1	NM_001004759
0.73	OR52A1	NM_012375
0.88	OR52B4	NM_001005161
0.63	OR52B6	NM_001005162
0.47	OR52E4	NM_001005165
0.66	OR52E6	NM_001005167
0.5	OR52E8	NM_001005168
0.6	OR52I2	NM_001005170
0.5	OR52N2	NM_001005174
0.88	OR52N4	NM_001005175
0.79	OR52W1	NM_001005178
0.85	OR56A1	NM_001001917
0.54	OR56A3	NM_001003443
0.63	OR56B1	NM_001005180
1.1	OR5A1	NM_001004728
0.5	OR5AK2	NM_001005323
0.69	OR5AP2	NM_001002925
0.47	OR5AR1	NM_001004730
0.69	OR5B12	NM_001004733
1.2	OR5B2	NM_001005566
0.63	OR5BU1	NM_001004734
0.85	OR5D16	NM_001005496
0.71	OR5H6	NM_001005479
0.6	OR5L1	NM_001004738
0.54	OR5M1	NM_001004740
0.54	OR5M11	NM_001005245
0.76	OR5P3	NM_153445
0.75	OR5U1	NM_030946
1.04	OR6A2	NM_003696
0.75	OR6C4	NM_001005494
0.71	OR6S1	NM_001001968
1.08	OR6V1	NM_001001667
1.1	OR6X1	NM_001005188
0.48	OR7A5	NM_017506
0.6	OR7C2	NM_012377
0.54	OR7D2	NM_175883
0.54	OR7D4	NM_001005191
0.66	OR8B12	NM_001005195
0.66	OR8B8	NM_012378
0.5	OR8D1	NM_001002917
0.54	OR8D2	NM_001002918
0.76	OR8H1	NM_001005199
0.73	OR8H2	NM_001005200
0.73	OR8H3	NM_001005201
0.79	OR8K1	NM_001002907
0.66	OR8U1	NM_001005204
0.66	OR8U8	NM_001013356
0.5	OR9G4	NM_001005284
1.01	OR9I1	NM_001005211
0.69	OR9Q1	NM_001005212
0.8	ORC2L	NM_006190
0.55	ORMDL2	NM_014182
0.65	ORMDL3	NM_139280
0.64	OSAP	NM_032623
0.79	OSBPL1A	NM_018030

0.8	OSGEP	NM_017807
0.63	OSGEPL1	NM_022353
0.59	OSTbeta	NM_178859
0.95	OTC	NM_000531
0.61	OTUB2	NM_023112
0.69	OTUD5	NM_017602
0.71	OTX2	NM_172337
0.82	OVCH1	NM_183378
0.54	OVCH2	NM_198185
1.05	OXGR1	NM_080818
0.68	OXTR	NM_000916
0.75	P11	NM_006025
0.65	P15RS	NM_018170
1.42	P2RX3	NM_002559
0.65	P2RY13	NM_176894
0.53	P2RY5	BC045651
0.57	P2RY6	NM_176798
0.69	P4HA1	NM_001017962
1.29	P4HB	BC029617
0.5	PABPC5	NM_080832
1.09	PABPN1	NM_004643
0.6	PACS1	NM_018026
0.95	PAFAH1B3	NM_002573
0.75	PAH	NM_000277
0.47	PAK1	NM_002576
1.22	PAK4	NM_005884
0.72	PANK1	NM_148978
0.46	PANK2	XM_926491
0.63	PANX1	NM_015368
0.7	PAP2D	NM_001037317
0.91	PAPPA	NM_002581
0.57	PAPSS2	NM_004670
0.92	PAQR5	NM_017705
0.47	PARG	NM_003631
0.95	PARP11	NM_020367
0.75	PARP15	NM_152615
0.65	PARP3	NM_005485
0.82	PARP6	NM_020213
0.63	PASK	NM_015148
0.6	PATE	NM_138294
0.82	PAWR	NM_002583
0.8	PAX1	NM_006192
0.8	PAX3	NM_181461
0.82	PAX4	NM_006193
1.35	PCBD1	NM_001001939
0.71	PCBP3	NM_020528
0.56	PCDH11Y	NM_032971
0.57	PCDH15	AY388963
0.59	PCDH17	NM_014459
0.59	PCDH20	NM_022843
0.86	PCDH9	NM_203487
0.64	PCDHA11	NM_031861
0.64	PCDHA3	NM_031497
0.74	PCDHA4	NM_031500
0.74	PCDHB12	NM_018932
0.71	PCDHB13	NM_018933
0.81	PCDHB14	NM_018934
0.74	PCDHB6	NM_018939
0.6	PCDHGA10	NM_032090
1.01	PCDHGA3	NM_032011
0.71	PCDHGA5	NM_032054
0.71	PCDHGB2	NM_032096
0.6	PCDHGB7	NM_032101
0.6	PCF11	NM_015885
0.77	PCGF2	NM_007144
1.01	PCGF5	BC051845
0.6	PCID1	NM_006360
0.79	PCID2	BC008975
0.55	PCK2	NM_004563
1.51	PCMTD2	BC033665
0.52	PCNA	NM_182649
1.19	PCNXL2	NM_024938
0.71	PCOLN3	NM_002768
1.23	PCTK1	NM_033018
0.56	PCTP	NM_021213
0.53	PDCD11	NM_014976
0.98	PDCD8	NM_145813
0.77	PDCL2	NM_152401
0.7	PDE1A	NM_005019
1.11	PDE1B	AJ401609
0.88	PDE2A	AY495087
0.74	PDE6A	NM_000440
0.62	PDE6H	NM_006205
0.75	PDE7A	NM_002603
0.62	PDE8A	NM_173457
0.75	PDGFRL	NM_006207
0.56	PDPK2	AJ785968
0.73	PDPN	NM_001006625
1.04	PDPR	NM_017990
0.69	PDZD3	NM_024791
0.65	PDZRN4	NM_013377
0.65	PECAM1	NM_000442
0.57	PEG3	NM_006210
0.61	PELI2	NM_021255
0.77	PELO	NM_015946
1.2	PELP1	NM_014389
0.56	PEMT	NM_148172
0.5	PEO1	NM_021830
0.6	PEPP-2	NM_032498
0.51	PEX26	NM_017929
1.11	PEX5	Z48054
0.78	PEX6	NM_000287
0.99	PFAS	NM_012393
0.87	PFDN1	NM_002622
0.59	PFDN5	NM_145897
1.08	PFKL	NM_002626
0.88	PFKM	BX537703
0.73	PGAP1	NM_024989
0.63	PGBD3	BC034479
0.75	PGK2	NM_138733
0.76	PGM1	NM_002633
0.76	PHACS	NM_032592
0.49	PHACTR3	NM_183246
1.06	PHC2	NM_198040
0.59	PHF11	NM_016119
0.7	PHF13	NM_153812
0.65	PHF20	AK090798
0.8	PHF21B	NM_138415
0.65	PHF23	BC002509
0.57	PHF8	NM_015107
0.5	PHKA1	NM_002637
0.5	PHKA2	NM_000292
0.47	PHKB	NM_001031835
0.72	PHLDA1	NM_007350
0.83	PHLDB3	NM_198850
0.5	PHOX2A	NM_005169
0.49	PIAS1	NM_016166
0.52	PIAS2	NM_173206
0.63	PIGF	NM_173074

0.73	PIGM	NM_145167
0.65	PIGS	NM_033198
0.59	PIGW	NM_178517
0.95	PIK3C2A	NM_002645
1.28	PIK3C3	NM_002647
0.65	PIK3CA	NM_006218
0.82	PIK3CG	NM_002649
0.83	PIK4CA	AB210002
0.57	PIM2	NM_006875
0.6	PIN4	NM_006223
1.19	PIP5K1C	NM_012398
0.49	PIP5K2C	NM_024779
0.65	PITPNB	NM_012399
1.23	PITPNM1	NM_004910
0.56	PITPNM3	NM_031220
0.76	PITX3	NM_005029
0.65	PIWIL2	NM_018068
0.66	PJA1	NM_145119
0.53	PKD1L3	NM_181536
0.85	PKIB	AK074397
1.41	PKM2	NM_182471
0.6	PLAC1	NM_021796
0.49	PLAC4	NM_182832
0.74	PLAC8	BC012205
0.65	PLAG1	BC075047
0.77	PLAGL2	NM_002657
0.72	PLCE1	AB040949
0.45	PLD3	NM_001031696
0.57	PLEKHA1	NM_001001974
0.65	PLEKHA9	NM_015899
0.45	PLEKHG2	NM_022835
0.75	PLEKHG6	NM_018173
0.65	PLEKHQ1	NM_025201
0.74	PLK2	NM_006622
1.04	PLK4	NM_014264
0.56	PLXDC2	NM_032812
0.59	PLXNB2	AB002313
0.62	PLXNC1	NM_005761
0.82	PML	AF370432
0.47	PMM2	NM_000303
0.47	PMP22CD	NM_001013743
0.82	PNMA3	NM_013364
0.59	PNPO	NM_018129
0.5	POF1B	NM_024921
0.55	POFUT1	NM_172236
0.56	POFUT2	NM_133635
1.04	POLB	NM_002690
0.68	POLDIP2	NM_015584
0.59	POLDIP3	NM_178136
0.65	POLG2	NM_007215
0.77	POLK	BC050718
0.73	POLR2G	NM_002696
0.86	POLR2I	NM_006233
0.82	POLR3A	BC014399
0.82	POLR3B	NM_018082
0.95	POLR3F	NM_006466
0.77	POMT2	NM_013382
0.45	POP4	NM_006627
0.78	POP5	NM_198202
0.92	POSTN	NM_006475
0.91	POT1	NM_015450
0.89	PPAP2C	NM_177543
0.62	PPARA	XM_926186
0.95	PPARG	NM_015869
0.77	PPEF2	NM_152934
0.72	PPFIBP1	NM_177444
0.85	PPFIBP2	NM_003621
0.55	PPGB	NM_000308
0.59	PPHLN1	NM_201515
0.59	PPM1H	XM_350880
0.78	PPM1L	NM_139245
0.98	PPM2C	BC098343
0.76	PPME1	AK123288
0.69	PPP1CC	NM_002710
0.59	PPP1R12A	NM_002480
0.57	PPP1R13L	NM_006663
1.07	PPP1R14A	NM_033256
0.62	PPP1R3A	NM_002711
0.68	PPP1R3B	NM_024607
0.57	PPP1R3C	NM_005398
0.71	PPP1R3D	NM_006242
0.6	PPP1R3F	NM_033215
0.63	PPP1R7	NM_002712
0.5	PPP2R2D	BC072402
0.78	PPP2R5D	NM_180977
0.72	PPP3CB	NM_021132
0.63	PPP3R1	NM_000945
0.6	PQBP1	NM_005710
0.52	PRAC	NM_032391
0.69	PRAF2	NM_007213
0.59	PRB1	NM_199354
0.59	PRB2	NM_006248
0.79	PRC1	NM_199414
0.91	PRCP	NM_199418
0.71	PRDM1	NM_182907
0.5	PRDM10	NM_199437
0.79	PRDM11	NM_020229
0.62	PRDM15	AF426259
0.7	PREPL	DQ023507
1.26	PRF1	NM_005041
0.63	PRG1	NM_002727
0.63	PRG3	NM_006093
0.75	PRH2	NM_005042
0.81	PRIM2A	NM_000947
0.65	PRKAB1	NM_006253
0.51	PRKACA	NM_207518
0.77	PRKCB1	NM_212535
0.99	PRKCBP1	DQ082998
0.47	PRKCDBP	NM_145040
0.68	PRKCG	NM_002739
0.52	PRKD1	NM_002742
0.8	PRKD3	NM_005813
0.82	PRKRIP1	NM_024653
0.95	PRMT8	AK026786
0.71	PRNT	NM_177549
0.44	PRO0149	NM_014117
0.56	PRO0478	AF090930
0.77	ProSAPiP1	BC038860
1.04	PROSC	NM_007198
0.67	PRP2	BC035310
0.63	PRPF38B	NM_018061
0.5	PRPS1	NM_002764
0.78	PRPS1L1	NM_175886
0.6	PRR10	NM_173579
0.59	PRR11	NM_018304
0.62	PRR14	NM_024031
0.78	PRR4	NM_007244
1.17	PRRG3	NM_024082
0.77	PRSS12	NM_003619

0.47	PRY	AF517635
0.47	PRY2	NM_001002758
0.63	PSCDBP	NM_004288
0.89	PSENEN	AF162447
0.51	PSG5	NM_002781
0.48	PSG7	NM_002783
0.71	PSMA6	NM_002791
0.77	PSMB3	NM_002795
0.48	PSMC1	NM_002802
0.46	PSMC3IP	NM_016556
0.65	PSMC5	NM_002805
0.61	PSMC6	NM_002806
0.69	PSMD13	NM_175932
0.63	PSMD14	NM_005805
0.5	PSMD4P2	BC068549
0.98	PSMD9	NM_002813
2.32	PSME2	NM_002818
0.63	PTCHD1	NM_173495
0.61	PTGDR	NM_000953
1.1	PTGIR	NM_000960
1.2	PTGIS	NM_000961
0.76	PTGS2	NM_000963
0.49	PTHLH	NM_198966
0.72	PTK2	BC028733
0.85	PTK2B	NM_173174
0.55	PTK9	NM_198974
0.68	PTOV1	NM_017432
0.78	PTPDC1	NM_177995
0.66	PTPN20A	XM_931660
0.66	PTPN20B	BC093750
0.66	PTPN22	NM_015967
0.63	PTPRE	NM_130435
0.56	PTPRM	NM_002845
0.82	PTPRO	NM_030667
0.93	PTRH2	NM_016077
0.65	PURG	NM_013357
0.62	PUS7	NM_019042
0.78	PUS7L	NM_031292
0.51	PVRL3	NM_015480
0.75	PWP1	NM_007062
0.95	PXMP4	NM_183397
0.82	PZP	NM_002864
0.57	QPCTL	NM_017659
0.78	RAB23	NM_183227
0.52	RAB27A	NM_183235
0.85	RAB27B	NM_004163
0.73	RAB30	NM_014488
0.98	RAB33A	NM_004794
0.66	RAB38	NM_022337
0.91	RAB39	NM_017516
0.66	RAB3IL1	NM_013401
1.04	RAB43	BC082988
1.04	RAB6IP1	NM_015213
0.76	RABGAP1L	AY364260
0.63	RABGGTB	NM_004582
0.99	RABL3	NM_173825
0.48	RABL4	NM_006860
1.57	RAD51AP1	NM_006479
1.17	RAD51C	NM_058217
0.55	RAD51L1	NM_133510
0.5	RAG1	BC037344
0.64	RAGE	BC043179
0.82	RAI17	NM_020338
0.62	RALA	NM_005402
0.86	RALY	NM_016732
1.01	RANBP17	AK027880
0.71	RANBP9	BC063849
0.74	RAP1GDS1	NM_021159
0.56	RAP2A	NM_021033
0.6	RAP2C	NM_021183
0.69	RAPGEF3	NM_006105
0.77	RAPGEF6	AK074318
0.52	RARA	NM_000964
1.11	RARG	BC093727
0.5	RARRES3	NM_004585
0.88	RASA2	NM_006506
0.52	RASAL1	NM_004658
0.57	RASGRP4	NM_170604
0.52	RASSF2	NM_170773
0.6	RAVER1	NM_133452
1.08	RAX	NM_013435
0.48	RAXLX	NM_001008494
0.82	RBBP8	NM_203292
0.65	RBL2	BX537767
1.14	RBM19	NM_016196
0.95	RBM21	NM_022830
0.82	RBM3	NM_006743
0.88	RBM7	NM_016090
0.5	RBMX2	NM_016024
0.47	RBMY1A1	NM_005058
0.53	RBMY1B	NM_001006121
0.64	RBMY1D	NM_001006120
0.53	RBMY1E	NM_001006118
0.64	RBMY1F	X76060
0.44	RBMY1J	NM_001006117
0.69	RBP5	NM_031491
0.73	RBP7	NM_052960
0.63	RCBTB2	NM_001268
0.57	RCN1	NM_002901
0.69	RCN2	NM_002902
1.38	RDH12	NM_152443
1.07	RDH13	AK075392
0.74	RDH8	NM_015725
0.74	RDM1	NM_145654
0.66	RDX	NM_002906
0.67	REEP2	NM_016606
0.57	REEP5	NM_005669
0.73	REG1B	NM_006507
0.66	REG3G	NM_198448
0.54	REPS2	NM_004726
0.88	RERG	NM_032918
0.8	REXO1	NM_020695
0.72	RFC3	NM_181558
0.62	RFPL2	BC051910
0.53	RFWD3	BC002574
0.6	RFX2	NM_134433
0.62	RFX3	BC067778
0.62	RFX4	NM_032491
0.75	RFXDC2	NM_022841
0.75	Rg9mtd1	XM_067605
0.81	RGMB	NM_173670
0.67	RGNEF	BC012946
0.6	RGS10	NM_001005339
0.83	RGS13	NM_144766
0.62	RGS20	NM_170587
0.68	RGS9	NM_003835
0.87	RHOJ	NM_020663
0.77	RHOT1	NM_018307
0.5	RHOT2	NM_138769

0.5	RIC3	NM_024557
0.49	RIC8B	AK128102
0.59	RIMBP2	BC007632
0.62	RIMS2	NM_014677
0.74	RIN3	NM_024832
1.11	RIT2	U78166
0.71	RNASE10	NM_001012975
0.9	RNASE11	NM_145250
0.9	RNASE12	NM_001024822
0.87	RNASE13	NM_001012264
0.93	RNASE4	NM_194431
0.87	RNASE7	NM_032572
0.54	RNASEH2A	NM_006397
0.56	RNF111	NM_017610
0.63	RNF113B	NM_178861
0.89	RNF167	NM_015528
0.71	RNF190	NM_152598
0.63	RNF2	NM_007212
0.57	RNF26	NM_032015
2.32	RNF31	NM_017999
0.85	RNF5	NM_006913
0.75	RNMT	AB022605
0.52	RNPC2	NM_184244
0.65	RNU3IP2	NM_004704
1.32	ROBO3	BC008623
1.04	RP11-114H20.1	NM_001013687
0.6	RP11-119E20.1	NM_020717
0.86	RP11-11C5.2	XM_933946
0.89	RP11-125A7.3	NM_015058
0.53	RP11-139H14.4	NM_001024609
0.54	RP11-217H1.1	NM_032121
0.66	RP11-298P3.3	U50529
0.76	RP11-308B5.5	AK125991
0.63	RP11-308D16.4	NM_001004355
0.5	RP11-38O23.2	NM_205856
0.66	RP11-393H10.2	NM_152780
0.6	RP11-479E16.1	NM_174912
0.79	RP11-493K23.2	NM_001012977
0.7	RP1-14N1.3	NM_001014342
0.63	RP11-54H7.1	NM_015011
0.89	RP11-5G9.1	NM_144974
0.98	RP1-172B20.3	NM_152512
0.8	RP11-82K18.3	BC000819
0.47	RP13-11B7.1	BC101700
0.57	RP1-32F7.2	NM_173698
0.51	RP3-477H23.1	BC016465
0.74	RP4-694E4.2	NM_173566
0.68	RP5-1153D9.3	BC105792
0.83	RP5-860F19.3	BC054347
1.33	RPA1	NM_002945
0.65	RPA3	NM_002947
0.52	RPESP	NM_153225
0.62	RPH3AL	BC093776
0.48	RPL13A	NM_012423
0.53	RPL21	NM_000982
0.51	RPL3	NM_001033853
0.71	RPL35A	NM_000996
0.63	RPL36A	NM_021029
0.52	RPL36AL	NM_001001
0.52	RPL4	NM_000968
0.49	RPL41	NM_021104
0.76	RPS27A	NM_002954
0.52	RPS27L	NM_015920
0.56	RPS4Y2	NM_001039567
0.85	RPS6KA6	NM_014496
0.68	RPS6KL1	NM_031464
0.92	RPSAP15	AF284768
0.69	RPUSD4	NM_032795
0.66	RRM2	NM_001034
0.8	RSC1A1	NM_006511
0.81	RSHL2	NM_031924
0.66	RTCD1	NM_003729
0.75	RTTN	NM_173630
0.74	RUFY3	AF112221
0.47	RUNDC2A	NM_032167
0.62	RUNX1	NM_001754
0.75	RWDD1	NM_016104
1.36	RWDD3	NM_015485
0.57	RYR1	NM_000540
0.8	S100A4	NM_019554
0.8	S100A5	NM_002962
0.73	SAA3P	AY209188
1.13	SAFB	NM_002967
1.13	SAFB2	NM_014649
1	SAMD4A	BC057838
0.85	SAMD8	NM_144660
0.62	SAMHD1	NM_015474
0.98	SAMM50	NM_015380
0.52	SAP30BP	NM_013260
0.67	SAP30L	NM_024632
0.59	SART3	NM_014706
0.95	SATB1	NM_002971
1.16	SAV1	NM_021818
0.72	SBNO1	NM_018183
0.54	SBP1	NM_178121
1.04	SC4MOL	NM_006745
0.69	SC5DL	NM_006918
0.59	SC65	NM_006455
0.68	SCAP1	NM_003726
0.65	SCARB1	NM_005505
0.87	SCC-112	CR749425
0.82	SCG3	NM_013243
0.52	SCG5	NM_003020
0.57	SCGB1D2	NM_006551
1.04	SCGB1D4	NM_206998
0.79	SCML1	NM_006746
0.71	SCN5A	AB158470
0.44	SCNN1G	NM_001039
1.04	SCOC	NM_032547
0.84	SCRG1	NM_007281
0.51	SCUBE1	NM_173050
0.73	SCYL1BP1	NM_152281
0.89	SCYL3	NM_181093
0.77	SDAD1	CR749574
0.61	SDCCAG1	BX640804
0.89	SDCCAG8	NM_006642
0.68	SDF2	NM_006923
0.76	SDHD	NM_003002
0.7	SDPR	NM_004657
0.65	SDR-O	NM_148897
0.55	SDSL	NM_138432
0.88	SEC11L3	NM_033280
0.53	SEC14L2	NM_012429
0.53	SEC14L3	NM_174975
0.78	SEC61A1	NM_013336
0.75	SEC61G	NM_014302
1.55	SEL1L	NM_005065
1.27	SEMA3C	NM_006379
0.52	SEMA6D	NM_153619

0.62	SEMA7A	NM_003612
0.86	SEMG1	NM_198139
0.56	SENP3	NM_015670
0.75	SENP7	NM_020654
0.75	SERAC1	NM_032861
0.69	SERF2	NM_001018108
0.83	SERHL	NM_170694
0.48	SERPINA13	NM_207378
0.78	SERPINB1	NM_030666
0.62	SERPINB11	NM_080475
0.56	SERPINB3	NM_006919
1.05	SERPINB7	NM_003784
0.68	SERTAD1	NM_013376
0.78	SESN1	NM_014454
0.88	SETBP1	NM_015559
0.59	SETD1A	NM_014712
0.71	SETD3	NM_199123
0.59	SETD4	NM_001007260
0.51	SEZ6L	NM_021115
1.29	SF3B1	NM_012433
0.91	SF3B2	NM_006842
0.76	SFRS7	NM_001031684
0.75	SFTPG	NM_205854
0.53	SFXN2	NM_178858
1.01	SGTA	NM_003021
0.95	SH2B	NM_015503
1.17	SH2D1A	NM_002351
0.73	SH2D1B	NM_053282
0.65	SH2D3A	NM_005490
0.6	SH2D4B	AK091518
0.46	SH3BGR	NM_007341
0.6	SH3KBP1	NM_001024666
1.18	SH3PX3	NM_153271
0.53	SH3PXD2A	AL833070
0.59	SHF	NM_138356
0.85	SIAE	NM_170601
0.59	SIAH1	NM_001006610
0.48	SIGLEC10	NM_033130
0.57	SIGLEC12	NM_053003
0.52	SILV	U01874
0.75	SIM1	NM_005068
0.68	SIN3B	NM_015260
0.97	SIPA1L1	AK122930
0.89	SIPA1L3	NM_015073
0.55	SIRPB2	XM_931413
0.86	SIRT2	NM_030593
0.66	SIRT3	NM_012239
0.51	SIX5	NM_175875
0.77	SKIP	NM_130766
0.5	SKIV2L2	NM_015360
0.55	SLC10A1	NM_003049
0.66	SLC10A3	NM_019848
0.59	SLC12A6	NM_005135
0.77	SLC13A3	NM_001011554
1.09	SLC15A1	AB001328
0.89	SLC16A4	NM_004696
0.5	SLC22A12	NM_153378
0.54	SLC22A18	NM_183233
0.82	SLC22A6	NM_153279
0.92	SLC23A2	NM_005116
0.89	SLC25A11	NM_003562
0.85	SLC25A28	NM_031212
0.73	SLC25A5	NM_001152
0.69	SLC26A3	NM_000111
0.69	SLC26A5	BC100835
0.48	SLC27A1	NM_198580
0.74	SLC2A11	NM_030807
0.75	SLC2A2	NM_000340
0.75	SLC2A3	NM_006931
0.66	SLC35A3	NM_012243
0.66	SLC36A4	NM_152313
0.6	SLC38A5	NM_033518
0.8	SLC39A2	NM_014579
0.85	SLC3A2	NM_002394
0.66	SLC40A1	NM_014585
0.62	SLC44A1	NM_022109
0.69	SLC4A2	NM_003040
0.64	SLC4A4	NM_003759
0.68	SLC4A7	CR627428
0.55	SLC4A8	AK128321
0.51	SLC5A1	NM_000343
0.53	SLC5A11	AJ305237
0.63	SLC5A12	NM_178498
0.59	SLC5A8	NM_145913
0.73	SLC5A9	NM_001011547
0.45	SLC6A16	NM_014037
0.63	SLC6A17	NM_001010898
1.14	SLC6A4	BC069484
0.5	SLC7A3	NM_032803
0.97	SLC7A8	NM_012244
0.58	SLC8A3	NM_182936
0.71	SLC9A10	NM_183061
0.77	SLC9A8	NM_015266
0.74	SLIT3	NM_003062
0.49	SLITRK1	NM_052910
0.85	SLITRK4	NM_173078
0.75	SLMAP	NM_007159
0.67	SMAD1	NM_001003688
0.52	SMAD4	U44378
0.55	SMAF1	NM_001018082
0.6	SMARCA1	NM_139035
0.82	SMARCC2	NM_139067
0.52	SMARCD2	NM_003077
0.52	SMCHD1	CR627458
0.46	SMCR7	NM_148886
0.69	SMCX	NM_004187
0.56	SMG1	NM_015092
0.68	SMOX	NM_019025
0.66	SMPX	NM_014332
0.67	SMR3A	NM_012390
0.95	SMS	NM_004595
0.62	SMUG1	NM_014311
0.46	SMURF2	NM_022739
1.33	SMYD4	NM_052928
0.98	SNAP23	NM_130798
0.83	SNAP29	NM_004782
0.52	SNAPC1	NM_003082
0.64	SNCA	NM_007308
1.02	SNF1LK	NM_173354
0.52	SNPH	NM_014723
0.77	SNRPB2	NM_198220
0.57	SNRPD2	BC001930
0.86	SNRPG	NM_003096
0.82	SNRPN	NM_022805
0.88	SNX1	NM_152826
0.77	SNX11	NM_152244
0.92	SNX12	NM_013346
1.37	SNX16	NM_152837
0.84	SNX24	NM_014035

0.99	SNX5	NM_152227
0.72	SOAT2	NM_003578
0.66	SOCS5	NM_144949
0.52	SON	NM_138927
0.6	SORBS1	NM_006434
0.53	SORT1	NM_002959
0.87	SOS2	NM_006939
1.11	SOX5	NM_006940
0.69	SP4	NM_003112
0.59	SP6	NM_199262
0.95	SP8	NM_198956
0.85	SPA17	NM_017425
1.05	SPAG4	NM_003116
0.8	SPAG4L	NM_080675
0.89	SPAG6	NM_172242
0.49	SPATA20	NM_022827
0.57	SPATA5	NM_145207
0.54	SPCS3	NM_021928
0.5	SPFH1	NM_006459
0.65	SPG21	NM_016630
1.2	SPI1	NM_003120
0.49	SPIC	NM_152323
0.59	SPIN1	NM_032038
0.71	SPINK1	NM_003122
1.08	SPINLW1	NM_181502
0.46	SPINT4	XM_372869
1.01	SPIRE1	NM_020148
0.54	SPPL2B	NM_152988
0.69	SPRN	NM_001012508
1.08	SPRY3	NM_005840
0.55	SPRYD4	NM_207344
0.57	SPRYD5	NM_032681
0.76	SPTBN2	NM_006946
0.74	SPTLC2L	AK075271
0.63	SPTY2D1	NM_194285
0.65	SQRDL	NM_021199
0.71	SR140	XM_031553
0.58	SRP54	NM_003136
0.52	SRP68	NM_014230
0.75	SRPK1	NM_003137
0.65	SRPK2	NM_182692
0.6	SRPR	NM_003139
0.5	SRRM2	NM_016333
0.78	SRY	NM_003140
0.57	SSR4	NM_006280
1.42	SSRP1	NM_003146
0.55	SSTR1	NM_001049
0.49	SSTR2	NM_001050
0.51	SSTR3	BC096829
0.5	SSX2	NM_175698
0.8	ST3GAL2	NM_006927
0.57	ST3GAL4	CR604023
1.02	ST6GALNAC1	NM_018414
0.82	ST7	AY009153
1.04	ST8SIA1	NM_003034
0.47	STAMBPL1	NM_020799
0.66	STARD13	NM_178008
0.62	STARD3NL	BC003074
0.79	STARD6	NM_139171
0.52	STARD9	CR627426
1.08	STAT3	NM_213662
0.62	STAU2	BC110447
0.67	STC2	NM_003714
0.86	STCH	NM_006948
0.65	STEAP1	NM_012449
0.59	STH	NM_001007532
0.64	STK10	BC014413
0.65	STK19	X77386
0.54	STK33	NM_030906
0.59	STK35	NM_080836
0.63	STS-1	NM_032873
0.52	STX16	AF305817
0.6	STX3A	NM_004177
1.08	STXBP4	NM_178509
0.59	STYK1	NM_018423
0.78	SUDS3	BC093990
0.62	SUHW4	AK000093
0.85	SULF1	AK074873
0.86	SULF2	BC020962
0.44	SULT1A3	NM_177552
0.57	SULT2A1	NM_003167
0.6	SULT2B1	NM_177973
0.65	SULT4A1	NM_176874
0.68	SUMO2	NM_006937
0.49	SUPT6H	NM_003170
1.21	SURB7	NM_004264
0.78	SVOP	NM_018711
1.36	SYAP1	NM_032796
0.55	SYCP2	Y08982
0.56	SYN3	NM_133633
0.51	SYNCRIP	NM_006372
1.06	SYNE2	NM_182913
0.69	SYT10	NM_198992
0.6	SYT12	NM_177963
0.66	SYT2	NM_177402
0.56	SYT4	NM_020783
0.85	SYT7	NM_004200
1.32	SYT9	BC029605
0.6	SYTL4	NM_080737
0.85	T1560	NM_199048
0.92	TAB3	NM_198312
0.46	TADA2L	BC011753
0.78	TAF1L	NM_153809
0.71	TAF4	NM_003185
0.66	TAF7L	NM_024885
0.65	TAGAP	NM_152133
0.9	TANK	NM_133484
0.84	TARS	NM_152295
0.55	TAS2R10	NM_023921
0.98	TAS2R14	NM_023922
0.59	TAS2R43	NM_176884
0.55	TAS2R44	NM_176885
0.62	TAS2R46	NM_176887
0.69	TAS2R8	NM_023918
0.69	TAS2R9	NM_023917
0.63	TATDN3	XM_931857
0.62	TBC1D15	NM_022771
0.55	TBC1D20	NM_144628
0.45	TBC1D22A	NM_014346
0.56	TBC1D2B	NM_015079
0.56	TBC1D3	BC078140
0.56	TBC1D3B	BC075809
0.56	TBC1D3C	BC071680
0.75	TBC1D5	NM_014744
0.57	TBL1X	NM_005647
0.5	TCEAL5	NM_001012979
0.56	TCEB3B	NM_016427
0.59	TCEB3C	NM_145653
0.52	TCF12	NM_207040

0.65	TCF2	NM_006481
0.81	TCP1	NM_030752
1.05	TCP10L	NM_144659
0.82	TCP11L1	NM_018393
0.85	TCP11L2	NM_152772
1.14	TDG	NM_003211
0.52	TDP1	NM_018319
0.5	TDRD1	NM_198795
1.22	TDRD9	NM_153046
1.01	TEAD1	BC026959
0.57	TEAD2	BC018803
1.04	TECTA	NM_005422
0.74	TEF	NM_003216
0.68	TEPP	NM_199456
0.57	TEX13A	NM_031274
1.14	TEX13B	NM_031273
1.17	TEX14	NM_198393
0.68	TEX15	NM_031271
1.05	TEX9	NM_198524
0.76	TFAM	NM_003201
0.55	TFAP2C	NM_003222
0.74	TFF2	NM_005423
0.74	TGFB1I1	NM_015927
0.63	TGFB2	NM_003238
0.68	TGFBR1	NM_004612
0.46	TGM2	NM_198951
0.68	TGM6	NM_198994
0.8	THAP11	NM_020457
0.75	THAP5	NM_182529
0.74	THAP9	NM_024672
0.49	THBD	NM_000361
0.62	THOC1	NM_005131
1.01	THOP1	NM_003249
0.56	THRA	NM_199334
0.89	THRAP1	NM_005121
0.68	THRAP4	NM_014815
0.72	THSD4	NM_024817
0.83	THUMPD2	AF380578
0.79	THY1	NM_006288
0.63	TIAL1	NM_003252
1.19	TIMM13	NM_012458
0.6	TIMM17B	NM_005834
0.54	TIMM8A	NM_004085
0.76	TIMM8B	NM_012459
1.06	TIMM9	NM_012460
0.76	TIMP1	NM_003254
0.68	TIMP2	AK057217
0.74	TIMP3	NM_000362
0.47	TIRAP	NM_148910
0.61	TITF1	NM_003317
0.59	TK2	Y10498
0.51	TLE6	NM_024760
0.5	TLL2	NM_012465
0.8	TM4SF20	NM_024795
0.93	TM9SF1	NM_006405
0.86	TM9SF2	NM_004800
0.77	TM9SF4	BC021107
0.7	TMBIM1	NM_022152
0.88	TMBIM4	NM_016056
0.68	TMC1	NM_138691
0.77	TMC5	AY358155
0.68	TMCC1	NM_001017395
0.62	TMCO5	NM_152453
0.86	TMED1	NM_006858
0.68	TMEFF1	AL831919
0.62	TMEM100	NM_018286
0.99	TMEM104	NM_017728
0.95	TMEM109	NM_024092
1.05	TMEM11	NM_003876
0.62	TMEM116	NM_138341
0.59	TMEM118	NM_032814
1.1	TMEM122	NM_173801
0.79	TMEM126A	NM_032273
0.5	TMEM126B	NM_018480
0.55	TMEM132B	NM_052907
1.07	TMEM136	NM_174926
0.91	TMEM16B	AK024010
0.54	TMEM16C	NM_031418
0.85	TMEM16D	NM_178826
0.53	TMEM23	NM_147156
0.57	TMEM25	NM_032780
0.5	TMEM29	BC067819
1.45	TMEM30B	NM_001017970
0.6	TMEM31	NM_182541
0.98	TMEM35	NM_021637
0.85	TMEM4	NM_014255
1.39	TMEM49	NM_030938
0.76	TMEM77	NM_178454
0.53	TMEM81	NM_203376
0.82	TMEM84	NM_173610
1.04	TMEM91	BC063705
1.01	TMPO	NM_003276
0.6	TMPRSS11D	NM_004262
0.6	TMPRSS11E	NM_014058
0.47	TMSB4Y	NM_004202
0.63	TMSL8	NM_021992
1.11	TMTC2	NM_152588
0.72	TMTC3	NM_181783
1.05	TMTC4	NM_032813
0.56	TNFAIP1	NM_021137
0.88	TNFAIP3	BC114480
0.7	TNFAIP6	NM_007115
0.62	TNFAIP8L3	NM_207381
0.68	TNFRSF11B	NM_002546
0.77	TNFRSF13B	NM_012452
0.59	TNFRSF13C	NM_052945
1.02	TNFRSF19	NM_148957
0.73	TNFRSF4	NM_003327
0.68	TNFSF15	AY434464
0.68	TNK2	NM_001010938
0.76	TNMD	NM_022144
0.62	TNNC2	NM_003279
0.66	TNNI3K	BC032865
0.64	TNPO1	NM_002270
0.83	TNPO2	NM_013433
0.53	TNRC6B	NM_001024843
0.74	TNRC6C	NM_018996
0.71	TOB2	NM_016272
1.2	TOM1L1	NM_005486
0.74	TOM1L2	NM_144678
0.68	TOMM22	NM_020243
0.65	TOMM70A	NM_014820
0.86	TOR1AIP2	BC112225
0.65	TP53BP1	NM_005657
0.73	TP53BP2	NM_005426
0.53	TP53TG3	NM_016212
0.68	TP73L	NM_003722
1.09	TPD52L1	AF004427
1.04	TPH1	NM_004179

0.69	TPH2	NM_173353
0.98	TPM1	NM_001018020
0.54	TPP1	NM_000391
0.86	TPP2	NM_003291
0.7	TPRKB	NM_016058
0.56	TPT1	NM_003295
0.84	TRA@	BC110354
0.65	TRA16	NM_176880
0.5	TRAF6	NM_145803
0.62	TRAFD1	BC003553
0.64	TRAM1L1	NM_152402
0.8	TRAPPC1	NM_021210
0.47	TRAPPC2	NM_014563
0.78	TRAT1	NM_016388
0.64	TRDV2	BC106077
0.85	TREML3	XM_929970
0.85	TREML4	NM_198153
0.78	TRIB1	NM_025195
0.49	TRIB3	NM_021158
0.63	TRIM29	NM_058193
1.42	TRIM3	AF220021
0.73	TRIM34	NM_001003827
0.57	TRIM44	NM_017583
0.6	TRIM49	BC075020
0.5	TRIM68	NM_018073
0.71	TRIM72	NM_001008274
0.65	TRIP4	NM_016213
0.59	TRPC4	NM_016179
0.65	TRPM3	NM_001007471
0.52	TRPV1	NM_080704
0.55	TRPV4	DQ059646
0.63	TRUB1	NM_139169
0.78	TRY1	NM_001001317
0.47	TSC22D3	NM_198057
0.71	TSKS	NM_021733
0.48	TSPAN16	NM_012466
0.68	TSPYL1	NM_003309
0.6	TSPYL2	NM_022117
0.68	TSPYL4	NM_021648
0.98	TSSK2	NM_053006
0.9	TSSK4	NM_174944
0.56	TTBK2	NM_173500
0.73	TTC13	NM_024525
1.36	TTC17	NM_018259
0.8	TTC19	NM_017775
0.7	TTC22	NM_017904
0.49	TTC3	D84296
1.29	TTC6	NM_001007795
0.6	TTC9C	NM_173810
0.64	TTLL5	NM_015072
1.23	TTLL7	AK026686
0.64	TTTY10	AF332239
0.58	TTTY11	AF332240
1.14	TTTY6	AF332237
0.53	TTTY8	BC026100
0.56	TTTY9A	AF332238
0.6	TUB	NM_177972
0.76	TUBA1	NM_006000
0.45	TUBA8	NM_018943
0.53	TUBAL3	NM_024803
0.71	TUBB1	NM_030773
0.65	TUBE1	NM_016262
0.47	TUFM	S75463
0.68	TUSC2	NM_007275
0.78	TXNDC6	NM_178130
0.7	TXNDC9	NM_005783
0.55	TXNRD1	NM_182743
1.23	U2AF1L2	NM_005089
0.56	UACA	NM_018003
0.87	UBAP2	AK026088
0.55	UBE2C	NM_181803
0.94	UBE2D1	NM_003338
0.91	UBE2N	NM_003348
0.78	UBE2W	NM_018299
1.08	UBE2Z	NM_023079
0.62	UBE3A	BC009271
0.52	UBE3B	NM_183415
0.8	UBE4B	NM_006048
1.88	UBL3	NM_007106
0.66	UBL4A	NM_014235
0.62	UBL7	NM_201265
0.5	UBQLN2	NM_013444
0.71	UBXD7	XM_931530
0.53	UFM1	NM_016617
0.7	UGT1A4	NM_007120
0.76	UGT1A6	NM_205862
0.71	UHRF2	NM_152896
0.59	UMOD	NM_003361
0.71	UNC93A	NM_018974
0.63	UNC93B1	NM_030930
0.5	UNKL	XM_930586
1.14	UNQ3072	NM_001013442
0.57	UNQ501	NM_198536
0.65	UNQ5783	NM_207103
0.62	UNQ739	NM_198570
0.5	UPK2	NM_006760
0.5	UQCRC2	NM_003366
0.63	UROD	NM_000374
0.76	USH2A	NM_206933
0.92	USH3A	NM_174878
0.57	USHBP1	NM_031941
1.14	USP11	U44839
0.76	USP12	NM_182488
1.37	USP15	NM_006313
0.76	USP26	NM_031907
0.54	USP28	NM_020886
0.85	USP3	NM_006537
1.11	USP32	NM_032582
0.99	USP33	NM_201626
0.71	USP36	BC071582
0.65	USP42	XM_927597
0.59	USP44	NM_032147
0.54	USP51	NM_201286
0.64	USP53	NM_019050
0.98	USP54	BC110845
0.6	UTP14A	NM_006649
0.64	UTP15	NM_032175
0.5	UTY	NM_182660
1.17	VAMP2	NM_014232
0.65	VAPB	AK128422
0.65	VAV1	NM_005428
0.92	VBP1	NM_003372
0.63	VCAM1	NM_080682
0.62	VCP	NM_007126
0.54	VCX2	NM_016378
1.55	VCX3A	NM_016379
0.44	VCY	NM_004679
0.56	VCY1B	NM_181880
0.79	VGCNL1	BC064343

0.79	VGLL1	NM_016267
0.89	VKORC1	NM_206824
0.74	VN1R4	NM_173857
0.5	VPS11	NM_021729
1.5	VPS13C	NM_020821
0.65	VPS29	NM_057180
0.49	VPS33A	NM_022916
0.82	VPS39	NM_015289
0.73	VPS45A	NM_007259
0.82	VSIG1	NM_182607
0.92	VSIG4	NM_007268
0.6	VTI1A	NM_145206
0.64	WARS	NM_213646
0.83	WASF2	NM_006990
0.73	WASPIP	NM_003387
0.49	WBP2	NM_012478
1.25	WBP4	NM_007187
0.62	WBSCR17	NM_022479
1	WDR1	NM_017491
1.61	WDR13	NM_017883
0.64	WDR25	NM_024515
0.83	WDR3	NM_006784
0.55	WDR31	NM_145241
0.76	WDR39	NM_004804
0.73	WDR40B	NM_178470
0.76	WDR42B	NM_001017930
0.56	WDR45L	NM_019613
0.82	WDR61	NM_025234
0.57	WDR62	BC017261
0.96	WDR63	NM_145172
1	WDR75	NM_032168
0.44	WFDC1	NM_021197
0.52	WFDC2	NM_080736
0.8	WFDC6	NM_080827
0.55	WFDC8	NM_181510
0.99	WFDC9	NM_147198
0.52	WHDC1L1	XM_931261
0.78	WIG1	NM_152240
0.71	WIPI1	NM_017983
0.65	WISP2	AK129660
1.04	WIZ	XM_930438
0.92	WNK3	NM_020922
0.98	WNT2	NM_003391
0.62	WNT3	NM_030753
0.55	WNT5B	NM_032642
0.93	WRB	NM_004627
0.65	WRN	NM_000553
0.68	WSB1	NM_134265
0.77	WWC2	AK126057
0.63	WWC3	BC035601
0.68	WWOX	NM_130844
0.5	WWP2	NM_199424
0.53	XBP1	NM_005080
0.89	XKR7	NM_001011718
0.59	XPO6	NM_015171
0.6	XRCC1	NM_006297
1.13	XRCC3	NM_005432
0.82	XRCC6BP1	NM_033276
0.52	XRN2	NM_012255
0.54	XRRA1	XM_934948
0.5	YAP1	NM_006106
0.79	YIPF6	NM_173834
0.9	YSK4	BC034417
0.62	YTHDF1	NM_017798
0.62	YTHDF3	NM_152758
0.49	YWHAB	BC010352
0.74	YWHAH	NM_003405
0.49	ZADH2	NM_175907
0.78	ZBTB10	BC050061
0.73	ZBTB3	NM_024784
0.77	ZBTB32	NM_014383
0.57	ZBTB33	NM_006777
0.49	ZBTB39	NM_014830
0.49	ZBTB4	NM_020899
0.48	ZBTB7A	NM_015898
0.49	ZC3H10	NM_032786
1.21	ZCCHC14	BC028304
0.85	ZCCHC2	NM_017742
0.49	ZCCHC3	NM_033089
0.62	ZCCHC6	NM_024617
0.72	ZCCHC8	NM_017612
0.71	ZCCHC9	NM_032280
0.66	ZCSL3	NM_181706
1.21	ZDHHC1	NM_013304
0.91	ZDHHC13	NM_019028
1.01	ZDHHC16	CR620516
0.66	ZDHHC20	NM_153251
0.9	ZDHHC22	NM_174976
0.85	ZDHHC24	NM_207340
0.5	ZDHHC6	NM_022494
0.74	ZDHHC7	BC017702
1.11	ZFP106	NM_022473
0.51	ZFP28	NM_020828
0.47	ZFP90	NM_133458
0.71	ZFYVE20	BC106940
0.55	ZFYVE26	NM_015346
0.88	ZIC3	NM_003413
0.57	ZIM2	NM_015363
1.42	ZMAT1	NM_032441
0.92	ZMAT5	NM_019103
0.7	ZMPSTE24	NM_005857
0.69	ZMYM3	X95808
0.73	ZMYM6	NM_007167
0.52	ZNF10	NM_015394
0.78	ZNF12	BX640749
0.54	ZNF132	NM_003433
1.02	ZNF133	NM_003434
0.65	ZNF134	NM_003435
0.51	ZNF136	NM_003437
0.66	ZNF143	NM_003442
0.45	ZNF154	NM_003444
0.65	ZNF155	NM_198089
0.68	ZNF17	NM_006959
0.54	ZNF175	NM_007147
0.8	ZNF177	NM_003451
0.52	ZNF18	BC036096
0.79	ZNF185	BX537525
0.98	ZNF20	NM_021143
0.6	ZNF202	NM_003455
0.81	ZNF204	AL833520
0.5	ZNF21	BC106875
0.98	ZNF214	NM_013249
0.98	ZNF215	BC014538
0.52	ZNF217	NM_006526
0.68	ZNF219	BC000694
0.57	ZNF221	NM_013359
0.68	ZNF222	NM_013360
0.95	ZNF223	NM_013361
0.71	ZNF226	NM_015919

0.98	ZNF227	NM_182490
0.68	ZNF229	NM_014518
0.72	ZNF24	NM_006965
1.55	ZNF256	NM_005773
0.56	ZNF267	NM_003414
1.79	ZNF268	NM_152943
0.56	ZNF271	BC017710
0.6	ZNF272	AK090816
0.56	ZNF276	BC110348
0.75	ZNF277	NM_021994
1.16	ZNF285	NM_152354
0.82	ZNF291	BC015212
0.65	ZNF292	XM_932361
0.59	ZNF294	NM_015565
0.62	ZNF295	NM_020727
0.98	ZNF30	NM_194325
0.86	ZNF302	NM_018443
0.45	ZNF320	NM_207333
0.51	ZNF324	AK023989
0.6	ZNF331	NM_018555
0.68	ZNF342	NM_145288
0.65	ZNF35	NM_003420
0.83	ZNF350	NM_021632
0.71	ZNF358	BC014002
0.87	ZNF366	NM_152625
1.01	ZNF382	NM_032825
0.55	ZNF384	NM_001039916
0.54	ZNF404	BC101333
0.78	ZNF406	NM_020863
0.56	ZNF407	NM_017757
0.54	ZNF416	NM_017879
0.65	ZNF419	NM_024691
0.65	ZNF42	NM_198055
0.5	ZNF423	NM_015069
0.54	ZNF430	NM_025189
0.86	ZNF432	NM_014650
0.54	ZNF433	NM_152602
0.56	ZNF434	AK000424
0.63	ZNF438	NM_182755
0.54	ZNF440	NM_152357
0.71	ZNF442	NM_030824
0.63	ZNF449	NM_152695
0.68	ZNF452	NM_052923
0.71	ZNF454	NM_182594
0.74	ZNF483	NM_133464
0.54	ZNF490	NM_020714
0.68	ZNF502	NM_033210
0.8	ZNF524	NM_153219
1.1	ZNF527	XM_933045
0.63	ZNF533	AK096822
0.62	ZNF536	NM_014717
0.62	ZNF540	AK131388
0.68	ZNF542	NM_194319
0.57	ZNF546	NM_178544
0.57	ZNF550	NM_153231
0.62	ZNF560	NM_152476
0.54	ZNF563	NM_145276
0.45	ZNF564	NM_144976
0.65	ZNF567	NM_152603
1.04	ZNF568	NM_198539
0.48	ZNF582	NM_144690
0.68	ZNF583	NM_152478
0.51	ZNF585A	NM_199126
1.04	ZNF585B	NM_152279
0.6	ZNF6	BC067294
0.74	ZNF611	NM_030972
0.51	ZNF614	AK025594
0.65	ZNF624	NM_020787
0.62	ZNF653	NM_138783
1.04	ZNF658	AL110217
1.02	ZNF659	NM_024697
1.04	ZNF667	NM_022103
0.82	ZNF673	NM_017776
0.59	ZNF690	NM_152455
0.57	ZNF699	XM_371132
0.57	ZNF708	NM_021269
0.45	ZNF709	AK095600
0.52	ZNF710	NM_198526
0.74	ZNF74	NM_003426
0.49	ZNF740	NM_001004304
0.63	ZNF75	NM_007131
0.62	ZNFN1A1	NM_006060
0.59	ZNFX1	NM_021035
0.73	ZNHIT2	NM_014205
0.71	ZNHIT3	NM_004773
0.59	ZSCAN2	NM_181877
0.48	ZSCAN5	BC043232
0.96	ZSWIM2	NM_182521
0.52	ZWILCH	NM_017975
0.5	ZWINT	NM_032997
0.62	ZZEF1	NM_015113

Supporting Information Table S2. Gene ontology from ChIP on chip data of EZH2 target genes in undifferentiated hMSCs.

(Peak Score > 0.2 and FDR < 0.05)

GO Terms (78)	number (3110 genes)
Amino Acid Metabolism	1
Auditory and Vestibular System Development and Functi	7
Auditory Disease	1
Behavior	1
Cancer	317
Carbohydrate Metabolism	7
Cardiovascular Disease	88
Cardiovascular System Development and Function	3
Cell Cycle	103
Cell Death	37
Cell Morphology	17
Cell Signaling	109
Cell-To-Cell Signaling and Interaction	35
Cellular Assembly and Organization	91
Cellular Compromise	8
Cellular Development	83
Cellular Function and Maintenance	33
Cellular Growth and Proliferation	218
Cellular Movement	135
Connective Tissue Development and Function	37
Connective Tissue Disorders	3
Dermatological Diseases and Conditions	53
Developmental Disorder	25
Digestive System Development and Function	1
DNA Replication, Recombination, and Repair	85
Drug Metabolism	4
Embryonic Development	24
Endocrine System Development and Function	4
Endocrine System Disorders	20
Energy Production	12
Gastrointestinal Disease	12
Gene Expression	297
Genetic Disorder	30
Hair and Skin Development and Function	10
Hematological Disease	27
Hematological System Development and Function	26
Hepatic System Disease	7
Immune and Lymphatic System Development and Functi	21
Immune Response	6
Immunological Disease	46
Infectious Disease	4
Inflammatory Disease	24
Lipid Metabolism	74
Metabolic Disease	6
Molecular Transport	10
Nervous System Development and Function	48
Neurological Disease	147
Nucleic Acid Metabolism	18
Ophthalmic Disease	36
Organ Development	10
Organ Morphology	23
Organismal Development	27
Organismal Functions	4
Organismal Injury and Abnormalities	66
Organismal Survival	21
Post-Translational Modification	66
Protein Degradation	48
Protein Synthesis	43
Protein Trafficking	4
Psychological Disorders	40
Renal and Urological Disease	7
Renal and Urological System Development and Function	2
Reproductive System Development and Function	23
Reproductive System Disease	20
Respiratory Disease	24
Respiratory System Development and Function	3
RNA Damage and Repair	3
RNA Post-Transcriptional Modification	17
Skeletal and Muscular Disorders	9
Skeletal and Muscular System Development and Functio	27
Small Molecule Biochemistry	106
Tissue Development	43
Tissue Morphology	45
Tumor Morphology	11
Viral Function	1
Viral Infection	1
Visual System Development and Function	4
Vitamin and Mineral Metabolism	1

	Groups	GO Terms	number	Total 3110 genes	%	include 388 genes
1	Metabolism	Amino Acid Metabolism	1	105	3.4	
		Carbohydrate Metabolism	7			
		Drug Metabolism	4			
		Vitamin and Mineral Metabolism	1			
		Nucleic Acid Metabolism	18			
		Lipid Metabolism	74			
2	Disease	Auditory Disease	1	525	17	
		Cardiovascular Disease	88			
		Connective Tissue Disorders	3			
		Dermatological Diseases and Conditions	53			
		Developmental Disorder	25			
		Hematological Disease	27			
		Endocrine System Disorders	20			
		Skeletal and Muscular Disorders	9			
		Respiratory Disease	24			
		Reproductive System Disease	20			
		Psychological Disorders	40			
		Gastrointestinal Disease	12			
		Hepatic System Disease	7			
		Ophthalmic Disease	36			
		Neurological Disease	147			
		Metabolic Disease	6			
		Renal and Urological Disease	7			
3	Development and Functio	Auditory and Vestibular System Development ar	7	371	12	
		Cardiovascular System Development and Funct	3			
		Connective Tissue Development and Function	37			
		Digestive System Development and Function	1			
		Embryonic Development	24			
		Endocrine System Development and Function	4			
		Hair and Skin Development and Function	10			
		Hematological System Development and Functi	26			
		Organismal Development	27			
		Visual System Development and Function	4			
		Tissue Development	43			
		Skeletal and Muscular System Development an	27			
		Respiratory System Development and Function	3			
		Reproductive System Development and Functio	23			
		Organ Development	10			
		Renal and Urological System Development and	2			
		Cellular Development	83			
		Cellular Function and Maintenance	33			
		Organismal Functions	4			
**4**	**Nervous System**	**Nervous System Development and Function**	**48**	**48**	**1.5**	
5	Other GO terms	Behavior	1	178	5.7	
		Cellular Compromise	8			
		Energy Production	12			
		Tissue Morphology	45			
		Viral Function	1			
		Viral Infection	1			
		Organismal Injury and Abnormalities	66			
		Organismal Survival	21			
		Organ Morphology	23			
6	Cell Cycle and Morpholog	Cellular Growth and Proliferation	218	745	24	
		Cell Cycle	103			
		Cell Death	37			
		Cell Morphology	17			
		Cell Signaling	109			
		Cell-To-Cell Signaling and Interaction	35			
		Cellular Assembly and Organization	91			
		Cellular Movement	135			
7	Immune	Immune and Lymphatic System Development ar	21	101	3.2	
		Immune Response	6			
		Immunological Disease	46			
		Infectious Disease	4			
		Inflammatory Disease	24			
8	Gene Expression	Gene Expression	297	327	11	
		Genetic Disorder	30			
9	Cancer	Cancer	317	328	11	
		Tumor Morphology	11			
10	Molecule Biochemistry	Protein Degradation	48	382	12	
		Protein Synthesis	43			
		Post-Translational Modification	66			
		Protein Trafficking	4			
		RNA Damage and Repair	3			
		RNA Post-Transcriptional Modification	17			
		Small Molecule Biochemistry	106			
		DNA Replication, Recombination, and Repair	85			
		Molecular Transport	10			

Supporting InformationTable S3. Genes list from gene ontology data of EZH2 target genes in undifferentiated hMSCs by ChIP-on-chip

(Peak Score > 0.2 and FDR < 0.05 for gene ontology analysis)

Name	accession	CHROMOSOME	PEAK_SCORE	PEAK_FDR	ncbi_gene_id	description
ABAT	BC008990	chr16	0.8	2.29E-02	18	4-aminobutyrate aminotransferase
ABCB7	NM_004299	chrX	1.07	5.68E-03	22	ATP-binding cassette, sub-family B (MDR/TAP), member 7
ACADL	NM_001608	chr2	0.96	1.17E-02	33	acyl-Coenzyme A dehydrogenase, long chain
ACIN1	NM_014977	chr14	1.03	7.66E-03	22985	apoptotic chromatin condensation inducer 1
ACSM1	NM_052956	chr16	0.77	2.69E-02	116285	acyl-CoA synthetase medium-chain family member 1
ADAM12	NM_021641	chr10	0.85	1.89E-02	8038	ADAM metallopeptidase domain 12 (meltrin alpha)
ADAMTS20	NM_175851	chr12	0.78	2.87E-02	80070	ADAM metallopeptidase with thrombospondin type 1 motif, 20
ADAMTS5	NM_007038	chr21	0.59	4.35E-02	11096	ADAM metallopeptidase with thrombospondin type 1 motif, 5 (aggrecanase-2)
ADAT1	NM_012091	chr16	0.74	3.27E-02	23536	adenosine deaminase, tRNA-specific 1
ADRA1D	NM_000678	chr20	1.02	6.89E-03	146	adrenergic, alpha-1D-, receptor
ADRB2	NM_000024	chr5	0.87	3.40E-02	154	adrenergic, beta-2-, receptor, surface
AGPAT1	NM_032741	chr6	0.85	4.51E-02	10554	1-acylglycerol-3-phosphate O-acyltransferase 1 (lysophosphatidic acid acyltransferase, alpha)
AKAP5	NM_004857	chr14	0.77	4.10E-02	9495	A kinase (PRKA) anchor protein 5
AKR1C3	NM_003739	chr10	1.03	3.95E-03	8644	aldo-keto reductase family 1, member C3 (3-alpha hydroxysteroid dehydrogenase, type II)
AKT2	NM_001626	chr19	1.22	0.00E+00	208	v-akt murine thymoma viral oncogene homolog 2
ANG	NM_001145	chr14	0.8	3.27E-02	283	angiogenin, ribonuclease, RNase A family, 5
ANKRD1	NM_014391	chr10	0.94	1.24E-02	27063	ankyrin repeat domain 1 (cardiac muscle)
ANXA4	BC063672	chr2	0.83	4.51E-02	307	annexin A4
AP3M2	NM_006803	chr8	0.62	3.01E-02	10947	adaptor-related protein complex 3, mu 2 subunit
APEX1	NM_080649	chr14	0.8	2.02E-02	328	APEX nuclease (multifunctional DNA repair enzyme) 1
APLP1	AK126907	chr19	0.83	1.91E-02	333	amyloid beta (A4) precursor-like protein 1
APOA5	NM_052968	chr11	1.04	6.89E-03	116519	apolipoprotein A-V
APRIN	NM_015928	chr13	0.86	2.69E-02	23047	androgen-induced proliferation inhibitor
AR	NM_00101164	chrX	0.82	2.69E-02	367	androgen receptor (dihydrotestosterone receptor%3B testicular feminization%3B spinal and bulbar muscular atrophy%3B Kennedy disease)
ARHGAP4	NM_001666	chrX	0.82	2.69E-02	393	Rho GTPase activating protein 4
ARHGEF6	NM_004840	chrX	0.76	4.10E-02	9459	Rac/Cdc42 guanine nucleotide exchange factor (GEF) 6
ARID4A	NM_023001	chr14	0.64	3.46E-02	5926	AT rich interactive domain 4A (RBP1-like)
ASF1A	NM_014034	chr6	0.92	2.38E-02	25842	ASF1 anti-silencing function 1 homolog A (S. cerevisiae)
ASMT	NM_004043	chrY	0.72	2.69E-02	438	acetylserotonin O-methyltransferase
ASPSCR1	NM_024083	chr17	0.74	4.10E-02	79058	alveolar soft part sarcoma chromosome region, candidate 1
ATF7IP	AK001001	chr12	0.55	3.01E-02	55729	activating transcription factor 7 interacting protein
ATM	NM_000051	chr11	0.82	2.69E-02	472	ataxia telangiectasia mutated (includes complementation groups A, C and D)
ATP6AP1	NM_001183	chrX	0.98	1.05E-02	537	ATPase, H+ transporting, lysosomal accessory protein 1
ATP8A2	NM_016529	chr13	0.89	4.48E-02	51761	ATPase, aminophospholipid transporter-like, Class I, type 8A, member 2
ATXN7	NM_000333	chr3	0.92	2.38E-02	6314	ataxin 7
AURKA	NM_198437	chr20	0.99	7.66E-03	6790	aurora kinase A
B2M	S82297	chr15	0.85	2.69E-02	567	beta-2-microglobulin
B3GNT2	BC030579	chr2	0.86	3.40E-02	10678	UDP-GlcNAc:betaGal beta-1,3-N-acetylglucosaminyltransferase 2
B4GALT5	NM_004776	chr20	0.77	3.27E-02	9334	UDP-Gal:betaGlcNAc beta 1,4- galactosyltransferase, polypeptide 5
BARHL2	NM_020063	chr1	0.83	4.51E-02	343472	BarH-like 2 (Drosophila)
BDNF	NM_170735	chr11	1.01	7.66E-03	627	brain-derived neurotrophic factor
BICD1	NM_001714	chr12	0.82	2.02E-02	636	bicaudal D homolog 1 (Drosophila)
BIRC4	NM_001167	chrX	0.79	3.27E-02	331	baculoviral IAP repeat-containing 4
BLMH	NM_000386	chr17	0.77	3.27E-02	642	bleomycin hydrolase
BNIP3	NM_004052	chr10	1.04	0.00E+00	664	BCL2/adenovirus E1B 19kDa interacting protein 3

BRD4	NM_058243	chr19	0.83	1.91E-02	23476	bromodomain containing 4
BRD7	NM_013263	chr16	0.8	2.29E-02	29117	bromodomain containing 7
C1QBP	NM_001212	chr17	0.99	7.66E-03	708	complement component 1, q subcomponent binding protein
C1QDC1	NM_032156	chr12	0.95	1.56E-02	65981	C1q domain containing 1
CA12	BC001012	chr15	1.15	4.99E-03	771	carbonic anhydrase XII
CA7	NM_005182	chr16	0.83	1.91E-02	766	carbonic anhydrase VII
CAND1	NM_018448	chr12	0.98	1.24E-02	55832	cullin-associated and neddylation-dissociated 1
CAPN1	NM_005186	chr11	0.76	4.10E-02	823	calpain 1, (mu/I) large subunit
CARHSP1	BC108283	chr16	0.56	4.35E-02	23589	calcium regulated heat stable protein 1, 24kDa
CASP5	NM_004347	chr11	0.76	4.10E-02	838	caspase 5, apoptosis-related cysteine peptidase
CBL	NM_005188	chr11	1.01	2.09E-02	867	Cas-Br-M (murine) ecotropic retroviral transforming sequence
CBX7	NM_175709	chr22	1.01	1.92E-02	23492	chromobox homolog 7
CC2D1A	CR608350	chr19	0.74	3.27E-02	54862	coiled-coil and C2 domain containing 1A
CCDC6	NM_005436	chr10	1.16	4.44E-03	8030	coiled-coil domain containing 6
CCKBR	NM_176875	chr11	1.8	0.00E+00	887	cholecystokinin B receptor
CD274	BC074984	chr9	0.84	3.40E-02	29126	CD274 molecule
CD33	NM_001772	chr19	1.16	7.52E-03	945	CD33 molecule
CDC16	NM_003903	chr13	1.12	1.92E-02	8881	CDC16 cell division cycle 16 homolog (S. cerevisiae)
CDC42SE2	NM_020240	chr5	0.84	4.51E-02	56990	CDC42 small effector 2
CDC6	NM_001254	chr17	0.83	2.29E-02	990	CDC6 cell division cycle 6 homolog (S. cerevisiae)
CDC73	NM_024529	chr1	0.86	3.40E-02	79577	cell division cycle 73, Paf1/RNA polymerase II complex component, homolog (S. cerevisiae)
CDH2	NM_001792	chr18	0.85	2.69E-02	1000	cadherin 2, type 1, N-cadherin (neuronal)
CDH4	AK091496	chr20	0.89	3.39E-02	1002	cadherin 4, type 1, R-cadherin (retinal)
CDK2AP1	NM_004642	chr12	0.98	1.24E-02	8099	CDK2-associated protein 1
CDK2AP2	NM_005851	chr11	1.23	8.35E-04	10263	CDK2-associated protein 2
CEP350	NM_014810	chr1	0.86	3.40E-02	9857	centrosomal protein 350kDa
CFDP1	NM_006324	chr16	0.71	4.10E-02	10428	craniofacial development protein 1
CFL1	NM_005507	chr11	1.04	2.07E-02	1072	cofilin 1 (non-muscle)
CHD4	NM_001273	chr12	1.21	4.44E-03	1108	chromodomain helicase DNA binding protein 4
CHD7	NM_017780	chr8	0.65	2.11E-02	55636	chromodomain helicase DNA binding protein 7
CHES1	XM_929939	chr14	0.77	4.10E-02	1112	checkpoint suppressor 1
CHKB	NM_005198	chr22	1.16	8.35E-04	1120	choline kinase beta
CHRM5	BC068528	chr15	0.85	2.69E-02	1133	cholinergic receptor, muscarinic 5
CLDN14	NM_012130	chr21	0.89	3.39E-02	23562	claudin 14
CLEC5A	NM_013252	chr7	1.21	0.00E+00	23601	C-type lectin domain family 5, member A
CLIC2	NM_001289	chrX	0.69	1.45E-02	1193	chloride intracellular channel 2
CLN5	NM_006493	chr13	0.89	2.29E-02	1203	ceroid-lipofuscinosis, neuronal 5
CNGA2	NM_005140	chrX	1.39	0.00E+00	1260	cyclic nucleotide gated channel alpha 2
CNP	AK124861	chr17	0.68	4.72E-02	1267	2',3'-cyclic nucleotide 3' phosphodiesterase
CNTN1	BC036569	chr12	1.04	7.66E-03	1272	contactin 1
CNTROB	NM_053051	chr17	0.8	2.69E-02	116840	centrobin, centrosomal BRCA2 interacting protein
COL17A1	NM_130778	chr10	0.76	4.10E-02	1308	collagen, type XVII, alpha 1
COL2A1	NM_033150	chr12	1.01	1.05E-02	1280	collagen, type II, alpha 1 (primary osteoarthritis, spondyloepiphyseal dysplasia, congenital)
COX15	NM_078470	chr10	1.54	0.00E+00	1355	COX15 homolog, cytochrome c oxidase assembly protein (yeast)
CPT1B	NM_152247	chr22	1.16	8.35E-04	1375	carnitine palmitoyltransferase 1B (muscle)
CRLF1	NM_004750	chr19	0.71	4.10E-02	9244	cytokine receptor-like factor 1
CROT	NM_021151	chr7	0.88	2.38E-02	54677	carnitine O-octanoyltransferase
CRY2	NM_021117	chr11	0.82	2.69E-02	1408	cryptochrome 2 (photolyase-like)

CTDP1	NM_048368	chr18	0.79	4.10E-02	9150	CTD (carboxy-terminal domain, RNA polymerase II, polypeptide A) phosphatase, subunit 1
CTF1	NM_001330	chr16	1.95	0.00E+00	1489	cardiotrophin 1
DAPK2	NM_014326	chr15	0.72	1.45E-02	23604	death-associated protein kinase 2
DCLRE1A	NM_014881	chr10	1.16	4.44E-03	9937	DNA cross-link repair 1A (PSO2 homolog, S. cerevisiae)
DCX	NM_178153	chrX	0.85	2.29E-02	1641	doublecortex%3B lissencephaly, X-linked (doublecortin)
DFNA5	NM_004403	chr7	0.85	4.21E-02	1687	deafness, autosomal dominant 5
DGKE	NM_003647	chr17	0.77	2.02E-02	8526	diacylglycerol kinase, epsilon 64kDa
DHX8	NM_004941	chr17	0.74	4.10E-02	1659	DEAH (Asp-Glu-Ala-His) box polypeptide 8
DNAJC5	NM_025219	chr20	1.2	8.35E-04	80331	DnaJ (Hsp40) homolog, subfamily C, member 5
DNASE1	NM_005223	chr16	0.95	7.66E-03	1773	deoxyribonuclease I
DPYD	NM_000110	chr1	1.26	0.00E+00	1806	dihydropyrimidine dehydrogenase
DRG1	NM_004147	chr22	0.86	3.39E-02	4733	developmentally regulated GTP binding protein 1
DTX3	NM_178502	chr12	0.85	2.69E-02	196403	deltex 3 homolog (Drosophila)
DTYMK	NM_012145	chr2	0.83	4.51E-02	1841	deoxythymidylate kinase (thymidylate kinase)
DUSP19	NM_080876	chr2	0.83	4.51E-02	142679	dual specificity phosphatase 19
DYNC2H1	BX538093	chr11	0.47	4.99E-02	79659	dynein, cytoplasmic 2, heavy chain 1
EDA	NM_001399	chrX	0.82	2.69E-02	1896	ectodysplasin A
EDA2R	NM_021783	chrX	0.69	1.75E-02	60401	ectodysplasin A2 receptor
EDEM1	NM_014674	chr3	1.29	0.00E+00	9695	ER degradation enhancer, mannosidase alpha-like 1
EDNRB	NM_003991	chr13	1.02	1.05E-02	1910	endothelin receptor type B
EGFR	NM_201284	chr7	0.82	0.00E+00	1956	epidermal growth factor receptor (erythroblastic leukemia viral (v-erb-b) oncogene homolog, avian)
EHF	NM_012153	chr11	0.82	2.69E-02	26298	ets homologous factor
EIF1AY	NM_004681	chrY	0.69	3.27E-02	9086	eukaryotic translation initiation factor 1A, Y-linked
ELAVL1	NM_001419	chr19	0.89	3.11E-02	1994	ELAV (embryonic lethal, abnormal vision, Drosophila)-like 1 (Hu antigen R)
ELF5	NM_198381	chr11	1.36	0.00E+00	2001	E74-like factor 5 (ets domain transcription factor)
ENAH	NM_018212	chr1	0.86	3.40E-02	55740	enabled homolog (Drosophila)
ENTPD4	BC034477	chr8	1.34	2.63E-02	9583	ectonucleoside triphosphate diphosphohydrolase 4
ENTPD7	NM_020354	chr10	0.85	4.48E-02	57089	ectonucleoside triphosphate diphosphohydrolase 7
EPB41L3	BC008377	chr18	0.88	2.29E-02	23136	erythrocyte membrane protein band 4.1-like 3
ERAF	NM_016633	chr16	0.74	3.27E-02	51327	erythroid associated factor
ESPL1	NM_012291	chr12	0.88	2.29E-02	9700	extra spindle poles like 1 (S. cerevisiae)
EVL	BC023997	chr14	0.84	2.69E-02	51466	Enah/Vasp-like
FANCA	NM_001018112	chr16	0.83	4.15E-02	2175	Fanconi anemia, complementation group A
FER1L3	NM_133337	chr10	1.35	0.00E+00	26509	fer-1-like 3, myoferlin (C. elegans)
FGF10	NM_004465	chr5	0.77	2.63E-02	2255	fibroblast growth factor 10
FHL1	NM_001449	chrX	0.79	3.27E-02	2273	four and a half LIM domains 1
FIGF	NM_004469	chrX	0.82	2.69E-02	2277	c-fos induced growth factor (vascular endothelial growth factor D)
FLOT2	BC017292	chr17	0.93	3.11E-02	2319	flotillin 2
FOXM1	NM_202003	chr12	1.53	0.00E+00	2305	forkhead box M1
FOXO3A	NM_201559	chr6	1.09	3.29E-03	2309	forkhead box O3A
FPRL2	BC069070	chr19	0.92	2.54E-02	2359	formyl peptide receptor-like 2
FTL	NM_000146	chr19	0.71	4.10E-02	2512	ferritin, light polypeptide
GABRA3	NM_000808	chrX	1.01	7.66E-03	2556	gamma-aminobutyric acid (GABA) A receptor, alpha 3
GABRE	Y07637	chrX	0.79	3.27E-02	2564	gamma-aminobutyric acid (GABA) A receptor, epsilon
GAD1	NM_013445	chr2	0.86	3.40E-02	2571	glutamate decarboxylase 1 (brain, 67kDa)
GALC	NM_001037525	chr14	0.87	2.29E-02	2581	galactosylceramidase
GALNT1	NM_020474	chr18	0.88	1.89E-02	2589	UDP-N-acetyl-alpha-D-galactosamine:polypeptide N-acetylgalactosaminyltransferase 1 (GalNAc-T1)

GALNT4	NM_003774	chr12	0.72	4.72E-02	8693	UDP-N-acetyl-alpha-D-galactosamine:polypeptide
						N-acetylgalactosaminyltransferase 4 (GalNAc-T4)
GALR1	NM_001480	chr18	0.92	1.91E-02	2587	galanin receptor 1
GBF1	NM_004193	chr10	0.76	4.10E-02	8729	golgi-specific brefeldin A resistance factor 1
GDF8	NM_005259	chr2	1	7.52E-03	2660	growth differentiation factor 8
GLYCAM1	XM_934129	chr12	0.75	7.52E-03	644076	glycosylation-dependent cell adhesion molecule 1
GLYCAM1	XM_934132	chr12	0.75	7.52E-03	644287	glycosylation-dependent cell adhesion molecule 1
GNA15	NM_002068	chr19	0.89	1.24E-02	2769	guanine nucleotide binding protein (G protein), alpha 15 (Gq class)
GNG2	BC020774	chr14	1.22	4.27E-03	54331	guanine nucleotide binding protein (G protein), gamma 2
GPC4	NM_001448	chrX	1.01	2.77E-03	2239	glypican 4
GPR1	NM_005279	chr2	0.83	4.51E-02	2825	G protein-coupled receptor 1
GPR143	NM_000273	chrX	1.07	1.92E-02	4935	G protein-coupled receptor 143
GRIK1	NM_175611	chr21	0.8	2.69E-02	2897	glutamate receptor, ionotropic, kainate 1
GRIN3A	NM_133445	chr9	0.87	2.38E-02	116443	glutamate receptor, ionotropic, N-methyl-D-aspartate 3A
GTF2E1	AK223401	chr3	0.99	1.17E-02	2960	general transcription factor IIE, polypeptide 1, alpha 56kDa
GTF3A	NM_002097	chr13	1.18	5.04E-03	2971	general transcription factor IIIA
GUCY2C	NM_004963	chr12	0.98	3.11E-02	2984	guanylate cyclase 2C (heat stable enterotoxin receptor)
GUCY2D	NM_000180	chr17	0.8	1.72E-02	3000	guanylate cyclase 2D, membrane (retina-specific)
HDAC3	NM_003883	chr5	0.84	4.51E-02	8841	histone deacetylase 3
HDAC6	NM_006044	chrX	0.98	1.05E-02	10013	histone deacetylase 6
HEY2	NM_012259	chr6	1.02	7.52E-03	23493	hairy/enhancer-of-split related with YRPW motif 2
HIRA	X77633	chr22	0.8	2.29E-02	7290	HIR histone cell cycle regulation defective homolog A (S. cerevisiae)
HNRPH1	NM_005520	chr5	0.97	1.17E-02	3187	heterogeneous nuclear ribonucleoprotein H1 (H)
HOMER2	NM_199332	chr15	1.08	3.76E-03	9455	homer homolog 2 (Drosophila)
HPS6	NM_024747	chr10	0.85	2.29E-02	79803	Hermansky-Pudlak syndrome 6
HPX	NM_000613	chr11	0.95	1.24E-02	3263	hemopexin
HSPA8	NM_153201	chr11	0.79	3.27E-02	3312	heat shock 70kDa protein 8
HSPG2	X62515	chr1	0.99	7.52E-03	3339	heparan sulfate proteoglycan 2 (perlecan)
HTATIP2	NM_006410	chr11	0.88	1.91E-02	10553	HIV-1 Tat interactive protein 2, 30kDa
HTR2A	NM_000621	chr13	1.12	5.68E-03	3356	5-hydroxytryptamine (serotonin) receptor 2A
HTRA1	NM_002775	chr10	0.79	3.27E-02	5654	HtrA serine peptidase 1
IFI16	AK094968	chr1	0.86	4.21E-02	3428	interferon, gamma-inducible protein 16
IFI30	NM_006332	chr19	0.86	3.39E-02	10437	interferon, gamma-inducible protein 30
IFNAR2	X89814	chr21	0.83	2.29E-02	3455	interferon (alpha, beta and omega) receptor 2
IFNE1	NM_176891	chr9	0.94	1.17E-02	338376	interferon epsilon 1
IFT88	NM_175605	chr13	1.05	2.09E-02	8100	intraflagellar transport 88 homolog (Chlamydomonas)
IL1A	BC013142	chr2	0.9	2.38E-02	3552	interleukin 1, alpha
ILK	NM_004517	chr11	0.91	1.56E-02	3611	integrin-linked kinase
IRAK1	NM_001569	chrX	0.85	2.29E-02	3654	interleukin-1 receptor-associated kinase 1
IRF1	NM_002198	chr5	1.04	3.95E-03	3659	interferon regulatory factor 1
ISG20	NM_002201	chr15	0.82	3.27E-02	3669	interferon stimulated exonuclease gene 20kDa
JUND	NM_005354	chr19	0.77	2.69E-02	3727	jun D proto-oncogene
KCNC2	NM_153748	chr12	1.04	2.09E-02	3747	potassium voltage-gated channel, Shaw-related subfamily, member 2
KCNE1	NM_000219	chr21	0.8	2.69E-02	3753	potassium voltage-gated channel, Isk-related family, member 1
KIAA0329	NM_014844	chr14	1.03	2.09E-02	9895	KIAA0329
KIAA0355	NM_014686	chr19	0.95	7.66E-03	9710	KIAA0355
KIAA0409	NM_015324	chr11	0.91	1.56E-02	23378	KIAA0409
KIAA0553	NM_00100290[illegible]	chr17	0.83	4.48E-02	23131	KIAA0553
KIAA0586	NM_014749	chr14	1.06	6.89E-03	9786	KIAA0586
KIAA0999	NM_025164	chr11	0.82	2.69E-02	23387	KIAA0999 protein
KIAA1128	BC030528	chr10	0.88	4.15E-02	54462	KIAA1128

KIAA1370	NM_019600	chr15	0.75	3.69E-02	56204	KIAA1370
KIAA1409	NM_020818	chr14	1.26	8.35E-04	57578	KIAA1409
KIAA1411	AK000183	chr6	1.02	7.52E-03	57579	KIAA1411
KIAA1632	BC036911	chr18	0.85	2.69E-02	57724	KIAA1632
KIAA1840	BC024161	chr15	0.92	4.15E-02	80208	KIAA1840
KIF4A	NM_012310	chrX	0.92	3.39E-02	24137	kinesin family member 4A
KLF4	NM_004235	chr9	0.81	4.51E-02	9314	Kruppel-like factor 4 (gut)
KLF9	NM_001206	chr9	0.84	3.40E-02	687	Kruppel-like factor 9
KLK6	NM_002774	chr19	1.22	5.01E-03	5653	kallikrein 6 (neurosin, zyme)
KLRD1	BC042884	chr12	1.01	1.05E-02	3824	killer cell lectin-like receptor subfamily D, member 1
LCP1	NM_002298	chr13	1.18	5.04E-03	3936	lymphocyte cytosolic protein 1 (L-plastin)
LECT1	NM_007015	chr13	1.25	4.27E-03	11061	leukocyte cell derived chemotaxin 1
LIAS	NM_194451	chr4	1.34	0.00E+00	11019	lipoic acid synthetase
LIG3	U40671	chr17	1.08	4.99E-03	3980	ligase III, DNA, ATP-dependent
LTA4H	NM_000895	chr12	0.85	2.69E-02	4048	leukotriene A4 hydrolase
LTB4R2	NM_019839	chr14	0.87	1.89E-02	56413	leukotriene B4 receptor 2
MAFF	NM_012323	chr22	0.74	3.27E-02	23764	v-maf musculoaponeurotic fibrosarcoma oncogene homolog F (avian)
MAP1A	NM_002373	chr15	0.95	1.02E-02	4130	microtubule-associated protein 1A
MAP3K7IP1	NM_153497	chr22	0.92	2.54E-02	10454	mitogen-activated protein kinase kinase kinase 7 interacting protein 1
MAP4	NM_030884	chr3	1.29	0.00E+00	4134	microtubule-associated protein 4
MAP4K1	NM_007181	chr19	1.16	8.35E-04	11184	mitogen-activated protein kinase kinase kinase kinase 1
MAPK8IP2	NM_139124	chr22	0.86	3.39E-02	23542	mitogen-activated protein kinase 8 interacting protein 2
MBD1	NM_015847	chr18	0.98	3.11E-02	4152	methyl-CpG binding domain protein 1
MBP	NM_002385	chr18	0.82	3.27E-02	4155	myelin basic protein
MCRS1	NM_006337	chr12	1.14	1.68E-02	10445	microspherule protein 1
MEFV	NM_000243	chr16	0.83	4.15E-02	4210	Mediterranean fever
MMP1	NM_002421	chr11	1.32	0.00E+00	4312	matrix metallopeptidase 1 (interstitial collagenase)
MMP12	NM_002426	chr11	0.85	2.29E-02	4321	matrix metallopeptidase 12 (macrophage elastase)
MMP20	NM_004771	chr11	0.88	1.28E-02	9313	matrix metallopeptidase 20 (enamelysin)
MMP9	NM_004994	chr20	0.92	1.24E-02	4318	matrix metallopeptidase 9 (gelatinase B, 92kDa gelatinase, 92kDa type IV collagenase)
MPP1	NM_002436	chrX	0.76	4.10E-02	4354	membrane protein, palmitoylated 1, 55kDa
MRPL51	NM_016497	chr12	0.85	2.69E-02	51258	mitochondrial ribosomal protein L51
MSI1	NM_002442	chr12	0.85	2.69E-02	4440	musashi homolog 1 (Drosophila)
MSR1	NM_138716	chr8	0.85	3.40E-02	4481	macrophage scavenger receptor 1
MT3	NM_005954	chr16	0.98	2.07E-02	4504	metallothionein 3 (growth inhibitory factor (neurotrophic))
MTCP1	NM_001018025	chrX	1.04	6.89E-03	4515	mature T-cell proliferation 1
MTSS1	AB007889	chr8	0.85	3.40E-02	9788	metastasis suppressor 1
NAGA	NM_000262	chr22	0.71	4.10E-02	4668	N-acetylgalactosaminidase, alpha-
NAPG	NM_003826	chr18	0.98	1.10E-02	8774	N-ethylmaleimide-sensitive factor attachment protein, gamma
NAV3	NM_014903	chr12	0.78	4.10E-02	89795	neuron navigator 3
NCOR1	AF303585	chr17	1.23	1.03E-03	9611	nuclear receptor co-repressor 1
NDFIP2	NM_019080	chr13	1.15	4.99E-03	54602	Nedd4 family interacting protein 2
NEBL	NM_213569	chr10	0.93	2.44E-02	10529	nebulette
NEDD8	NM_006156	chr14	0.71	1.45E-02	4738	neural precursor cell expressed, developmentally down-regulated 8
NFE2L3	AF125534	chr7	1.18	1.32E-02	9603	nuclear factor (erythroid-derived 2)-like 3
NFKBIB	NM_002503	chr19	0.86	1.56E-02	4793	nuclear factor of kappa light polypeptide gene enhancer in B-cells inhibitor, beta
NLGN4X	NM_181332	chrX	1.04	6.89E-03	57502	neuroligin 4, X-linked
NPM3	NM_006993	chr10	0.79	3.27E-02	10360	nucleophosmin/nucleoplasmin, 3
NUDT21	NM_007006	chr16	0.95	2.09E-02	11051	nudix (nucleoside diphosphate linked moiety X)-type motif 21
NUTF2	NM_005796	chr16	1.18	1.03E-03	10204	nuclear transport factor 2

NXF1	NM_006362	chr11		0.76	4.10E-02	10482	nuclear RNA export factor 1
ODZ1	NM_014253	chrX		0.63	3.46E-02	10178	odz, odd Oz/ten-m homolog 1(Drosophila)
OPCML	NM_00101239	chr11		0.88	1.91E-02	4978	opioid binding protein/cell adhesion molecule-like
OR1D2	NM_002548	chr17		0.86	4.15E-02	4991	olfactory receptor, family 1, subfamily D, member 2
OR5A1	NM_00100472	chr11		1.1	4.99E-03	219982	olfactory receptor, family 5, subfamily A, member 1
OR6A2	NM_003696	chr11		1.04	6.89E-03	8590	olfactory receptor, family 6, subfamily A, member 2
OSBPL1A	NM_018030	chr18		0.79	4.10E-02	114876	oxysterol binding protein-like 1A
OXGR1	NM_080818	chr13		1.05	7.66E-03	27199	oxoglutarate (alpha-ketoglutarate) receptor 1
P2RX3	NM_002559	chr11		1.42	0.00E+00	5024	purinergic receptor P2X, ligand-gated ion channel, 3
PABPN1	NM_004643	chr14		1.09	5.68E-03	8106	poly(A) binding protein, nuclear 1
PAPPA	NM_002581	chr9		0.91	2.44E-02	5069	pregnancy-associated plasma protein A, pappalysin 1
PAWR	NM_002583	chr12		0.82	3.27E-02	5074	PRKC, apoptosis, WT1, regulator
PAX1	NM_006192	chr20		0.8	2.69E-02	5075	paired box gene 1
PAX4	NM_006193	chr7		0.82	4.51E-02	5078	paired box gene 4
PCGF2	NM_007144	chr17		0.77	3.27E-02	7703	polycomb group ring finger 2
PDE1B	AJ401609	chr12		1.11	5.68E-03	5153	phosphodiesterase 1B, calmodulin-dependent
PDE2A	AY495087	chr11		0.88	1.91E-02	5138	phosphodiesterase 2A, cGMP-stimulated
PELP1	NM_014389	chr17		1.2	8.35E-04	27043	proline, glutamic acid and leucine rich protein 1
PEX5	Z48054	chr12		1.11	0.00E+00	5830	peroxisomal biogenesis factor 5
PFDN5	NM_145897	chr12		0.59	2.63E-02	5204	prefoldin subunit 5
PHC2	NM_198040	chr1		1.06	3.29E-03	1912	polyhomeotic-like 2 (Drosophila)
PHLDA1	NM_007350	chr12		0.72	4.72E-02	22822	pleckstrin homology-like domain, family A, member 1
PIK3C2A	NM_002645	chr11		0.95	3.11E-02	5286	phosphoinositide-3-kinase, class 2, alpha polypeptide
PIK3C3	NM_002647	chr18		1.28	8.35E-04	5289	phosphoinositide-3-kinase, class 3
PIK3CG	NM_002649	chr7		0.82	4.51E-02	5294	phosphoinositide-3-kinase, catalytic, gamma polypeptide
PIK4CA	AB210002	chr22		0.83	1.91E-02	5297	phosphatidylinositol 4-kinase, catalytic, alpha polypeptide
PIP5K1C	NM_012398	chr19		1.19	1.03E-03	23396	phosphatidylinositol-4-phosphate 5-kinase, type I, gamma
PITPNM1	NM_004910	chr11		1.23	8.35E-04	9600	phosphatidylinositol transfer protein, membrane-associated 1
PITX3	NM_005029	chr10		0.76	4.10E-02	5309	paired-like homeodomain transcription factor 3
PKM2	NM_182471	chr15		1.41	0.00E+00	5315	pyruvate kinase, muscle
PML	AF370432	chr15		0.82	3.27E-02	5371	promyelocytic leukemia
POLB	NM_002690	chr8		1.04	3.29E-03	5423	polymerase (DNA directed), beta
POLR2I	NM_006233	chr19		0.86	1.56E-02	5438	polymerase (RNA) II (DNA directed) polypeptide I, 14.5kDa
POLR3A	BC014399	chr10		0.82	2.69E-02	11128	polymerase (RNA) III (DNA directed) polypeptide A, 155kDa
POP5	NM_198202	chr12		0.78	2.87E-02	51367	processing of precursor 5, ribonuclease P/MRP subunit (S. cerevisiae)
POSTN	NM_006475	chr13		0.92	4.15E-02	10631	periostin, osteoblast specific factor
PPAP2C	NM_177543	chr19		0.89	1.24E-02	8612	phosphatidic acid phosphatase type 2C
PPARG	NM_015869	chr3		0.95	2.44E-02	5468	peroxisome proliferative activated receptor, gamma
PPM2C	BC098343	chr8		0.98	7.52E-03	54704	protein phosphatase 2C, magnesium-dependent, catalytic subunit
PPME1	AK123288	chr11		0.76	4.10E-02	51400	protein phosphatase methylesterase 1
PRC1	NM_199414	chr15		0.79	4.10E-02	9055	protein regulator of cytokinesis 1
PRF1	NM_005041	chr10		1.26	6.58E-03	5551	perforin 1 (pore forming protein)
PRKCB1	NM_212535	chr16		0.77	2.69E-02	5579	protein kinase C, beta 1
PRKCBP1	DQ082998	chr20		0.99	2.09E-02	23613	protein kinase C binding protein 1
PRMT8	AK026786	chr12		0.95	1.56E-02	56341	protein arginine methyltransferase 8
PSENEN	AF162447	chr19		0.89	1.24E-02	55851	presenilin enhancer 2 homolog (C. elegans)
PSMB3	NM_002795	chr17		0.77	3.27E-02	5691	proteasome (prosome, macropain) subunit, beta type, 3
PSMD9	NM_002813	chr12		0.98	1.24E-02	5715	proteasome (prosome, macropain) 26S subunit, non-ATPase, 9
PSME2	NM_002818	chr14		2.32	0.00E+00	5721	proteasome (prosome, macropain) activator subunit 2 (PA28 beta)
PTGDR	NM_000953	chr14		0.61	4.35E-02	5729	prostaglandin D2 receptor (DP)
PTGIR	NM_000960	chr19		1.1	1.42E-02	5739	prostaglandin I2 (prostacyclin) receptor (IP)

PTGIS	NM_000961	chr20	1.2	7.52E-03	5740	prostaglandin I2 (prostacyclin) synthase
PTPRO	NM_030667	chr12	0.82	2.02E-02	5800	protein tyrosine phosphatase, receptor type, O
PUS7L	NM_031292	chr12	0.78	4.10E-02	83448	pseudouridylate synthase 7 homolog (S. cerevisiae)-like
RAD51AP1	NM_006479	chr12	1.57	0.00E+00	10635	RAD51 associated protein 1
RARG	BC093727	chr12	1.11	5.68E-03	5916	retinoic acid receptor, gamma
RBBP8	NM_203292	chr18	0.82	3.27E-02	5932	retinoblastoma binding protein 8
RBM3	NM_006743	chrX	0.82	2.69E-02	5935	RNA binding motif (RNP1, RRM) protein 3
RDH12	NM_152443	chr14	1.38	0.00E+00	145226	retinol dehydrogenase 12 (all-trans and 9-cis)
RERG	NM_032918	chr12	0.88	4.48E-02	85004	RAS-like, estrogen-regulated, growth inhibitor
RGS13	NM_144766	chr1	0.83	4.51E-02	6003	regulator of G-protein signalling 13
RGS9	NM_003835	chr17	0.68	1.45E-02	8787	regulator of G-protein signalling 9
RIT2	U78166	chr18	1.11	5.68E-03	6014	Ras-like without CAAX 2
ROBO3	BC008623	chr11	1.32	0.00E+00	64221	roundabout, axon guidance receptor, homolog 3 (Drosophila)
RPA1	NM_002945	chr17	1.33	0.00E+00	6117	replication protein A1, 70kDa
RPS6KA6	NM_014496	chrX	0.85	2.29E-02	27330	ribosomal protein S6 kinase, 90kDa, polypeptide 6
SAFB	NM_002967	chr19	1.13	4.27E-03	6294	scaffold attachment factor B
SATB1	NM_002971	chr3	0.95	2.44E-02	6304	special AT-rich sequence binding protein 1 (binds to nuclear matrix/scaffold-associating DNA's)
SCGB1D4	NM_206998	chr11	1.04	2.07E-02	404552	secretoglobin, family 1D, member 4
SDHD	NM_003002	chr11	0.76	4.10E-02	6392	succinate dehydrogenase complex, subunit D, integral membrane protein
SEL1L	NM_005065	chr14	1.55	0.00E+00	6400	sel-1 suppressor of lin-12-like (C. elegans)
SERPINB7	NM_003784	chr18	1.05	7.66E-03	8710	serpin peptidase inhibitor, clade B (ovalbumin), member 7
SH2B	NM_015503	chr16	0.95	2.09E-02	25970	SH2-B homolog
SH2D1A	NM_002351	chrX	1.17	4.44E-03	4068	SH2 domain protein 1A, Duncan's disease (lymphoproliferative syndrome)
SIRT2	NM_030593	chr19	0.86	1.56E-02	22933	sirtuin (silent mating type information regulation 2 homolog) 2 (S. cerevisiae)
SLC3A2	NM_002394	chr11	0.85	2.29E-02	6520	solute carrier family 3 (activators of dibasic and neutral amino acid transport), member 2
SLC6A4	BC069484	chr17	1.14	4.44E-03	6532	solute carrier family 6 (neurotransmitter transporter, serotonin), member 4
SLITRK4	NM_173078	chrX	0.85	4.78E-03	139065	SLIT and NTRK-like family, member 4
SMARCC2	NM_139067	chr12	0.82	3.27E-02	6601	SWI/SNF related, matrix associated, actin dependent regulator of chromatin, subfamily c, member 2
SMS	NM_004595	chrX	0.95	1.24E-02	6611	spermine synthase
SNAP23	NM_130798	chr15	0.98	1.24E-02	8773	synaptosomal-associated protein, 23kDa
SNAP29	NM_004782	chr22	0.83	1.91E-02	9342	synaptosomal-associated protein, 29kDa
SNRPB2	NM_198220	chr20	0.77	3.27E-02	6629	small nuclear ribonucleoprotein polypeptide B''
SNRPG	NM_003096	chr2	0.86	3.40E-02	6637	small nuclear ribonucleoprotein polypeptide G
SNRPN	NM_022805	chr15	0.82	3.27E-02	6638	small nuclear ribonucleoprotein polypeptide N
SNX1	NM_152826	chr15	0.88	2.29E-02	6642	sorting nexin 1
SOX5	NM_006940	chr12	1.11	5.68E-03	6660	SRY (sex determining region Y)-box 5
SP8	NM_198956	chr7	0.95	1.17E-02	221833	Sp8 transcription factor
SPI1	NM_003120	chr11	1.2	4.27E-03	6688	spleen focus forming virus (SFFV) proviral integration oncogene spi1
SPIRE1	NM_020148	chr18	1.01	1.05E-02	56907	spire homolog 1 (Drosophila)
SRY	NM_003140	chrY	0.78	1.91E-02	6736	sex determining region Y
SSRP1	NM_003146	chr11	1.42	0.00E+00	6749	structure specific recognition protein 1
SSTR1	NM_001049	chr14	0.55	3.51E-02	6751	somatostatin receptor 1
SSTR2	NM_001050	chr17	0.49	4.33E-02	6752	somatostatin receptor 2
ST3GAL2	NM_006927	chr16	0.8	2.29E-02	6483	ST3 beta-galactoside alpha-2,3-sialyltransferase 2
ST6GALNAC	NM_018414	chr17	1.02	2.07E-02	55808	ST6 (alpha-N-acetyl-neuraminyl-2,3-beta-galactosyl-1,3)-N-acetylgalactosaminide alpha-2,6-sialyltransferase 1
ST8SIA1	NM_003034	chr12	1.04	7.66E-03	6489	ST8 alpha-N-acetyl-neuraminide alpha-2,8-sialyltransferase 1
STAT3	NM_213662	chr17	1.08	0.00E+00	6774	signal transducer and activator of transcription 3 (acute-phase response factor)

STXBP4	NM_178509	chr17	1.08	1.68E-02	252983	syntaxin binding protein 4
SULF1	AK074873	chr8	0.85	0.00E+00	23213	sulfatase 1
SURB7	NM_004264	chr12	1.21	1.42E-02	9412	SRB7 suppressor of RNA polymerase B homolog (yeast)
SYT7	NM_004200	chr11	0.85	4.48E-02	9066	synaptotagmin VII
TANK	NM_133484	chr2	0.9	2.38E-02	10010	TRAF family member-associated NFKB activator
TDG	NM_003211	chr12	1.14	4.99E-03	6996	thymine-DNA glycosylase
TEAD1	BC026959	chr11	1.01	7.66E-03	7003	TEA domain family member 1 (SV40 transcriptional enhancer factor)
TEF	NM_003216	chr22	0.74	3.27E-02	7008	thyrotrophic embryonic factor
TFAM	NM_003201	chr10	0.76	4.10E-02	7019	transcription factor A, mitochondrial
TFF2	NM_005423	chr21	0.74	4.10E-02	7032	trefoil factor 2 (spasmolytic protein 1)
TGFB1I1	NM_015927	chr16	0.74	3.27E-02	7041	transforming growth factor beta 1 induced transcript 1
THRAP1	NM_005121	chr17	0.89	1.56E-02	9969	thyroid hormone receptor associated protein 1
THY1	NM_006288	chr11	0.79	2.02E-02	7070	Thy-1 cell surface antigen
TIMM8B	NM_012459	chr11	0.76	4.10E-02	26521	translocase of inner mitochondrial membrane 8 homolog B (yeast)
TIMM9	NM_012460	chr14	1.06	6.89E-03	26520	translocase of inner mitochondrial membrane 9 homolog (yeast)
TIMP3	NM_000362	chr22	0.74	3.27E-02	7078	TIMP metallopeptidase inhibitor 3 (Sorsby fundus dystrophy, pseudoinflammatory)
TMED1	NM_006858	chr19	0.86	1.56E-02	11018	transmembrane emp24 protein transport domain containing 1
TMEM4	NM_014255	chr12	0.85	2.69E-02	10330	transmembrane protein 4
TMEM49	NM_030938	chr17	1.39	0.00E+00	81671	transmembrane protein 49
TMPO	NM_003276	chr12	1.01	1.05E-02	7112	thymopoietin
TNFAIP3	BC114480	chr6	0.88	3.40E-02	7128	tumor necrosis factor, alpha-induced protein 3
TNFRSF13B	NM_012452	chr17	0.77	3.27E-02	23495	tumor necrosis factor receptor superfamily, member 13B
TNFRSF19	NM_148957	chr13	1.02	1.05E-02	55504	tumor necrosis factor receptor superfamily, member 19
TNMD	NM_022144	chrX	0.76	4.10E-02	64102	tenomodulin
TOB2	NM_016272	chr22	0.71	4.10E-02	10766	transducer of ERBB2, 2
TOM1L1	NM_005486	chr17	1.2	0.00E+00	10040	target of myb1-like 1 (chicken)
TPD52L1	AF004427	chr6	1.09	3.29E-03	7164	tumor protein D52-like 1
TPH1	NM_004179	chr11	1.04	2.07E-02	7166	tryptophan hydroxylase 1 (tryptophan 5-monooxygenase)
TPM1	NM_00101802(	chr15	0.98	1.10E-02	7168	tropomyosin 1 (alpha)
TPP2	NM_003291	chr13	0.86	2.69E-02	7174	tripeptidyl peptidase II
TSSK2	NM_053006	chr22	0.98	6.89E-03	23617	testis-specific serine kinase 2
UBE2D1	NM_003338	chr10	0.94	1.24E-02	7321	ubiquitin-conjugating enzyme E2D 1 (UBC4/5 homolog, yeast)
UBE2N	NM_003348	chr12	0.91	1.91E-02	7334	ubiquitin-conjugating enzyme E2N (UBC13 homolog, yeast)
UHRF2	NM_152896	chr9	0.71	3.76E-02	115426	ubiquitin-like, containing PHD and RING finger domains, 2
USP11	U44839	chrX	1.14	5.04E-03	8237	ubiquitin specific peptidase 11
USP3	NM_006537	chr15	0.85	1.72E-02	9960	ubiquitin specific peptidase 3
VPS39	NM_015289	chr15	0.82	3.27E-02	23339	vacuolar protein sorting 39 (yeast)
WASF2	NM_006990	chr1	0.83	4.51E-02	10163	WAS protein family, member 2
WDR1	NM_017491	chr4	1	7.52E-03	9948	WD repeat domain 1
XRCC3	NM_005432	chr14	1.13	4.99E-03	7517	X-ray repair complementing defective repair in Chinese hamster cells 3
XRCC6BP1	NM_033276	chr12	0.82	3.27E-02	91419	XRCC6 binding protein 1
YWHAH	NM_003405	chr22	0.74	3.27E-02	7533	tyrosine 3-monooxygenase/tryptophan 5-monooxygenase activation protein, eta polypeptide
ZBTB32	NM_014383	chr19	0.77	4.05E-03	27033	zinc finger and BTB domain containing 32
ZDHHC1	NM_013304	chr16	1.21	5.01E-03	29800	zinc finger, DHHC-type containing 1
ZDHHC13	NM_019028	chr11	0.91	3.39E-02	54503	zinc finger, DHHC-type containing 13
ZDHHC7	BC017702	chr16	0.74	3.27E-02	55625	zinc finger, DHHC-type containing 7
ZIC3	NM_003413	chrX	0.88	1.91E-02	7547	Zic family member 3 heterotaxy 1 (odd-paired homolog, Drosophila)
ZNF177	NM_003451	chr19	0.8	2.29E-02	7730	zinc finger protein 177
ZNF350	NM_021632	chr19	0.83	1.91E-02	59348	zinc finger protein 350
ZNF382	NM_032825	chr19	1.01	5.68E-03	84911	zinc finger protein 382

Supporting Information Table S4. Heatmap derived from gene microarray data of EZH2 regulated genes in hMSCs

(log2 (Ratio) > 1 was considered confidence increased expression)

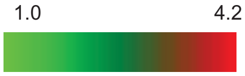

log2 (Ratio)	Name	accession
1.09	ACAT1	NM_000019.3
1.15	PIGA	NM_020473.2,NM_002641.2
2.04	CPA3	NM_001870.2
1.21	EPCAM	NM_002354.2
1.20	CSTB	NM_000100.2
1.45	MAPK3	NM_002746.2,NM_001040056.1,NM_001109891.1
1.32	DNAJC10	NM_018981.1
1.65	ACP5	NA
1.38	IL11	NM_000641.2
1.21	CCNF	NM_001761.2
1.15	PTX3	NM_002852.3
1.53	CEBPG	NM_001806.2
1.01	VSNL1	NM_003385.4
1.28	CASP1	NM_033293.2,NM_033292.2,NM_001223.3
1.34	PKN1	NM_213560.1,NM_002741.3
1.10	ALDH9A1	NM_000696.3
1.26	JUND	NM_005354.4
1.31	ST6GALNAC4	NM_175039.3,NM_175040.3
1.07	KLHL24	NM_017644.3
1.20	XPR1	NA
1.56	BHLHB9	NA
1.91	FAM49A	NM_030797.2
1.15	MGAT5	NA
1.07	SRGAP2	NM_015326.2
1.20	UBE2T	NM_014176.3
1.78	ZZZ3	NM_015534.4
1.11	SPATA5L1	NM_024063.2
1.43	SACM1L	NM_014016.3
1.79	DNAJB9	NM_012328.1
1.08	PAFAH1B3	NA
1.79	ACBD3	NM_022735.3
1.91	RND3	NM_005168.3
1.37	DSTYK	NM_199462.2,NM_015375.2
1.06	SEMA4C	NM_017789.4
1.65	MAD1L1	NM_001013837.1,NM_001013836.1,NM_003550.2
1.12	ATP6V0B	NM_004047.3,NM_001039457.1
1.15	MFSD1	NM_022736.1
1.04	FUNDC1	NM_173794.3
1.02	C19orf25	NM_152482.2
1.73	LRRC20	NM_018239.2,NM_018205.2,NM_207119.1
1.10	SLC25A38	NM_017875.2
1.63	ANGPTL2	NM_012098.2
1.55	SMYD3	NM_022743.1
1.46	PISD	NM_014338.3
1.03	TM9SF3	NM_020123.3
1.66	RPP25	NM_017793.2
1.29	UBAC1	NM_016172.2
1.01	TMEM147	NM_032635.2
2.34	CRELD1	NM_015513.3,NM_001031717.2,NM_001077415.1
1.25	TOM1	NA
1.69	GNA13	NM_006572.4
2.30	ARMCX1	NM_016608.1
2.07	KIDINS220	NM_020738.2
1.14	SEC61G	NM_001012456.1,NM_014302.3
1.16	EMP3	NM_001425.2
1.97	SLC38A7	NM_018231.1
1.27	DIRC2	NM_032839.2
1.15	YTHDF1	NM_017798.3
1.72	DHCR7	NM_001360.2
1.41	HMGCR	NA
1.21	ASGR1	NM_001671.3
1.24	C5orf26	NA
1.26	SRPRB	NM_021203.2
1.36	C3orf39	NM_032806.4
1.41	EML1	NM_001008707.1,NM_004434.2
1.16	PTDSS2	NM_030783.1
1.85	SH2B3	NM_005475.2
1.25	UVRAG	NM_003369.3
1.13	KLHDC8B	NM_173546.1
1.26	SCO1	NM_004589.2
1.73	IFI30	NM_006332.3
1.63	GPR137B	NM_003272.3
1.18	GALNT14	NM_024572.2
2.47	PODXL	NM_005397.3,NM_001018111.2
1.12	USP31	NM_020718.3
1.06	CRBN	NM_016302.2
1.22	ZDHHC18	NM_032283.1
1.82	ATP6V1H	NM_015941.2,NM_213620.1,NM_213619.1
1.06	C16orf35	NM_001039476.1,NM_001077350.1
1.17	MAMLD1	NM_005491.2
1.14	CDK5RAP3	NM_176096.1
1.29	DUSP16	NA
1.64	COL4A3BP	NA
1.12	UBN1	NM_001079514.1,NM_016936.3
1.27	PPM1H	NM_020700.1
1.13	FAM134A	NM_024293.4
1.32	PEO1	NM_021830.3
1.29	CCDC104	NM_080667.5
1.56	KIFC3	NA
1.77	EML2	NM_012155.1
1.51	TAF4	NM_003185.3
1.34	MOAP1	NM_022151.4
1.26	PPAP2C	NM_177543.1,NM_177526.1,NM_003712.2
1.07	TOLLIP	NM_019009.2
1.22	FOXJ3	NM_014947.3
1.13	TRPS1	NM_014112.2
1.23	DSCR3	NM_006052.1
1.00	KIAA0649	NM_014811.3
1.33	DPAGT1	NM_001382.3
1.23	C20orf100	NM_001098798.1,NM_001098797.1,NM_032883.2,NM_001098796.1
2.97	PGM2L1	NM_173582.3
1.41	ABCA3	NM_001089.2
1.35	FAM108C1	NM_021214.1
1.13	CECR5	NM_033070.2,NM_017829.5
1.63	FAM174B	NM_207446.2
1.23	APPL	NM_012096.2
1.09	MID1	NM_000381.2,NM_001098624.1,NM_033290.2
1.27	PATZ1	NM_032052.1,NM_032050.1,NM_014323.2
1.19	FNDC4	NM_022823.2
2.44	CRELD2	NA
1.50	KIAA0753	NM_014804.2
1.24	DDRGK1	NM_023935.1
1.36	UBP1	NA
1.37	SAT1	NM_002970.2
1.85	EXPH5	NA
1.13	ANKRD50	NM_020337.1
1.66	HSPBAP1	NM_024610.4
1.13	GBL	NM_022372.3
1.53	ETNK1	NM_018638.4
1.37	ZUFSP	NM_145062.1
1.06	RC3H1	NM_172071.2
1.40	GAB2	NM_080491.1,NM_012296.2
1.12	PMVK	NM_006556.3

1.45	SESTD1	NM_178123.4
1.10	MTF2	NM_007358.3
1.31	SCAMP1	NM_004866.4
1.05	C1orf128	NM_020362.4
1.38	USP42	NM_032172.2
1.45	SEMA3F	NM_004186.3
1.07	AC093283.3-1	NA
1.14	ZBTB4	NA
1.23	TSPAN1	NM_005727.2
1.27	FJX1	NM_014344.3
1.24	C2orf69	NM_153689.5
1.52	Z83826.13-1	NA
1.16	F2RL2	NM_004101.2
1.06	CLN6	NM_017882.2
1.42	CPEB2	NM_182646.1,NM_182485.1
1.42	DNAJC6	NM_014787.2
1.69	C20orf58	NM_152864.3
1.37	IFFO1	NM_080730.2,NM_001039670.1,NM_080731.2
1.40	SCFD2	NM_152540.3
1.00	ZFP3	NM_153018.2
1.21	SOCS1	NM_003745.1
1.32	NAGLU	NM_000263.3
1.15	VPS13A	NM_033305.2,NM_001018037.1
1.80	TUB	NM_177972.2,NM_003320.4
1.10	ABCC10	NM_033450.2
1.20	NKX3-1	NM_006167.3
1.18	RRP15	NM_016052.3
1.31	RAB33A	NM_004794.2
1.16	PIP5K2A	NM_005028.4
1.13	MCM2	NM_004526.2
2.97	STEAP3	NM_018234.2,NM_001008410.1,NM_182915.2
1.06	KEAP1	NM_203500.1,NM_012289.3
1.25	PFTK1	NM_012395.2
1.26	RG9MTD1	NM_017819.2
1.49	RHBDF1	NM_022450.3
1.82	PLEKHF1	NM_024310.4
1.22	ROCK2	NM_004850.3
1.21	VMA21	NM_001017980.2
1.13	TIGD2	NM_145715.2
1.02	SLC9A6	NM_006359.2,NM_001042537.1
1.80	ATAD2B	NM_017552.1
1.30	ACBD6	NM_032360.3
1.12	NT5C	NM_014595.1
1.15	SPHK1	NA
2.39	LY96	NM_015364.3
1.44	C16orf51	NM_015421.3
1.05	CDKN2B	NM_078487.2,NM_004936.3
1.31	FVT1	NM_002035.2
1.47	CASP4	NM_033306.2,NM_001225.3
1.78	NOC3L	NM_022451.9
1.05	TSN	NM_004622.2
1.02	MAPKAPK2	NM_032960.2,NM_004759.3
1.35	TOMM20	NM_014765.2
1.41	PPP3CC	NM_005605.3
1.01	PTPRF	NM_002840.3,NM_130440.2
1.12	MDH1	NM_005917.2
2.57	QDPR	NM_000320.2
1.29	DYRK1A	NM_130438.2,NM_130437.2,NM_101395.2,NM_001396.3,NM_130436.2
2.07	IGF2R	NM_000876.2
1.32	SNCA	NA
1.24	SERTAD2	NM_014755.2
1.11	PRPSAP1	NM_002766.2
1.06	COX10	NM_001303.3
1.46	ITPR3	NM_002224.2
1.37	RAB5B	NM_002868.2
1.54	VPS37B	NM_024667.2
1.09	SMARCA4	NA
1.13	VEGFB	NM_003377.3
1.27	HSPB2	NM_001541.3
1.85	NFIL3	NM_005384.2
1.16	LGALS3BP	NM_005567.3
1.49	RGS7	NM_002924.4
1.30	FBN2	NM_001999.3
1.78	CLCN7	NA
1.52	S100A4	NM_002961.2,NM_019554.2
1.23	LPIN1	NM_145693.1
1.03	ZCCHC14	NM_015144.2
3.17	KIT	NM_000222.2,NM_001093772.1
1.35	GYPC	NM_016815.2,NM_002101.3
3.52	STX3	NM_004177.3
1.14	GART	NA
1.58	PHF16	NM_001077445.1,NM_014735.3
1.41	WASF3	NM_006646.5
1.73	GPD1L	NM_015141.2
1.20	MMP1	NA
1.17	EFR3A	NM_015137.3
1.26	SUMO3	NM_006936.2
1.92	ZFP36L1	NM_004926.2
1.21	MLF1	NA
1.54	LIPA	NA
1.33	ATP6V1C1	NM_001695.4
1.37	NFAT5	NA
1.85	EVC2	NM_147127.3
1.48	DICER1	NM_030621.3,NM_177438.2
1.06	WDR43	NM_015131.1
1.10	PFN2	NM_053024.3,NM_002628.4
1.00	CLCC1	NM_001048210.1,NM_015127.3
3.04	PDIA4	NM_004911.4
1.49	AKAP3	NM_006422.2
1.46	HOXB6	NM_018952.4
1.32	CEBPB	NM_005194.2
1.30	ALOX5AP	NM_001629.2
1.56	TMEM22	NM_001097599.1,NM_025246.2,NM_001097600.1
1.17	DENND1B	NM_001142795.1
1.09	ELOVL4	NM_022726.3
1.64	TULP4	NM_001007466.1,NM_020245.3
1.00	ZNF593	NM_015871.4
1.01	MCTS1	NA
1.32	FNDC3A	NM_014923.3,NM_001079673.1
1.16	UPF3B	NM_080632.1,NM_023010.2
1.74	CYLN2	NM_032421.2,NM_003388.4
1.50	GMDS	NM_001500.2
1.05	SLK	NM_014720.2
1.45	UGCGL1	NM_020120.3
1.14	INTS3	NM_023015.3
2.22	SUPV3L1	NM_003171.3
1.05	GDE1	NM_016641.3
1.04	ATP5L	NM_006476.4
1.17	B4GALT5	NM_004776.3
1.58	C1orf9	NM_016227.2,NM_014283.3
1.22	RAB38	NM_022337.1
1.09	GLG1	NA
2.07	RRAGC	NM_022157.2
1.74	PANK1	NM_148978.1,NM_148977.1,NM_138316.2
1.06	PRTG	NM_173814.4
1.38	AIFM2	NM_032797.4
2.16	SLC17A5	NM_012434.4
1.37	CTSA	NA
1.30	TIMELESS	NM_003920.3

1.06	FLAD1	NM_201398.1,NM_025207.3
1.13	FUT8	NM_178157.1,NM_178156.1,NM_178155.1,NM_178154.1,NM_004480.3
1.14	AC011322.29	NA
1.65	C20orf194	NM_001009984.1
1.26	C12orf49	NM_024738.1
1.32	ZNF330	NM_014487.4
1.03	SHCBP1	NM_024745.4
1.56	PCF11	NM_015885.3
1.03	ACCS	NA
1.14	UBXN7	NM_015562.1
1.37	DOK5	NM_018431.3
2.60	F2R	NM_001992.3
1.01	DAGLB	NA
1.45	POMT2	NM_013382.5
1.39	MTMR4	NM_004687.4
1.96	UAP1L1	NM_207309.2
1.24	WDR4	NM_018669.4
1.22	GTF2E1	NM_005513.2
1.56	HERPUD1	NM_001010990.1,NM_001010989.1,NM_014685.2
1.99	CTDSPL	NM_001008392.1,NM_005808.2
1.14	ELF4	NA
1.20	MAP3K5	NM_005923.3
1.19	FZD5	NM_003468.3
1.40	MTHFR	NM_005957.3
1.62	INPP4B	NM_001101669.1,NM_003866.2
1.11	MTRR	NM_002454.2,NM_024010.2
1.12	CALCOCO2	NM_005831.3
1.80	TRAK2	NM_015049.2
1.19	F2RL1	NM_005242.4
1.39	EDEM1	NM_014674.2
1.04	SS18L1	NM_198935.1
1.56	GNB5	NM_006578.3,NM_016194.3
1.11	FAM53B	NM_014661.3
1.17	VAT1	NM_006373.3
1.08	RHEBL1	NM_144593.1
1.06	UBE2E1	NM_003341.3,NM_182666.1
1.28	IL15	NM_172174.2,NM_000585.3
1.91	C9orf91	NM_153045.3
1.34	PACS2	NM_001100913.1,NM_015197.2
1.90	SEMA7A	NA
1.20	PVR	NA
1.10	DPYSL2	NM_001386.4
1.34	LRRC8A	NA
1.17	USP33	NM_015017.3,NM_201624.1
2.26	TMEM41B	NM_015012.1
1.14	WDR36	NM_139281.2
1.45	C18orf45	NM_032933.4
1.17	MAPK13	NM_002754.3
1.63	AYTL2	NM_024830.3
2.19	LONRF1	NM_152271.3
1.25	CHCHD1	NM_203298.2
2.22	SEL1L	NA
1.52	MLEC	NM_014730.2
1.22	IFIT3	NM_001549.4,NM_001031683.2
1.76	SQSTM1	NA
2.10	LRPPRC	NM_133259.3
1.87	PPARGC1A	NM_013261.3
2.56	HMOX1	NM_002133.1
1.02	HECTD1	NM_015382.2
1.20	SEC24A	NM_021982.1
1.06	TXN2	NM_012473.3
1.00	PAPD4	NA
1.02	RAB31	NM_006868.3
1.12	WIPI2	NM_001033520.1,NM_016003.3,NM_015610.3,NM_001033519.1,NM_001033518.1
1.96	FKBP2	NA
1.06	TMEM203	NM_053045.1
1.97	WBP2	NM_012478.3
1.80	FICD	NM_007076.2
1.26	SLC15A3	NM_016582.2
1.19	C6orf115	NM_021243.2
1.04	RMND1	NM_017909.2
1.44	KCTD3	NM_016121.3
1.61	DGKE	NM_003647.2
1.13	GNPTG	NM_032520.3
2.32	TIMP3	NM_000362.4
1.21	TRPC1	NM_003304.4
1.11	AC016582.2	NA
2.34	C7orf57	NM_001100159.1
1.57	DNAJC12	NM_201262.1,NM_021800.2
1.03	CRISPLD2	NM_031476.3
2.17	RSG17	NM_012419.4
1.81	APOC1	NM_001645.3
1.07	SLC16A8	NM_013356.2
1.72	LMBR1L	NM_018113.2
1.47	GFM1	NM_024996.5
1.13	KATNB1	NM_005886.2
2.38	HYOU1	NA
2.21	GNPDA1	NM_005471.4
1.54	KRT86	NM_002284.3
1.65	EIF4EBP2	NM_004096.4
1.08	GLOD4	NM_016080.3
1.69	TRPV2	NM_016113.3
1.69	CHMP4A	NM_014169.2
1.36	SDR39U1	NM_020195.2
1.35	SLC25A37	NM_016612.2
1.63	SLC33A1	NM_004733.2
1.08	DDX55	NM_020936.1
1.07	TM2D3	NM_025141.3,NM_078474.2
1.18	TMEM8	NM_021259.2
1.05	COQ10B	NM_025147.3
1.29	ESCO1	NM_052911.2
1.02	ZFYVE27	NM_001002262.2,NM_001002261.2,NM_144588.5
1.01	DEDD2	NM_133328.2
1.03	BOK	NM_032515.3
1.35	C17orf63	NM_018182.2,NM_001077498.1
2.24	MLPH	NM_024101.5,NM_001042467.1
1.22	RPRD1A	NM_018170.3
1.47	SEH1L	NM_001013437.1,NM_031216.3
1.53	FZD8	NM_031866.2
2.00	SDF2L1	NM_022044.2
1.51	TMEM222	NM_032125.2
1.26	TOMM40L	NM_032174.4
1.26	MAPKAP1	NM_024117.3,NM_001006617.1,NM_001006619.1,NM_001006621.1,NM_001006620.1
1.05	DCTN4	NA
1.36	ORAI3	NM_152288.2
1.05	ACY1	NM_000666.1
1.13	FANCI	NA
1.17	C9orf30	NM_003692.3
1.44	CTSD	NM_001909.3
1.34	RNFT2	NM_001109903.1,NM_032814.3
1.27	NR2F2	NA
1.14	ZNF618	NM_133374.2
1.64	THAP8	NM_152658.2
1.31	LONP1	NM_004793.2
1.04	ATP6V0E2	NM_145230.2
1.30	FAM69B	NM_152421.3
2.19	CALR	NM_004343.3
1.87	CYB561	NM_001017917.1,NM_001017916.1,NM_001915.3
1.35	COMTD1	NM_144589.2

1.05	MIAT	NA
1.48	MRPL52	NM_181307.2,NM_181306.2,NM_181305.2,NM_181304.2,NM_180982.2,NM_178336.2
1.16	SRGAP1	NM_020762.2
1.13	MAPK8IP2	NM_012324.3,NM_016431.3
1.63	NHLRC3	NM_001017370.1,NM_001012754.2
1.15	TMCC1	NA
1.83	ARL1	NM_001177.3
1.32	ANKS1B	NM_181670.2
1.04	UBE2H	NM_003344.2,NM_182697.1
1.20	RAPGEF2	NM_014247.2
1.68	FAM73A	NM_198549.2
1.16	PRPF4	NM_004697.3
1.10	DUSP15	NM_001012644.1,NM_177991.1,NM_080611.3
1.10	CHCHD5	NM_032309.2
1.62	TSEN54	NM_207346.2
1.27	MFHAS1	NM_004225.2
1.24	TMEM136	NM_174926.1
1.39	PPP2R2D	NM_001003656.1,NM_018461.2
1.02	DDAH1	NA
1.94	SGK3	NM_001033578.1,NM_170709.1,NM_013257.3
1.82	PPIF	NM_005729.3
1.72	FAM26F	NM_001010919.1
1.24	C6orf1	NM_178508.3,NM_001008704.1,NM_001008703.1
1.46	PSAP	NM_001042466.1,NM_002778.2,NM_001042465.1
1.21	SPRY4	NA
1.87	SUNC1	NM_001030019.1,NM_152782.3
1.66	MAP1LC3A	NM_032514.2,NM_181509.1
1.01	ATG10	NA
1.58	HEXA	NM_000520.4
1.23	SLC31A1	NM_001859.3
1.33	KCNS3	NM_002252.3
1.06	C11orf75	NM_020179.2
1.26	POP7	NM_005837.2
1.01	C16orf14	NM_138418.2
1.19	AC021649.18	NM_033168.2,NM_033167.2,NM_033169.2,NM_001038628.1,NM_003781.3
1.30	RBP7	NM_052960.2
1.77	ARAP2	NM_015230.2
1.16	TATDN1	NA
1.37	G0S2	NM_015714.3
1.79	HDHD1A	NA
1.23	EXOSC6	NM_058219.2
1.04	MAPK14	NA
1.45	CHST11	NM_018413.4
1.21	NPC2	NM_006432.3
1.82	CFDP1	NM_006324.2
1.42	PITPNA	NM_006224.3
1.08	LAMP2	NA
1.10	IL17D	NM_138284.1
1.06	GFOD1	NA
1.41	SLC5A6	NM_021095.1
2.02	RASL10B	NM_033315.3
1.57	MAN2A1	NM_002372.2
1.29	C1orf112	NM_018186.2
2.21	DYSF	NA
1.73	DNAJC3	NM_006260.3
1.14	EVC	NA
1.87	AC004917.2	NM_175884.3
1.03	DPP7	NM_013379.2
1.34	ABHD6	NM_020676.5
1.20	SEC11L3	NM_033280.2
1.07	RAP2A	NM_021033.6
2.21	HEXB	NM_000521.3
1.34	TSC1	NA
1.36	MYST3	NM_001099413.1,NM_006766.3,NM_001099412.1
1.93	SLC43A3	NM_014096.2,NM_199329.1,NM_017611.2
3.65	APOE	NM_000041.2
1.06	OPA1	NM_130837.2,NM_130836.2,NM_130835.2,NM_130834.2,NM_130833.2,NM_130832.2,NM_130831.2,NM_015560.2
1.20	PES1	NM_014303.2
1.60	SLC38A1	NM_001077484.1,NM_030674.3
1.05	MAP2K1	NM_002755.3
1.09	TPM1	NM_001018005.1,NM_000366.5
1.52	NUP188	NM_015354.1
1.31	PYGB	NM_002862.3
1.28	LEPROTL1	NM_015344.2
1.31	ABCA1	NM_005502.2
1.24	LRFN4	NM_024036.4
1.39	ADARB1	NA
1.53	CNDP2	NM_018235.1
1.50	HOXB5	NM_002147.3
1.15	CADM1	NM_001098517.1,NM_014333.3
1.03	ANKRD10	NM_017664.2
1.08	SASH1	NM_015278.3
1.08	PLEK2	NM_016445.1
1.07	MALAT1	NA
2.13	CTNS	NM_001031681.2,NM_004937.2
1.07	MOSC1	NM_022746.3
1.34	RNF213	NM_020914.3
1.28	LRRC8D	NA
1.14	SLC38A6	NM_153811.1
1.21	PAPOLA	NM_032632.3
1.32	SETBP1	NM_015559.2
1.80	C1orf85	NM_144580.1
1.19	APEH	NM_001640.3
1.01	MEGF9	NM_001080497.1
1.00	SPG21	NA
1.31	GCHFR	NM_005258.2
1.40	WEE1	NA
1.93	NEDD4L	NA
1.32	GUSB	NM_000181.2
1.07	TSPAN3	NM_198902.1,NM_005724.4
1.29	LAMA1	NM_005559.2
1.60	CUL4A	NM_001008895.1,NM_003589.2
1.11	ANTXR1	NM_032208.2
1.11	HACE1	NM_020771.3
1.11	C9orf97	NM_139246.4
1.57	AGPAT5	NM_018361.3
1.12	PYGL	NM_002863.3
2.08	HS1BP3	NM_022460.3
1.58	DCTN2	NM_006400.3
1.12	LETM2	NM_144652.1
1.24	TRIM33	NM_033020.2,NM_015906.3
1.06	UTP20	NM_014503.2
1.35	AUTS2	NA
1.45	DUSP6	NM_022652.2,NM_001946.2
1.62	C16orf62	NM_020314.4
1.32	TUBB2B	NM_178012.4
1.28	KLRAQ1	NA
1.94	MME	NM_007289.2,NM_007288.2,NM_000902.3,NM_007287.2
1.24	TMEM180	NM_024789.3
4.11	HSPA5	NM_005347.3
2.12	AQP11	NM_173039.1
1.09	FOXRED1	NM_017547.2
1.22	MPND	NA
1.49	TRAF7	NM_032271.2
1.06	ARVCF	NM_001670.1
1.54	REPS1	NA
1.43	QPRT	NM_014298.3
1.12	SUZ12	NM_015355.2
2.16	TRIB3	NM_021158.3

1.04	AKAP13	NM_144767.3,NM_007200.3,NM_006738.4
1.27	TBC1D24	NA
1.44	PTPRK	NA
1.43	ZBTB43	NA
1.76	XIAP	NM_001167.2
1.33	OLFML2A	NM_182487.2
1.23	BIRC6	NM_016252.3
1.20	DOCK9	NA
1.49	PAQR3	NM_001040202.1
1.52	THUMPD2	NM_025264.3
1.73	TBX3	NM_016569.3,NM_005996.3
1.18	STARD13	NM_178007.2,NM_052851.2,NM_178006.3
1.09	AP2B1	NM_001030006.1,NM_001282.2
1.54	ATG2A	NM_015104.2
1.25	AC018450.26-1	NM_001080415.1
1.35	ATP13A3	NM_024524.3
1.92	FAM126B	NM_173822.3
1.35	CLCN3	NM_173872.2,NM_001829.2
2.68	HMGA2	NM_003483.4
1.78	APAF1	NM_013229.2,NM_001160.2,NM_181869.1,NM_181868.1,NM_181861.1
1.48	KIF21A	NM_017641.2
1.33	ZC3H12C	NM_033390.1
1.33	CAD	NM_004341.3
1.05	FNIP2	NM_020840.1
1.38	ZHX3	NM_015035.3
1.25	CDS1	NM_001263.3
1.08	NIN	NM_020921.3
1.56	BMF	NM_001003943.1,NM_001003942.1,NM_033503.3,NM_001003940.1
1.37	MTM1	NM_000252.2
1.34	SLC35D2	NM_007001.2
1.10	ICAM1	NM_000201.2
1.20	NMB	NM_205858.1,NM_021077.3
1.67	NEK6	NA
1.20	FOXRED2	NM_001102371.1,NM_024955.5
2.04	PPARG	NM_005037.5
1.21	SART2	NM_013352.2,NM_001080976.1
1.28	TSR1	NM_018128.4
1.09	OPTN	NM_021980.4,NM_001008213.1,NM_001008212.1,NM_001008211.1
1.09	RABEP1	NM_004703.4,NM_001083585.1
1.02	PDE4DIP	NM_022359.4
1.20	UHRF2	NM_152896.1
1.37	SLC25A19	NA
1.16	APBB1IP	NM_019043.3
1.45	FBXO32	NM_148177.1,NM_058229.2
1.13	PTPN3	NA
1.46	P2RX5	NM_175080.1,NM_002561.2
1.22	DPY19L1	NM_015283.1
1.33	ATP6V1A	NM_001690.2
2.21	SLC40A1	NM_014585.5
1.31	SAPS1	NM_014931.3
1.26	HYI	NM_031207.2
1.20	COL18A1	NA
1.09	WDR59	NM_030581.3
1.08	ASPH	NM_032466.2,NM_032468.3
1.46	HIRA	NM_003325.3
1.09	DUSP12	NM_007240.1
1.33	E2F7	NM_203394.2
1.24	VPS39	NM_015289.2
1.48	C9orf5	NM_001099734.1,NM_032012.3
1.22	APPBP1	NM_001018160.1,NM_001018159.1,NM_003905.3
1.64	JAM3	NM_032801.3
1.69	FBXO30	NM_032145.4
1.54	C11orf59	NM_017907.1
1.44	HERPUD2	NM_022373.4
2.65	TPP1	NM_000391.3
1.01	CHD1L	NM_004284.3
1.35	RELN	NM_173054.2,NM_005045.3
1.56	TPP2	NM_003291.2
1.06	ESD	NM_001984.1
2.03	ASRGL1	NM_001083926.1,NM_025080.3
1.65	ETV4	NM_001986.2,NM_001079675.1
2.47	C3orf52	NM_024616.2
1.92	RNF157	NM_052916.2
1.11	FANCG	NM_004629.1
1.02	DNAJC27	NM_016544.1
1.77	DENND5B	NM_144973.3
1.07	HOXB7	NM_004502.3
1.79	TMEM192	NM_001100389.1
1.15	EXOC2	NM_018303.4
1.14	AFG3L2	NM_006796.1
1.68	CLOCK	NM_004898.2
1.48	ATP10D	NM_020453.3
1.98	LAMA4	NM_001105207.1,NM_001105206.1,NM_002290.3
1.27	CDK5RAP2	NM_001011649.1,NM_018249.4
1.02	PTPRE	NM_006504.4,NM_130435.3
1.65	ENTPD6	NA
1.68	WDR19	NM_025132.3
1.08	NECAP1	NM_015509.3
1.53	COL11A1	NM_080629.2,NM_080630.2,NM_001854.3
1.17	FAM96A	NM_001014812.1,NM_032231.4
1.41	SERP1	NM_014445.3
1.14	USP47	NM_017944.3
1.31	ADM	NM_001124.1
1.05	PEX19	NA
1.09	CYB5B	NM_030579.2
1.28	MYLIP	NM_013262.3
1.01	SYNE2	NM_182914.2,NM_015180.4,NM_182913.2,NM_182910.2
1.06	SLC35E3	NM_018656.2
1.04	STARD7	NM_020151.3
1.09	EXOC4	NM_021807.3
1.14	RAD51	NM_002875.3,NM_133487.2
2.03	GNG4	NM_001098722.1,NM_004485.3,NM_001098721.1
1.84	ANKRD1	NM_014391.2
1.13	PKD2	NM_000297.2
2.02	PDXK	NM_003681.4
1.23	IFITM1	NM_003641.3
1.18	CDC2L2,RP11-345P4.4	NM_001110781.1
1.43	AC097382.3-1	NM_152293.2
1.56	TMEM126B	NM_018480.2
1.57	KIAA0226	NA
1.15	DLG5	NM_004747.3
1.17	DYM	NM_017653.3
1.23	FADS1	NM_013402.4
1.12	SMCR7L	NM_019008.4
1.96	DUSP8	NM_004420.2
1.08	FOXM1	NM_202002.1,NM_021953.2,NM_202003.1
1.09	MKNK2	NM_017572.3,NM_199054.2
1.16	NR3C1	NM_001024094.1,NM_001018077.1,NM_001018076.1,NM_001018075.1,NM_001018074.1,NM_000176.2
2.35	ASB1	NM_001040445.1
1.03	HEATR2	NM_017802.3
1.02	TUBB2A	NM_001069.2
1.00	ZP3	NA
1.81	ERMP1	NM_024896.2
1.05	GFOD2	NM_030819.3
1.25	IMP3	NM_018285.2
1.65	RNF13	NM_007282.4,NM_183381.2
1.13	MACROD1	NM_014067.3
2.45	TMC6	NA
1.45	PPP1R7	NM_002712.1

1.33	FAM129A	NM_052966.2
1.00	EHD4	NM_139265.3
1.34	GFOD1	NM_018988.2
1.46	SLC3A2	NM_002394.4,NM_001013251.1,NM_001012664.1,NM_001012663.1,NM_001012662.1,NM_001012661.1
1.27	AFF1	NM_005935.2
1.13	SNX6	NM_021249.3,NM_152233.2
1.07	EI24	NM_001007277.1,NM_004879.3
3.22	FAM101B	NM_182705.2
1.29	IFI16	NM_005531.2
1.08	AC024937.5	NA
1.27	EIF3S10	NM_003750.2
1.14	ADA	NM_000022.2
1.20	FOLR3	NM_000804.2
1.17	DHRS13	NM_144683.3
1.46	TMEM55A	NM_018710.2
1.46	DDX3Y	NA
2.45	FABP3	NM_004102.3
1.15	CCNL2	NA
1.65	TMEM66	NM_016127.4
2.07	RP11-34D15.5	NA
1.24	SENP6	NM_001100409.1,NM_015571.2
2.13	SPP1	NM_001040060.1,NM_001040058.1,NM_000582.2
1.07	SOX9	NM_000346.3
1.14	GPR172A	NM_024531.3
1.83	ATP6AP1	NM_001183.4
1.07	ZFYVE26	NM_015346.3
1.68	GRPEL2	NM_152407.3
1.29	FUCA2	NM_032020.3
1.33	TACC2	NM_206861.1,NM_206860.1,NM_006997.2,NM_206862.2
1.74	ADORA2B	NM_000676.2
1.02	UCHL3	NM_006002.3
1.05	LEMD3	NM_014319.3
1.17	PIP4K2C	NA
1.22	MSN	NM_002444.2
1.07	NUDT9	NM_024047.3,NM_198038.1
1.30	FEM1A	NM_018708.2
1.02	AC097458.2	NM_023934.3
1.59	SLC6A8	NA
2.39	TCEAL2	NM_080390.3
1.84	RTN3	NM_006054.2,NM_201428.1,NM_201430.1,NM_201429.1
1.53	LGALS3	NM_002306.2
1.87	S100A2	NM_005978.3
1.20	PNKD	NM_015488.4,NM_022572.3
1.22	KIAA0430	NM_014647.2
1.35	EIF5	NM_183004.3,NM_001969.3
1.84	LBR	NM_194442.1,NM_002296.2
1.65	R3HDM2	NM_014925.3
2.34	CLN3	NM_000086.2,NM_001042432.1
1.14	REXO4	NM_020385.2
1.05	TDG	NM_003211.4
1.05	MRFAP1L1	NM_203462.2
1.23	CLCN6	NM_001286.2
1.78	HPCAL1	NM_134421.1,NM_002149.2
2.30	PPT1	NA
1.24	KLF13	NM_015995.2
1.04	DNAJB12	NM_017626.4,NM_001002762.2
1.28	PDCD6IP	NA
1.37	FAF1	NM_007051.2
1.92	LCLAT1	NM_182551.3,NM_001002257.1
1.34	ADCY9	NM_001116.2
1.07	CXYorf3	NM_005088.2
2.16	PGM2L1	NM_173582.3
1.11	PATZ1	NM_032052.1,NM_032050.1,NM_014323.2
1.22	KIAA1012	NM_014939.3
1.40	WDFY1	NM_020830.3
1.23	RAP2A	NM_021033.6
1.16	ACOT4	NM_152331.3
1.45	CHKA	NM_212469.1,NM_001277.2
1.81	PLAU	NA
1.36	RGS10	NM_001005339.1,NM_002925.3
1.45	ZFP36L1	NM_004926.2
1.44	NCK2	NM_001004722.1,NM_001004720.1,NM_003581.2
1.15	ZNF12	NM_006956.2,NM_016265.3
1.50	IPO5	NM_002271.4
1.41	CHD9	NM_025134.4
1.42	NGRN	NM_001033088.1,NM_016645.2
1.26	AC010896.3	NM_033505.2
1.08	GRB10	NM_001001555.2,NM_001001550.2,NM_001001549.2,NM_005311.4
1.14	DKK3	NM_001018057.1,NM_013253.4,NM_015881.5
1.23	ADNP2	NM_014913.3
1.16	DFFB	NM_004402.2
1.56	TRPM7	NM_017672.3
1.86	DERL3	NM_001002862.2,NM_198440.3
1.27	C9orf30	NM_080655.1
2.31	SH2D5	NM_001103160.1,NM_001103161.1
1.03	OGDH	NM_002541.2
1.06	TTLL4	NM_014640.4
1.00	RABEP1	NM_004703.4,NM_001083585.1
1.08	NKIRAS1	NM_020345.3
1.90	DCBLD2	NM_080927.3
1.34	GLCE	NM_015554.1
1.05	FDFT1	NM_004462.3
1.42	NDUFV3	NM_001001503.1,NM_021075.3
1.70	B3GAT3	NM_012200.2
1.04	TNPO2	NA
1.13	INHBE	NM_031479.3
1.46	UBQLN4	NM_020131.3
1.26	ERLIN1	NM_001100626.1,NM_006459.3
1.44	GAPVD1	NM_015635.2
1.62	SYVN1	NM_172230.2,NM_032431.2
1.91	P2RX4	NM_002560.2
1.01	FAM102A	NM_001035254.1,NM_203305.2
1.19	TTL	NM_153712.4
1.09	RPS4Y2	NM_001039567.2
1.24	PUS7	NM_019042.3
2.83	DDIT3	NM_004083.4
1.42	MGLL	NM_001003794.1,NM_007283.5
1.62	HEXA	NM_000520.4
1.20	C14orf135	NM_022495.5
1.10	RAC1	NM_018890.3,NM_006908.4
1.19	CYP51A1	NA
1.07	COX7A2L	NM_004718.2
1.03	SURF1	NM_003172.2
1.15	C6orf62	NM_030939.4
2.61	ARMET	NM_006010.3
1.27	MAP3K9	NM_033141.2
1.35	GNL3L	NM_019067.4
1.18	TBL1XR1	NM_024665.4
1.01	AEBP1	NM_001129.3
1.63	TMEM198	NM_001005209.1
1.23	FAM35A	NM_019054.2
2.14	PDXK	NM_003681.4
1.00	MYO1D	NM_015194.1
1.01	LA16c-4G1.3	NA
1.10	TMEM126B	NM_018480.2
1.86	LRRC8E	NM_025061.3
1.09	ZP3	NA
1.73	METRNL	NM_001004431.1
1.20	NARG1	NM_057175.3

1.46	PLA2G16	NA
1.99	APH1B	NA
1.31	PIM3	NM_001001852.3
1.28	ARG2	NM_001172.3
1.01	AC011475.6-2	NA
1.15	ACAT1	NM_000019.3
2.19	HSD17B14	NM_016246.2
1.10	PSME1	NM_176783.1,NM_006263.2
1.23	CAPG	NM_001747.2
1.33	ZNF226	NA
1.24	ADCK2	NM_052853.3
1.36	WIPI2	NM_001033520.1,NM_016003.3,NM_015610.3,NM_001033519.1,NM_001033518.1
1.23	SPG21	NA
1.13	AURKB	NM_004217.2
1.23	RC3H2	NM_018835.2,NM_001100588.1
1.58	AL009183.10	NA
1.27	AC099759.1	NA
1.12	FAM101B	NM_182705.2
2.72	SCD	NM_005063.4
1.34	SLC38A1	NM_001077484.1,NM_030674.3
1.20	TNPO1	NM_002270.3,NM_153188.2
2.39	DNAJC3	NM_006260.3
1.25	FBXW2	NM_012164.3
1.11	C14orf21	NA
2.64	CLCN5	NA
1.09	C8orf83	NA
1.32	TNIK	NA
1.21	HOXA7	NM_006896.3
1.08	RHOT1	NM_001033568.1,NM_001033566.1,NM_018307.3
1.44	TRIM25	NM_005082.4
1.23	GSPT1	NA
1.11	RPL7L1	NA
1.59	AMDHD2	NM_001145815.1
1.10	KIAA0114	NA
1.21	FUNDC2	NM_023934.3
1.16	RGMB	NM_001012761.2
1.09	SNHG5	NA
1.13	TBC1D15	NA
1.22	SFMBT1	NM_001005159.1,NM_016329.2,NM_001005158.1
1.63	SPNS1	NA
1.94	GDAP1	NM_001040875.1,NM_018972.2
1.02	ALG1	NM_019109.4
1.08	ADA	NM_000022.2
2.35	EXOSC6	NA
1.19	HSD17B7	NA
1.31	SPG20	NA
1.14	XIAP	NM_001167.2

Supporting Information Table S5. Gene ontology from gene microarray data of EZH2-regulated genes in undifferentiated hMSCs

(log2(Ratio) >1 and p-value < 0.05)

GO Terms (218)	number (1423 genes)
GO:0045768~positive regulation of anti-apoptosis	4
GO:0045767~regulation of anti-apoptosis	4
GO:0008629~induction of apoptosis by intracellular signa	3
GO:0008285~negative regulation of cell proliferation	22
GO:0042127~regulation of cell proliferation	41
GO:0051726~regulation of cell cycle	18
GO:0016049~cell growth	5
GO:0040007~growth	10
GO:0007050~cell cycle arrest	6
GO:0000075~cell cycle checkpoint	5
GO:0050678~regulation of epithelial cell proliferation	4
GO:0008284~positive regulation of cell proliferation	17
GO:0045787~positive regulation of cell cycle	3
GO:0010564~regulation of cell cycle process	5
GO:0007346~regulation of mitotic cell cycle	6
GO:0008283~cell proliferation	15
GO:0045786~negative regulation of cell cycle	3
GO:0051329~interphase of mitotic cell cycle	3
GO:0051325~interphase	3
GO:0055085~transmembrane transport	33
GO:0051181~cofactor transport	4
GO:0051050~positive regulation of transport	15
GO:0051051~negative regulation of transport	10
GO:0000041~transition metal ion transport	5
GO:0006826~iron ion transport	3
GO:0032368~regulation of lipid transport	3
GO:0016197~endosome transport	4
GO:0006892~post-Golgi vesicle-mediated transport	4
GO:0048193~Golgi vesicle transport	7
GO:0006839~mitochondrial transport	4
GO:0008643~carbohydrate transport	3
GO:0006813~potassium ion transport	3
GO:0051048~negative regulation of secretion	3
GO:0030155~regulation of cell adhesion	10
GO:0043062~extracellular structure organization	11
GO:0010648~negative regulation of cell communication	14
GO:0030198~extracellular matrix organization	7
GO:0007159~leukocyte adhesion	3
GO:0006944~membrane fusion	4
GO:0070507~regulation of microtubule cytoskeleton orga	3
GO:0031589~cell-substrate adhesion	5
GO:0007160~cell-matrix adhesion	4
GO:0045785~positive regulation of cell adhesion	3
GO:0007015~actin filament organization	3
GO:0000226~microtubule cytoskeleton organization	5
GO:0001816~cytokine production	3

	Groups	GO Terms	number	Total 1423 genes	%
1	Cell Cycle and Proliferation	GO:0045768~positive regulation of anti-apoptosis	4	177	12.4
		GO:0045767~regulation of anti-apoptosis	4		
		GO:0008629~induction of apoptosis by intracellular sig	3		
		GO:0008285~negative regulation of cell proliferation	22		
		GO:0042127~regulation of cell proliferation	41		
		GO:0051726~regulation of cell cycle	18		
		GO:0016049~cell growth	5		
		GO:0040007~growth	10		
		GO:0007050~cell cycle arrest	6		
		GO:0000075~cell cycle checkpoint	5		
		GO:0050678~regulation of epithelial cell proliferation	4		
		GO:0008284~positive regulation of cell proliferation	17		
		GO:0045787~positive regulation of cell cycle	3		
		GO:0010564~regulation of cell cycle process	5		
		GO:0007346~regulation of mitotic cell cycle	6		
		GO:0008283~cell proliferation	15		
		GO:0045786~negative regulation of cell cycle	3		
		GO:0051329~interphase of mitotic cell cycle	3		
		GO:0051325~interphase	3		
2	Cellular Transport	GO:0055085~transmembrane transport	33	101	7.1
		GO:0051181~cofactor transport	4		
		GO:0051050~positive regulation of transport	15		
		GO:0051051~negative regulation of transport	10		
		GO:0000041~transition metal ion transport	5		
		GO:0006826~iron ion transport	3		
		GO:0032368~regulation of lipid transport	3		
		GO:0016197~endosome transport	4		
		GO:0006892~post-Golgi vesicle-mediated transport	4		
		GO:0048193~Golgi vesicle transport	7		
		GO:0006839~mitochondrial transport	4		
		GO:0008643~carbohydrate transport	3		
		GO:0006813~potassium ion transport	3		
		GO:0051048~negative regulation of secretion	3		
3	Cytoskeleton and Cell adhe	GO:0030155~regulation of cell adhesion	10	75	5.27
		GO:0043062~extracellular structure organization	11		
		GO:0010648~negative regulation of cell communicatior	14		
		GO:0030198~extracellular matrix organization	7		
		GO:0007159~leukocyte adhesion	3		
		GO:0006944~membrane fusion	4		
		GO:0070507~regulation of microtubule cytoskeleton or	3		
		GO:0031589~cell-substrate adhesion	5		
		GO:0007160~cell-matrix adhesion	4		
		GO:0045785~positive regulation of cell adhesion	3		
		GO:0007015~actin filament organization	3		

GO:0045444~fat cell differentiation	7
GO:0070584~mitochondrion morphogenesis	3
GO:0001890~placenta development	6
GO:0045446~endothelial cell differentiation	3
GO:0001501~skeletal system development	18
GO:0002062~chondrocyte differentiation	3
GO:0045596~negative regulation of cell differentiation	12
GO:0050873~brown fat cell differentiation	3
GO:0051216~cartilage development	5
GO:0001889~liver development	4
GO:0048589~developmental growth	4
GO:0043473~pigmentation	3
GO:0051094~positive regulation of developmental proces	10
GO:0009791~post-embryonic development	3
GO:0001655~urogenital system development	4
GO:0060348~bone development	4
GO:0001822~kidney development	3
GO:0048732~gland development	3
GO:0008544~epidermis development	3
GO:0007398~ectoderm development	3
GO:0045428~regulation of nitric oxide biosynthetic proce	5
GO:0045429~positive regulation of nitric oxide biosynthet	4
GO:0006809~nitric oxide biosynthetic process	3
GO:0046209~nitric oxide metabolic process	3
GO:0046112~nucleobase biosynthetic process	3
GO:0006004~fucose metabolic process	3
GO:0032269~negative regulation of cellular protein meta	11
GO:0008299~isoprenoid biosynthetic process	3
GO:0007588~excretion	5
GO:0051248~negative regulation of protein metabolic pro	11
GO:0009112~nucleobase metabolic process	3
GO:0009066~aspartate family amino acid metabolic proc	3
GO:0007017~microtubule-based process	13
GO:0000272~polysaccharide catabolic process	3
GO:0032886~regulation of microtubule-based process	4
GO:0009124~nucleoside monophosphate biosynthetic pr	4
GO:0009064~glutamine family amino acid metabolic proc	4
GO:0043112~receptor metabolic process	3
GO:0003012~muscle system process	9
GO:0043648~dicarboxylic acid metabolic process	3
GO:0006220~pyrimidine nucleotide metabolic process	3
GO:0009123~nucleoside monophosphate metabolic proc	4
GO:0006029~proteoglycan metabolic process	3
GO:0006720~isoprenoid metabolic process	3
GO:0042035~regulation of cytokine biosynthetic process	4
GO:0044272~sulfur compound biosynthetic process	3
GO:0006638~neutral lipid metabolic process	3
GO:0006662~glycerol ether metabolic process	3
GO:0006733~oxidoreduction coenzyme metabolic proces	3
GO:0006518~peptide metabolic process	3
GO:0044264~cellular polysaccharide metabolic process	3

		GO:0000226~microtubule cytoskeleton organization	5		
		GO:0001816~cytokine production	3		
4	Development and Function	GO:0045444~fat cell differentiation	7	104	7.31
		GO:0070584~mitochondrion morphogenesis	3		
		GO:0001890~placenta development	6		
		GO:0045446~endothelial cell differentiation	3		
		GO:0001501~skeletal system development	18		
		GO:0002062~chondrocyte differentiation	3		
		GO:0045596~negative regulation of cell differentiation	12		
		GO:0050873~brown fat cell differentiation	3		
		GO:0051216~cartilage development	5		
		GO:0001889~liver development	4		
		GO:0048589~developmental growth	4		
		GO:0043473~pigmentation	3		
		GO:0051094~positive regulation of developmental proc	10		
		GO:0009791~post-embryonic development	3		
		GO:0001655~urogenital system development	4		
		GO:0060348~bone development	4		
		GO:0001822~kidney development	3		
		GO:0048732~gland development	3		
		GO:0008544~epidermis development	3		
		GO:0007398~ectoderm development	3		
5	Metabolism	GO:0045428~regulation of nitric oxide biosynthetic proc	5	188	13.2
		GO:0045429~positive regulation of nitric oxide biosynth	4		
		GO:0006809~nitric oxide biosynthetic process	3		
		GO:0046209~nitric oxide metabolic process	3		
		GO:0046112~nucleobase biosynthetic process	3		
		GO:0006004~fucose metabolic process	3		
		GO:0032269~negative regulation of cellular protein me	11		
		GO:0008299~isoprenoid biosynthetic process	3		
		GO:0007588~excretion	5		
		GO:0051248~negative regulation of protein metabolic p	11		
		GO:0009112~nucleobase metabolic process	3		
		GO:0009066~aspartate family amino acid metabolic pro	3		
		GO:0007017~microtubule-based process	13		
		GO:0000272~polysaccharide catabolic process	3		
		GO:0032886~regulation of microtubule-based process	4		
		GO:0009124~nucleoside monophosphate biosynthetic	4		
		GO:0009064~glutamine family amino acid metabolic pr	4		
		GO:0043112~receptor metabolic process	3		
		GO:0003012~muscle system process	9		
		GO:0043648~dicarboxylic acid metabolic process	3		
		GO:0006220~pyrimidine nucleotide metabolic process	3		
		GO:0009123~nucleoside monophosphate metabolic pro	4		
		GO:0006029~proteoglycan metabolic process	3		
		GO:0006720~isoprenoid metabolic process	3		
		GO:0042035~regulation of cytokine biosynthetic proces	4		
		GO:0044272~sulfur compound biosynthetic process	3		
		GO:0006638~neutral lipid metabolic process	3		

GO:0018904~organic ether metabolic process	3
GO:0019882~antigen processing and presentation	4
GO:0051054~positive regulation of DNA metabolic proces	3
GO:0043603~cellular amide metabolic process	3
GO:0006790~sulfur metabolic process	5
GO:0042445~hormone metabolic process	4
GO:0009309~amine biosynthetic process	3
GO:0016485~protein processing	3
GO:0051052~regulation of DNA metabolic process	3
GO:0006091~generation of precursor metabolites and en	22
GO:0051130~positive regulation of cellular component or	16
GO:0007005~mitochondrion organization	13
GO:0048259~regulation of receptor-mediated endocytosi	4
GO:0008535~respiratory chain complex IV assembly	3
GO:0002685~regulation of leukocyte migration	4
GO:0031529~ruffle organization	3
GO:0007030~Golgi organization	4
GO:0055114~oxidation reduction	35
GO:0045932~negative regulation of muscle contraction	3
GO:0007032~endosome organization	3
GO:0022406~membrane docking	4
GO:0034381~lipoprotein particle clearance	3
GO:0032768~regulation of monooxygenase activity	3
GO:0033273~response to vitamin	5
GO:0001666~response to hypoxia	8
GO:0034101~erythrocyte homeostasis	4
GO:0051258~protein polymerization	4
GO:0051260~protein homooligomerization	6
GO:0070482~response to oxygen levels	8
GO:0051291~protein heterooligomerization	4
GO:0007006~mitochondrial membrane organization	3
GO:0048872~homeostasis of number of cells	6
GO:0034097~response to cytokine stimulus	5
GO:0009628~response to abiotic stimulus	17
GO:0017157~regulation of exocytosis	3
GO:0019229~regulation of vasoconstriction	3
GO:0007585~respiratory gaseous exchange	3
GO:0001817~regulation of cytokine production	9
GO:0045454~cell redox homeostasis	4
GO:0045765~regulation of angiogenesis	4
GO:0051341~regulation of oxidoreductase activity	3
GO:0033189~response to vitamin A	3
GO:0033500~carbohydrate homeostasis	3
GO:0042593~glucose homeostasis	3
GO:0042493~response to drug	9
GO:0042542~response to hydrogen peroxide	3
GO:0050900~leukocyte migration	3
GO:0050866~negative regulation of cell activation	3
GO:0006936~muscle contraction	6
GO:0030258~lipid modification	3
GO:0008016~regulation of heart contraction	3

		GO:0006662~glycerol ether metabolic process	3		
		GO:0006733~oxidoreduction coenzyme metabolic proc	3		
		GO:0006518~peptide metabolic process	3		
		GO:0044264~cellular polysaccharide metabolic proces	3		
		GO:0018904~organic ether metabolic process	3		
		GO:0019882~antigen processing and presentation	4		
		GO:0051054~positive regulation of DNA metabolic proc	3		
		GO:0043603~cellular amide metabolic process	3		
		GO:0006790~sulfur metabolic process	5		
		GO:0042445~hormone metabolic process	4		
		GO:0009309~amine biosynthetic process	3		
		GO:0016485~protein processing	3		
		GO:0051052~regulation of DNA metabolic process	3		
		GO:0006091~generation of precursor metabolites and	22		

6	Molaculer Biochemistry	GO:0051130~positive regulation of cellular component	16	254	17.8
		GO:0007005~mitochondrion organization	13		
		GO:0048259~regulation of receptor-mediated endocyto	4		
		GO:0008535~respiratory chain complex IV assembly	3		
		GO:0002685~regulation of leukocyte migration	4		
		GO:0031529~ruffle organization	3		
		GO:0007030~Golgi organization	4		
		GO:0055114~oxidation reduction	35		
		GO:0045932~negative regulation of muscle contraction	3		
		GO:0007032~endosome organization	3		
		GO:0022406~membrane docking	4		
		GO:0034381~lipoprotein particle clearance	3		
		GO:0032768~regulation of monooxygenase activity	3		
		GO:0033273~response to vitamin	5		
		GO:0001666~response to hypoxia	8		
		GO:0034101~erythrocyte homeostasis	4		
		GO:0051258~protein polymerization	4		
		GO:0051260~protein homooligomerization	6		
		GO:0070482~response to oxygen levels	8		
		GO:0051291~protein heterooligomerization	4		
		GO:0007006~mitochondrial membrane organization	3		
		GO:0048872~homeostasis of number of cells	6		
		GO:0034097~response to cytokine stimulus	5		
		GO:0009628~response to abiotic stimulus	17		
		GO:0017157~regulation of exocytosis	3		
		GO:0019229~regulation of vasoconstriction	3		
		GO:0007585~respiratory gaseous exchange	3		
		GO:0001817~regulation of cytokine production	9		
		GO:0045454~cell redox homeostasis	4		
		GO:0045765~regulation of angiogenesis	4		
		GO:0051341~regulation of oxidoreductase activity	3		
		GO:0033189~response to vitamin A	3		
		GO:0033500~carbohydrate homeostasis	3		
		GO:0042593~glucose homeostasis	3		
		GO:0042493~response to drug	9		
		GO:0042542~response to hydrogen peroxide	3		

GO:0010817~regulation of hormone levels	5
GO:0060191~regulation of lipase activity	3
GO:0010038~response to metal ion	4
GO:0009314~response to radiation	6
GO:0008344~adult locomotory behavior	3
GO:0009266~response to temperature stimulus	4
GO:0009612~response to mechanical stimulus	3
GO:0007612~learning	3
GO:0051606~detection of stimulus	5
GO:0007605~sensory perception of sound	4
GO:0050954~sensory perception of mechanical stimulus	4
GO:0030534~adult behavior	3
GO:0009416~response to light stimulus	4
GO:0007611~learning or memory	3
GO:0009582~detection of abiotic stimulus	3
GO:0033554~cellular response to stress	39
GO:0007568~aging	9
GO:0006968~cellular defense response	4
GO:0051235~maintenance of location	4
GO:0007623~circadian rhythm	3
GO:0031346~positive regulation of cell projection organiz	3
GO:0051651~maintenance of location in cell	3
GO:0016032~viral reproduction	3
GO:0045087~innate immune response	4
GO:0007565~female pregnancy	3
GO:0006955~immune response	18
GO:0009615~response to virus	3
GO:0007249~I-kappaB kinase/NF-kappaB cascade	3
GO:0007264~small GTPase mediated signal transductior	24
GO:0007243~protein kinase cascade	26
GO:0007242~intracellular signaling cascade	67
GO:0000165~MAPKKK cascade	15
GO:0007265~Ras protein signal transduction	8
GO:0009968~negative regulation of signal transduction	13
GO:0017015~regulation of transforming growth factor bet	4
GO:0042058~regulation of epidermal growth factor recep	3
GO:0007205~activation of protein kinase C activity by G-	3
GO:0007259~JAK-STAT cascade	3
GO:0030522~intracellular receptor-mediated signaling pa	4
GO:0008277~regulation of G-protein coupled receptor pr	3
GO:0007167~enzyme linked receptor protein signaling pa	14
GO:0030518~steroid hormone receptor signaling pathwa	3
GO:0007178~transmembrane receptor protein serine/thre	4
GO:0007169~transmembrane receptor protein tyrosine ki	8
GO:0016055~Wnt receptor signaling pathway	4
GO:0007166~cell surface receptor linked signal transduc	46
GO:0007186~G-protein coupled receptor protein signalin	20
GO:0007266~Rho protein signal transduction	3
GO:0042770~DNA damage response, signal transductior	3
GO:0016311~dephosphorylation	14
GO:0006470~protein amino acid dephosphorylation	11

		GO:0050900~leukocyte migration	3		
		GO:0050866~negative regulation of cell activation	3		
		GO:0006936~muscle contraction	6		
		GO:0030258~lipid modification	3		
		GO:0008016~regulation of heart contraction	3		
		GO:0010817~regulation of hormone levels	5		
		GO:0060191~regulation of lipase activity	3		
		GO:0010038~response to metal ion	4		
		GO:0009314~response to radiation	6		
7	Nervous System	GO:0008344~adult locomotory behavior	3	39	2.74
		GO:0009266~response to temperature stimulus	4		
		GO:0009612~response to mechanical stimulus	3		
		GO:0007612~learning	3		
		GO:0051606~detection of stimulus	5		
		GO:0007605~sensory perception of sound	4		
		GO:0050954~sensory perception of mechanical stimulu	4		
		GO:0030534~adult behavior	3		
		GO:0009416~response to light stimulus	4		
		GO:0007611~learning or memory	3		
		GO:0009582~detection of abiotic stimulus	3		
8	Other GO Terms	GO:0033554~cellular response to stress	39	96	6.75
		GO:0007568~aging	9		
		GO:0006968~cellular defense response	4		
		GO:0051235~maintenance of location	4		
		GO:0007623~circadian rhythm	3		
		GO:0031346~positive regulation of cell projection orgai	3		
		GO:0051651~maintenance of location in cell	3		
		GO:0016032~viral reproduction	3		
		GO:0045087~innate immune response	4		
		GO:0007565~female pregnancy	3		
		GO:0006955~immune response	18		
		GO:0009615~response to virus	3		
9	Cell Signaling	GO:0007249~I-kappaB kinase/NF-kappaB cascade	3	281	19.7
		GO:0007264~small GTPase mediated signal transducti	24		
		GO:0007243~protein kinase cascade	26		
		GO:0007242~intracellular signaling cascade	67		
		GO:0000165~MAPKKK cascade	15		
		GO:0007265~Ras protein signal transduction	8		
		GO:0009968~negative regulation of signal transductior	13		
		GO:0017015~regulation of transforming growth factor b	4		
		GO:0042058~regulation of epidermal growth factor rec	3		
		GO:0007205~activation of protein kinase C activity by (	3		
		GO:0007259~JAK-STAT cascade	3		
		GO:0030522~intracellular receptor-mediated signaling	4		
		GO:0008277~regulation of G-protein coupled receptor	3		
		GO:0007167~enzyme linked receptor protein signaling	14		
		GO:0030518~steroid hormone receptor signaling pathv	3		
		GO:0007178~transmembrane receptor protein serine/tl	4		

GO:0051259~protein oligomerization	11
GO:0032392~DNA geometric change	3
GO:0032508~DNA duplex unwinding	3
GO:0006493~protein amino acid O-linked glycosylation	3
GO:0018108~peptidyl-tyrosine phosphorylation	4
GO:0018212~peptidyl-tyrosine modification	4
GO:0017148~negative regulation of translation	3
GO:0006457~protein folding	9
GO:0009451~RNA modification	3
GO:0006367~transcription initiation from RNA polymeras	3
GO:0006352~transcription initiation	3
GO:0006323~DNA packaging	4
GO:0051604~protein maturation	4
GO:0006333~chromatin assembly or disassembly	3
GO:0006412~translation	7
GO:0050821~protein stabilization	3
GO:0046777~protein amino acid autophosphorylation	5
GO:0031647~regulation of protein stability	3
GO:0006512~ubiquitin cycle	5

		GO:0007169~transmembrane receptor protein tyrosine	8		
		GO:0016055~Wnt receptor signaling pathway	4		
		GO:0007166~cell surface receptor linked signal transdu	46		
		GO:0007186~G-protein coupled receptor protein signa	20		
		GO:0007266~Rho protein signal transduction	3		
		GO:0042770~DNA damage response, signal transducti	3		

10	Transcription & Translation	GO:0016311~dephosphorylation	14	108	7.59
		GO:0006470~protein amino acid dephosphorylation	11		
		GO:0051259~protein oligomerization	11		
		GO:0032392~DNA geometric change	3		
		GO:0032508~DNA duplex unwinding	3		
		GO:0006493~protein amino acid O-linked glycosylation	3		
		GO:0018108~peptidyl-tyrosine phosphorylation	4		
		GO:0018212~peptidyl-tyrosine modification	4		
		GO:0017148~negative regulation of translation	3		
		GO:0006457~protein folding	9		
		GO:0009451~RNA modification	3		
		GO:0006367~transcription initiation from RNA polymera	3		
		GO:0006352~transcription initiation	3		
		GO:0006323~DNA packaging	4		
		GO:0051604~protein maturation	4		
		GO:0006333~chromatin assembly or disassembly	3		
		GO:0006412~translation	7		
		GO:0050821~protein stabilization	3		
		GO:0046777~protein amino acid autophosphorylation	5		
		GO:0031647~regulation of protein stability	3		
		GO:0006512~ubiquitin cycle	5		

SupplementaryTable S6. Genes list from gene ontology data of EZH2 regulated genes in undifferentiated hMSCs by gene microarray

(log2(Ratio) >1 and p-value < 0.05 for gene ontology analysis)

gene_symbol	RefSeq_ID	ID	Gene_ID	chr_name	log2 (Ratio) shEZH2/Contro	P-value shEZH2/Contro	gene_description
ABCA1	NM_005502	PH_hs_0022625	19	chr9	1.307441653	0.000476178	ATP-binding cassette sub-family A member 1
ABCA3	NM_001089	PH_hs_0002351	21	chr16	1.407495801	0.000362013	ATP-binding cassette sub-family A member 3
ABCC10	NM_033450	PH_hs_0003648	89845	chr6	1.096105832	0.00847673	Multidrug resistance-associated protein 7
ACOT4	NM_152331	PH_hs_0031224	122970	chr14	1.162944494	0.002209179	Acyl-coenzyme A thioesterase 4
ACP5	NA	PH_hs_0000161	NA	chr19	1.646098725	5.08166E-05	Tartrate-resistant acid phosphatase type 5 Precursor
ADA	NM_000022	PH_hs_0040289	100	chr20	1.078990409	4.04187E-06	Adenosine deaminase
ADARB1	NA	PH_hs_0022649	NA	chr21	1.387901488	0.002754179	Double-stranded RNA-specific editase 1
ADCY9	NM_001116	PH_hs_0031049	115	chr16	1.340121346	0.001251695	Adenylate cyclase type 9
ADM	NM_001124	PH_hs_0025829	133	chr11	1.308690355	6.27512E-05	ADM Precursor [Contains Adrenomedullin(AM)
ADORA2B	NM_000676	PH_hs_0028884	136	chr17	1.737288209	3.0086E-10	Adenosine receptor A2b
AEBP1	NM_001129	PH_hs_0033445	165	chr7	1.009671768	8.52551E-06	Adipocyte enhancer-binding protein 1 Precursor
AFG3L2	NM_006796	PH_hs_0025528	10939	chr18	1.139084221	1.35592E-07	AFG3-like protein 2
AIFM2	NM_032797	PH_hs_0006413	84883	chr10	1.381749855	3.07035E-07	Apoptosis-inducing factor 2
AKAP13	NM_144767	PH_hs_0023930	11214	chr15	1.036368327	0.00084657	A-kinase anchor protein 13 (AKAP 13)
AKAP3	NM_006422	PH_hs_0005424	10566	chr12	1.49062946	4.43094E-07	A-kinase anchor protein 3 (Protein kinase A-anchoring protein 3)
ALDH9A1	NM_000696	PH_hs_0000337	223	chr1	1.102784154	5.86955E-05	4-trimethylaminobutyraldehyde dehydrogenase
ALG1	NM_0191094	PH_hs_0040277	56052	chr16	1.021543691	0.000167027	Chitobiosyldiphosphodolichol beta-mannosyltransferase
ALOX5AP	NM_001629	PH_hs_0005601	241	chr13	1.301565142	0.000267177	Arachidonate 5-lipoxygenase-activating protein
FANCI	NA	PH_hs_0016481	NA	chr15	1.133166325	2.41057E-05	Fanconi anemia group I protein (Protein FANCI)
ANTXR1	NM_032208	PH_hs_0023373	84168	chr2	1.107053579	0.001041521	Anthrax toxin receptor 1 Precursor (Tumor endothelial marker 8)
APH1B	NA	PH_hs_0034630	NA	chr15	1.992912207	3.04663E-05	Gamma-secretase subunit APH-1B (APH-1b)(Aph-1beta)
APOC1	NM_001645	PH_hs_0013741	341	chr19	1.81276294	1.22292E-05	Apolipoprotein C-I Precursor (Apo-CI)(ApoC-I)
APOE	NM_000041	PH_hs_0022287	348	chr19	3.650358555	3.60406E-25	Apolipoprotein E Precursor (Apo-E)
AQP11	NM_173039	PH_hs_0023735	282679	chr11	2.117621802	5.18168E-09	Aquaporin-11 (AQP-11)
ARG2	NM_001172	PH_hs_0034837	384	chr14	1.279281857	0.000246505	Arginase-2, mitochondrial Precursor (Arginase II)
ARL1	NM_001177	PH_hs_0018024	400	chr12	1.828919536	4.63451E-05	ADP-ribosylation factor-like protein 1
ASB1	NM_00104044	PH_hs_0026771	51665	chr2	2.35106538	0.001493013	Ankyrin repeat and SOCS box protein 1 (ASB-1)
ASPH	NM_032466	PH_hs_0024953	444	chr8	1.077591226	0.000243432	Aspartyl/asparaginyl beta-hydroxylase
ASRGL1	NM_00108392	PH_hs_0025272	80150	chr11	2.030642145	0.00012287	L-asparaginase
ATG10	NA	PH_hs_0019535	NA	chr5	1.012865251	0.001166101	Autophagy-related protein 10 (APG10-like)

ATP5L	NM_006476	PH_hs_0005969	10632	chr11	1.035943512	0.00043409	ATP synthase subunit g, mitochondrial (ATPase subunit g)
ATP6AP1	NM_001183	PH_hs_0028734	537	chrX	1.82879222	9.23468E-09	V-type proton ATPase subunit S1 Precursor (V-ATPase subunit S1)
ATP6V0B	NM_0040473	PH_hs_0000774	533	chr1	1.123594	1.18017E-06	V-type proton ATPase 21 kDa proteolipid subunit (V-ATPase 21 kDa proteolipid subunit
ATP6V0E2	NM_145230	PH_hs_0016999	155066	chr7	1.036333281	0.000150149	V-type proton ATPase subunit e 2 (V-ATPase subunit e 2)(Vacuolar proton pump subunit e 2)
ATP6V1A	NM_001690	PH_hs_0024782	523	chr3	1.33459104	0.000160431	V-type proton ATPase catalytic subunit A (V-ATPase subunit A)
ATP6V1C1	NM_001695	PH_hs_0004880	528	chr8	1.334114945	6.99639E-07	V-type proton ATPase subunit C 1 (V-ATPase subunit C 1)
ATP6V1H	NM_015941	PH_hs_0001719	51606	chr8	1.821977573	8.83232E-11	V-type proton ATPase subunit H (V-ATPase subunit H
BIRC6	NM_016252	PH_hs_0024017	57448	chr2	1.23183832	2.32449E-06	Baculoviral IAP repeat-containing protein 6 (Ubiquitin-conjugating BIR domain enzyme apollon)
CAD	NM_004341	PH_hs_0024275	790	chr2	1.331125405	0.002830208	CAD protein [Includes Glutamine-dependent carbamoyl-phosphate synthase
CADM1	NM_00109851	PH_hs_0022714	23705	chr11	1.151115932	1.0607E-06	Cell adhesion molecule 1 Precursor (Immunoglobulin superfamily member 4)
CALCOCO2	NM_005831	PH_hs_0009285	10241	chr17	1.12035914	0.002313124	Calcium-binding and coiled-coil domain-containing protein 2
CALR	NM_004343	PH_hs_0017137	811	chr19	2.187279821	3.27275E-05	Calreticulin Precursor (CRP55)(Calregulin)(HACBP)(ERp60)(grp60)
CASP1	NM_033293	PH_hs_0000334	834	chr11	1.282321491	4.34032E-07	Caspase-1 Precursor (CASP-1)
CCNF	NM_001761	PH_hs_0000262	899	chr16	1.205953075	0.000534538	G2/mitotic-specific cyclin-F [Source:UniProtKB/Swiss-Prot;Acc:P41002]
CCNL2	NA	PH_hs_0027861	NA	chr1	1.149538348	6.66739E-06	Cyclin-L2 (Paneth cell-enhanced expression protein) [Source:UniProtKB/Swiss-Prot;Acc:Q96S94]
CDK5RAP3	NM_176096	PH_hs_0001754	80279	chr17	1.138157469	0.001057039	CDK5 regulatory subunit-associated protein 3 (CDK5 activator-binding protein C53)(Protein HSF-27)
CDKN2B	NM_078487	PH_hs_0004184	1030	chr9	1.048806041	7.14195E-07	Cyclin-dependent kinase 4 inhibitor B (p14-INK4b)(p15-INK4b)(p15INK4B)(Multiple tumor suppressor 2)(MTS-2)
CEBPB	NM_005194	PH_hs_0005496	1051	chr20	1.316034756	0.000177985	CCAAT/enhancer-binding protein beta (C/EBP beta)(Nuclear factor NF-IL6)(Transcription factor 5)
CEBPG	NM_001806	PH_hs_0000293	1054	chr19	1.530893933	9.93655E-06	CCAAT/enhancer-binding protein gamma (C/EBP gamma)
CFDP1	NM_006324	PH_hs_0020118	10428	chr16	1.817533016	0.000314304	Craniofacial development protein 1 (Bucentaur)
CHD1L	NM_004284	PH_hs_0025208	9557	chr1	1.01305464	0.004878163	Chromodomain-helicase-DNA-binding protein 1-like
CHD9	NM_025134	PH_hs_0031574	80205	chr16	1.407782806	0.004651546	Chromodomain-helicase-DNA-binding protein 9
CHST11	NM_018413	PH_hs_0020084	50515	chr12	1.448243	0.002299224	Carbohydrate sulfotransferase 11
CLCN3	NM_173872	PH_hs_0024212	1182	chr4	1.352366206	0.000456454	Chloride channel protein 3 (ClC-3)
CLCN5	NA	PH_hs_0038365	NA	chrX	2.637934543	5.73126E-12	Chloride channel protein 5 (ClC-5)
CLCN6	NM_001286	PH_hs_0030766	1185	chr1	1.231750009	1.87397E-05	Chloride channel protein 6 (ClC-6)
CLCN7	NA	PH_hs_0004645	NA	chr16	1.780886741	4.88324E-15	Chloride channel protein 7 (ClC-7)
CLN3	NM_000086	PH_hs_0030338	1201	chr16	2.34062333	1.58057E-08	Battenin (Protein CLN3)(Batten disease protein)
CLOCK	NM_004898	PH_hs_0025553	9575	chr4	1.684352086	4.07922E-06	Circadian locomoter output cycles protein kaput (hCLOCK)
COL11A1	NM_080629	PH_hs_0025731	1301	chr1	1.530918881	0.000130036	Collagen alpha-1(XI) chain Precursor
COL18A1	NA	PH_hs_0024936	NA	chr21	1.201105485	0.001500493	Collagen alpha-1(XVIII) chain Precursor [Contains Endostatin] [Source:UniProtKB/Swiss-Prot;Acc:P39060]
COL4A3BP	NA	PH_hs_0001835	NA	chr5	1.6445846	6.022E-05	Collagen type IV alpha-3-binding protein (Ceramide transfer protein
COX10	NM_001303	PH_hs_0004469	1352	chr17	1.058603593	0.008742198	Protoheme IX farnesyltransferase, mitochondrial Precursor
CRISPLD2	NM_031476	PH_hs_0013642	83716	chr16	1.02580807	0.004736226	Cysteine-rich secretory protein LCCL domain-containing 2 Precursor

CSTB	NM_000100.2	PH_hs_0000080	1476	chr21	1.200624932	5.47643E-05	Cystatin-B (Stefin-B)(Liver thiol proteinase inhibitor)(CPI-B)
CTNS	NM_00103168	PH_hs_0022892	1497	chr17	2.127152647	7.33082E-16	Cystinosin
CTSD	NM_001909	PH_hs_0016632	1509	chr11	1.43604675	7.97138E-05	CD225 family protein FLJ76511
CUL4A	NM_00100889	PH_hs_0023344	8451	chr13	1.600502644	7.01387E-06	Cullin-4A (CUL-4A)
CYB561	NM_00101791	PH_hs_0017204	1534	chr17	1.872213936	1.17593E-12	Cytochrome b561 (Cytochrome b-561)
CYB5B	NM_030579	PH_hs_0025903	80777	chr16	1.092627226	0.000261262	Cytochrome b5 type B Precursor (Cytochrome b5 outer mitochondrial membrane isoform)
CYP51A1	NA	PH_hs_0033255	NA	chr7	1.189430772	2.965E-09	Leucine-rich repeat and death domain-containing protein LOC401387
DCBLD2	NM_080927	PH_hs_0031999	131566	chr3	1.899608551	1.36644E-06	Discoidin, CUB and LCCL domain-containing protein 2 Precursor
DCTN2	NM_006400	PH_hs_0023515	10540	chr12	1.583659974	0.000101839	Dynactin subunit 2 (Dynactin complex 50 kDa subunit)(DCTN-50)
DDAH1	NA	PH_hs_0018551	NA	chr1	1.024410964	0.000325322	N(G),N(G)-dimethylarginine dimethylaminohydrolase 1
DDIT3	NM_004083	PH_hs_0033101	1649	chr12	2.832507286	3.42843E-21	DNA damage-inducible transcript 3 (DDIT-3)
DEDD2	NM_133328	PH_hs_0015226	162989	chr19	1.010953438	0.006592583	DNA-binding death effector domain-containing protein 2
DERL3	NM_00100286	PH_hs_0031927	91319	chr22	1.862114231	0.002244751	Derlin-3 (Der1-like protein 3)(Degradation in endoplasmic reticulum protein 3
DFFB	NM_004402	PH_hs_0031751	1677	chr1	1.161522639	0.001371649	DNA fragmentation factor subunit beta
DGKE	NM_003647	PH_hs_0012903	8526	chr17	1.612114195	0.00255229	Diacylglycerol kinase epsilon (DAG kinase epsilon)
DHCR7	NM_001360	PH_hs_0001117	1717	chr11	1.715779611	4.12807E-05	7-dehydrocholesterol reductase (7-DHC reductase)
DHRS13	NM_144683	PH_hs_0027552	147015	chr17	1.171104169	8.79919E-05	Dehydrogenase/reductase SDR family member 13 Precursor
DICER1	NM_030621	PH_hs_0004928	23405	chr14	1.478510133	0.000473505	Endoribonuclease Dicer
DKK3	NM_00101805	PH_hs_0031712	27122	chr11	1.144625811	1.28459E-07	Dickkopf-related protein 3 Precursor (Dickkopf-3)
DLG5	NM_004747	PH_hs_0026534	9231	chr10	1.153025366	9.44953E-05	Disks large homolog 5 (Placenta and prostate DLG)
DNAJB12	NM_017626	PH_hs_0030954	54788	chr10	1.040271745	0.004735289	DnaJ homolog subfamily B member 12
DNAJB9	NM_012328	PH_hs_0000684	4189	chr7	1.790960704	1.19057E-05	DnaJ homolog subfamily B member 9
DNAJC10	NM_018981	PH_hs_0000143	54431	chr2	1.319307835	1.23053E-07	DnaJ homolog subfamily C member 10 Precursor
DNAJC12	NM_201262	PH_hs_0013621	56521	chr10	1.566427036	0.0008507	DnaJ homolog subfamily C member 12
DNAJC27	NM_016544	PH_hs_0025370	51277	chr2	1.016819159	0.004145564	DnaJ homolog subfamily C member 27
DNAJC3	NM_006260	PH_hs_0037639	5611	chr13	2.387227932	2.14901E-20	DnaJ homolog subfamily C member 3 Precursor
DNAJC6	NM_014787	PH_hs_0003254	9829	chr1	1.416745777	5.09538E-05	Putative tyrosine-protein phosphatase auxilin
DOK5	NM_018431	PH_hs_0007344	55816	chr20	1.366248251	0.001226608	Docking protein 5 (Downstream of tyrosine kinase 5)
DPAGT1	NM_001382	PH_hs_0002328	1798	chr11	1.3269533	3.0478E-07	UDP-N-acetylglucosamine--dolichyl-phosphate N-acetylglucosaminephosphotransferase
DUSP12	NM_007240	PH_hs_0025045	11266	chr1	1.089086648	0.00158509	Dual specificity protein phosphatase 12
DUSP15	NM_00101264	PH_hs_0018247	128853	chr20	1.103881878	0.004078553	Dual specificity protein phosphatase 15
DUSP16	NA	PH_hs_0001766	NA	chr12	1.285714758	8.5309E-08	Dual specificity protein phosphatase 16
DUSP6	NM_022652	PH_hs_0023584	1848	chr12	1.449265091	0.002141353	Dual specificity protein phosphatase 6
DUSP8	NM_004420	PH_hs_0026661	1850	chr11	1.955779834	6.84861E-10	Dual specificity protein phosphatase 8

DYRK1A	NM_130438	PH_hs_0004378	1859	chr21	1.286696949	0.001763197	Dual specificity tyrosine-phosphorylation-regulated kinase 1A
DYSF	NA	PH_hs_0021965	NA	chr2	2.206168254	2.34877E-06	Dysferlin (Dystrophy-associated fer-1-like protein)
E2F7	NM_203394	PH_hs_0025052	144455	chr12	1.333302245	9.0689E-05	Transcription factor E2F7 (E2F-7)
EI24	NM_00100727	PH_hs_0027383	9538	chr11	1.068503809	2.9062E-07	Etoposide-induced protein 2.4 homolog (p53-induced gene 8 protein)
EIF4EBP2	NM_004096	PH_hs_0014304	1979	chr10	1.653912591	1.41754E-14	Eukaryotic translation initiation factor 4E-binding protein 2 (eIF4E-binding protein 2)
EIF5	NM_183004	PH_hs_0030187	1983	chr14	1.352123356	0.000293591	Eukaryotic translation initiation factor 5 (eIF-5)
ELF4	NA	PH_hs_0009109	NA	chrX	1.136401089	0.000216498	ETS-related transcription factor Elf-4 (E74-like factor 4)
EML2	NM_012155	PH_hs_0002134	24139	chr19	1.771372729	3.99039E-10	Echinoderm microtubule-associated protein-like 2 (EMAP-2)(HuEMAP-2)
EMP3	NM_001425	PH_hs_0001032	2014	chr19	1.160562426	0.004484718	Epithelial membrane protein 3 (EMP-3)(Protein YMP)(Hematopoietic neural membrane protein)(HNMP-1)
ESCO1	NM_052911	PH_hs_0015077	114799	chr18	1.292915608	0.001183962	N-acetyltransferase ESCO1
ETNK1	NM_018638	PH_hs_0002556	55500	chr12	1.525227302	0.000581617	Ethanolamine kinase 1 (EKI 1)
EVC	NA	PH_hs_0022074	NA	chr4	1.138618915	0.001644178	Ellis-van Creveld syndrome protein (DWF-1)
EXOC4	NM_021807	PH_hs_0026072	60412	chr7	1.091661966	0.001310685	Exocyst complex component 4
F2R	NM_001992	PH_hs_0007383	2149	chr5	2.595832313	1.24431E-08	Proteinase-activated receptor 1 Precursor (PAR-1)
F2RL1	NM_005242	PH_hs_0009290	2150	chr5	1.190303665	2.12676E-07	Proteinase-activated receptor 2 Precursor (PAR-2)
F2RL2	NM_004101	PH_hs_0003115	2151	chr5	1.163792436	0.000233273	Proteinase-activated receptor 3 Precursor (PAR-3)
FABP3	NM_004102	PH_hs_0027773	2170	chr1	2.451695913	1.14547E-20	Fatty acid-binding protein, heart (Heart-type fatty acid-binding protein)
FADS1	NM_013402	PH_hs_0026554	3992	chr11	1.229546741	0.000189547	Fatty acid desaturase 1
FAF1	NM_007051	PH_hs_0031002	11124	chr1	1.371126831	0.004121926	FAS-associated factor 1 (hFAF1)
FAM129A	NM_052966	PH_hs_0027123	116496	chr1	1.334809042	1.05189E-08	Protein Niban (Protein FAM129A)
FANCG	NM_004629	PH_hs_0025352	2189	chr9	1.107737281	3.03722E-05	Fanconi anemia group G protein (Protein FACG)
FDFT1	NM_004462	PH_hs_0032086	2222	chr8	1.053843615	0.003124223	Squalene synthetase (SQS)
FKBP2	NA	PH_hs_0012659	NA	chr11	1.961908997	2.41772E-11	FK506-binding protein 2 Precursor
FLAD1	NM_201398	PH_hs_0006544	80308	chr1	1.05951985	3.57114E-05	FAD synthetase
FOLR3	NM_000804	PH_hs_0027518	2352	chr11	1.201702957	0.001583817	Folate receptor gamma Precursor (FR-gamma)(Folate receptor 3)
FOXM1	NM_202002	PH_hs_0026679	2305	chr12	1.077127504	0.000498128	Forkhead box protein M1 (Forkhead-related protein FKHL16)
FOXRED1	NM_017547	PH_hs_0023767	55572	chr11	1.092944131	0.00212178	FAD-dependent oxidoreductase domain-containing protein 1
FOXRED2	NM_00110237	PH_hs_0024429	80020	chr22	1.19533421	3.99634E-05	FAD-dependent oxidoreductase domain-containing protein 2 Precursor
FUCA2	NM_032020	PH_hs_0028786	2519	chr6	1.291528404	0.001857091	Plasma alpha-L-fucosidase Precursor
FUT8	NM_178157	PH_hs_0006549	2530	chr14	1.126981526	3.84854E-06	Alpha-(1,6)-fucosyltransferase (alpha1-6FucT)
FZD5	NM_003468	PH_hs_0009160	7855	chr2	1.187088603	6.27744E-08	Frizzled-5 Precursor (Fz-5)(hFz5)(FzE5)
FZD8	NM_031866	PH_hs_0015628	8325	chr10	1.529387432	0.004253728	Frizzled-8 Precursor (Fz-8)(hFz8)
GAB2	NM_080491	PH_hs_0002620	9846	chr11	1.399060506	0.000502773	GRB2-associated-binding protein 2
GART	NA	PH_hs_0004709	NA	chr21	1.135993007	0.002530783	Trifunctional purine biosynthetic protein adenosine-3

GCHFR	NM_005258	PH_hs_0023208	2644	chr15	1.31172286	4.45196E-06	GTP cyclohydrolase 1 feedback regulatory protein
GDAP1	NM_00104087	PH_hs_0040045	54332	chr8	1.944798231	4.69163E-09	Ganglioside-induced differentiation-associated protein 1 (GDAP1)
GFM1	NM_024996	PH_hs_0014174	85476	chr3	1.470399762	9.54402E-06	Elongation factor G 1, mitochondrial Precursor (mEF-G 1)
GFOD1	NA	PH_hs_0020427	NA	chr6	1.060200879	4.89983E-07	Glucose-fructose oxidoreductase domain-containing protein 1 Precursor
GFOD2	NM_030819	PH_hs_0026854	81577	chr16	1.050975652	0.005681491	Glucose-fructose oxidoreductase domain-containing protein 2 Precursor
GLCE	NM_015554	PH_hs_0032044	26035	chr15	1.341775557	0.000225639	D-glucuronyl C5-epimerase
GMDS	NM_001500	PH_hs_0005859	2762	chr6	1.504383299	1.44861E-10	GDP-mannose 4,6 dehydratase
GNA13	NM_006572	PH_hs_0001001	10672	chr17	1.686711282	1.73189E-07	Guanine nucleotide-binding protein subunit alpha-13 (G-protein subunit alpha-13)
GNB5	NM_006578	PH_hs_0009338	10681	chr15	1.557102414	0.001324575	Guanine nucleotide-binding protein subunit beta-5 (Transducin beta chain 5)
GNG4	NM_00109872	PH_hs_0026185	2786	chr1	2.027410154	1.20162E-06	Guanine nucleotide-binding protein G(I)/G(S)/G(O) subunit gamma-4 Precursor
GNPDA1	NM_005471	PH_hs_0014282	10007	chr5	2.205194477	1.18799E-11	Glucosamine-6-phosphate isomerase 1
GPD1L	NM_015141	PH_hs_0004724	23171	chr3	1.728722033	4.33708E-06	Glycerol-3-phosphate dehydrogenase 1-like protein
GRB10	NM_00100155	PH_hs_0031701	2887	chr7	1.077325608	0.00012691	Growth factor receptor-bound protein 10
GRPEL2	NM_152407	PH_hs_0028783	134266	chr5	1.681399277	1.18678E-08	GrpE protein homolog 2, mitochondrial Precursor (Mt-GrpE#2)
GSPT1	NA	PH_hs_0039013	NA	chr16	1.226590464	0.005646732	Eukaryotic peptide chain release factor GTP-binding subunit ERF3A
GTF2E1	NM_005513	PH_hs_0008629	2960	chr3	1.22353905	0.000820583	General transcription factor IIE subunit 1
GUSB	NM_000181	PH_hs_0023271	2990	chr7	1.324195026	0.005188891	Beta-glucuronidase Precursor
GYPC	NM_016815	PH_hs_0004683	2995	chr2	1.347282496	1.76452E-06	Glycophorin-C (PAS-2')(Glycoprotein beta)(GLPC)(Glycoconnectin)
HERPUD1	NM_00101099	PH_hs_0008735	9709	chr16	1.559688422	4.94275E-06	Homocysteine-responsive endoplasmic reticulum-resident ubiquitin-like domain member 1 protein
HEXA	NM_000520	PH_hs_0033214	3073	chr15	1.618830535	1.64864E-08	Beta-hexosaminidase subunit alpha Precursor
HEXB	NM_000521	PH_hs_0022175	3074	chr5	2.211012579	0.001292752	Beta-hexosaminidase subunit beta Precursor
HMGCR	NA	PH_hs_0001121	NA	chr5	1.407056732	1.1739E-05	3-hydroxy-3-methylglutaryl-coenzyme A reductase (HMG-CoA reductase)
HMOX1	NM_002133	PH_hs_0011943	3162	chr22	2.558727267	9.98257E-15	Heme oxygenase 1 (HO-1)
HOXA7	NM_006896	PH_hs_0038546	3204	chr7	1.213096011	0.001412952	Homeobox protein Hox-A7 (Hox-1A)(Hox 1.1)
HOXB5	NM_002147	PH_hs_0022664	3215	chr17	1.50290531	9.76095E-11	Homeobox protein Hox-B5 (Hox-2A)(HHO.C10)(HU-1)
HOXB6	NM_018952	PH_hs_0005427	3216	chr17	1.464820252	0.001308839	Homeobox protein Hox-B6 (Hox-2B)(Hox-2.2)(HU-2)
HOXB7	NM_004502	PH_hs_0025481	3217	chr17	1.066101347	0.003760447	Homeobox protein Hox-B7 (Hox-2C)(HHO.C1)
HSD17B14	NM_016246	PH_hs_0035373	51171	chr19	2.19354719	7.79847E-07	17-beta-hydroxysteroid dehydrogenase 14
HSD17B7	NA	PH_hs_0040570	NA	chr1	1.186700715	0.002425702	3-keto-steroid reductase
HSPA5	NM_005347	PH_hs_0023711	3309	chr9	4.114044407	3.18696E-21	78 kDa glucose-regulated protein Precursor (GRP 78)
HSPB2	NM_001541	PH_hs_0004545	3316	chr11	1.274449412	1.19442E-05	Heat shock protein beta-2 (HspB2)(DMPK-binding protein)(MKBP)
HYOU1	NA	PH_hs_0014249	NA	chr11	2.384608163	7.94466E-20	Hypoxia up-regulated protein 1 Precursor (150 kDa oxygen-regulated protein)
ICAM1	NM_000201	PH_hs_0024395	3383	chr19	1.100206955	4.05452E-05	Intercellular adhesion molecule 1 Precursor (ICAM-1)
IFI16	NM_005531	PH_hs_0027408	3428	chr1	1.288374323	0.000322229	Gamma-interferon-inducible protein Ifi-16

IFI30	NM_006332	PH_hs_0001383	10437	chr19	1.72959364	1.07409E-13	Gamma-interferon-inducible lysosomal thiol reductase Precursor
IFITM1	NM_003641	PH_hs_0026376	8519	chr11	1.231423803	0.001573357	Interferon-induced transmembrane protein 1
IL11	NM_000641	PH_hs_0000234	3589	chr19	1.383927386	1.36533E-08	Interleukin-11 Precursor (IL-11)
IL15	NM_172174	PH_hs_0010067	3600	chr4	1.282058348	0.000463156	Interleukin-15 Precursor (IL-15)
INTS3	NM_023015	PH_hs_0005894	65123	chr1	1.135817576	0.001132527	Integrator complex subunit 3 (Int3)
ITPR3	NM_0022242	PH_hs_0004470	3710	chr6	1.46198763	5.01718E-05	Inositol 1,4,5-trisphosphate receptor type 3
JUND	NM_005354	PH_hs_0000356	3727	chr19	1.256037177	1.74277E-06	Transcription factor jun-D
KATNB1	NM_005886	PH_hs_0014204	10300	chr16	1.131068986	0.0015999	Katanin p80 WD40-containing subunit B1
KCNS3	NM_002252	PH_hs_0019600	3790	chr2	1.332649629	0.001368586	Potassium voltage-gated channel subfamily S member 3
KCTD3	NM_016121	PH_hs_0012893	51133	chr1	1.442319265	5.71934E-07	BTB/POZ domain-containing protein KCTD3
KIAA1012	NM_014939	PH_hs_0031188	22878	chr18	1.215225822	0.002288624	Protein TRS85 homolog
KIDINS220	NM_0207382	PH_hs_0001022	57498	chr2	2.067074776	5.34616E-05	Kinase D-interacting substrate of 220 kDa
KIF21A	NM_0176412	PH_hs_0024231	55605	chr12	1.48342089	7.2645E-05	Kinesin-like protein KIF21A (Kinesin-like protein KIF2)
KIFC3	NA	PH_hs_0002033	NA	chr16	1.555729069	3.82438E-08	Kinesin-like protein KIFC3
KIT	NM_000222	PH_hs_0004671	3815	chr4	3.174191023	2.27331E-18	Mast/stem cell growth factor receptor Precursor (SCFR)
LAMA1	NM_005559	PH_hs_0023338	284217	chr18	1.289565208	0.002147738	Laminin subunit alpha-1 Precursor (Laminin A chain)
LAMA4	NM_00110520	PH_hs_0025617	3910	chr6	1.978511891	7.091E-05	Laminin subunit alpha-4 Precursor
LEMD3	NM_014319	PH_hs_0029077	23592	chr12	1.054487322	0.001336143	Inner nuclear membrane protein Man1 (LEM domain-containing protein 3)
LGALS3	NM_002306	PH_hs_0030094	3958	chr14	1.526199989	1.40717E-06	Galectin-3 (Galactose-specific lectin 3
LGALS3BP	NM_005567	PH_hs_0004589	3959	chr17	1.162856184	1.60505E-08	Galectin-3-binding protein Precursor
LIPA	NA	PH_hs_0004818	NA	chr10	1.537726398	9.37203E-14	Lysosomal acid lipase/cholesteryl ester hydrolase Precursor (LAL)
LONP1	NM_004793	PH_hs_0016877	9361	chr19	1.312168859	2.14372E-05	Lon protease homolog, mitochondrial Precursor
LPIN1	NM_145693	PH_hs_0004665	23175	chr2	1.229777611	1.64857E-05	Lipin-1
LRPPRC	NM_133259	PH_hs_0010771	10128	chr2	2.100017414	1.49041E-12	Leucine-rich PPR motif-containing protein, mitochondrial Precursor
LY96	NM_015364	PH_hs_0004099	23643	chr8	2.394153856	3.9366E-14	Lymphocyte antigen 96 Precursor (Protein MD-2)
MAD1L1	NM_00101383	PH_hs_0000759	8379	chr7	1.647678584	1.35517E-09	Mitotic spindle assembly checkpoint protein MAD1
MAN2A1	NM_002372	PH_hs_0020904	4124	chr5	1.569970288	2.04241E-07	Alpha-mannosidase 2
MAP1LC3A	NM_032514	PH_hs_0019488	84557	chr20	1.659144309	3.11519E-05	Microtubule-associated proteins 1A/1B light chain 3A Precursor
MAP2K1	NM_002755	PH_hs_0022483	5604	chr15	1.046233596	0.003100079	Dual specificity mitogen-activated protein kinase kinase 1
MAP3K5	NM_005923	PH_hs_0009153	4217	chr6	1.198825785	1.24231E-06	Mitogen-activated protein kinase kinase kinase 5
MAP3K9	NM_033141	PH_hs_0033407	4293	chr14	1.265708007	0.002606798	Mitogen-activated protein kinase kinase kinase 9
MAPK13	NM_002754	PH_hs_0010614	5603	chr6	1.167339946	0.002874594	Mitogen-activated protein kinase 13
MAPK14	NA	PH_hs_0020050	NA	chr6	1.037811565	0.002244945	Mitogen-activated protein kinase 14
MAPK3	NM_002746	PH_hs_0000106	5595	chr16	1.445172479	0.003833763	Mitogen-activated protein kinase 3

MAPK8IP2	NM_012324	PH_hs_0017290	23542	chr22	1.129476354	0.004139124	C-jun-amino-terminal kinase-interacting protein 2
MAPKAP1	NM_024117	PH_hs_0016132	79109	chr9	1.259220162	0.001446204	Target of rapamycin complex 2 subunit MAPKAP1
MAPKAPK2	NM_032960	PH_hs_0004270	9261	chr1	1.015771629	0.000545943	MAP kinase-activated protein kinase 2
MCM2	NM_004526	PH_hs_0003681	4171	chr3	1.131489443	6.44906E-05	DNA replication licensing factor MCM2
MCTS1	NA	PH_hs_0005693	NA	chrX	1.012761992	2.5792E-07	Malignant T cell amplified sequence 1 (MCT-1)
MFSD1	NM_022736	PH_hs_0000777	64747	chr3	1.153804508	1.25685E-07	Major facilitator superfamily domain-containing protein 1
MID1	NM_000381	PH_hs_0002447	4281	chrX	1.089441566	0.003190364	Midline-1
MKNK2	NM_017572	PH_hs_0026721	2872	chr19	1.093440819	0.003313686	MAP kinase-interacting serine/threonine-protein kinase 2
MLF1	NA	PH_hs_0004817	NA	chr3	1.209204176	0.000768475	Myeloid leukemia factor 1 (Myelodysplasia-myeloid leukemia factor 1)
MLPH	NM_024101	PH_hs_0015371	79083	chr2	2.244558927	2.10777E-10	Melanophilin (Exophilin-3)
MME	NM_007289	PH_hs_0023672	4311	chr3	1.940429902	0.009925371	Neprilysin
MOSC1	NM_022746	PH_hs_0022942	64757	chr1	1.070134261	0.000487083	MOSC domain-containing protein 1, mitochondrial Precursor
HMOX1	NM_002133	PH_hs_0011943	3162	chr22	2.558727267	9.98257E-15	Heme oxygenase 1 (HO-1)
MRPL52	NM_181307	PH_hs_0017250	122704	chr14	1.484398131	0.004472665	39S ribosomal protein L52, mitochondrial Precursor (L52mt)
MSN	NM_002444	PH_hs_0029317	4478	chrX	1.224600144	0.000253585	Moesin (Membrane-organizing extension spike protein)
MTHFR	NM_005957	PH_hs_0009177	4524	chr1	1.401864615	0.001621072	Methylenetetrahydrofolate reductase
MTM1	NM_000252	PH_hs_0024384	4534	chrX	1.369090476	0.000260957	Myotubularin
MTMR4	NM_004687	PH_hs_0008374	9110	chr17	1.391280201	4.34045E-06	Myotubularin-related protein 4
MTRR	NM_002454	PH_hs_0009249	4552	chr5	1.113066714	0.000313912	Methionine synthase reductase, mitochondrial Precursor (MSR)
MYST3	NM_00109941	PH_hs_0022213	7994	chr8	1.35573373	0.001577854	Histone acetyltransferase MYST3 (MYST protein 3)
NCK2	NM_00100472	PH_hs_0031477	8440	chr2	1.43947021	0.000141562	Cytoplasmic protein NCK2 (NCK adaptor protein 2)
NDUFV3	NM_00100150	PH_hs_0032130	4731	chr21	1.424408767	0.001046563	NADH dehydrogenase [ubiquinone] flavoprotein 3, mitochondrial Precursor
NEDD4L	NA	PH_hs_0023225	NA	chr18	1.932378336	3.33849E-11	E3 ubiquitin-protein ligase NEDD4-like
NEK6	NA	PH_hs_0024415	NA	chr9	1.665804412	0.000197609	Serine/threonine-protein kinase Nek6
NFAT5	NA	PH_hs_0004887	NA	chr16	1.369275311	4.76329E-05	Nuclear factor of activated T-cells 5 (NF-AT5)
NFIL3	NM_005384	PH_hs_0004587	4783	chr9	1.853029688	0.002629315	Nuclear factor interleukin-3-regulated protein
NIN	NM_020921	PH_hs_0024377	51199	chr14	1.075772772	0.002335518	Ninein (hNinein)(Glycogen synthase kinase 3 beta-interacting protein)(GSK3B-interacting protein)
NKIRAS1	NM_020345	PH_hs_0031998	28512	chr3	1.079312758	0.004241338	NF-kappa-B inhibitor-interacting Ras-like protein 1
NKX3-1	NM_006167	PH_hs_0003656	4824	chr8	1.203824849	0.00168924	Homeobox protein Nkx-3.1 (Homeobox protein NK-3 homolog A)
NMB	NM_205858	PH_hs_0024396	4828	chr15	1.199396822	0.004112495	Neuromedin-B Precursor
NOC3L	NM_022451	PH_hs_0004264	64318	chr10	1.782124455	1.86957E-08	Nucleolar complex protein 3 homolog (NOC3 protein homolog)
NR2F2	NA	PH_hs_0016756	NA	chr15	1.272623734	0.003937378	COUP transcription factor 2 (COUP-TF II)
NR3C1	NM_00102409	PH_hs_0026745	2908	chr5	1.155725031	0.00340945	Glucocorticoid receptor (GR)(Nuclear receptor subfamily 3 group C member 1)
NT5C	NM_014595	PH_hs_0004068	30833	chr17	1.120741876	0.000557527	5'(3')-deoxyribonucleotidase, cytosolic type

NUP188	NM_015354	PH_hs_0022534	23511	chr9	1.522520116	0.003837306	Nucleoporin NUP188 homolog (hNup188)
OGDH	NM_002541	PH_hs_0031965	4967	chr7	1.025226537	0.004265843	2-oxoglutarate dehydrogenase E1 component, mitochondrial Precursor
OLFML2A	NM_182487	PH_hs_0024008	169611	chr9	1.32596101	9.36299E-09	Olfactomedin-like protein 2A Precursor (Photomedin-1)
OPA1	NM_130837	PH_hs_0022462	4976	chr3	1.061980027	1.62879E-06	Dynamin-like 120 kDa protein, mitochondrial Precursor (Optic atrophy protein 1)
OPTN	NM_021980	PH_hs_0024518	10133	chr10	1.088362654	2.99738E-05	Optineurin (Optic neuropathy-inducing protein)
P2RX4	NM_002560	PH_hs_0032657	5025	chr12	1.907201546	0.000181635	P2X purinoceptor 4 (P2X4)(ATP receptor)
P2RX5	NM_175080	PH_hs_0024759	5026	chr17	1.463924095	1.91776E-05	P2X purinoceptor 5 (P2X5)(ATP receptor)
PDIA4	NM_004911	PH_hs_0005142	9601	chr7	3.037887792	8.85977E-13	Protein disulfide-isomerase A4 Precursor
PDXK	NM_003681	PH_hs_0033908	8566	chr21	2.136453212	3.64893E-07	Pyridoxal kinase
PES1	NM_014303	PH_hs_0022478	23481	chr22	1.203206189	0.000941152	Pescadillo homolog
PIM3	NM_00100185	PH_hs_0034689	415116	chr22	1.312856326	9.05861E-08	Serine/threonine-protein kinase Pim-3
PKD2	NM_000297	PH_hs_0026338	5311	chr4	1.131413115	1.1125E-05	Polycystin-2 (Polycystic kidney disease 2 protein)
PKN1	NM_213560	PH_hs_0000335	5585	chr19	1.343294927	0.000531573	Serine/threonine-protein kinase N1
PLA2G16	NA	PH_hs_0034609	NA	chr11	1.464953408	1.59278E-10	HRAS-like suppressor 3
PLAU	NA	PH_hs_0031336	NA	chr10	1.805259641	4.14893E-13	Urokinase-type plasminogen activator Precursor (U-plasminogen activator)(uPA)
PLEK2	NM_016445	PH_hs_0022812	26499	chr14	1.076210755	0.007142103	Pleckstrin-2
PODXL	NM_005397	PH_hs_0001519	5420	chr7	2.474508158	1.23815E-08	Podocalyxin-like protein 1 Precursor
POMT2	NM_013382	PH_hs_0007985	29954	chr14	1.4452992	6.65571E-07	Protein O-mannosyl-transferase 2
PPARG	NM_015869	PH_hs_0024432	5468	chr3	2.145263128	1.72456E-06	Peroxisome proliferator-activated receptor gamma
PPARGC1A	NM_013261	PH_hs_0011689	10891	chr4	1.868723353	1.17093E-06	Peroxisome proliferator-activated receptor gamma coactivator 1-alpha (PPAR-gamma coactivator 1-alpha)
PPIF	NM_005729	PH_hs_0018569	10105	chr10	1.821245165	3.02001E-10	Peptidyl-prolyl cis-trans isomerase, mitochondrial Precursor (PPIase)
PPT1	NA	PH_hs_0030902	NA	chr1	2.303207222	3.60804E-12	Palmitoyl-protein thioesterase 1 Precursor (PPT-1)
PSME1	NM_176783	PH_hs_0035453	5720	chr14	1.101834733	3.82183E-05	Proteasome activator complex subunit 1 (Proteasome activator 28 subunit alpha)
PTPN3	NA	PH_hs_0024660	NA	chr9	1.127982131	0.000228395	Tyrosine-protein phosphatase non-receptor type 3
PTPRE	NM_006504	PH_hs_0025649	5791	chr10	1.023733699	0.003179533	Receptor-type tyrosine-protein phosphatase epsilon Precursor
PTPRF	NM_002840	PH_hs_0004288	5792	chr1	1.014850621	0.00082125	Receptor-type tyrosine-protein phosphatase F Precursor
PTPRK	NA	PH_hs_0023965	NA	chr6	1.440771283	0.000213621	Receptor-type tyrosine-protein phosphatase kappa Precursor (Protein-tyrosine phosphatase kappa)
PTX3	NM_002852	PH_hs_0000274	5806	chr3	1.151332249	0.000608554	Pentraxin-related protein PTX3 Precursor (Pentaxin-related protein PTX3)
PUS7	NM_019042	PH_hs_0032971	54517	chr7	1.237672064	0.001216184	Pseudouridylate synthase 7 homolog
PYGB	NM_002862	PH_hs_0022609	5834	chr20	1.313908211	7.15125E-11	Glycogen phosphorylase, brain form
PYGL	NM_002863	PH_hs_0023452	5836	chr14	1.117548197	6.50611E-06	Glycogen phosphorylase, liver form
QDPR	NM_000320	PH_hs_0004327	5860	chr4	2.574477166	0.003055506	Dihydropteridine reductase
QPRT	NM_014298	PH_hs_0023865	23475	chr16	1.42694191	6.43115E-06	Nicotinate-nucleotide pyrophosphorylase
RAB31	NM_006868	PH_hs_0012499	11031	chr18	1.017581169	0.001158457	Ras-related protein Rab-31 (Rab-22B)

RAB33A	NM_004794	PH_hs_0003671	9363	chrX	1.311550894	1.44627E-05	Ras-related protein Rab-33A (Small GTP-binding protein S10)
RAB38	NM_022337	PH_hs_0006020	23682	chr11	1.219514059	1.94419E-05	Ras-related protein Rab-38 (Antigen NY-MEL-1)]
RAB5B	NM_002868	PH_hs_0004481	5869	chr12	1.366347151	7.87821E-11	Ras-related protein Rab-5B
RABEP1	NM_004703	PH_hs_0024519	9135	chr17	1.093691983	1.17779E-05	Rab GTPase-binding effector protein 1 (Rabaptin-5)
RAC1	NM_018890	PH_hs_0033233	5879	chr7	1.096146124	0.001209931	Ras-related C3 botulinum toxin substrate 1 Precursor (p21-Rac1)
RAD51	NM_002875	PH_hs_0026109	5888	chr15	1.142037325	5.64505E-09	DNA repair protein RAD51 homolog 1 (hRAD51)
RAP2A	NM_021033	PH_hs_0031220	5911	chr13	1.226260988	2.00164E-06	Ras-related protein Rap-2a Precursor (RbBP-30)
RAPGEF2	NM_014247	PH_hs_0018074	9693	chr4	1.202040747	0.000274088	Rap guanine nucleotide exchange factor 2 (Neural RAP guanine nucleotide exchange protein)
RASL10B	NM_033315	PH_hs_0020624	91608	chr17	2.017172045	1.67667E-06	Ras-like protein family member 10B Precursor
RC3H1	NM_172071	PH_hs_0002599	149041	chr1	1.060056435	0.000103449	Roquin (RING finger and C3H zinc finger protein 1)
RELN	NM_173054	PH_hs_0025219	5649	chr7	1.345911035	7.25066E-05	Reelin Precursor (EC 3.4.21.-)
RGMB	NM_00101276	PH_hs_0039269	285704	chr5	1.163721848	0.005412095	RGM domain family member B Precursor
RGS10	NM_00100533	PH_hs_0031365	6001	chr10	1.359134421	0.001170271	Regulator of G-protein signaling 10 (RGS10)
RGS7	NM_002924	PH_hs_0004592	6000	chr1	1.487505875	0.000466368	Regulator of G-protein signaling 7 (RGS7)
RHEBL1	NM_144593	PH_hs_0009654	121268	chr12	1.082079147	0.000233265	GTPase RhebL1 Precursor (Ras homolog enriched in brain-like protein 1)
RHOT1	NM_00103356	PH_hs_0038716	55288	chr17	1.080280493	7.74369E-05	Mitochondrial Rho GTPase 1 (hMiro-1)(MIRO-1)
RND3	NM_005168	PH_hs_0000694	390	chr2	1.907806047	2.00901E-08	Rho-related GTP-binding protein RhoE Precursor (Rho family GTPase 3)
ROCK2	NM_004850	PH_hs_0003771	9475	chr2	1.215936652	0.000403569	Rho-associated protein kinase 2
RPL7L1	NA	PH_hs_0039021	NA	chr6	1.107167431	7.45775E-05	60S ribosomal protein L7-like 1
RPS4Y2	NM_00103956	PH_hs_0032876	140032	chrY	1.092522087	0.002183732	40S ribosomal protein S4, Y isoform 2
RRAGC	NM_022157	PH_hs_0006103	64121	chr1	2.070334883	3.34042E-10	Ras-related GTP-binding protein C (Rag C)(RagC)
SACM1L	NM_014016	PH_hs_0000594	22908	chr3	1.431405575	0.002520666	Phosphatidylinositide phosphatase SAC1
SCAMP1	NM_004866	PH_hs_0002661	9522	chr5	1.3053754	1.70795E-05	Secretory carrier-associated membrane protein 1
SCD	NM_005063	PH_hs_0037413	6319	chr10	2.721413121	1.02162E-08	Acyl-CoA desaturase
SCFD2	NM_152540	PH_hs_0003403	152579	chr4	1.396752985	0.000203978	Sec1 family domain-containing protein 2
SCO1	NM_0045892	PH_hs_0001375	6341	chr17	1.260061178	0.000280105	Protein SCO1 homolog, mitochondrial Precursor
SEC24A	NM_021982	PH_hs_0012278	10802	chr5	1.197712518	0.003156187	Protein transport protein Sec24A (SEC24-related protein A)
SEC61G	NM_00101245	PH_hs_0001028	23480	chr7	1.141085231	0.000680462	Protein transport protein Sec61 subunit gamma
SEH1L	NM_00101343	PH_hs_0015504	81929	chr18	1.468830693	0.00094804	Nucleoporin SEH1-like (SEC13-like protein)
SEMA3F	NM_004186	PH_hs_0002778	6405	chr3	1.454559573	1.05597E-10	Semaphorin-3F Precursor (Semaphorin IV)(Sema IV)(Sema III/F)
SEMA7A	NA	PH_hs_0010222	NA	chr15	1.898310448	4.3096E-06	Semaphorin-7A Precursor (Semaphorin-L)
SERP1	NM_014445	PH_hs_0025770	27230	chr3	1.407145933	2.40016E-09	Stress-associated endoplasmic reticulum protein 1 (Ribosome-attached membrane protein 4)
SH2B3	NM_005475	PH_hs_0001260	10019	chr12	1.854343036	1.68877E-16	SH2B adapter protein 3 (Lymphocyte-specific adapter protein Lnk)
SLC16A8	NM_013356	PH_hs_0013901	23539	chr22	1.069317798	0.001164649	Monocarboxylate transporter 3 (MCT 3)(Solute carrier family 16 member 8)

SLC17A5	NM_012434	PH_hs_0006462	26503	chr6	2.157947159	8.96256E-05	Sialin (Solute carrier family 17 member 5)(Sodium/sialic acid cotransporter)(AST)
SLC25A19	NA	PH_hs_0024557	NA	chr17	1.37251651	3.74596E-09	Mitochondrial thiamine pyrophosphate carrier (Solute carrier family 25 member 19)
SLC25A37	NM_016612	PH_hs_0014670	51312	chr8	1.351099784	7.47314E-10	Mitoferrin-1 (Mitochondrial iron transporter 1)(Solute carrier family 25 member 37)
SLC25A38	NM_017875	PH_hs_0000846	54977	chr3	1.10011914	6.42777E-05	Solute carrier family 25 member 38
SLC31A1	NM_001859	PH_hs_0019597	1317	chr9	1.231234313	0.000842894	High affinity copper uptake protein 1 (Copper transporter 1)
SLC35D2	NM_007001	PH_hs_0024386	11046	chr9	1.342437776	4.60697E-07	UDP-N-acetylglucosamine/UDP-glucose/GDP-mannose transporter
SLC3A2	NM_002394	PH_hs_0027162	6520	chr11	1.464356431	8.62542E-06	4F2 cell-surface antigen heavy chain (4F2hc)(Lymphocyte activation antigen 4F2 large subunit)
SLC40A1	NM_014585	PH_hs_0024808	30061	chr2	2.208406274	0.000211809	Solute carrier family 40 member 1 (Ferroportin-1)(Iron-regulated transporter 1)
SLC5A6	NM_021095	PH_hs_0020432	8884	chr2	1.414668734	3.84397E-05	Sodium-dependent multivitamin transporter (Na(+)-dependent multivitamin transporter)
SLC6A8	NA	PH_hs_0030003	NA	chrX	1.592989546	3.00019E-07	Sodium- and chloride-dependent creatine transporter 1 (Creatine transporter 1)
SLC9A6	NM_006359	PH_hs_0003938	10479	chrX	1.020228759	0.003899395	Sodium/hydrogen exchanger 6 (Na(+)/H(+) exchanger 6)
SLK	NM_014720	PH_hs_0005882	9748	chr10	1.051939626	0.003468485	STE20-like serine/threonine-protein kinase (STE20-like kinase
SMARCA4	NA	PH_hs_0004515	NA	chr19	1.092016688	0.001625828	Probable global transcription activator SNF2L4
SNCA	NA	PH_hs_0004441	NA	chr4	1.323098391	0.00299974	Alpha-synuclein (Non-A beta component of AD amyloid)
SNX6	NM_021249	PH_hs_0027344	58533	chr14	1.133552529	0.000196584	Sorting nexin-6 (TRAF4-associated factor 2)
SOCS1	NM_003745	PH_hs_0003492	8651	chr16	1.214813285	0.0027347	Suppressor of cytokine signaling 1 (SOCS-1)(JAK-binding protein)
SOX9	NM_000346	PH_hs_0028708	6662	chr17	1.065746529	0.000294294	Transcription factor SOX-9
SPG21	NA	PH_hs_0035716	NA	chr15	1.22851198	0.000404694	Maspardin (Spastic paraplegia 21 autosomal recessive Mast syndrome protein)
SPHK1	NA	PH_hs_0004080	NA	chr17	1.150688642	3.34301E-06	Sphingosine kinase 1 (SPK 1)
SPNS1	NA	PH_hs_0040014	NA	chr16	1.625817739	3.96744E-09	Protein spinster homolog 1 (Spinster-like protein 1)
SPP1	NM_00104006	PH_hs_0028680	6696	chr4	2.125745641	1.60013E-07	Osteopontin Precursor (Bone sialoprotein 1)
SQSTM1	NA	PH_hs_0010763	NA	chr5	1.756496813	7.1052E-06	Sequestosome-1 (Phosphotyrosine-independent ligand for the Lck SH2 domain of 62 kDa)
STEAP3	NM_018234	PH_hs_0003687	55240	chr2	2.967343517	3.24768E-13	Metalloreductase STEAP3
STX3	NM_004177	PH_hs_0004706	6809	chr11	3.520706592	6.89451E-12	Syntaxin-3
SUPV3L1	NM_003171	PH_hs_0005923	6832	chr10	2.218043842	1.18597E-05	ATP-dependent RNA helicase SUPV3L1, mitochondrial Precursor
SURF1	NM_003172	PH_hs_0033280	6834	chr9	1.031319564	0.005641901	Surfeit locus protein 1
SUZ12	NM_015355	PH_hs_0023866	23512	chr17	1.119942045	1.79564E-06	Polycomb protein SUZ12 (Suppressor of zeste 12 protein homolog)
TACC2	NM_206861	PH_hs_0028808	10579	chr10	1.334039112	4.3732E-06	Transforming acidic coiled-coil-containing protein 2 (Anti Zuai-1)
TAF4	NM_003185	PH_hs_0002168	6874	chr20	1.51163068	0.001354843	Transcription initiation factor TFIID subunit 4 (TBP-associated factor 4)
TBL1XR1	NM_024665	PH_hs_0033440	79718	chr3	1.179035449	0.00201215	F-box-like/WD repeat-containing protein TBL1XR1 (Transducin beta-like 1X-related protein 1)
TBX3	NM_016569	PH_hs_0024082	6926	chr12	1.726608954	2.76864E-09	T-box transcription factor TBX3 (T-box protein 3)
TDG	NM_003211	PH_hs_0030709	6996	chr12	1.04729479	1.1403E-05	G/T mismatch-specific thymine DNA glycosylase
TIMELESS	NM_003920	PH_hs_0006539	8914	chr12	1.297677466	8.02769E-06	Protein timeless homolog (hTIM)
TIMP3	NM_000362	PH_hs_0013028	7078	chr22	2.316060346	5.74486E-14	Metalloproteinase inhibitor 3 Precursor (Tissue inhibitor of metalloproteinases 3)(TIMP-3)

TNIK	NA	PH_hs_0038528	NA	chr3	1.319106663	0.000479886	TRAF2 and NCK-interacting protein kinase
TOLLIP	NM_019009	PH_hs_0002235	54472	chr11	1.072654532	0.000131016	Toll-interacting protein
TOM1	NA	PH_hs_0000980	NA	chr22	1.249051255	7.55356E-06	Target of Myb protein 1
TOMM20	NM_014765	PH_hs_0004283	9804	chr1	1.353584516	0.000604122	Mitochondrial import receptor subunit TOM20 homolog (Mitochondrial 20 kDa outer membrane protein)
TOMM40L	NM_032174	PH_hs_0016061	84134	chr1	1.25710629	0.005427663	Mitochondrial import receptor subunit TOM40B (Protein TOMM40-like)
TPM1	NM_00101800	PH_hs_0022510	7168	chr15	1.093137975	6.65263E-05	Tropomyosin alpha-1 chain (Tropomyosin-1)(Alpha-tropomyosin)
TPP1	NM_000391	PH_hs_0025203	1200	chr11	2.648896547	7.51419E-06	Tripeptidyl-peptidase 1 Precursor (TPP-1)
TRAF7	NM_032271	PH_hs_0023801	84231	chr16	1.493243389	1.15033E-06	E3 ubiquitin-protein ligase TRAF7
TRAK2	NM_015049	PH_hs_0009286	66008	chr2	1.796227963	1.01064E-05	Trafficking kinesin-binding protein 2 (Amyotrophic lateral sclerosis 2 chromosomal region candidate gene 3 protein)
TRIM33	NM_033020	PH_hs_0023549	51592	chr1	1.244117335	0.000136883	E3 ubiquitin-protein ligase TRIM33
TRPC1	NM_003304	PH_hs_0013274	7220	chr3	1.20850117	0.001540282	Short transient receptor potential channel 1 (TrpC1)
TRPM7	NM_017672	PH_hs_0031800	54822	chr15	1.557318732	0.001356161	Transient receptor potential cation channel subfamily M member 7
TRPS1	NM_014112	PH_hs_0002304	7227	chr8	1.129906218	0.001276637	Zinc finger transcription factor Trps1 (Tricho-rhino-phalangeal syndrome type I protein)
TRPV2	NM_016113	PH_hs_0014393	51393	chr17	1.693856198	2.88572E-13	Transient receptor potential cation channel subfamily V member 2 (TrpV2)
TSC1	NA	PH_hs_0022176	NA	chr9	1.344797667	0.00093758	Hamartin (Tuberous sclerosis 1 protein)
TTL	NM_153712	PH_hs_0032861	150465	chr2	1.188816567	0.001123027	Tubulin--tyrosine ligase (TTL)(EC 6.3.2.25)
TUBB2A	NM_001069	PH_hs_0026825	7280	chr6	1.023975559	0.004117168	Tubulin beta-2A chain
TUBB2B	NM_178012	PH_hs_0023602	347733	chr6	1.324092957	0.00099438	Tubulin beta-2B chain
TULP4	NM_00100746	PH_hs_0005668	56995	chr6	1.637639767	0.001626774	Tubby-related protein 4 (Tubby-like protein 4)
TXN2	NM_012473	PH_hs_0012289	25828	chr22	1.061695597	1.86562E-07	Thioredoxin, mitochondrial Precursor (Mt-Trx)(MTRX)(Thioredoxin-2)
UBE2E1	NM_003341	PH_hs_0009892	7324	chr3	1.05707155	0.003325202	Ubiquitin-conjugating enzyme E2 E1
UBE2H	NM_003344	PH_hs_0018037	7328	chr7	1.035937373	0.001705469	Ubiquitin-conjugating enzyme E2 H
UBP1	NA	PH_hs_0002485	NA	chr3	1.362549865	8.30142E-06	Upstream-binding protein 1 (LBP-1)
UHRF2	NM_152896	PH_hs_0024552	115426	chr9	1.19627383	0.000491561	E3 ubiquitin-protein ligase UHRF2
UTP20	NM_0145032	PH_hs_0023565	27340	chr12	1.057193122	0.000491229	Small subunit processome component 20 homolog (Down-regulated in metastasis protein)
UVRAG	NM_003369	PH_hs_0001261	7405	chr11	1.252068317	0.000781998	UV radiation resistance-associated gene protein (p63)
VAT1	NM_006373	PH_hs_0009515	10493	chr17	1.166785935	1.07947E-06	Synaptic vesicle membrane protein VAT-1 homolog
VEGFB	NM_003377	PH_hs_0004543	7423	chr11	1.128797601	6.15529E-05	Vascular endothelial growth factor B Precursor (VEGF-B)(VEGF-related factor)
VPS13A	NM_033305	PH_hs_0003548	23230	chr9	1.153558489	0.001737344	Vacuolar protein sorting-associated protein 13A (Chorein)
WASF3	NM_006646	PH_hs_0004713	10810	chr13	1.410862536	5.54765E-09	Wiskott-Aldrich syndrome protein family member 3 (WASP family protein member 3)
WDR4	NM_018669	PH_hs_0008457	10785	chr21	1.240260547	0.007938827	tRNA (guanine-N(7)-)-methyltransferase subunit WDR4 (WD repeat-containing protein 4)
XIAP	NM_001167	PH_hs_0023989	331	chrX	1.756753027	1.49154E-05	Baculoviral IAP repeat-containing protein 4
XPR1	NA	PH_hs_0000463	NA	chr1	1.198482152	0.001782211	Xenotropic and polytropic retrovirus receptor 1 (Protein SYG1 homolog)
ZNF12	NA	PH_hs_0031479	7559	chr7	1.149451128	0.000205203	Zinc finger protein 12 (Zinc finger protein KOX3)(Zinc finger protein 325)
